# Synthesis of 6-Membered-Ring Fused Thiazine-Dicarboxylates and Thiazole-Pyrimidines via One-Pot Three-Component Reactions

**DOI:** 10.3390/molecules26185493

**Published:** 2021-09-10

**Authors:** Reagan L. Mohlala, Elena Mabel Coyanis, Muhammad Q. Fish, Manuel A. Fernandes, Moira L. Bode

**Affiliations:** 1 Advanced Materials Division, Mintek, Private Bag X3015, Randburg 2125, South Africa; reaganm@mintek.co.za (R.L.M.); QasimF@mintek.co.za (M.Q.F.); 2 Molecular Sciences Institute, School of Chemistry, University of the Witwatersrand, Private Bag 3, PO Wits, Johannesburg 2050, South Africa; Manuel.Fernandes@wits.ac.za

**Keywords:** multicomponent reaction, isocyanides, dialkyl acetylenedicarboxylates, thiourea, α-haloketone, thiazine, thiazole-pyrimidines

## Abstract

A facile and efficient one-pot three-component reaction method for the synthesis of thiazine-dicarboxylates is reported. Reaction of an isocyanide and dialkyl acetylenedicarboxylate with 2-amino-4*H*-1,3-thiazin-4-one derivatives containing both an acidic proton and an internal nucleophile gave the products in good yields of 76–85%. The reactivity of dialkyl acetylenedicarboxylates was further tested in the synthesis of thiazole-pyrimidines where a two-component reaction of 2-aminothiazole with dialkyl acetylenedicarboxylates was successfully converted to a more efficient three-component reaction of a thiourea, α-haloketone and dialkyl acetylenedicarboxylate (DMAD/DEtAD) to give thiazole-pyrimidines in good yields of 70–91%.

## 1. Introduction

Multicomponent reactions (MCRs) are one-pot reactions that comprise at least three reactants [1,2,3]. Some of the well-known MCRs include the classic Ugi four-component reaction [4] (U-4CR), the Passerini three-component reaction [5] (P-3CR), the Biginelli three-component reaction [6] (B-3CR) and the Hantzsch three-component reaction [7] (H-3CR). The history and discovery of MCRs can be traced back to Strecker’s [8] multicomponent reaction (S-3CR), which was reported in 1850, and three decades on, the Hantzsch reaction for preparing dihydropyridines (DHP) was reported [7]. The Biginelli 3-CR was reported in 1891, whereas Mannich [9] reported his eponymous MCR in 1912. The first isocyanide-based MCRs were reported in 1921 and 1959 by Passerini [5] and Ugi [10], respectively. MCRs represent one of the most convenient synthetic approaches in chemistry, with the main advantages being a reduction in the number of sequential reaction steps required and often better yields [11,12]. The Passerini and Ugi reactions have gained a great deal of attention since the early 1990s [13,14]. The versatility of these reactions relies on the dual reactivity of the isocyanide carbon atom, leading to the formation of diverse and complex products [15,16].

**Scheme 1:**
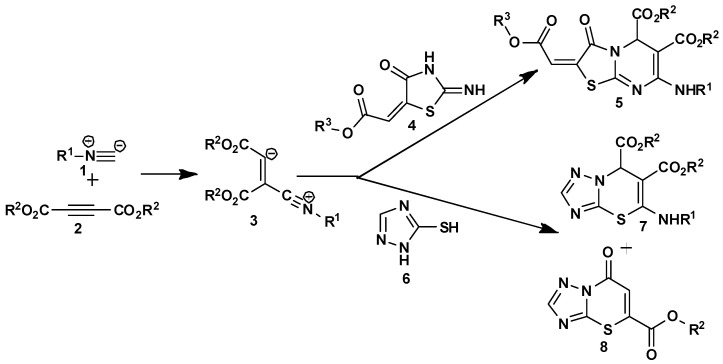
Our previous work showing synthesis of pyrimidine derivatives and their analogues.

The first reaction of isocyanides **1** with electron deficient alkynes such as DMAD **2** to give zwitterions **3** (Scheme 1) was reported by Winterfeldt and co-workers [17]. This zwitterion adduct **3** can readily undergo cycloaddition reactions with various third components, generating a range of heterocyclic scaffolds of interest [18]. Previously, our group reported the reaction of zwitterion adduct **3** and different five-membered rings, for example, **4**, containing both an acidic proton and internal nucleophile, to give products **5** incorporating all three reaction components (Scheme 1). Depending on the nature of the five-membered ring, in certain cases such as for thiol **6**, we observed that there was competition between the three-component reaction (to give **7**) and the two-component reaction (to give **8**). For the latter, DMAD **2** reacts directly with the five-membered ring, as shown in Scheme 1 for 1*H-*1,2,4-triazole-5-thiol **6**, to give methyl 7-oxo-7*H*-[1,2,4]triazolo[5,1-*b*][1,3]thiazine-5-carboxylate **8** [19]. We found that the most successful three-component reactions involved five-membered rings containing more acidic protons.

In this work, we set out to investigate alternative rings to act as the third component substrate in these reactions and selected the acidic six-membered ring 2-amino-4*H*-1,3-thiazin-4-one derivatives **9**, with predicted pK_a_ values in the range of −1.80 to −2.21. Thiazines are bioactive scaffolds with a wide range of pharmacological activities, such as antimicrobial [20,21], anti-inflammatory [22], anticancer [23], antitubercular [24] and antiviral [25] activities. Another aim of this work was the further investigation of the two-component reactions between various five-membered rings and DMAD to prepare compounds such as **8** and their analogues. Of particular interest to us were the thiazole-pyrimidine derivatives, which display interesting biological activity. Compounds derived from thiazolopyrimidines are known to exhibit antioxidant and antitumor activities [26]. Derivatives containing a thiazole-pyrimidine moiety as part of a more complex structure were shown to possess activity against *Mycobacterium tuberculosis* [27], while thiazolopyrimidine containing compounds ritanserin [28] and setoperone [29] have been used in the study of psychiatric disorders.

We initially investigated the direct reactivity of dialkyl acetylenedicarboxylate towards 2-aminothiazoles for the preparation of thiazole-pyrimidine derivatives and subsequently converted this two-component reaction into a one-pot, three-component reaction of dialkyl acetylenedicarboxylate with thiourea and an α-haloketone for the formation of thiazole-pyrimidines. Here we present a facile and efficient one-pot, three component reaction method for the synthesis of novel dihydropyrimido-thiazine-6,7-dicarboxylates from an isocyanide, dialkyl acetylenedicarboxylate and 2-amino-4*H*-1,3-thiazin-4-one derivatives; and the synthesis of 5*H*-thiazolo[3,2-*a*]pyrimidine-7-carboxylates from thiourea, an α-haloketone and dialkyl acetylenedicarboxylates.

## 2. Results and Discussion

### 2.1. Synthesis of 4-Oxo-4,6-Dihydropyrimido[2,1-b][1,3]Thiazine-6,7-Dicarboxylates by Three-Component Reaction

**Figure 1:**
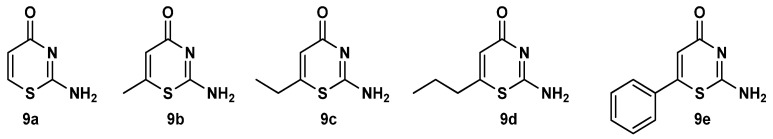
Six-membered rings **9a–e** chosen for the three-component reaction.

The six-membered rings **9a–e**, which were selected for use in these three-component reactions, are shown in Figure 1. To the best of our knowledge, none of these compounds has previously been tested in reaction with acetylenedicarboxylates **2** and isocyanides **1**. In addition, compounds **9c** and **9d** have not been previously reported. The initial reaction of 2-amino-4*H*-1,3-thiazin-4-one **9a** [30] with isocyanide **1a** and acetylene dicarboxylates **2a–b** resulted in products **10a–b** (Table 1). This success encouraged us to further test the reaction of 2-amino-6-methyl-4*H*-1,3-thiazin-4-one **9b** [31], 2-amino-6-ethyl-4*H*-1,3-thiazin-4-one **9c**, 2-amino-6-propyl-4*H*-1,3-thiazin-4-one **9d** and 2-amino-6-phenyl-4*H*-1,3-thiazin-4-one **9e [31]** with the zwitterion adduct formed by the reaction of aliphatic isocyanides **1a–c** and acetylenedicarboxylates **2a–b**. Generally, the reactions were carried out by the addition of isocyanide **1** to acetylenedicarboxylate **2** at 0 °C under inert conditions, as shown in Table 1, followed by slow introduction of compound **9a–e** as a solution in dry dichloromethane to the reaction mixture at room temperature. After purification, novel products **10a–u** were obtained in good yields (Table 1). When selected reactions were tested using the green solvents ethanol and isopropanol instead of dichloromethane, low yields of thiazine compounds **10** were obtained. When acetone was employed as a solvent for the synthesis of thiazine compounds **10a–u**, using starting materials **9a–e**, average yields were obtained. Nonetheless, acetone can be considered as an alternative greener solvent to afford thiazine compounds **10a–u** using a three-component reaction. Compound **10o** gave the highest yield in acetone of 58%, while the lowest yield obtained was for **10g**, at 41%.

**Scheme 2:**
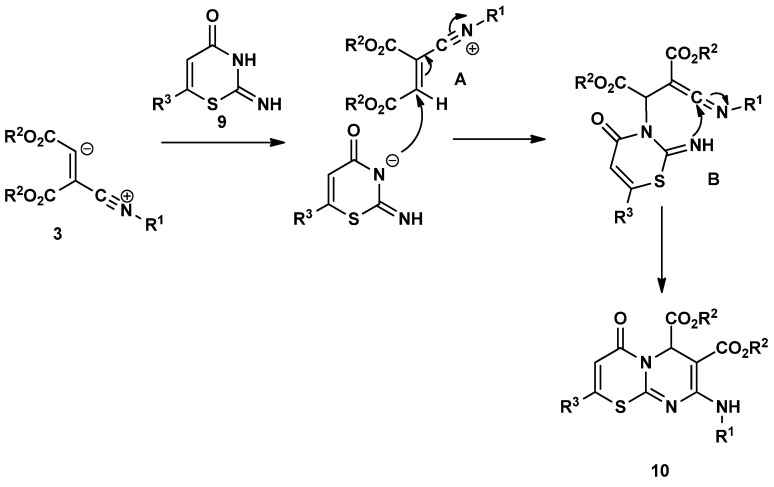
Proposed reaction mechanism for the formation of **10**.

Based on our previous results, we believe the very good yields obtained for **10a–u** were due to the presence of an acidic proton on the 2-amino-4*H*-1,3-thiazin-4-one derivatives **9a–e** which enables the reaction with the zwitterion adduct to occur readily in dichloromethane. The highest yields obtained were 85% for **10b**, **10h** and **10n** and the lowest yield was 76% for **10h**. Based on the isolated yields it is clear that the 2-amino-4*H*-1,3-thiazin-4-one derivatives **9** were completely inert towards direct reaction with either dialkyl acetylenedicarboxylates **2** or alkyl isocyanides **1**, while they demonstrated very high reactivity towards the zwitterion adduct. Although we have not definitively established the mechanism for the formation of **10**, a plausible mechanism for this reaction is proposed (after Esmaeili and co-workers [32]) and shown in Scheme 2. Zwitterion adduct **3** deprotonates **9**, followed by 1,4-nucleophilic attack on the nitrilium ion A. The resulting ketenimine intermediate B cyclizes to afford compound **10**.

### 2.2. Synthesis of 5H-Thiazolo[3,2-a]Pyrimidine-7-Carboxylates Using Two-Component and Three-Component Reactions

**Scheme 3:**
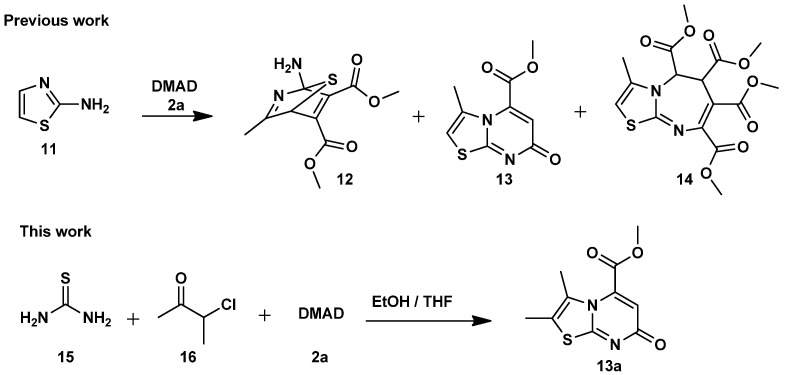
Synthesis of thiazole-pyrimidines **13**.

We went on to investigate the direct reaction of various five-membered ring substrates with electron deficient alkynes (DMAD/DEtAD). This study was started when we noticed the presence of co-products resulting from direct reaction of DMAD with some of the five-membered rings when we were attempting three-component reactions including an isocyanide [19]. Crank and co-workers [33] previously reported the reaction of 4-methylthiazol-2-amine **11** with DMAD **2** to afford Diels–Alder adduct **12**, methyl 3-methyl-7-oxo-7*H*-thiazolo[3,2-*a*]pyrimidine-5-carboxylate **13** and product **14**, resulting from reaction with two moles of DMAD (Scheme 3). Acheson and Wallis [34] also reported the direct reaction of 2-aminothiazole **11** and DMAD **2** using methanol under reflux to obtain methyl 7-oxo-7*H*-thiazolo[3,2-*a*]pyrimidine-5-carboxylate **13**. Thiazolopyrimidines can be obtained using two component reactions, under various conditions, starting from 2-aminothiazole derivatives and Michael acceptors, such as vinyl trichloromethylketones [35], ethyl acetoacetate [36], bis(2,4,6-trichlorophenyl) malonate [37], diethylmalonate, ethyl cyanoacetate, cyanoacetamide, ketonitriles and DMAD [38]. Our first approach was to react DMAD **2a** and pre-prepared five-membered ring substrates **11** directly to obtain fused 5-membered ring thiazole-pyrimidines **13**. However, the moderate yields achieved using this approach led us to optimize the preparation as a one-pot, three-component reaction from thiourea **15,** α-haloketones **16** and DMAD or DEtAD. To the best of our knowledge, this is the first report of such a one-pot preparation.

**Figure 2:**
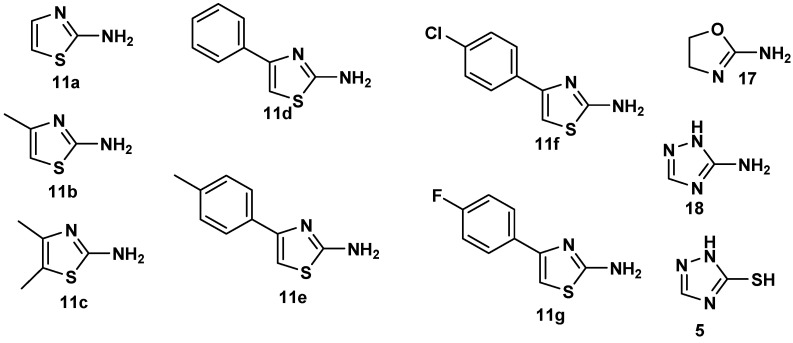
Five membered-ring substrates chosen for the reactions.

Initially, in order to achieve thiazole-pyrimidines **13**, we prepared 5-membered ring substrates **11a–g** [39,40,41,42] (Figure 2) to react with DMAD **2a**. The reactions were carried out by treating **11c** and **11e–g** with **2a** under ethanol reflux conditions to obtain **13a–d** (Method A). Repetition of these reactions using DEtAD **2b** afforded **13e–i**, whilst di-*tert*-butyl acetylenedicarboxylate (DTAD) **2c** and substrate **11a** gave rise to **13j** (Table 2). Despite the disappointing yields, the success of this reaction led us to extend it to the use of alternative five-membered ring reactants **17 [43]**, **18** [44] and **5** [45], with these substrates giving rise to compounds **19a–b**, **20** and **8**, respectively, in good yields.

Using Method A, low-to-average yields were obtained for most of the products **13**. The highest yield from this set of compounds was for **13g** with 79% yield and **13f** with 71%. However, when using substrates **11d–g** with phenyl groups at **R^1^** the yields dropped slightly due to the weakly activating phenyl ring and the deactivating groups on the phenyl ring (such as fluorine and chlorine) for both DMAD and DEtAD reactions. The presence of fluorine and chlorine at the *para*-position of substrates **11f** and **11g** resulted in a slight drop in yield for compounds **13c**, **13d**, **13h** and **13i** compared to **13b**, with a methyl substituent at the *para*-position. The reaction of dialkyl acetylenedicarboxylate **2c** and substrate **11a** gave a 70% yield of product **13j**. When substrate **17** was treated with dialkyl acetylenedicarboxylates **2a–b** the reaction gave 82% and 80% yield of **19a** and **19b**, respectively. Substrates **5** and **18** were also treated with DEtAD **2b** and gave rise to compounds **8** and **20** in good yields of 88% and 82%, respectively. When comparing the reactivity of the substrates it is evident that substrates **5**, **17** and **18** were more reactive towards **2a–b** than **11** under these conditions, giving consistently better yields.

The disappointing yields obtained from the direct reaction of substrates **11a–h** with dialkyl acetylenedicarboxylates **2a–b** prompted us to apply a second approach via a 3-CR to give product **13** (Method B). The reactions were carried out by initially mixing thiourea **15** with α-haloketone **16**, followed by introduction of dialkyl acetylenedicarboxylate **2** in a 1:1 ratio of ethanol and THF at room temperature, after which the reactions were heated to ethanol reflux temperature for at least 12 h. This gave rise to products **13a–d** in improved yields compared to the first approach (Table 3). The reactions were repeated using DEtAD **2b** and gave rise to products **13e–i** as expected. Room temperature reactions resulted in incomplete reactions, with recovery of some of the starting materials, even after 48 h. The three-component reaction (3-CR) was thus found to be suitable for the synthesis of products **13a–i**, which were isolated in good yields of 70–91%, based on the nature of the functional groups in the reactants **16a–e** and **2a–b**, as shown in Table 3. The highest yields for this set of compounds, using DMAD **2a**, was 82% for **13a**. The percentage yields of compounds **13b–d** were found to be slightly lower when compared to **13a**. Even starting materials substituted with fluorine or chlorine at the *para*-position (**16c** and **16d**) gave very good yields of product. Yields increased when using DEtAD **2b** rather than DMAD **2a**.

**Scheme 4:**
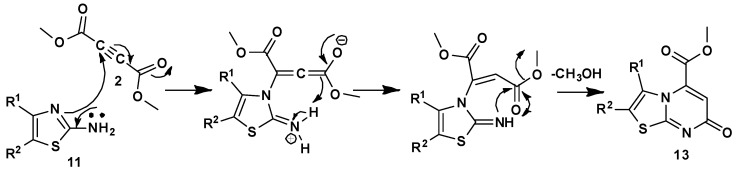
Proposed reaction mechanism for the formation of compounds **13a–j**.

Scheme 4 shows the proposed mechanism for the formation of compound **13** (see Acheson and Wallis [34]). The reaction proceeds by initial nucleophilic attack of the ring-nitrogen of substrate **11** on the α,β-unsaturated ester of DMAD forming an intermediate which undergoes internal cyclisation through nucleophilic attack of the side-chain nitrogen on the ester carbonyl carbon, followed by the loss of methanol, to afford product **13**.

**Figure 3:**
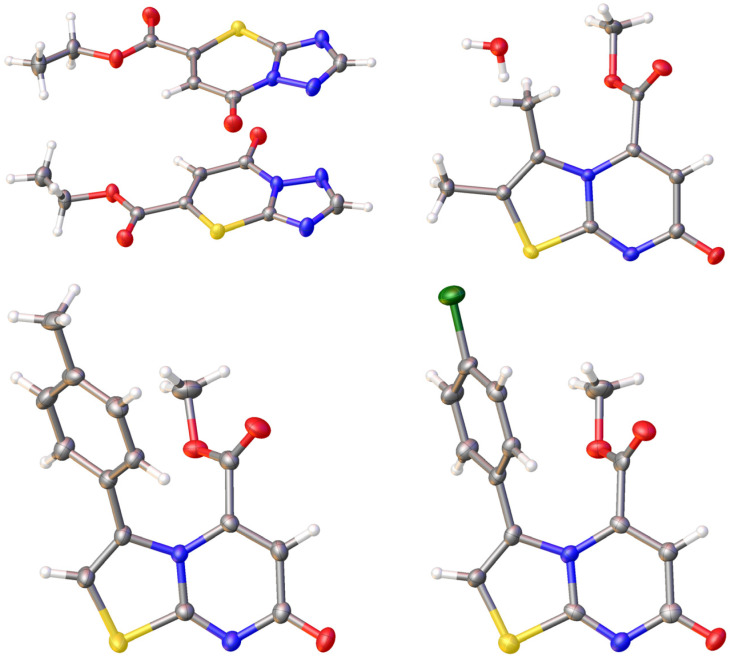
Single-crystal X-ray structure (ORTEP diagrams) of **8** (**top left**), **13a** (**top right**), **13b** (**bottom left**) and **13d** (**bottom right**). Carbon atoms are shown as gray spheres, nitrogen atoms as blue spheres, oxygen atoms as red spheres, sulfur atoms as yellow spheres, chlorine atoms as green spheres and hydrogen atoms as white spheres.

The structures of the synthesized compounds were confirmed by mass spectrometry, NMR spectroscopy and FTIR spectroscopy (Supplementary Materials). Explicit confirmation for the structures of **13a**, **13b**, **13d** and **8** was obtained from single-crystal X-ray analysis (Figure 3).

These compounds were prepared as part of our ongoing efforts towards identifying novel heterocycles with biological activity [46,47,48]. Compounds showing cytotoxicity values of >200 µM in MT-4 cells were evaluated for activity in an HIV assay (see Supplementary Materials). Unfortunately, these compounds were not found to be active as antiviral agents. However, due to the relatively high cytotoxicity values seen for some of the compounds, the possibility of their application as anticancer agents is being further investigated.

## 3. Materials and Methods

### 3.1. Chemical Synthesis

#### 3.1.1. General Information

All commercially available reagents were supplied by Sigma Aldrich (Schnelldorf, Germany) and used without further purification. Dry solvents were used directly from an LC-Tech SP-1 Solvent Purification System (LC Technology Solutions Inc., Seabrook, TX, USA) stored under argon. All solvents used for chromatographic purposes were supplied by RadChem (Johannesburg, South Africa) and were used without further distillation. NMR spectra were recorded at 298 K on a 400 MHz Bruker Avance Spectrometer (Bruker BioSpin, Rheinstetten, Germany). Chemical shifts are reported in ppm and are referenced internally to residual solvent resonances [7.26 (CDCl_3_) and 2.50 (DMSO-*d_6_*) for ^1^H NMR; 77.16 (CDCl_3_) 40.45 (DMSO-*d_6_*) for ^13^C NMR]. For mass spectrometry (LC–MS/MS), high-resolution mass spectra were obtained. Fourier infrared spectra (FTIR) were recorded by using a Perkin Elmer FTIR Spectrometer Spectrum Two (Perkin Elmer, Midrand, South Africa). All signals are reported on the wavenumber scale (*v*/cm^−1^).

#### 3.1.2. General Synthesis of 4-Oxo-4,6-Dihydropyrimido[2,1-*b*][1,3]Thiazine-6,7-Dicarboxylate Derivatives (**10a–u**)

In a round-bottomed flask equipped with a magnetic stirrer, isocyanide derivative (**1a–c**) in dry CH_2_Cl_2_ (2.5 mL) was slowly added to DMAD (**2a**) or DEtAD (**2b**) in dry CH_2_ Cl_2_ (5 mL) at 0 °C, under inert argon, and the solution was allowed to stir for 0.5 h. Then 2-amino-4*H*-1,3-thiazin-4-one derivatives **9a–e** in dry CH_2_Cl_2_ (2.5 mL) were slowly added to the solution and stirred at room temperature under argon for 6–24 h. The solvent was removed under vacuum, and the residue was purified by silica gel column chromatography, using hexane-ethyl acetate as eluent to afford the products.

*Dimethyl 8-(tert-butylamino)-4-oxo-4,6-dihydropyrimido[2,1-b][1,3]thiazine-6,7-dicarboxylate* (**10a**); 2-amino-4*H*-1,3-thiazin-4-one (**9a**) (0.25 g, 1.95 mmol), *tert*-butyl isocyanide (**1a**) (0.16 g, 1.95 mmol) and dimethyl acetylenedicarboxylate (**2a**) (0.28 g, 1.95 mmol). Physical characteristics: yellow solid; Yield: 0.55 g, 81%; Mp: 145–147 °C, **R_f_**: 0.5, hexane/ethyl acetate (90%:10%); ^1^H NMR (400 MHz, CDCl_3_) δ 8.73 (1H, br s, NH), 7.36 (1H, d, *J* = 10.8 Hz, Ar-CH), 6.51 (1H, d, *J* = 10.4 Hz, Ar-CH), 6.35 (1H, s, CH), 3.75 (3H, s, OCH_3_), 3.71 (3H, s, OCH_3_), 1.42 (9H, s, C(CH_3_)_3_); ^13^C NMR (101 MHz, CDCl_3_) δ 170.9 (C=O), 168.4 (C=O), 160.7 (C=O), 157.5 (C-10), 154.9 (C-8), 136.8 (C-3), 118.2 (Ar-CH), 72.3 (C-7), 52.9 (C-6), 52.7 (OCH_3_), 51.1 (OCH_3_), 50.8(C(CH_3_)_3_), 30.9 (C(CH_3_)_3_); FTIR ν_max_/cm^−1^: 2982, 2913, 1728, 1690, 1648, 1600, 1533, 1472, 1431; HRMS (ESI–TOF) *m*/*z*: [M + H]^+^ calculated for C_15_H_20_N_3_O_5_S^+^ 354.1118 found 354.1113.

*Diethyl 8-(tert-butylamino)-4-oxo-4,6-dihydropyrimido[2,1-b][1,3]thiazine-6,7-dicarboxylate* (**10b**); 2-amino-4*H*-1,3-thiazin-4-one (**9a**) (0.25 g, 1.95 mmol), *tert*-butyl isocyanide (**1a**) (0.16 g, 1.95 mmol) and diethyl acetylenedicarboxylate (**2b**) (0.33 g, 1.95 mmol). Physical characteristics: yellow solid; Yield: 0.63 g, 85%; Mp: 143–145 °C, R_f_: 0.6, hexane/ethyl acetate (90%:10%); ^1^H NMR (400 MHz, CDCl_3_) δ 8.75 (1H, br s, NH), 7.34 (1H, d, *J* = 10.8 Hz, Ar-CH), 6.51 (1H, d, *J* = 10.4 Hz, Ar-CH), 6.34 (1H, s, CH), 4.31–4.11 (4H, m, 2 × CH_2_), 1.43 (9H, s, C(CH_3_)_3_), 1.33 (3H, t, *J* = 7.2 Hz, CH_3_), 1.26 (3H, t, *J* = 7.2 Hz, CH_3_); ^13^C NMR (101 MHz, CDCl_3_) δ 170.6 (C=O), 168.1 (C=O), 160.8 (C=O), 157.3 (C-10), 154.8 (C-8), 136.7 (C-3), 118.2 (Ar-CH), 72.5 (C-7), 61.9 (O-CH_2_), 59.3 (O-CH_2_), 52.6 (C-6),) 51.3(C(CH_3_)_3_, 30.9 (C(CH_3_)_3_, 14.9(CH_3_), 14.2 (CH_3_); FTIR ν_max_/cm^−1^: 3067, 2983, 2864, 1728, 1690, 1647, 1599, 1534, 1431; HRMS (ESI–TOF) *m*/*z*: [M + H]^+^ calculated for C_17_H_24_N_3_O_5_S^+^ 382.1431 found 382.1439.

*Dimethyl 8-(tert-butylamino)-2-methyl-4-oxo-4,6-dihydropyrimido[2,1-b][1,3]thiazine-6,7-dicarboxylate* (**10c**); 2-amino-6-methyl-4*H*-1,3-thiazin-4-one (**9b**) (0.25 g, 1.76 mmol), *tert*-butyl isocyanide (**1a**) (0.15 g, 1.76 mmol) and dimethyl acetylenedicarboxylate (**2a**) (0.25 g, 1.76 mmol). Physical characteristics: yellow solid; Yield: 0.53 g, 82%; Mp: 149–151 °C, **R_f_**: 0.5, hexane/ethyl acetate (90%:10%); ^1^H NMR (400 MHz, CDCl_3_) δ 8.63 (1H, br s, NH), 6.24 and 6.23 (2H, 2 × s, Ar-CH and H-6), 3.66 (3H, s, O-CH_3_), 3.61 (3H, s, O-CH_3_), 2.17 (Ar-CH_3_), 1.33 (9H, s, C(CH_3_)_3_); ^13^C NMR (101 MHz, CDCl_3_) δ 171.1 (C=O), 168.5 (C=O), 161.2 (C=O), 157.8 (C-10), 155.3 (C-8), 149.7 (C-2), 116.1 (C-3), 72.1 (C-7), 52.9 (C-6), 52.6 (OCH_3_), 51.0 (OCH_3_), 50.8 (C(CH_3_)_3_), 30.8 (C(CH_3_)_3_) 22.1 (Ar-CH_3_); FTIR ν_max_/cm^−1^: 2992, 2963, 2902, 1734, 1648, 1682, 1653, 1602, 1525; HRMS (ESI–TOF) *m*/*z*: [M + H]^+^ calculated for C_16_H_22_N_3_O_5_S^+^ 368.1275 found 368.1279.

*Diethyl 8-(tert-butylamino)-2-methyl-4-oxo-4,6-dihydropyrimido[2,1-b][1,3]thiazine-6,7-dicarboxylate* (**10d**); 2-amino-6-methyl-4*H*-1,3-thiazin-4-one (**9b**) (0.25 g, 1.76 mmol), *tert*-butyl isocyanide (**1a**) (0.15 g, 1.76 mmol) and diethyl acetylenedicarboxylate (**2b**) (0.30 g, 1.76 mmol). Physical characteristics: yellow solid; Yield: 0.57 g, 83%; Mp: 135–137 °C, **R_f_**: 0.6, hexane/ethyl acetate (90%:10%); ^1^H NMR (400 MHz, CDCl_3_) δ 8.72 (1H, br s, NH), 6.28 (2H, 2 × s, Ar-CH and H-6), 4.27–4.06 (4H, m, 2 × CH_2_), 2.22 (3H, Ar-CH_3_), 1.39 (9H, s, C(CH_3_)_3_), 1.29 (3H, t, *J* = 7.2 Hz, CH_3_), 1.22 (3H, t, *J* = 7.2 Hz, CH_3_); ^13^C NMR (101 MHz, CDCl_3_) δ 170.7 (C=O), 168.2 (C=O), 161.4 (C=O), 157.7 (C-10), 155.2 (C-8), 149.7 (C-2), 116.1 (C-3), 72.4 (C-7), 61.8 (O-CH_2_), 59.2 (O-CH_2_), 52.6 (C-6), 51.3 (N-C(CH_3_)_3_), 30.9 (C(CH_3_)_3_, 22.1 (Ar-CH_3_), 14.9 (CH_3_), 14.2 (CH_3_); FTIR ν_max_/cm^−1^: 3285, 2975, 2890, 1735, 1690, 1643, 1600, 1535, 14377; HRMS (ESI–TOF) *m*/*z*: [M + H]^+^ calculated for C_18_H_26_N_3_O_5_S^+^ 396.1588 found 396.1597.

*Dimethyl 2-methyl-4-oxo-8-((2,4,4-trimethylpentan-2-yl)amino)-4,6-dihydropyrimido [2,1-b][1,3]thiazine-6,7-dicarboxylate* (**10e**); 2-Amino-6-methyl-4H-1,3-thiazin-4-one (**9b**) (0.25 g, 1.76 mmol), 1,1,3,3-tetramethylbutyl isocyanide (**1b**) (0.25 g, 1.76 mmol) and dimethyl acetylenedicarboxylate (**2a**) (0.25 g, 1.76 mmol). Physical characteristics: yellow solid; Yield: 0.56 g, 76%; Mp: 114–116 °C, R_f_: 0.6, hexane/ethyl acetate (90%:10%); ^1^H NMR (400 MHz, CDCl_3_) δ 8.74 (1H, br s, NH), 6.34 and 6.33 (2H, 2 × s, Ar-CH and H-6), 3.76 (3H, s, OCH_3_), 3.69 (3H, s, OCH_3_), 2.27 (3H, Ar-CH_3_), 1.83 (CH_2_), 1.48 and 1.46 (6H, 2 × s, 2CH_3_), 0.97 (9H, s, C(CH_3_)_3_); ^13^C NMR (101 MHz, CDCl_3_) δ 171.1 (C=O), 168.5 (C=O), 161.3 (C=O), 157.6 (C-10), 155.4 (C-8), 149.8, (C-2), 116.1 (C-3), 72.0 (C-7), 56.2 (C(CH_3_)_3_), 53.3 (CH_2_), 52.9 (C-6), 51.1 (OCH_3_), 50.8 (OCH_3_), 31.7 (C(CH_2_)_2_), 31.8 (C(CH_3_)_3_), 31.6 (CH_3_), 31.5 (CH_3_), 22.1 (Ar-CH_3_); FTIR ν_max_/cm^−1^: 3285, 2951, 2899, 1739, 1687, 1649, 1599, 1535, 1442; HRMS (ESI–TOF) *m*/*z*: [M + H]^+^ calculated for C_20_H_30_N_3_O_5_S^+^ 424.1901 found 424.1906.

*Diethyl 2-methyl-4-oxo-8-((2,4,4-trimethylpentan-2-yl)amino)-4,6-dihydropyrimido[2,1-b][1,3]thiazine-6,7-dicarboxylate (***10f***)*; 2-amino-6-methyl-4*H*-1,3-thiazin-4-one (**9b**) (0.25 g, 1.76 mmol), 1,1,3,3-tetramethylbutyl isocyanide (**1b**) (0.25 g, 1.76 mmol) and dimethyl acetylenedicarboxylate (**2a**) (0.25 g, 1.76 mmol). Physical characteristics: yellow solid; Yield: 0.61 g, 77%; Mp: 112–114 °C, R_f_: 0.7, hexane/ethyl acetate (90%:10%); ^1^H NMR (400 MHz, CDCl_3_) δ 8.73 (1H, br s, NH), 6.29 (2H, 2 × s, Ar-CH, H-6), 4.26–4.01 (4H, m, 2 × CH_2_O) 2.23 (3H, s, Ar-CH_3_), 1.80 (2H, q, *J* = 14.4 Hz, CH_2_), 1.44 and 1.42 (6H, 2 × s, 2 × CH_3_), 1.31 (3H, t, *J* = 6.8 Hz, CH_3_), 1.21 (3H, t, *J* = 7.2 Hz, CH_3_), 0.94 (9H, s, C(CH_3_)_3_); ^13^C NMR (101 MHz, CDCl_3_) δ 170.7 (C=O), 168.2 (C=O), 161.4 (C=O), 157.4 (C-10), 155.3 (C-8), 149.6 (C-2), 116.1 (C-3), 72.3 (C-7), 61.8 (O-CH_2_), 59.1 (O-CH_2_), 56.2 (C-(CH_3_)_3_), 53.1 (CH_2_), 51.3 (C-6), 31.6 (C-(CH_3_)_3_), 22.1 (Ar-CH_3_), 14.9 (CH_3_CH_2_), 14.2 (CH_3_CH_2_); FTIR ν_max_/cm^−1^: 3285, 2975, 2890, 1735, 1690, 1643, 1600, 1535, 1477; HRMS (ESI–TOF) *m*/*z*: [M + H]^+^ calculated for C_22_H_34_N_3_O_5_S^+^ 452.2214 found 452.2223.

*Dimethyl 8-(cyclohexylamino)-2-methyl-4-oxo-4,6-dihydropyrimido[2,1-b][1,3]thiazine-6,7-dicarboxylate (***10g**); 2-amino-6-methyl-4*H*-1,3-thiazin-4-one (**9b**) (0.25 g, 1.76 mmol), cyclohexyl isocyanide (**1c**) (0.19 g, 1.76 mmol) and dimethyl acetylenedicarboxylate (**2a**) (0.25 g, 1.76 mmol). Physical characteristics: yellow solid; Yield: 0.69 g, 84%; Mp: 148–150 °C, R_f_: 0.42, hexane/ethyl acetate (90%:10%); ^1^H NMR (400 MHz, CDCl_3_) δ 8.52 (1H, br s, NH), 6.29 (2H, 2 × s, Ar-CH, H-6), 3.90 (1H, s N-CH), 3.72 (3H, s, OCH_3_), 3.67 (3H, s, OCH_3_), 2.23 (CH_3_, s, Ar-CH_3_), 1.93–1.70 (5H, m, cyclohexyl) 1.67–1.32 (5H, m, cyclohexyl); ^13^C NMR (101 MHz, CDCl_3_) δ 171.1 (C=O), 168.3 (C=O), 161.2 (C=O), 159.1 (C-10), 154.2 (C-8), 149.6 (C-2), 116.0 (C-3), 71.5 (C-7), 52.9 (O-CH_3_), 51.2 (C-6), 50.7 (O-CH_3_), 49.6 (CH-NH), 34.3 (CH_2_, cyclohexyl), 33.7 (CH_2_, cyclohexyl), 25.7 (CH_2_, cyclohexyl), 24.8 (CH_2_, cyclohexyl), 24.7 (CH_2_, cyclohexyl), 22.1 (Ar-CH_3_). FTIR ν_max_/cm^−1^: 3285, 2946, 2858, 1762, 1730, 1692, 1653, 1618, 1590, 1525, 1445; HRMS (ESI–TOF) *m*/*z*: [M + H]^+^ calculated for C_18_H_24_N_3_O_5_S^+^ 394.1431 found 394.1433.

*Diethyl 8-(cyclohexylamino)-2-methyl-4-oxo-4,6-dihydropyrimido[2,1-b][1,3]thiazine-6,7-dicarboxylate (***10h**); 2-amino-6-methyl-4*H*-1,3-thiazin-4-one (**9b**) (0.25 g, 1.76 mmol), cyclohexyl isocyanide (**1c**) (0.19 g, 1.76 mmol) and diethyl acetylenedicarboxylate (**2b**) (0.30 g, 1.76 mmol). Physical characteristics: yellow solid; Yield: 0.63 g, 85%; Mp: 170–172 °C, **R_f_**: 0.50, hexane/ethyl acetate (90%:10%); ^1^H NMR (400 MHz, CDCl_3_) δ 8.55 (1H, br s, NH), 6.28 (2H, 2 × s, Ar-CH, H-6), 4.27–4.09 (4H, m, 2 × CH_2_), 3.92 (1H, s, CH), 2.23 (3H, s, Ar-CH_3_), 1.93–1.69 (4H, m, cyclohexyl), 1.66–1.55 (4H, m, cyclohexyl), 1.41–1.33 (2H, m, cyclohexyl) 1.34 (3H, s, *J* = 7.2 Hz, CH_3_), 1.28 (3H, s, *J* = 6.8 Hz, CH_3_); ^13^C NMR (101 MHz, CDCl_3_) δ 170.7 (C=O), 168.2 (C=O), 161.3 (C=O), 158.9 (C-10), 154.1 (C-8), 149.6 (C-2), 115.9 (C-3), 71.8 (C-7), 61.7 (O-CH_2_), 59.1 (O-CH_2_), 51.4 (C-6), 49.6 (CH), 34.3(CH_2_, cyclohexyl), 33.7 (CH_2_, cyclohexyl), 25.7 (CH_2_, cyclohexyl), 24.8 (CH_2_, cyclohexyl), 24.7 (CH_2_, cyclohexyl), 22.1 (Ar-CH_3_), 14.9 (CH_2_CH_3_), 14.1 (CH_2_CH_3_); FTIR ν_max_/cm^−1^: 2992, 2945, 2867, 1735, 1689, 1648, 1588, 1540, 1440; HRMS (ESI–TOF) *m*/*z*: [M + H]^+^ calculated for C_20_H_28_N_3_O_5_S^+^ 422.1744 found 422.1712.

*Dimethyl 8-(tert-butylamino)-2-ethyl-4-oxo-4,6-dihydropyrimido[2,1-b][1,3]thiazine-6,7-dicarboxylate* (**10i**); 2-amino-6-ethyl-4*H*-1,3-thiazin-4-one (**9c**) (0.25 g, 1.60 mmol), *tert*-butyl isocyanide (**1a**) (0.13 g, 1.60 mmol) and dimethyl acetylenedicarboxylate (**2a**) (0.22 g, 1.60 mmol). Physical characteristics: yellow solid; Yield: 0.51 g, 84%; Mp: 135–137 °C, R_f_: 0.48, hexane/ethyl acetate (90%:10%); ^1^H NMR (400 MHz, CDCl_3_) δ 8.70 (1H, br s, NH), 6.31 (2H, 2 × s, Ar-CH, H-6), 3.73 (3H, s, OCH_3_), 3.68 (3H, s, OCH_3_), 2.50 (2H, q, *J* = 7.2 Hz, Ar-CH_2_), 1.40 (9H, s, C(CH_3_)_3_), 1.28 (3H, t, *J* = 7.6 Hz, CH_3_); ^13^C NMR (101 MHz, CDCl_3_) δ 171.2 (C=O), 168.5 (C=O), 161.5 (C=O), 157.9 (C-10), 155.8 (C-8), 155.3 (C-2), 114.4 (C-3), 72.1 (C-7), 52.9 (OCH_3_), 52.6 (C-(CH_3_)_3_), 51.2 (C-6), 50.8 (OCH_3_), 30.8 (C(CH_3_)_3_), 29.4 (CH_2_CH_3_), 12.5 (CH_2_CH_3_); FTIR ν_max_/cm^−1^: 3273, 2967, 2876, 1739, 1674, 1644, 1616, 1591, 1531, 1438; HRMS (ESI–TOF) *m*/*z*: [M + H]^+^ calculated for C_17_H_24_N_3_O_5_S^+^ 382.1430 found 382.1427.

*Diethyl 8-(tert-butylamino)-2-ethyl-4-oxo-4,6-dihydropyrimido[2,1-b][1,3]thiazine-6,7-dicarboxylate* (**10j**); 2-amino-6-ethyl-4*H*-1,3-thiazin-4-one (**9c**) (0.25 g, 1.60 mmol), *tert*-butyl isocyanide (**1a**) (0.13 g, 1.60 mmol) and diethyl acetylenedicarboxylate (**2b**) (0.27 g, 1.60 mmol). Physical characteristics: yellow solid; Yield: 0.52 g, 79%; Mp: 94–96 °C, R_f_: 0.6, hexane/ethyl acetate (90%:10%); ^1^H NMR (400 MHz, CDCl_3_) δ 8.72 (1H, br s, NH), 6.30 and 6.28 (2H, 2 × s, Ar-CH, H-6), 4.27–4.09 (4H, m, 2 × CH_2_), 2.50 (2H, q, *J* = 7.6 Hz, Ar-CH_2_), 1.40 (9H, s, C(CH_3_)_3_), 1.29–1.26 (6H, m, 2 × CH_3_); ^13^C NMR (101 MHz, CDCl_3_) δ 170.7 (C=O), 168.2 (C=O), 161.6 (C=O), 157.8 (C-10), 155.7 (C-8), 155.2 (C-2), 114.4 (C-3), 72.31 (C-7), 61.8 (O-CH_2_), 59.2 (O-CH_2_), 52.5 (C(CH_3_)_3_), 51.4 (C-6), 30.9 (C(CH_3_)_3,_ 29.3 (Ar-CH_2_), 14.9 (CH_3_), 14.2 (CH_3_), 12.5 (CH_3_); FTIR ν_max_/cm^−1^: 3285, 2975, 2890, 1735, 1690, 1643, 1600, 1535, 14377; HRMS (ESI–TOF) *m*/*z*: [M + H]^+^ calculated for C_19_H_28_N_3_O_5_S^+^ 410.1744 found 410.1749.

*Dimethyl 8-((2,4-dimethylpentan-2-yl)amino)-2-ethyl-4-oxo-4,6-dihydropyrimido[2,1-b][1,3]thiazine-6,7-dicarboxylate* (**10k**); 2-amino-6-ethyl-4*H*-1,3-thiazin-4-one (**9c**) (0.25 g, 1.60 mmol), 1,1,3,3-tetramethylbutyl isocyanide (**1b**) (0.22 g, 1.76 mmol) and dimethyl acetylenedicarboxylate (**2a**) (0.23 g, 1.60 mmol). Physical characteristics: yellow paste; Yield: 0.57 g, 81%, R_f_: 0.5, hexane/ethyl acetate (90%:10%); ^1^H NMR (400 MHz, CDCl_3_) δ 8.64 (1H, br s, NH), 6.25 and 6.24 (2H, 2 × s, Ar-CH, H-6), 3.66 (3H, s, OCH_3_), 3.59 (3H, s, OCH_3_), 2.44 (2H, q, *J* = 7.6 Hz, Ar-CH_2_), 1.73 (CH_2_), 1.38 and 1.36 (6H, 2 × s, 2 × CH_3_), 1.21 (3H, t, *J* = 7.2 Hz, CH_3_CH_2_), 0.87 (9H, s, C(CH_3_)_3_); ^13^C NMR (101 MHz, CDCl_3_) δ 171.1 (C=O), 168.4 (C=O), 161.4 (C=O), 157.7 (C-10), 155.8 (C-8), 155.4 (C-2), 114.3 (C-3), 71.9 (C-7), 56.2 (C(CH_3_)_3_), 53.3 (CH_2_), 52.8 (OCH_3_), 51.1 (C-6), 50.7 (OCH_3_), 31.8 (C(CH_3_)_3_), 31.7, 31.6 (2 × CH_3_) 29.3 (Ar-CH_2_), 12.4 (CH_3_); FTIR ν_max_/cm^−1^: 3285, 2950, 2902, 1780, 1738, 1687, 1649, 1598, 1532; HRMS (ESI–TOF) *m*/*z*: [M + H]^+^ calculated for C_21_H_32_N_3_O_5_S^+^ 438.2057 found 438.2021.

*Diethyl 8-((2,4-dimethylpentan-2-yl)amino)-2-ethyl-4-oxo-4,6-dihydropyrimido[2,1-b][1,3]thiazine-6,7-dicarboxylate* (**10l**); 2-amino-6-ethyl-4*H*-1,3-thiazin-4-one (**9c**) (0.25 g, 1.60 mmol), 1,1,3,3-tetramethylbutyl isocyanide (**1b**) (0.22 g, 1.60 mmol) and diethyl acetylenedicarboxylate (**2b**) (0.27 g, 1.60 mmol). Physical characteristics: yellow solid; Yield: 0.61 g, 81%; Mp: 98–100 °C, R_f_: 0.6, hexane-ethyl acetate (90%:10%); ^1^H NMR (400 MHz, CDCl_3_) δ 8.73 (1H, br s, NH), 6.30 and 6.29 (2H, 2 × s, Ar-CH, H-6), 4.26–4.08 (4H, m, 2 × CH_2_O), 2.50 (2H, q, *J* = 7.2 Hz, Ar-CH_2_), 1.80 (2H, q, *J* = 14.8 Hz, CH_2_), 1.45 and 1.42 (6H, 2 × s, (CH_3_)_2_-C), 1.31–1.25 (6H, m, 2 × OCH_2_CH_3_), 1.21 (3H, s, *J* = 7.2 Hz, ArCH_2_CH_3_), 0.94 (9H, s, C(CH_3_)_3_); ^13^C NMR (101 MHz, CDCl_3_) δ 170.7 (C=O), 168.2 (C=O), 161.6 (C=O), 157.6 (C-10), 155.7 (C-8), 155.3 (C-2), 114.4 (C-3), 72.2 (C-7), 61.8 (O-CH_2_), 59.1 (O-CH_2_), 56.1 (C(CH_3_)_3_), 53.3 (Ar-CH_2_), 51.4 (C-6), 31.7 (C(CH_3_)_2_), 29.3 (C-(CH_3_)_3_), 29.3 (CH_2_), 14.9 (CH_3_), 14.2 (CH_3_), 12.5 (CH_3_); FTIR ν_max_/cm^−1^: 3270, 2973, 2913, 1737, 1688, 1646, 1600, 1535, 1432; HRMS (ESI–TOF) *m*/*z*: [M + H]^+^ Calculated for C_23_H_36_N_3_O_5_S^+^ 466.2370 found 466.2333.

*Dimethyl 8-(tert-butylamino)-4-oxo-2-propyl-4,6-dihydropyrimido[2,1-b][1,3]thiazine-6,7-dicarboxylate* (**10m**); 2-amino-6-propyl-4*H*-1,3-thiazin-4-one (**9d**) (0.25 g, 1.47 mmol), *tert*-butyl isocyanide (**1a**) (0.12 g, 1.47 mmol) and dimethyl acetylenedicarboxylate (**2a**) (0.21 g, 1.47 mmol). Physical characteristics: yellow paste; Yield: 0.48 g, 84%, R_f_: 0.5, hexane/ethyl acetate (90%:10%); ^1^H NMR (400 MHz, CDCl_3_) δ 8.72 (1H, br s, NH), 6.32 (2H, s, Ar-CH, H-6), 3.74 (3H, s, OCH_3_), 3.69 (3H, s, OCH_3_), 2.46 (2H, t, *J* = 7.6 Hz, Ar-CH_2_), 1.71 (2H, sextet, *J* = 7.6 Hz, CH_2_), 1.42 (9H, s, C(CH_3_)_3_), 1.03 (3H, t, *J* = 7.2 Hz, CH_3_CH_2_); ^13^C NMR (101 MHz, CDCl_3_) δ 171.1 (C=O), 168.4 (C=O), 161.3 (C=O), 157.9 (C-10), 155.3 (C-8), 154.4 (C-2), 115.1 (C-3), 72.0 (C-7), 52.9 (OCH_3_), 52.6 (C(CH_3_)_3_), 51.1 (C-6), 50.8 (OCH_3_), 37.9 (C(CH_3_)_3_), 30.8 (Ar-CH_2_), 21.6 (CH_3_CH_2_) 13.4 (CH_3_); FTIR ν_max_/cm^−1^: 3282, 2960, 2916, 1739, 1685, 1647, 1597, 1567, 1531, 1441; HRMS (ESI–TOF) *m*/*z*: [M + H]^+^ calculated for C_18_H_26_N_3_O_5_S^+^ 396.1588 found 396.1545.

*Diethyl 8-(tert-butylamino)-4-oxo-2-propyl-4,6-dihydropyrimido[2,1-b][1,3]thiazine-6,7-dicarboxylate* (**10n**); 2-amino-6-propyl-4*H*-1,3-thiazin-4-one (**9d**) (0.25 g, 1.47 mmol), *tert*-butyl isocyanide (**1a**) (0.12 g, 1.47 mmol) and diethyl acetylenedicarboxylate (**2b**) (0.25 g, 1.60 mmol). Physical characteristics: yellow paste; Yield: 0.53 g, 85%, R_f_: 0.56, hexane/ethyl acetate (90%:10%); ^1^H NMR (400 MHz, CDCl_3_) δ 8.74 (1H, br s, NH), 6.30 (2H, s, Ar-CH, H-6), 4.29–4.13 (4H, m, 2 × CH_2_), 2.46 (2H, t, *J* = 7.6 Hz, Ar-CH_2_), 1.71 (2H, sextet, *J* = 7.2 Hz, CH_2_), 1.42 (9H, s, C(CH_3_)_3_), 1.34–1.32 (6H, m, 2 × CH_3_), 1.32 (3H, t, *J* = 7.2 Hz, CH_3_); ^13^C NMR (101 MHz, CDCl_3_) δ 170.7 (C=O), 168.2 (C=O), 161.5 (C=O), 157.8 (C-10), 155.2 (C-8), 154.3 (C-2), 115.2 (C-3), 72.3 (C-7), 61.7 (O-CH_2_), 59.1 (O-CH_2_), 52.5 (C(CH_3_)_3_), 51.3 (C-6), 38.0 (Ar-CH_2_)_,_ 30.8 (C(CH_3_)_3,_ 21.6 (CH_3_CH_2_), 14.9, 14.1 and 13.4 (3 × CH_3_); FTIR ν_max_/cm^−1^: 3285, 2975, 2910, 2870, 1737, 1687, 1646, 1600, 1535, 1432; HRMS (ESI–TOF) *m*/*z*: [M+H]^+^ calculated for C_20_H_30_N_3_O_5_S^+^ 424.1901 found 424.1873.

*Dimethyl 4-oxo-2-propyl-8-((2,4,4-trimethylpentan-2-yl)amino)-4,6-dihydropyrimido [2,1-b][1,3]thiazine-6,7-dicarboxylate* (**10o**); 2-amino-6-propyl-4H-1,3-thiazin-4-one (**9d**) (0.25 g, 1.47 mmol), 1,1,3,3-tetramethylbutyl isocyanide (**1b**) (0.21 g, 1.47 mmol) and dimethyl acetylenedicarboxylate (**2a**) (0.21 g, 1.47 mmol). Physical characteristics: yellow paste; Yield: 0.52 g, 79%, R_f_: 0.5, hexane/ethyl acetate (90%:10%); ^1^H NMR (400 MHz, CDCl_3_) δ 8.64 (1H, br s, NH), 6.24 (2H, 2 × s, Ar-CH, H-6), 3.65 (3H, s, OCH_3_), 3.59 (3H, s, OCH_3_), 2.38 (2H, t, *J* = 7.6 Hz, Ar-CH_2_), 1.73 (2H, s, C-CH_2_), 1.64 (2H, sextet, *J* = 7.2 Hz, CH_3_CH_2_), 1.38 and 1.36 (6H, 2 × s, 2 × CH_3_), 0.94 (3H, *J* = 7.2 Hz, CH_3_), 0.87 (9H, s, C(CH_3_)_3_); ^13^C NMR (101 MHz, CDCl_3_) δ 171.1 (C=O), 168.4 (C=O), 161.3 (C=O), 157.8 (C-10), 155.4 (C-8), 154.4 (C-2), 115.1 (C-3), 71.9 (C-7), 56.2 (C(CH_3_)_3_), 53.3 (Ar-CH_2_), 52.8 (OCH_3_), 51.1 (C-6), 50.7 (OCH_3_), 37.9 (C-CH_2_), 31.8 (C(CH_3_)_3_), 31.7, 31.6 (2 × CH_3_) 21.6 (CH_3_CH_2_), 13.4 (CH_2_CH_3_); FTIR ν_max_/cm^−1^: 3279, 2951, 2873, 1739, 1686, 1648, 1598, 1598, 1532, 1442; HRMS (ESI–TOF) *m*/*z*: [M + H]^+^ calculated for C_22_H_34_N_3_O_5_S^+^ 452.2214 found 452.2190.

*Diethyl 4-oxo-2-propyl-8-((2,4,4-trimethylpentan-2-yl)amino)-4,6-dihydropyrimido[2,1-b][1,3]thiazine-6,7-dicarboxylate* (**10p**); 2-amino-6-propyl-4*H*-1,3-thiazin-4-one (**9d**) (0.25 g, 1.47 mmol), 1,1,3,3-tetramethylbutyl isocyanide (**1b**) (0.21 g, 1.47 mmol) and diethyl acetylenedicarboxylate (**2b**) (0.25 g, 1.60 mmol). Physical characteristics: yellow paste; Yield: 0.55 g, 79%, R_f_: 0.6, hexane/ethyl acetate (90%:10%); ^1^H NMR (400 MHz, CDCl_3_) δ 8.75 (1H, br s, NH), 6.30 (2H,2 × s, Ar-CH, H-6), 4.29–4.09 (4H, m, 2 × CH_2_CH_3_), 2.46 (2H, t, *J* = 7.2 Hz, Ar-CH_2_), 1.85–1.69 (2 × 2H, m, 2 x CH_2_), 1.46 and 1.44 (6H, 2 × s, (CH_3_)_2_-C), 1.33 and 1.21 (6H, 2 × s, 2 × CH_3_), 1.31 (3H, t, *J* = 6.8 Hz, CH_3_), 1.22 (3H, t, *J* = 7.2 Hz, CH_3_), 1.03 (3H, t, *J* = 7.2 Hz, CH_3_), 0.96 (9H, s, C(CH_3_)_3_); ^13^C NMR (101 MHz, CDCl_3_) δ 170.7 (C=O), 168.1(C=O), 161.5 (C=O), 157.6 (C-10), 155.3 (C-8), 154.3 (C-2), 115.1 (C-3), 72.1 (C-7), 61.7 (O-CH_2_), 59.1 (O-CH_2_), 56.1 (C(CH_3_)_3_), 53.2 (O-CH_2_), 51.3 (C-6), 37.9 (Ar-CH_2_), 31.6 (C(CH_3_)_3_), 21.6 (CH_3_CH_2_), 14.9 (CH_3_), 14.1 (CH_3_), 13.4 (CH_3_); FTIR ν_max_/cm^−1^: 3285, 2957, 2910, 2870, 1737, 1687, 1646, 1600, 1535, 1432; HRMS (ESI–TOF) *m*/*z*: [M + H]^+^ calculated for C_24_H_38_N_3_O_5_S^+^ 480.2527 found 480.2526.

*Dimethyl 8-(tert-butylamino)-4-oxo-2-phenyl-4,6-dihydropyrimido[2,1-b][1,3]thiazine-6,7-dicarboxylate* (**10q**); 2-amino-6-phenyl-4*H*-1,3-thiazin-4-one (**9e**) (0.25 g, 1.22 mmol), *tert*-butyl isocyanide (**1a**) (0.10 g, 1.22 mmol) and dimethyl acetylenedicarboxylate (**2a**) (0.17 g, 1.22 mmol). Physical characteristics: yellow solid; Yield: 0.40 g, 77%; Mp: 99–101 °C, R_f_: 0.58, hexane/ethyl acetate (90%:10%); ^1^H NMR (400 MHz, CDCl_3_) δ 8.74 (1H, br s, NH), 7.59–7.48 (5H, m, Ar-H) 6.69 (1H, s, Ar-CH), 6.36 (1H, s, H-6), 3.75 (3H, s, OCH_3_), 3.71 (3H, s, OCH_3_), 1.43 (9H, s, C(CH_3_)_3_); ^13^C NMR (101 MHz, CDCl_3_) δ 171.1 (C=O), 168.5 (C=O), 161.8 (C=O), 157.7 (C-10), 155.1 (C-8), 150.9 (C-2), 133.9 (Ar-C), 132.0 (Ar-C), 129.6 (Ar-CH), 126.6 (Ar-CH), 126.5 (Ar-CH), 113.9 (C-3), 72.1 (C-7), 53.0 (C-6), 52.7 (OCH_3_), 51.3 (OCH_3_), 50.9 (C(CH_3_)_3_), 30.9 (C(CH_3_)_3_); FTIR ν_max_/cm^−1^: 2977, 2841, 1735, 1683, 1582, 1448, 1370; HRMS (ESI–TOF) *m*/*z*: [M + H]+ calculated for C_21_H_24_N_3_O_5_S^+^ 430.1431 found 430.1445.

*Diethyl 8-(tert-butylamino)-4-oxo-2-phenyl-4,6-dihydropyrimido[2,1-b][1,3]thiazine-6,7-dicarboxylate* (**10r**); 2-amino-6-phenyl-4*H*-1,3-thiazin-4-one (**9e**) (0.25 g, 1.22 mmol), *tert*-butyl isocyanide (**1a**) (0.10 g, 1.22 mmol) diethyl acetylenedicarboxylate (**2b**) (0.21 g, 1.22 mmol). Physical characteristics: yellow solid; Yield: 0.45 g, 80%; Mp: 145–147 °C, R_f_: 0.5, hexane/ethyl acetate (90%:10%); ^1^H NMR (400 MHz, CDCl_3_) δ 8.76 (1H, br s, NH), 7.59-7.47 (5H, m, Ar-H), 6.68 (1H, s, Ar-CH), 6.34 (1H, s, H-6), 4.29–4.10 (4H, m, 2 × CH_2_), 1.43 (9H, s, C(CH_3_)_3_), 1.33–1.22 (6H, m, 2 × CH_3_); ^13^C NMR (101 MHz, CDCl_3_) δ 170.7 (C=O), 168.2 (C=O), 161.8 (C=O), 157.5 (C-10), 155.2 (C-8), 150.9 (C-2), 133.9 (Ar-C), 131.9 (Ar-C), 129.6 (Ar-C), 126.4 (ArCH), 113.9 (C-3), 72.4 (C-7), 61.9 (O-CH_2_), 59.2 (O-CH_2_), 52.6 (C-6),) 51.5 (C(CH_3_)_3_), 30.9 (C(CH_3_)_3_), 14.9 (CH_3_), 14.2 (CH_3_); FTIR ν_max_/cm^−1^: 3279, 2976, 2873, 1734, 1676, 1645, 1606, 1535, 1462; HRMS (ESI–TOF) m/z: [M+H]^+^ calculated for C_23_H_28_N_3_O_5_S^+^ 458.1744 found 458.1744.

*Dimethyl 4-oxo-2-phenyl-8-((2,4,4-trimethylpentan-2-yl)amino)-4,6-dihydropyrimido[2,1-b][1,3]thiazine-6,7-dicarboxylate* (**10s**); 2-amino-6-phenyl-4*H*-1,3-thiazin-4-one (**9e**) (0.25 g, 1.22 mmol), 1,1,3,3-tetramethylbutyl isocyanide (**1b**) (0.17 g, 1.22 mmol) and dimethyl acetylenedicarboxylate (**2b**) (3.(0.17 g, 1.22 mmol). Physical characteristics: yellow solid; Yield: 0.45 g, 76%; Mp: 162–164 °C, R_f_: 0.4, hexane/ethyl acetate (90%:10%); ^1^H NMR (400 MHz, CDCl_3_) δ 8.77 (1H, br s, NH), 7.62–7.49 (5H, m, Ar-H), 6.71 (1H, s, Ar-CH), 6.39 (1H, s, H-6), 3.77 (3H, s, OCH_3_), 3.71 (3H, s, OCH_3_) 1.85 (CH_2_), 1.50 and 1.48 (6H, 2 × s, 2 × CH_3_), 0.98 (9H, s, C(CH_3_)_3_); ^13^C NMR (101 MHz, CDCl_3_) δ 171.0 (C=O), 168.4 (C=O), 161.6 (C=O), 157.4 (C-10), 155.4 (C-8), 150.9 (C-2), 133.8(Ar-C), 131.9 (Ar-CH), 129.5 (Ar-CH), 126.4 (Ar-CH), 113.9 (C-3), 71.2 (C-7), 56.2 (C(CH_3_)_3_), 53.3 (CH_2_), 52.9 (C-6), 51.3 and 50.7 (2 × OCH_3_), 31.8 (C(CH_3_)_2_), 31.7 (C(CH_3_)_3_), 31.6 (CH_3_O), 31.5 (CH_3_O); FTIR ν_max_/cm^−1^: 2953, 2878, 1732, 1681, 1651, 1613, 1542, 1450;^–^HRMS (ESI–TOF) *m*/*z*: [M + H]^+^ calculated for C_25_H_32_N_3_O_5_S^+^ 486.2057 found 486.2044.

*Diethyl 4-oxo-2-phenyl-8-((2,4,4-trimethylpentan-2-yl)amino)-4,6-dihydropyrimido[2,1-b][1,3]thiazine-6,7-dicarboxylate* (**10t**); 2-amino-6-phenyl-4*H*-1,3-thiazin-4-one (**9e**) (0.25 g, 1.22 mmol), 1,1,3,3-tetramethylbutyl isocyanide (**1b**) (0.17 g, 1.22 mmol) and dimethyl acetylenedicarboxylate (**2b**) (0.21 g, 1.22 mmol). Physical characteristics: yellow solid; Yield: 0.52 g, 83%; Mp: 88–90 °C, R_f_: 0.5, hexane-ethyl acetate (90%:10%); ^1^H NMR (400 MHz, CDCl_3_) δ 8.00 (1H, br s, NH), 7.63–7.50 (5H, m, Ar-H), 6.72 (1H, s, Ar-CH), 6.37 (1H, s, H-6), 4.34–4.11 (4H, m, 2 × O-CH_2_), 1.87 (2H, q, *J* = 14.8 Hz, CH_2_), 1.50 and 1.48 (6H, 2 × s, 2 × CH_3_), 1.34 (3H, t, *J* = 7.2 Hz, CH_2_CH_3_), 1.26 (3H, t, *J* = 6.8 Hz, CH_2_CH_3_), 0.98 (9H, s, C(CH_3_)_3_); ^13^C NMR (101 MHz, CDCl_3_) δ 170.7 (C=O), 168.2 (C=O), 161.8 (C=O), 157.31 (C-10), 155.3 (C-8), 150.9 (C-2), 133.9 (Ar-C), 131.9 (Ar-CH), 129.6 (Ar-CH), 126.4 (Ar-CH), 113.9 (C-3), 72.2 (C-7), 61.8 (O-CH_2_), 59.2 (O-CH_2_), 56.2 (C(CH_3_)_3_), 53.3 (O-CH_2_), 51.5 (C-6), 31.8 (C(CH_3_)_3_), 31.8 (CH_3_), 31.7 (CH_3_), 14.9 (CH_3_), 14.2 (CH_3_); FTIR ν_max_/cm^−1^: 2963, 2925, 2864, 1735, 1671, 1641, 1611, 1535, 1441; HRMS (ESI–TOF) *m*/*z*: [M + H]^+^ Calculated for C_27_H_36_N_3_O_5_S^+^ 514.2370 found 514.2350.

*Dimethyl 8-(cyclohexylamino)-4-oxo-2-phenyl-4,6-dihydropyrimido[2,1-b][1,3]thiazine-6,7-dicarboxylate* (**10u**); 2-amino-6-phenyl-*4H*-1,3-thiazin-4-one (**9e**) (0.25 g, 1.22 mmol), cyclohexyl isocyanide (**1c**) (0.13 g, 1.22 mmol) and dimethyl acetylenedicarboxylate (**2a**) (0.17 g, 1.22 mmol). Physical characteristics: yellow solid; Yield: 0.44 g, 79%; Mp: 168–170 °C, R_f_: 0.4, hexane/ethyl acetate (90%:10%); ^1^H NMR (400 MHz, CDCl_3_) δ 8.59 (1H, br s, NH), 7.63–7.51 (5H, m, Ar-H), 6.71 (1H, s, Ar-CH), 6.39 (1H, s, CH-6), 3.99 (1H, s, N-CH), 3.78 (3H, s, OCH_3_), 3.73 (3H, s, OCH_3_), 1.99–1.59 (6H, m, cyclohexyl), 1.45–1.24 (4H, m, cyclohexyl); ^13^C NMR (101 MHz, CDCl_3_) δ 171.1 (C=O), 168.4 (C=O), 161.7 (C=O), 159.0 (C-10), 154.2 (C-8), 150.9 (C-2), 133.9 (Ar-C), 132.0 (Ar-CH), 129.6 (Ar-CH), 126.4 (Ar-CH), 113.8 (C-3), 71.4 (C-7), 52.9 (O-CH_3_), 51.5 (C-6), 50.8 (O-CH_3_), 49.7 (CHNH), 34.3 (CH_2_, cyclohexyl), 33.7 (CH_2_, cyclohexyl), 25.7 (CH_2_, cyclohexyl), 24.8 (CH_2_, cyclohexyl), 24.7 (CH_2_, cyclohexyl); FTIR ν_max_/cm^−1^: 3282, 2918, 2846, 1740, 1677, 1651, 1604, 1533, 1444; HRMS (ESI–TOF) *m*/*z*: [M + H]^+^ calculated for C_23_H_26_N_3_O_5_S^+^ 456.1588 found 456.1592.

#### 3.1.3. Synthesis of Methyl 7-Oxo-7*H*-Thiazolo[3,2-*a*]Pyrimidine-5-Carboxylate Derivatives (**13a–j**). Method B (3-CR)

In a round-bottomed flask equipped with a magnetic stirrer bar, thiourea (**15**) was added to a solution of α-haloketone (**16**) in absolute ethanol (15 mL) and tetrahydrofuran (THF) (15 mL), and the reaction mixture was allowed to stir at room temperature for at least 15 min. After this time, dimethyl acetylenedicarboxylate (DMAD) or diethyl acetylenedicarboxylate (DEtAD) (**2**) was slowly added over at least 10 min, and the reaction mixture was heated at 80 °C for 6–12 h while being monitored by TLC. After completion, the reaction was cooled, and the solvents were removed under reduced pressure to obtain a residue which was washed with cold ethanol and filtered to give a solid product.

*Methyl 2,3-dimethyl-7-oxo-7H-thiazolo[3,2-a]pyrimidine-5-carboxylate* (**13a**). Thiourea (**15**) (0.36 g, 4.6 mmol), 3-chloro-2-butanone (**16c**) (0.50 g, 4.6 mmol) and DMAD (**2a**) (0.65 g, 4.6 mmol). Physical properties brown solid; Yield: 0.89 g, 82%; Mp: 165–167 °C; **R_f_** 0.5, hexane/ethyl acetate (40%:60 %); ^1^H NMR (400 MHz, DMSO-d6), 6.50 (1H, s, H-6), 3.95 (3H, s, O-CH_3_), 2.28 (3H, s, CH_3_), 2.10 (3H, s, CH_3_); ^13^C NMR (101 MHz, DMSO-d6) δ 164.9 (C=O), 164.7 (C=O), 161.5 (C-9), 138.9 (C-5), 127.9 (C-2), 116.7 (C-3), 112.1 (C-6), 54.0 (O-CH_3_), 12.6 (CH_3_), 11.8 (CH_3_); FTIR νmax/cm^−1^: 2993, 1734, 1638, 1608, 1491, 1413; HRMS (ESI–TOF) *m*/*z*: [M + H]^+^ calculated for C_10_H_11_N_2_O_3_S^+^ 239.0485, found 239.0486.

*Methyl 7-oxo-3-(p-tolyl)-7H-thiazolo[3,2-a]pyrimidine-5-carboxylate* (**13b**). Thiourea (**15**) (0.19 g, 2.4 mmol), 2-bromo-4-methylacetophenone (**16d**) (0.52 g, 2.4 mmol) and DMAD (**2a**) (0.34 g, 2.4 mmol). Physical properties: orange solid; Yield: 0.51 g, 71%; Mp: 176–178 °C; R_f_ 0.4, hexane/ethyl acetate (40%:60%); ^1^H NMR (400 MHz, DMSO-*d_6_*) 7.35–7.33 (2H, m, ArH), 7.30–7.27 (3H, m, ArH), 6.47 (1H, s, H-6), 3.15 (3H, s, O-CH_3_), 2.35 (3H, s, CH_3_); ^13^C NMR (101 MHz, DMSO-d6) δ 166.8 (C=O), 165.3 (C=O), 160.6 (C-9), 139.4 (C-5), 138.9 (Ar-C), 136.6 (Ar-C), 129.4 (Ar-CH), 127.5 (Ar-C), 127.2 (ArCH), 113.01 (C-6), 108.14 (C-2), 52.8 (O-CH_3_); FTIR νmax/cm^−1^: 3041, 1742, 1639, 1587, 1509, 1485, 1416; HRMS (ESI–TOF) *m*/*z*: [M + H]^+^ calculated for C_15_H_13_N_2_O_3_S^+^ 301.0641, found 301.0648.

*Methyl 3-(4-fluorophenyl)-7-oxo-7H-thiazolo[3,2-a]pyrimidine-5-carboxylate* (**13c**). Thiourea (**15**) (0.19 g, 2.3 mmol), 2-bromo-4-fluoroacetophenone (**16e**) (0.51 g, 2.3 mmol) and DMAD (**2a**) (0.33 g, 2.3 mmol). Physical properties: yellow solid; Yield: 0.49 g, 70%; Mp: 220–222 °C; R_f_ 0.5, hexane/ethyl acetate (40%:60%); ^1^H NMR (400 MHz, DMSO-*d6*) 7.56–7.53 (2H, m, ArH), 7.37–7.34 (3H, m, ArH), 6.49 (1H, s, H-6), 3.23 (3H, s, O-CH_3_); **^13^C NMR** (101 MHz, DMSO-*d6*) δ 166.7 (C=O), 165.3 (C=O), 162.6 (d, *J_CF_* = 246 Hz, Ar-C), 161.3 (ArC), 160.6 (ArC), 138.7 (Ar-C),135.4 (ArC), 129.9 (d, *J_CF_* = 8.1 Hz, Ar-CH), 126.9 (d, *J_CF_* = 3.0 Hz, Ar-C), 115.9 (d, *J_CF_* = 22.1 Hz, Ar-CH), 113.1 (C-6), 109.0 (C-2), 52.9 (O-CH_3_); FTIR νmax/cm^−1^: 3058, 1742, 1640, 1581, 1489; HRMS (ESI–TOF) *m*/*z*: [M + H]^+^ calculated for C_14_H_10_FN_2_O_3_S^+^ 305.0391, found 305.0391.

*Methyl 3-(4-chlorophenyl)-7-oxo-7H-thiazolo[3,2-a]pyrimidine-5-carboxylate* (**13d**). Thiourea (**15**) (0.16 g, 2.1 mmol), 2-bromo-4-chloroacetophenone (**16f**) (0.50 g, 2.1 mmol) and DMAD (**2a**) (0.30 g, 2.1 mmol). Physical properties: yellow solid; Yield: 0.48 g, 71%; Mp: 200–202 °C; R_f_ 0.8, hexane/ethyl acetate (40%:60%); ^1^H NMR (400 MHz, DMSO-*d6*) 7.58 (2H, d, *J* = 8.4 Hz, ArH), 7.50 (2H, d, *J* = 8.4 Hz, ArH), 7.39 (1H, s, H-2), 6.50 (1H, s, H-6), 3.23 (3H, s, O-CH_3_); ^13^C NMR (101 MHz, DMSO-*d6*) δ 166.8 (C=O), 165.3 (C=O), 160.6 (C-9), 138.6 (Ar-C), 135.2 (Ar-C), 134.3 (Ar-C), 129.3 (Ar-C), 129.1 (Ar-CH), 129.0 (Ar-CH), 128.7 (Ar-CH), 127.4 (Ar-CH), 113.2 (C-6), 109.5 (C-2), 52.90 (O-CH_3_); FTIR νmax/cm^−1^: 3049, 1734, 1633, 1583, 1494, 1481; HRMS (ESI–TOF) m/z: [M+H]^+^ calculated for C_14_H_10_ClN_2_O_3_S^+^ 321.0095, found 321.0087.

*Ethyl 7-oxo-7H-thiazolo[3,2-a]pyrimidine-5-carboxylate* (**13e**). Thiourea (**15**) (0.31 g, 4.1 mmol), chloroacetaldehyde (**16a**) (0.50 g, 4.1 mmol) and DEtAD (**2b**) (0.69 g, 4.1 mmol); Physical properties: yellow solid; Yield: 0.84 g, 91%; Mp: 180–182 °C; R_f_ 0.4, hexane/ethyl acetate (40%:60%); ^1^H NMR (400 MHz, DMSO-*d6*) 8.30 (1H, d, *J* = 4.8 Hz, H-2), 7.36 (1H, d, *J* = 4.8 Hz, H-3), 6.76 (1H, s, H-6), 4.38 (2H, q, *J* = 6.8 Hz, O-CH_2_), 1.34 (3H, t, *J* = 7.2 Hz, CH_3_); ^13^C NMR (101 MHz, DMSO-*d6*) δ 166.8 (C=O), 166.0 (C=O), 160.3 (C-9), 136.6 (C-5), 124.6 (C-2), 114.3 (C-6), 110.0 (C-3), 62.9 (O-CH_2_), 13.7 (CH_3_); FTIR νmax/cm^−1^: 3176, 3077, 2978, 1720, 1621, 1557, 1471; HRMS (ESI–TOF) *m*/*z*: [M + H]^+^ calculated for C_9_H_9_N_2_O_3_S^+^ 225.0328, found 225.0339.

*Ethyl 3-methyl-7-oxo-7H-thiazolo[3,2-a]pyrimidine-5-carboxylate* (**13f**). Thiourea (**15**) (0.41 g, 5.4 mmol), chloroacetone (**16b**) (0.5 g, 5.4 mmol) and DEtAD (**2b**) (0.92 g, 5.4 mmol); Physical properties: brown solid; Yield: 1.1 g, 86%; Mp: 150–152 °C; R_f_ 0.5, hexane/ethyl acetate (40%:60%); ^1^H NMR (400 MHz, DMSO-*d6*) 7.10 (1H, s, H-2), 6.53 (1H, s, H-6), 4.38 (2H, q, *J* = 6.8 Hz, O-CH_2_), 2.23 (3H, s, CH_3_) 1.34 (3H, t, *J* = 6.8 Hz, CH_3_); ^13^C NMR (101 MHz, DMSO-*d6*) δ 166.8 (C=O), 165.0 (C=O), 160.9 (C-9), 139.2 (Ar-C), 132.9 (Ar-C), 111.9 (C-6), 107.1 (C-2), 63.5 (CH_2_), 15.3 (CH_3_) 13.6 (CH_3_); FTIR νmax/cm^−1^: 2973, 1731, 1634, 1493; HRMS (ESI–TOF) *m*/*z*: [M + H]^+^ calculated for C_10_H_11_N_2_O_3_S^+^ 239.0485, found 239.0497.

*Ethyl 2,3-dimethyl-7-oxo-7H-thiazolo[3,2-a]pyrimidine-5-carboxylate* (**13g**). Thiourea (**15**) (0.36 g, 4.6 mmol), 3-chloro-2-butanone (**16c**) (0.50 g, 4.6 mmol) and DEtAD (**2b**) (0.78 g, 4.6 mmol). Physical properties: brown solid; Yield: 0.94 g, 81%; Mp: 153–155 °C; R_f_ 0.5, hexane/ethyl acetate (40%:60%); ^1^H NMR (400 MHz, DMSO-*d6*), 6.5 (1H, s, H-6), 4.40 (2H, q, *J* = 5.2 Hz, CH_2_), 2.28 (3H, s, CH_3_), 2.12 (3H, s, CH_3_), 1.33 (3H, t, *J* = 7.6 Hz, CH_2_CH_3_); ^13^C NMR (101 MHz, DMSO-*d6*) δ 165.0 (C=O), 164.7 (C=O), 161.0 (C-9), 139.2 (Ar-C), 127.9 (Ar-C), 116.7 (Ar-C), 112.1 (C-6), 63.5 (O-CH_2_), 13.6, 12.7, (2 × CH_3_), 11.8 (CH_2_CH_3_); FTIR νmax/cm^−1^: 2988, 1732, 1641, 1489, 1452; HRMS (ESI–TOF) *m*/*z*: [M + H]^+^ calculated for C_11_H_13_N_2_O_3_S^+^ 253.0641, found 253.0652.

*Ethyl 3-(4-fluorophenyl)-7-oxo-7H-thiazolo[3,2-a]pyrimidine-5-carboxylate* (**13h**). Thiourea (**15**) (0.19 g, 2.3 mmol), 2-bromo-4-fluoroacetophenone (**16e**) (0.51 g, 2.3 mmol) and DEtAD (**2b**) (0.39 g, 2.3 mmol). Physical properties: yellow solid, Yield 0.63 g, 86%; **Mp:** 180–182 °C; R_f_ 0.5, hexane/ethyl acetate (40%:60%); ^1^H NMR (400 MHz, DMSO-*d6*) 7.58–7.52 (2H, m, ArH), 7.37–7.27 (3H, m, ArH), 6.49 (1H, s, H-6), 3.61 (2H, q, *J* = 7.2 Hz, O-CH_2_) 1.04 (3H, t, *J* = 7.2 Hz, CH_3_); ^13^C NMR (101 MHz, DMSO-*d6*) δ 166.8 (C=O), 165.4 (C=O), 162.6 (d, *J_CF_* = 246 Hz, Ar-C), 161.4 (Ar-C), 160.2 (Ar-C), 138.9 (Ar-C), 135.4 (Ar-C), 129.9 (d, *J_CF_* = 9.1 Hz, Ar-CH), 128.0 (d, *J_CF_* = 8.1 Hz, Ar-CH), 126.9 (d, *J_CF_* = 4.0 Hz, Ar-CH), 115.9 (d, *J_CF_* = 22.2 Hz, Ar-CH) 113.1 (C-6), 109.1 C-2), 62.7 (CH_2_-O), 13.3 (O-CH_3_); FTIR νmax/cm^−1^: 3061, 1733, 1639, 1489; HRMS (ESI–TOF) *m*/*z*: [M + H]^+^ calculated for C_15_H_12_FN_2_O_3_S^+^ 319.0547, found 319.0565.

*Ethyl 3-(4-chlorophenyl)-7-oxo-7H-thiazolo[3,2-a]pyrimidine-5-carboxylate* (**13i**). Thiourea (**15**) (0.16 g, 2.1 mmol), 2-bromo-4-chloroacetophenone (**16f**) (0.50 g, 2.1 mmol) and DEtAD (**2b**) (0.36 g, 2.1 mmol). Physical properties: yellow solid, Yield: 0.59 g, 84%; Mp: 198–200 °C; R_f_ 0.9, hexane/ethyl acetate (40%:60%); ^1^H NMR (400 MHz, DMSO-*d6*) 7.58 (2H, d, *J* = 8.4 Hz, Ar-H), 7.51 (2H, d, *J* = 8.4 Hz, Ar-H), 7.39 (1H, s, H-2), 6.51 (1H, s, H-6), 3.63 (2H, q, *J* = 7.2 Hz, O-CH_2_), 1.03 (3H, t, *J* = 6.8, Hz, CH_3_); ^13^C NMR (101 MHz, DMSO-*d6*) δ 166.8 (C=O), 165.3 (C=O), 160.1 (C-9), 138.7 (Ar-C), 135.3 (Ar-CH), 134.3 (Ar-C), 129.4 (Ar-C), 129.1 (Ar-CH), 128.9 (Ar-CH), 113.2 (C-6), 109.38 (C-2), 62.7 (CH_2_-O), 13.2 (CH_3_); FTIR νmax/cm^−1^: 3057, 2986, 1733, 1640, 1580, 1501, 1482; HRMS (ESI–TOF) *m*/*z*: [M + H]^+^ calculated for C_15_H_12_ClN_2_O_3_S^+^ 335.0252, found 335.0267.

#### 3.1.4. Synthesis of Methyl 7-Oxo-7*H*-Thiazolo[3,2-*a*]Pyrimidine-5-Carboxylate Derivatives (**13j**, **19a–b**, **20** and **5**) Method A (2-CR)

In a round-bottomed flask equipped with a magnetic stirrer bar, DMAD/DEtAD (**2**) was added to the mixture of 2-aminothiazole derivative (**11a–h**) or 2-imino-1,3-oxazolidine (**17**) or 1*H*-1,2,4-triazol-5-amine (**18**) or 1*H*-1,2,4-triazole-5-thiol (**5**) in ethanol. The reaction mixture was allowed to reflux for 12–15 h while being monitored by TLC. After completion, the reaction was cooled and the solvent was removed under reduced pressure to obtain a residue which was washed with cold ethanol and filtered to give solid product.

*N-(tert-butyl)-7-oxo-5H-thiazolo[3,2-a]pyrimidine-5-carboxylate* (**13j**). Thiazol-2-amine (**11a**) (0.20 g, 1.99 mmol), di-*tert*-butyl acetylenedicarboxylate (DTAD) (**2c**) (0.45 g, 1.99 mmol); Physical properties: yellow solid; Yield: 0.36 g, 72%; **Mp**:246–248 °C; R_f_ 0.5, hexane/ethyl acetate (40%:60%); ^1^H NMR (400 MHz, DMSO-*d6*) 8.29 (1H, d, *J* = 4.4 Hz, H-2), 7.34 (1H, d, *J* = 5.2 Hz, H-3), 6.73 (1H, s, H-6), 1.57 (9H, s, ((CH_3_)_3_); ^13^C NMR (101 MHz, DMSO-d6) δ 166.8 (C=O), 166.2 (C=O), 159.3 (C-9), 137.4 (C-5), 124.5 (C-2), 114.2 (C-6), 109.9 (C-3), 85.0 (C(CH_3_)_3_), 27.4 ((CH_3_)_3_); FTIR νmax/cm^−1^: 2923, 1729, 1639; HRMS (ESI–TOF) *m*/*z*: [M + H]^+^ calculated for C_11_H_13_N_2_O_3_S^+^ 253.0641, found 253.0642.

*Methyl 5-oxo-3,5-dihydro-2H-oxazolo[3,2-a]pyrimidine-7-carboxylate* (**19a**); 2-imino-1,3-oxazolidine (0.51 g, 5.8 mmol) and DMAD (**2a**) (0.83 g, 5.8 mmol); Physical properties: White solid; Yield: 0.95 g, 82%; Mp: 251–253 °C; R_f_ 0.5, ethyl acetate (100 %); ^1^H NMR (400 MHz, DMSO-*d6*) 6.37 (1H, s, H-6) 4.67 (2H, t, *J* = 8.4 Hz, CH_2_), 4.49 (2H, t, *J* = 8.4 Hz, CH_2_), 3.87 (3H, s, O-CH_3_); ^13^C NMR (101 MHz, DMSO-d6) δ 170.9, (C=O) 162.1 (C=O), 160.8 (C-9), 138.1 (C-7), 110.9 (C-6), 66.6 (CH_2_), 53.5 (O-CH_3_), 47.03 (CH_2_); FTIR νmax/cm^−1^: 2965, 1732, 1624, 1535, 1476, 1421; HRMS (ESI–TOF) *m*/*z*: [M + H]^+^ calculated for C_8_H_9_N_2_O_4_^+^ 197.0557, found 197.0549.

*Ethyl 5-oxo-3,5-dihydro-2H-oxazolo[3,2-a]pyrimidine-7-carboxylate* (**19b**); 2-imino-1,3-oxazolidine (0.51 g, 5.8 mmol) and DEtAD (**2b**) (0.98 g, 5.8 mmol). Physical properties: White solid: Yield 0.98 g, 80%; Mp: 297–299 °C; R_f_ 0.5, ethyl acetate (100%); ^1^H NMR (400 MHz, DMSO-*d6*) 5.64 (1H, s, H-6), 4.27 (2H, q, *J* = 7.2 Hz, O-CH_2_), 4.04 (2H, t, *J* = 6.0 Hz, CH_2_), 3.62 (2H, t, *J* = 5.6 Hz, CH_2_), 1.27 (3H, t, *J* = 7.2 Hz, CH_3_); ^13^C NMR (101 MHz, DMSO-*d6*) δ 170.4 (C=O) 163.2 (C=O), 161.6 (C-9), 155.7 (C-7), 89.5 (C-6), 61.1 (CH_2_), 58.5 (CH_2_), 48.1 (CH_2_), 13.8 (CH_3_); FTIR νmax/cm^−1^: 3125, 2958, 1700, 1698, 1662, 1642, 1637, 1381; HRMS (ESI–TOF) *m*/*z*: [M + H]^+^ calculated for C_9_H_11_N_2_O_4_^+^ 211.0713, found 211.0708.

*Ethyl 5-oxo-5,8-dihydro-[1,2,4]triazolo[4,3-a]pyrimidine-7-carboxylate* (**20**); 1*H*-1,2,4-triazol-5-amine (0.50 g, 6.0 mmol) and DEtAD (**2b**) (1.02 g, 6.0 mmol). Physical properties: Brown solid: Yield: 1.1 g, 88%; Mp: 185–187 °C; R_f_ 0.5, ethyl acetate (100 %); ^1^H NMR (400 MHz, DMSO-*d6*) 8.91 (1H, s, H-5), 6.66 (1H, s, H-7), 4.50 (2H, q, *J* = 6.8 Hz, O-CH_2_), 1.40 (3H, t, *J* = 7.2 Hz, CH_3_); ^13^C NMR (101 MHz, DMSO-d6) δ 160.5 (C=O), 158.9 (C=O), 151.9 (C-5), 134.1 (Ar-C), 132.9 (Ar-C) 112.9 (C-7), 62.5 (CH_2_), 12.9 (CH_3_); FTIR νmax/cm^−1^: 3150, 3068, 2909, 2731, 1722, 1680, 1610, 1579; HRMS (ESI–TOF) *m*/*z*: [M + H]^+^ calculated for C_8_H_9_N_4_O_3_^+^ 209.0669, found 209.0677.

*7-Oxo-7H-[1,2,4]triazolo[5,1-b][1,3]thiazine-5-carboxylate* (**5**); 1*H*-1,2,4-Triazole-5-thiol (0.50 g, 5.0 mmol) and DEtAD (**2b**) (0.85 g, 5.0 mmol). Physical properties: White solid; Yield: 0.92 g, 82%; Mp: 110–112 °C; R_f_ 0.9 hexane/ethyl acetate (20%:80%); ^1^H NMR (400 MHz, DMSO-*d6*) 8.68 (1H, s, H-4), 7.54 (1H, s, H-6), 4.48 (2H, q, *J* = 7.2 Hz, O-CH_2_), 1.38 (3H, t, *J* = 6.8 Hz, CH_3_); ^13^C NMR (101 MHz, DMSO-d6) δ 160.9 (C=O), 155.3 (C=O), 153.7 (C-4), 151.56 (C-2), 139.6 (C-7) 122.2 (C-6), 64.1 (CH_2_), 13.8 (CH_3_); FTIR νmax/cm^−1^: 3123, 3053, 2987, 1698, 1582, 1491; HRMS (ESI–TOF) *m*/*z*: [M + H]^+^ calculated for C_8_H_8_N_3_O_3_S^+^ 226.0281, found 226.0288.

### 3.2. X-ray Crystallography

Intensity data for **8**, **13a**, **13b** and **13d** were collected on a Bruker Apex-II CCD area detector (Bruker AXS, Karlsruhe, Germany) diffractometer with graphite monochromated Mo Kα radiation (50 kV, 30 mA). The collection method involved *ω*- and *φ*-scans of width 0.5° and 1024 × 1024 bit data frames. Using *Olex2* [49], the crystal structures were solved by with the *ShelXT* [50] structure solution program, using Intrinsic Phasing, and refined with the *ShelXL* [51] refinement package, using least-squares minimization. Non-hydrogen atoms were first refined isotropically, followed by anisotropic refinement by full matrix least-squares calculations based on *F*^2^.

**Crystal Datafor 8:** C_8_H_7_N_3_O_3_S (*M* =225.23 g/mol): triclinic, space group *P*-1 (no. 2), *a* = 8.6289(5) Å, *b* = 9.9003(6) Å, *c* = 11.9343(7) Å, *α* = 76.618(2)°, *β* = 72.127(2)°, *γ* = 89.767(2)°, *V* = 941.51(10) Å^3^, *Z* = 4, *T* = 173.15 K, μ(MoKα) = 0.333 mm^−1^, *Dcalc* = 1.589 g/cm^3^, 16,340 reflections measured (3.696° ≤ 2Θ ≤ 56.728°), 4715 unique (*R*_int_ = 0.0461, R_sigma_ = 0.0464) which were used in all calculations. The final *R*_1_ was 0.0333 (I > 2σ(I)) and *wR*_2_ was 0.0771 (all data). CCDC 2103713.

**Crystal Datafor 13a:** C_10_H_12_N_2_O_4_S (*M* =256.28 g/mol): monoclinic, space group *P*2_1_/*c* (no. 14), *a* = 7.4587(3) Å, *b* = 20.1251(9) Å, *c* = 8.0128(3) Å, *β* = 108.8923(15)°, *V* = 1137.98(8) Å^3^, *Z* = 4, *T* = 173.15 K, μ(MoKα) = 0.290 mm^−1^, *Dcalc* = 1.496 g/cm^3^, 11779 reflections measured (4.048° ≤ 2Θ ≤ 56.708°), 2839 unique (*R*_int_ = 0.0356, R_sigma_ = 0.0325) which were used in all calculations. The final *R*_1_ was 0.0359 (I > 2σ(I)) and *wR*_2_ was 0.0973 (all data). CCDC 2103714.

**Crystal Datafor 13b:** C_15_H_12_N_2_O_3_S (*M* =300.33 g/mol): monoclinic, space group *C*2/*c* (no. 15), *a* = 21.5767(7) Å, *b* = 8.7603(3) Å, *c* = 14.6735(5) Å, *β* = 93.5869(11)°, *V* = 2768.13(16) Å^3^, *Z* = 8, *T* = 173.15 K, μ(MoKα) = 0.245 mm^−1^, *Dcalc* = 1.441 g/cm^3^, 15439 reflections measured (3.782° ≤ 2Θ ≤ 56.796°), 3474 unique (*R*_int_ = 0.0236, R_sigma_ = 0.0204) which were used in all calculations. The final *R*_1_ was 0.0345 (I > 2σ(I)) and *wR*_2_ was 0.0887 (all data). CCDC 2103715.

**Crystal Datafor 13d:** C_14_H_9_ClN_2_O_3_S (*M* =320.74 g/mol): orthorhombic, space group *Pbca* (no. 61), *a* = 15.167(2) Å, *b* = 8.9457(14) Å, *c* = 19.944(3) Å, *V* = 2706.0(7) Å^3^, *Z* = 8, *T* = 173.15 K, μ(MoKα) = 0.447 mm^−1^, *Dcalc* = 1.575 g/cm^3^, 16592 reflections measured (4.084° ≤ 2Θ ≤ 50°), 2381 unique (*R*_int_ = 0.1235, R_sigma_ = 0.1070) which were used in all calculations. The final *R*_1_ was 0.0362 (I > 2σ(I)) and *wR*_2_ was 0.0696 (all data). CCDC 2103716.

CCDC 2103713-2103716 contains the supplementary crystallographic data for this paper. These data can be obtained free of charge via http://www.ccdc.cam.ac.uk/conts/retrieving.html (accessed 20 August 2021) (or from the CCDC, 12 Union Road, Cambridge CB2 1EZ, UK; Fax: +44 1223 336033; E-mail: deposit@ccdc.cam.ac.uk).

## 4. Conclusions

The synthesis of novel thiazine derivatives was accomplished by using a one-pot, three-component reaction. The facile and convenient reaction of isocyanides and DMAD or DEtAD under inert conditions at 0 °C formed a zwitterion intermediate that further reacted with compounds **9a–e**. On the other hand, the synthesis of 5*H*-thiazolo [3,2-*a*]pyrimidine-7-carboxylates was successfully accomplished by using two methods, either by two-component or three-component reaction. The one-pot, three-component reaction using thiourea, α-haloketone and dialkyl acetylenedicarboxylate was found to be more effective, achieving improved yields. The 2-aminothiazole derivatives were found to be less reactive towards DMAD and DEtAD as compared to 2-imino-1,3-oxazolidine (**17**), 1*H*-1,2,4-triazol-5-amine (**18**) or 1*H*-1,2,4-triazole-5-thiol (**5**) when using the two-component approach.

## Figures and Tables

**Table 1:**
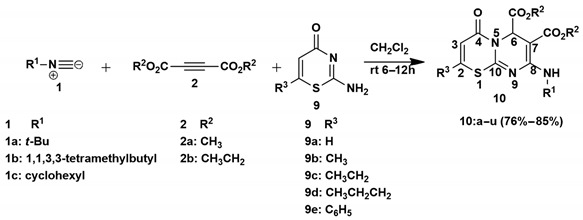
Isolated yields of products **10a–u** from the reaction of isocyanides **1**, dialkyl acetylenedicarboxylates **2** and thiazines **9** at room temperature.

Compound	R^1^	R^2^	R^3^	Yield % (Time)
**10a**	*t*-Bu	CH_3_	H	81 (6 h)
**10b**	*t*-Bu	CH_3_	H	85 (6 h)
**10c**	*t*-Bu	CH_3_	CH_3_	82 (6 h)
**10d**	*t*-Bu	CH_3_CH_2_	CH_3_	83 (6 h)
**10e**	1,1,3,3-tetramethylbutyl	CH_3_	CH_3_	76 (6 h)
**10f**	1,1,3,3-tetramethylbutyl	CH_3_CH_2_	CH_3_	77 (6 h)
**10g**	cyclohexyl	CH_3_	CH_3_	84 (12 h)
**10h**	cyclohexyl	CH_3_CH_2_	CH_3_	85 (12 h)
**10i**	*t*-Bu	CH_3_	CH_3_CH_2_	84 (6 h)
**10j**	*t*-Bu	CH_3_CH_2_	CH_3_CH_2_	79 (6 h)
**10k**	1,1,3,3-tetramethylbutyl	CH_3_	CH_3_CH_2_	81 (6 h)
**10l**	1,1,3,3-tetramethylbutyl	CH_3_CH_2_	CH_3_CH_2_	81 (6 h)
**10m**	*t*-Bu	CH_3_	CH_3_CH_2_CH_2_	84 (6 h)
**10n**	*t*-Bu	CH_3_CH_2_	CH_3_CH_2_CH_2_	85 (6 h)
**10o**	1,1,3,3-tetramethylbutyl	CH_3_	CH_3_CH_2_CH_2_	79 (6 h)
**10p**	1,1,3,3-tetramethylbutyl	CH_3_CH_2_	CH_3_CH_2_CH_2_	79 (6 h)
**10q**	*t*-Bu	CH_3_	C_6_H_5_	77 (6 h)
**10r**	*t*-Bu	CH_3_CH_2_	C_6_H_5_	80 (6 h)
**10s**	1,1,3,3-tetramethylbutyl	CH_3_	C_6_H_5_	76 (6 h)
**10t**	1,1,3,3-tetramethylbutyl	CH_3_CH_2_	C_6_H_5_	83 (6 h)
**10u**	cyclohexyl	CH_3_	C_6_H_5_	79 (12 h)

**Table 2:**
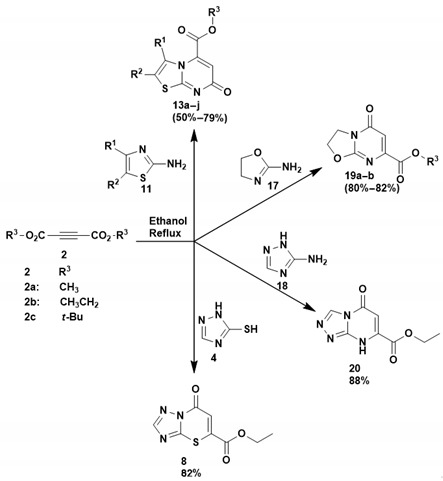
Isolated yields and reaction times for preparation of **8**, **13a–j, 19a–b** and **20**.

Compound	R^1^	R^2^	R^3^	Yield	Time (h)
**13a**	CH_3_	CH_3_	CH_3_	67	12
**13b**	Ph-CH_3_	H	CH_3_	58	6
**13c**	Ph-F	H	CH_3_	48	6
**13d**	Ph-Cl	H	CH_3_	50	6
**13e**	H	H	CH_3_CH_2_	69	12
**13f**	CH_3_	H	CH_3_CH_2_	71	12
**13g**	CH_3_	CH_3_	CH_3_CH_2_	79	12
**13h**	Ph-F	H	CH_3_CH_2_	51	6
**13i**	Ph-Cl	H	CH_3_CH_2_	52	6
**13j**	H	H	*t*-Bu	70	12
**19a**			CH_3_	82	12
**19b**			CH_3_CH_2_	80	12
**20**			CH_3_CH_2_	88	12
**8**			CH_3_CH_2_	82	12

**Table 3:**
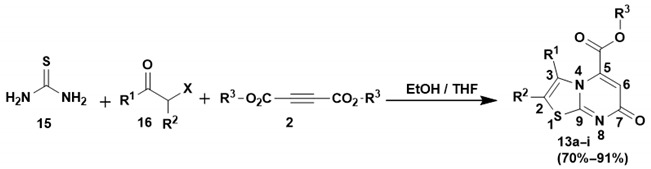
Isolated yields of **13a–i** from one-pot, three-component reaction.

Compound	X	R^1^	R^2^	R^3^	Yield (%)	Time (h)
**13a**	Cl	CH_3_	CH_3_	CH_3_	82	12
**13b**	Br	Ph-CH_3_	CH_3_	CH_3_	71	6
**13c**	Cl	Ph-F	CH_3_	CH_3_	70	6
**13d**	Br	Ph-Cl	H	CH_3_	71	12
**13e**	Cl	H	H	CH_3_CH_2_	83	12
**13f**	Cl	CH_3_	H	CH_3_CH_2_	91	12
**13g**	Cl	CH_3_	CH_3_	CH_3_CH_2_	86	12
**13h**	Cl	Ph-F	H	CH_3_CH_2_	86	6
**13i**	Br	Ph-Cl	H	CH_3_CH_2_	84	6

## Data Availability

Data supporting reported results that does not appear in the supplementary materials section may be supplied by the authors upon request.

## Supplementary Materials

The supplementary materials including NMR and IR spectra for the reported compounds are available online.

## Sample Availability

Samples of the compounds are available from the authors.
