# Supplementary material for: Synthesis of 6-Membered-Ring Fused Thiazine-Dicarboxylates and Thiazole-Pyrimidines via One-Pot Three-Component Reactions
Source: Molecules. 2021 Sep 10;26(18):5493. doi: 10.3390/molecules26185493 (PMC8467237; doi:10.3390/molecules26185493)
Supplement: Supplementary file 1 [file molecules-26-05493-s001.zip › molecules-1368554-supplementary.pdf]

Synthesis of 6-membered-ring fused thiazine-dicarboxylates and thiazole-pyrimidines *via* one-pot three-component reactions

Reagan L. Mohlala[1,2]; E. Mabel Coyanis[1*]; Muhammad Q. Fish[1], Manuel A. Fernandes[2]; Moira L. Bode[2*]

1 *Advanced Materials Division, Mintek, Private Bag X3015, Randburg, 2125, South Africa*
2 *Molecular Sciences Institute, School of Chemistry, University of the Witwatersrand, PO Wits, 2050, South Africa;*
*Correspondence: *mabelc@mintek.co.za (EM. Coyanis); Moira.Bode@wits.ac.za (M.L Bode)*

Table of Contents

1. Biological Evaluation Results

2. Biological Evaluation Methods

3. NMR and Mass Spectra for Compounds 9c/d, 10a–u, 13a–j, 19a/b, 20 and 8

4. CheckCIF Files for X-Ray Crystallography—Compounds 8, 13a, 13b and 13d

1. Biological evaluation results

The synthesized pyrimidines and their analogues **8**, **13**, **19** and **20** were evaluated for toxicity in the MT-4 cell line at 100 μM. MT-4 cells were originally isolated from an adult Japanese patient with acute lymphoblastic leukemia [1]. By determining the cytotoxicity of the compounds, it shows that they have the potential to kill cancer cells, as most of the compounds have values up to 7.3 μM. Auranofin [2] was used as a cytotoxic control compound, starting at a concentration of 100 μM. DMSO was used as a background control. Most of the compounds were considered as relatively toxic, with CC50 values below 100 μM. Further biological studies using a range of cancer cell lines and primary cells would need to be performed to determine if the observed toxicity is directed to particular cancer types or if it is stochastic. On the other hand, six of the thiazole-pyrimidine derivatives and analogues, namely **13a**, **13j**, **13h**, **13o**, **19a/b** and **20**, were found to have CC50 values above 200 μM, as shown in Table S1, whilst at least compounds 10c, 10m and 13e were found to have CC50 values of above 100 μM. These compounds, namely **3a**, **13j**, **13h**, **13o**, **19a/b** and **20**, can be considered to be relatively nontoxic and were subjected to antiviral activity evaluation. The antiviral activities were performed in infected MT4 cells, using dolutegravir and raltegravir as control compounds. The tests were carried out in a cell-based assay, and each compound was analyzed at a single dose of 100 μM. Based on the outcome, none of these compounds showed any significant activity.

Table S1: CC_{50} values and antiviral percentage inhibition of synthesised thiazine derivatives **10a-u**, **13a-j**, **19**, **20** and **8**

Compound	CC_{50} (μM)	% HIV Antiviral inhibition (in infected MT4 cells)
**10a**	7.3	
**10b**	12	
**10c**	102	
**10d**	51	
**10e**	16	
**10f**	33	
**10g**	33	
**10h**	51	
**10i**	49	
**10j**	80	
**10k**	25	
**10l**	20	
**10m**	120	
**10n**	17	
**10o**	21	
**10p**	16	
**10q**	39	
**10r**	26	
**10s**	57	
**10t**	33	
**10u**	27	
**13a**	> 200	-7.18
**13b**	59	

**13c**	17	
**13d**	101	
**13e**	> 200	0.98
**13f**	36	
**13g**	> 200	-2.63
**13h**	14	
**13i**	17	
**13j**	> 200	-9.18
**19a**	> 200	-4.83
**19b**	> 200	1.19
**20**	> 200	0.55
**8**	27	-7.18

CC_{50} - concentration of compounds that causes 50% reduction of cell growth

Most of the synthesised thiazines **10** and pyrimidines **13** were shown to have toxicity towards a leukaemia cell line used in this study, with the most toxic being **10a**, indicating potential anticancer effects. Future work will be directed towards determining if this toxicity is specific or stochastic.

2. Biological evaluation methods

2.1 Cytotoxicity

The day of cytotoxicity testing, MT-4 cells (obtained through the NIH AIDS Reagent Program, Division of AIDS, NIAID, NIH: MT-4 catalogue number 120) were counted and seeded at 5×104 cells per well. A total of 100 μL of cells was added to each well of 96-well cell culture plates (TTP) for testing. The volume in the last row of wells of each plate was further increased to 197 μL of media containing MT-4 cells. The plate was placed into the incubator to equilibrate to 37 °C and 5% CO_2. Test compounds were prepared in plate format, and each compound was at 25 mM stock concentration in DMSO. A total of 3.2 μL compound was added to the final row of wells containing 197 μL cells and mixed to ensure the solution was homogeneous with the cells. A two-fold serial dilution of an eight-times series was performed, and the final 100 μL was discarded. The plate was placed at 37 °C and 5% CO_2 for 5 days. On the fifth day, the cell viability was assessed by using the Cell Titre 96® Aqueous One Solution Cell Proliferation assay (Promega) as per the manufacturer's instructions. Briefly, 20 μL of MTS solution was added to each well and incubated at 37 °C for 4 hours. The absorbance of the plates was then read at 490 nm on a Spectra Max Spectrophotometer (Molecular Devices). The data analysis was completed on Origin 8.1 with the log value of the compounds molar concentration plotted against the percentage cell viability determined by the absorbance level by sigmoidal fitting. From the curve, a half maximal cytotoxicity CC50 for compounds was determined. Auranofin was used as a cytotoxic control compound, starting at 100 μM, across a two-fold serial dilution of eight dilution series. DMSO was used as a background control and two-fold serially diluted from 1.5%.

2.2 Antiviral procedure

An HIV-1 chronically infected ACH-2 clone was obtained through the NIH HIV Reagent Program, Division of AIDS, NIAID, NIH: ACH-2 Cells, ARP-349, contributed by Dr. Thomas Folks. The cells were grown and maintained in RPMI 1640 (Sigma) supplemented with 10 mM HEPES, 2 mM L-glutamine, non-essential amino acids and 10% heat inactivated fetal bovine serum at 37 °C and 5% CO_2. The ACH-2 cells were activated by 1 μM phorbol myristate acetate (PMA) [3]. Supernatants were harvested at 48 h by centrifugation at 4000 x g and stored at −80 °C until use. MT4 cells were grown and maintained in RPMI (Gibco) supplemented with 2 mM L-glutamine, non-essential amino acids and 10% heat-inactivated fetal bovine serum at 37 °C and 5% CO_2. Harvested supernatant containing HIV-1 was added to MT4 cells, and viral absorption was allowed for 24 h, similar to the method by Rosenwirth and co-workers [4]. After viral absorption, the cells were washed once with fresh media, a sample was taken and HIV1 infection was allowed to develop. Seven days after infection, MT4 supernatant samples were tested for the presence of p24 antigen, using the bioelisa HIV-1+2 Ag/Ab kit (biokit) as per the manufacturer's instructions. After HIV-1 infection was confirmed, supernatant samples were isolated by centrifugation at 4000 x g and immediately used to infect 1x105 cells/ml of MT4 cells. The virus was allowed to adsorb, and 24 h later, the cells were washed with fresh media and, 100 μL of cells, 1x104 cells, was

aliquoted into a round-bottom 96-well plate (TPP). Uninfected cells were also prepared. A day 1, a sample was taken and stored at -80 °C until final testing. Compounds were diluted in fresh media to 200 μM concentrations, and 100 μL was added to each test well; the final concentration of compounds was 100 μM. Control compounds include dolutegravir and raltegravir and were used at 10 μM each; blank wells contained equal volumes of DMSO. Upper and lower asymptotes were determined by untreated MT4 cells either infected with HIV (100%) or not infected with HIV (0%). Four days after infection, p24 presence was determined by using the bioelisa HIV-1+2 Ag/Ab kit as per the manufacturer's instructions. The data were used to determine if the selected concentration of compounds would reduce the amount of p24 and thereby indicate a reduction in HIV infectivity.

3. NMR and Mass Spectra

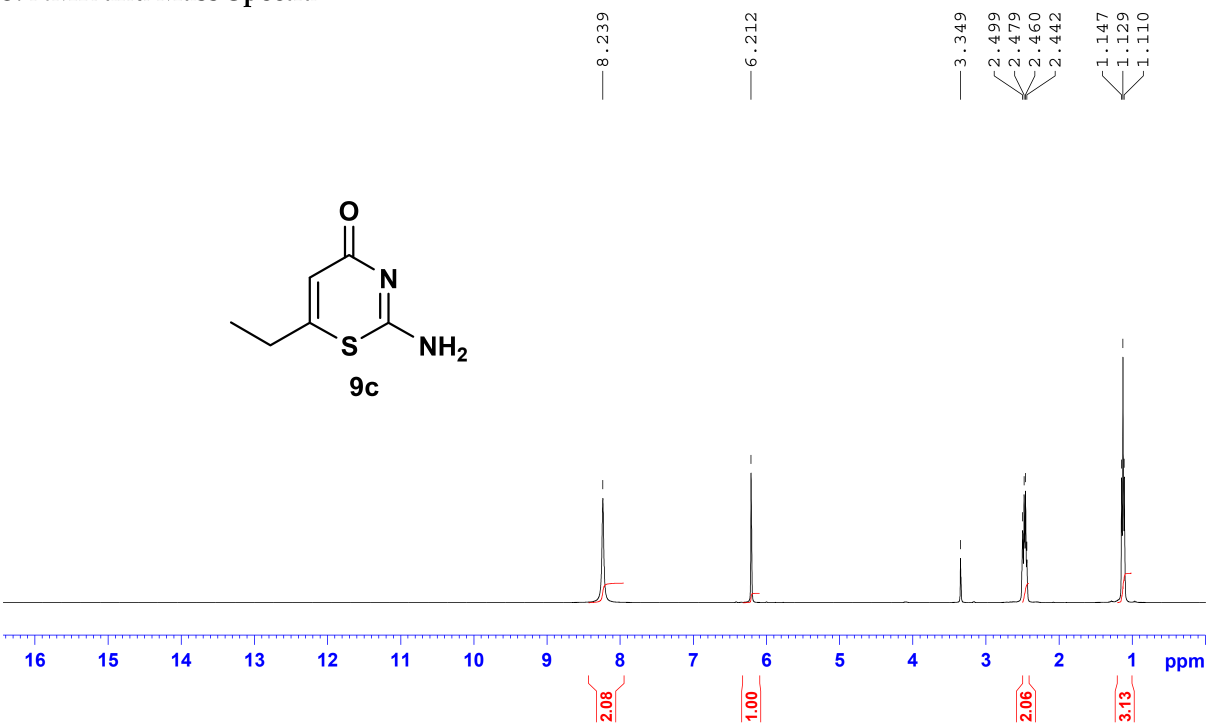

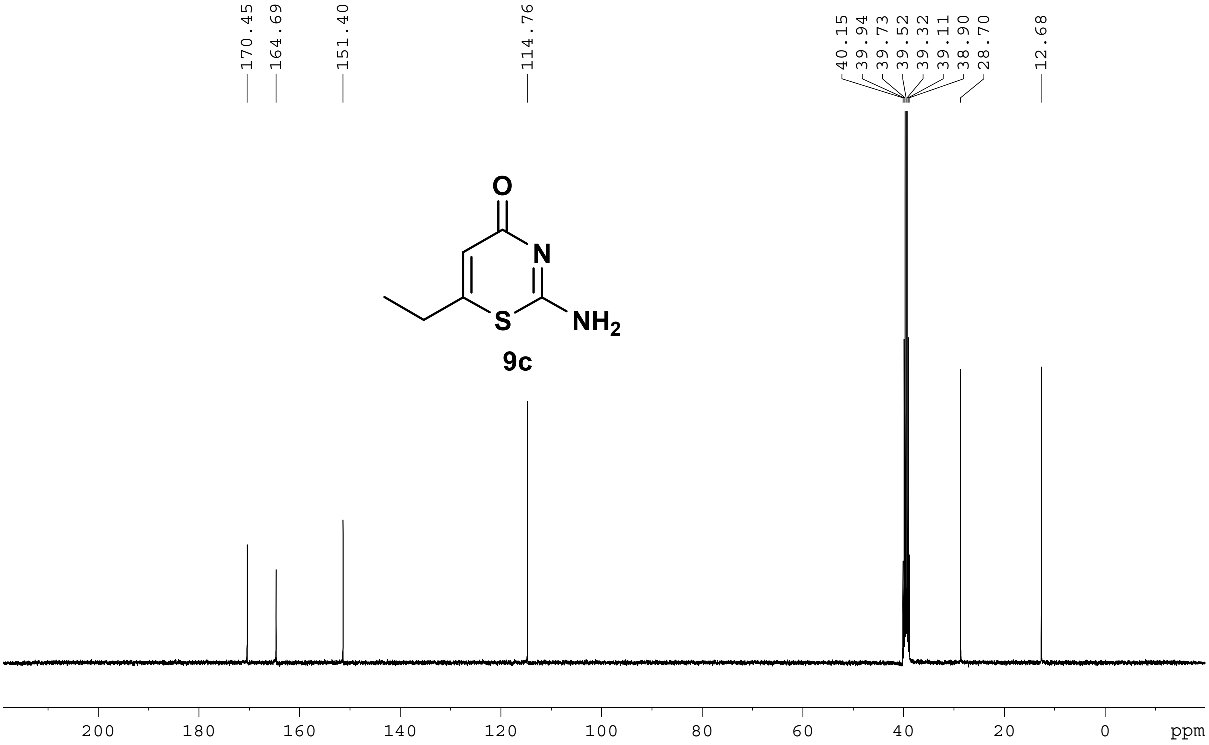
170.45
164.69
151.40
114.76
40.15
39.94
39.73
39.52
39.32
39.11
38.90
28.70
12.68
O
N
S
NH2
9c
200
180
160
140
120
100
80
60
40
20
0
ppm

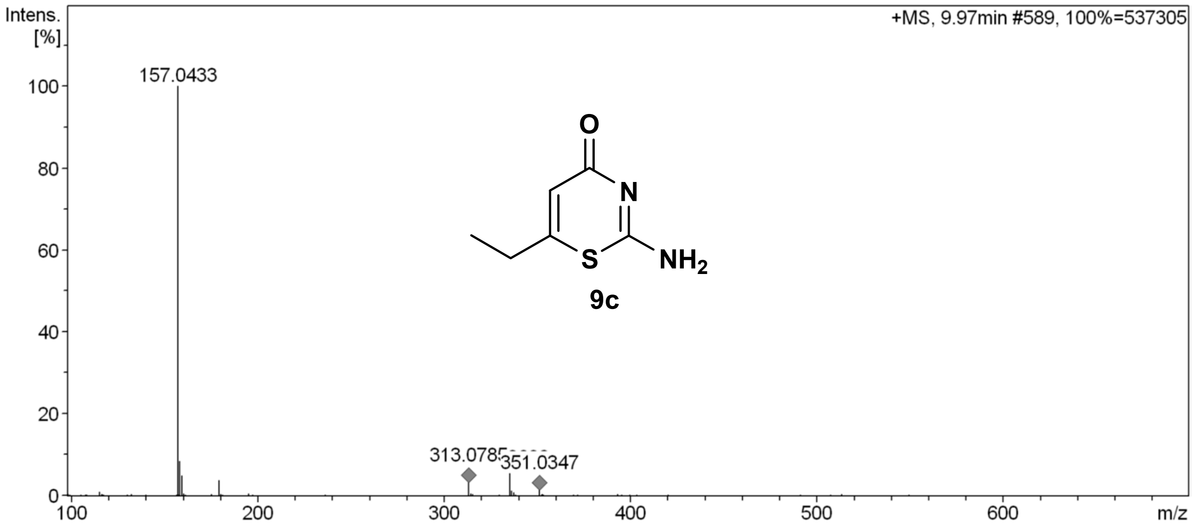
Intens.
[%]
+MS, 9.97min #589, 100%=537305
157.0433
313.0785
351.0347
O
N
S
NH2
9c
100
80
60
40
20
0
100
200
300
400
500
600
m/z

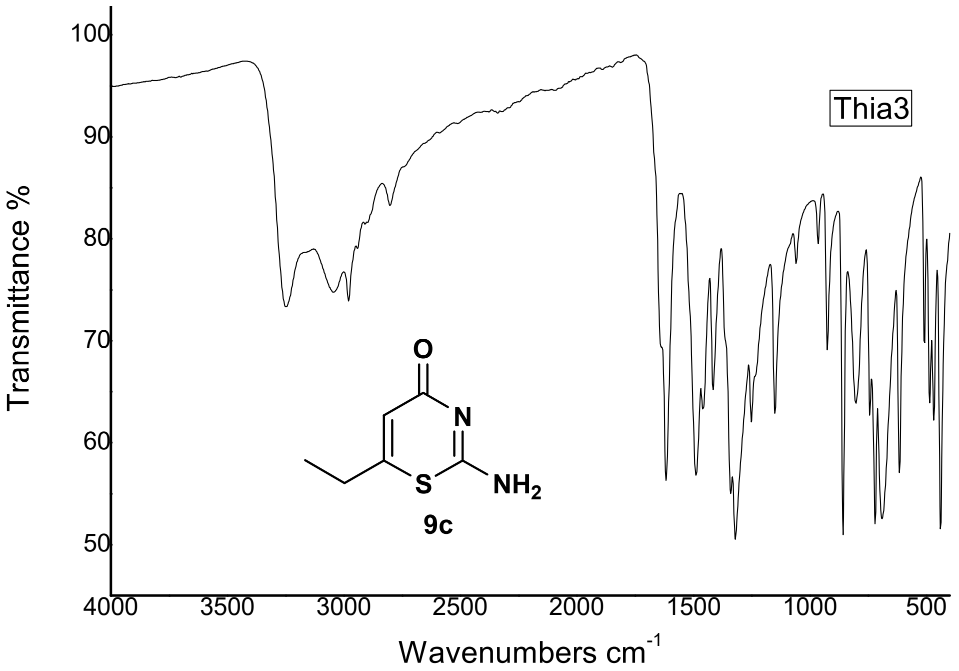
Thia3
Transmittance %
100
90
80
70
60
50
4000
3500
3000
2500
2000
1500
1000
500
Wavenumbers cm-1
O
N
S
NH2
9c

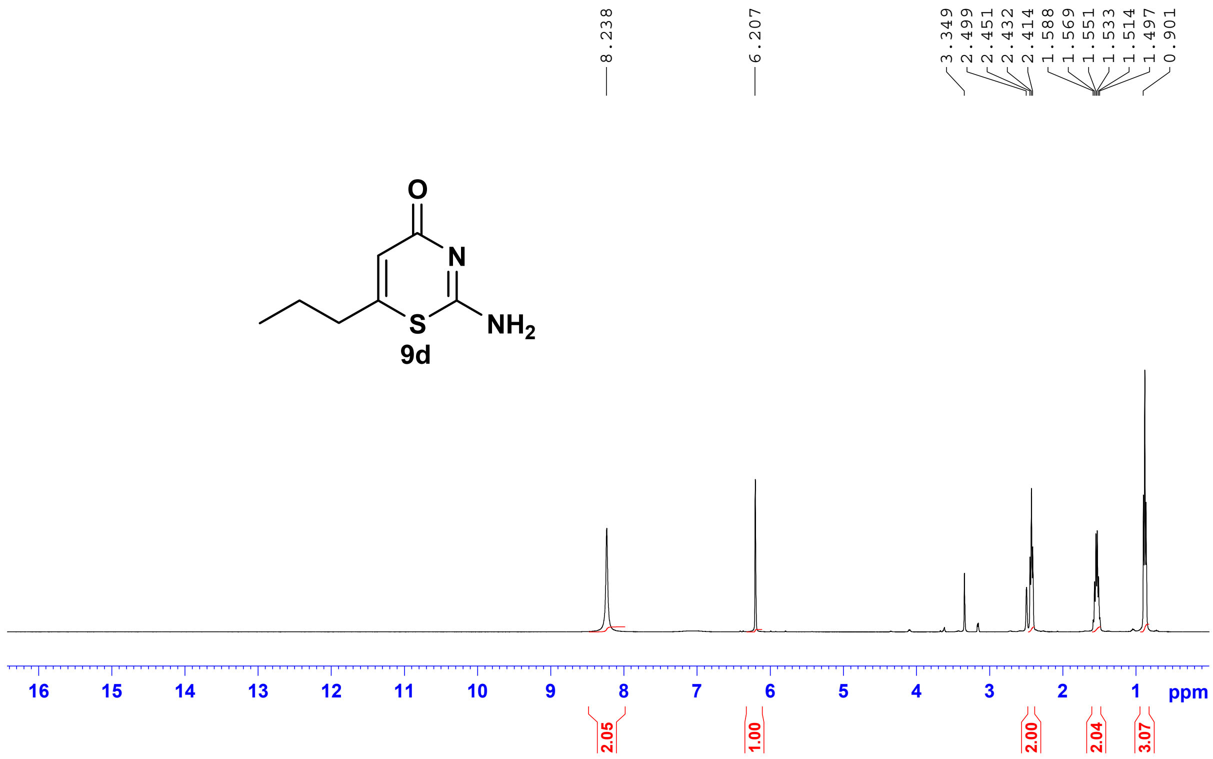
8.238
6.207
3.349
2.499
2.451
2.432
2.414
1.588
1.569
1.551
1.533
1.514
1.497
0.901
O
N
S
NH2
9d
16
15
14
13
12
11
10
9
8
7
6
5
4
3
2
1
ppm
2.05
1.00
2.00
2.04
3.07

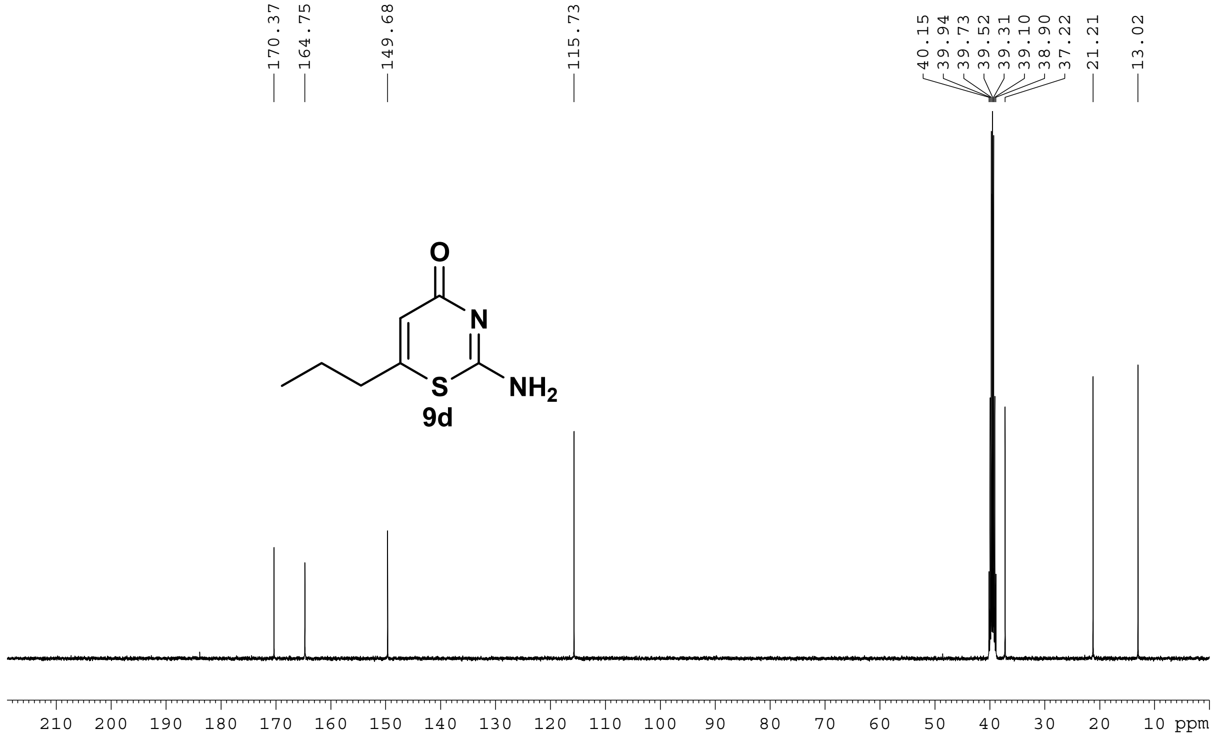
170.37
164.75
149.68
115.73
40.15
39.94
39.73
39.52
39.31
39.10
38.90
37.22
21.21
13.02
O
N
S
NH2
9d
210 200 190 180 170 160 150 140 130 120 110 100 90 80 70 60 50 40 30 20 10 ppm

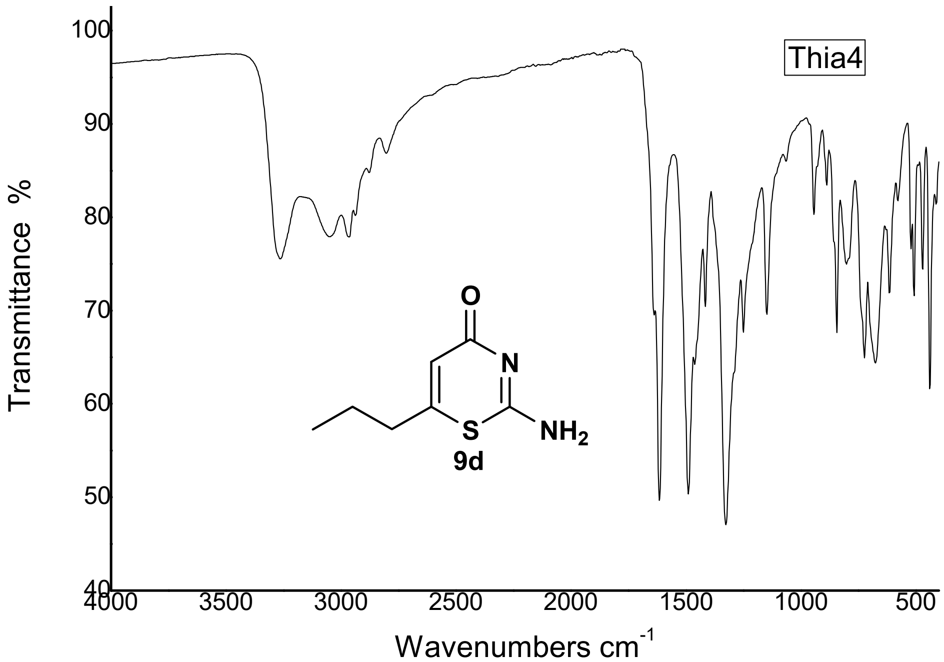
Thia4
Transmittance %
100
90
80
70
60
50
40
4000 3500 3000 2500 2000 1500 1000 500
Wavenumbers cm-1
O
N
S
NH2
9d

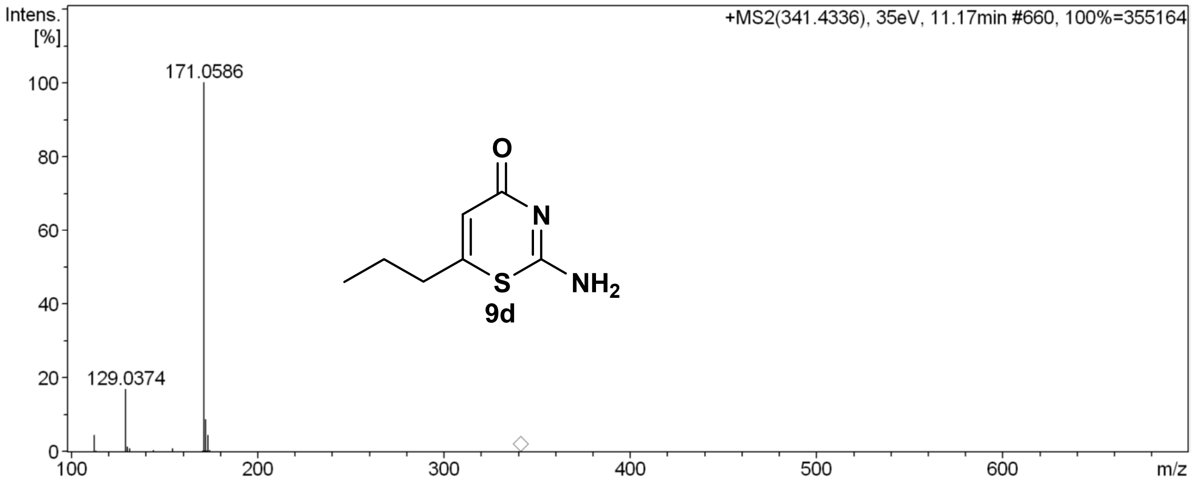

4-Oxo-4,6-dihydropyrimido[2,1-*b*][1,3]thiazine-6,7-dicarboxylate derivatives

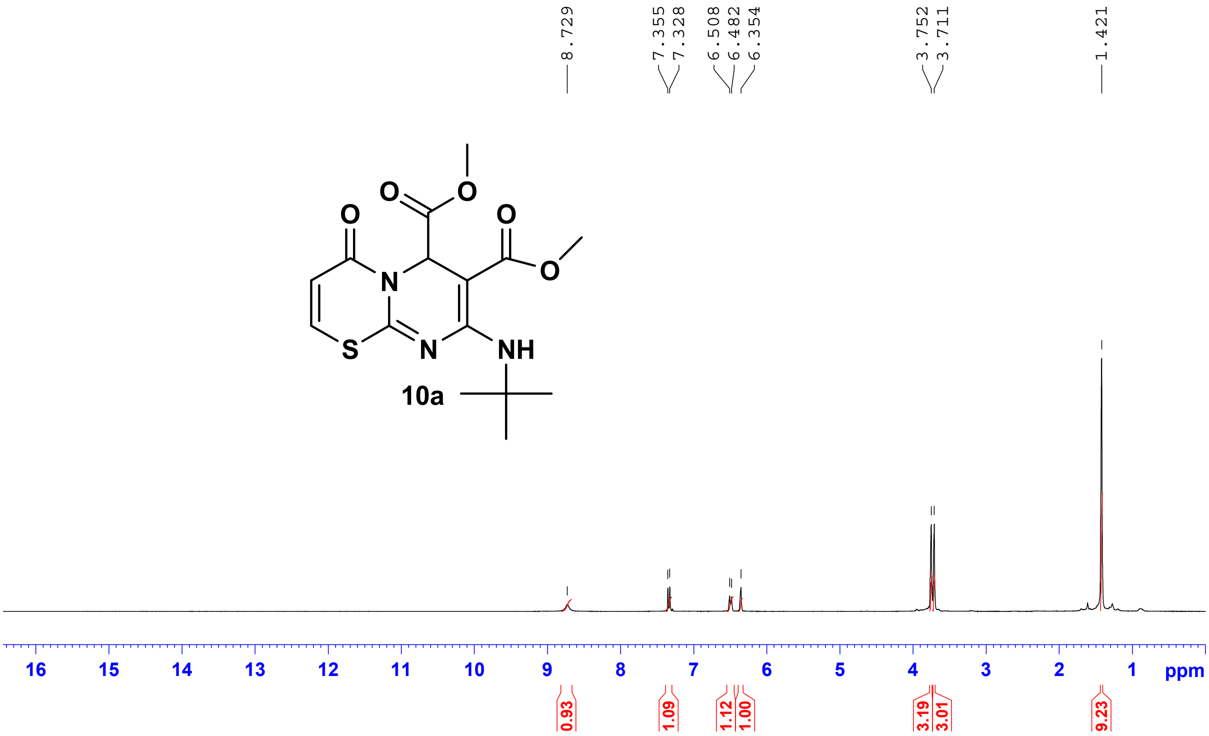

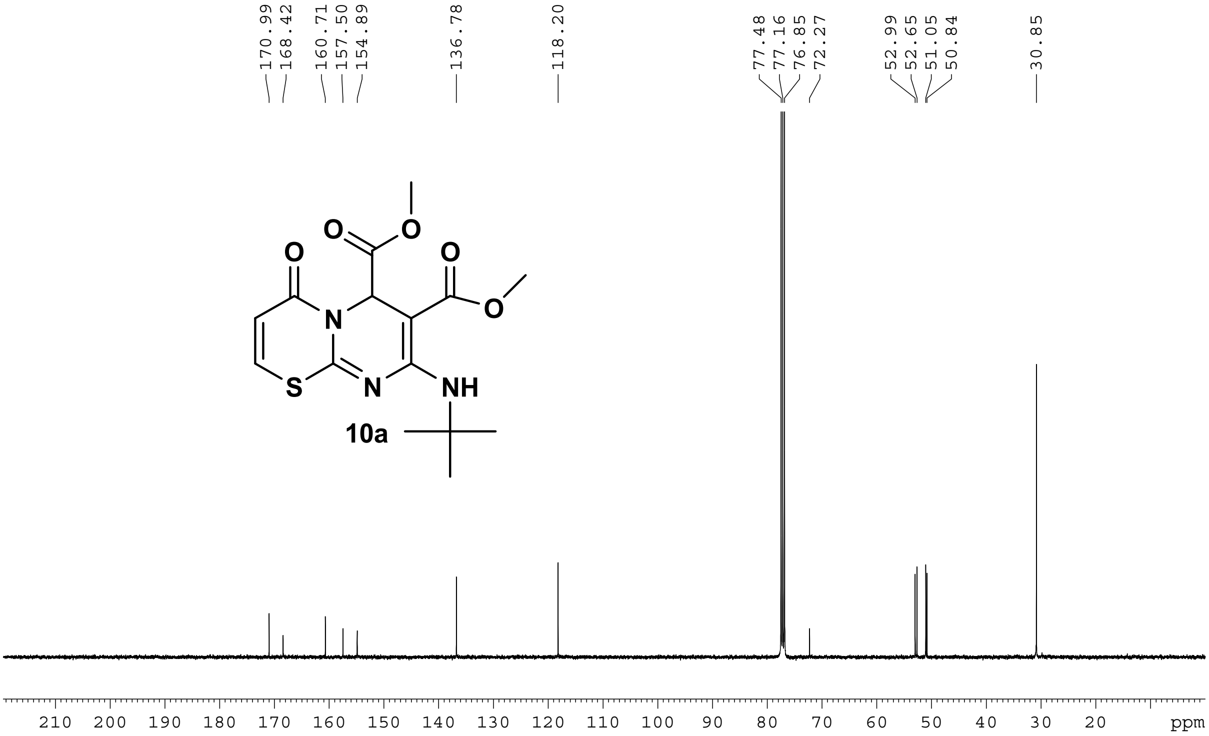
170.99
168.42
160.71
157.50
154.89
136.78
118.20
77.48
77.16
76.85
72.27
52.99
52.65
51.05
50.84
30.85
10a
210 200 190 180 170 160 150 140 130 120 110 100 90 80 70 60 50 40 30 20
ppm

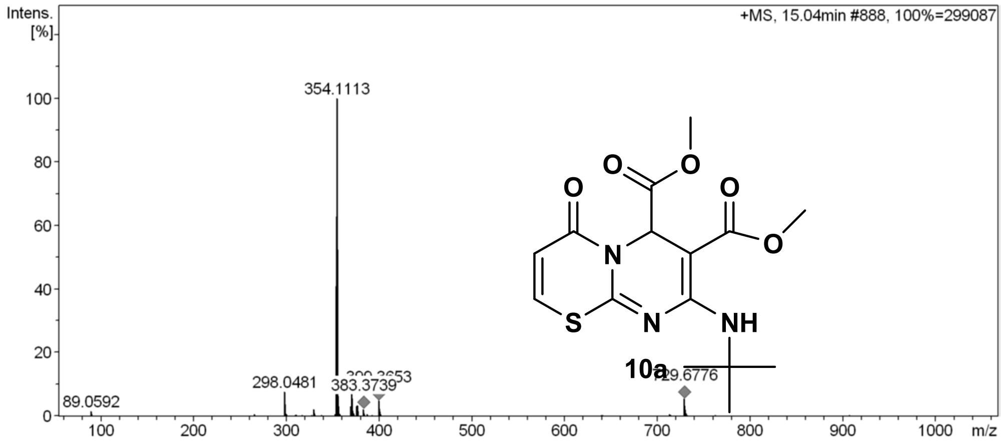
Intens.
[%]
+MS, 15.04min #888, 100%=299087
354.1113
298.0481
383.3739
89.0592
10a
100 200 300 400 500 600 700 800 900 1000
m/z

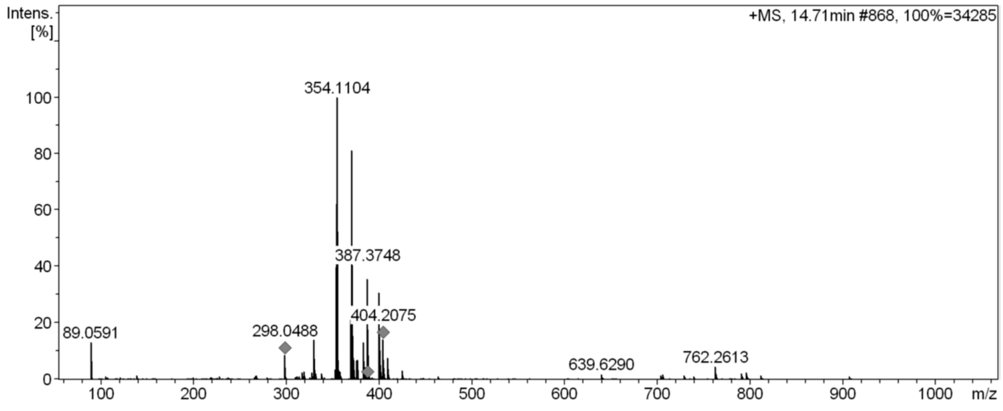
Intens.
[%]
+MS, 14.71min #868, 100%=34285
354.1104
387.3748
404.2075
89.0591
298.0488
639.6290
762.2613
100 200 300 400 500 600 700 800 900 1000
m/z

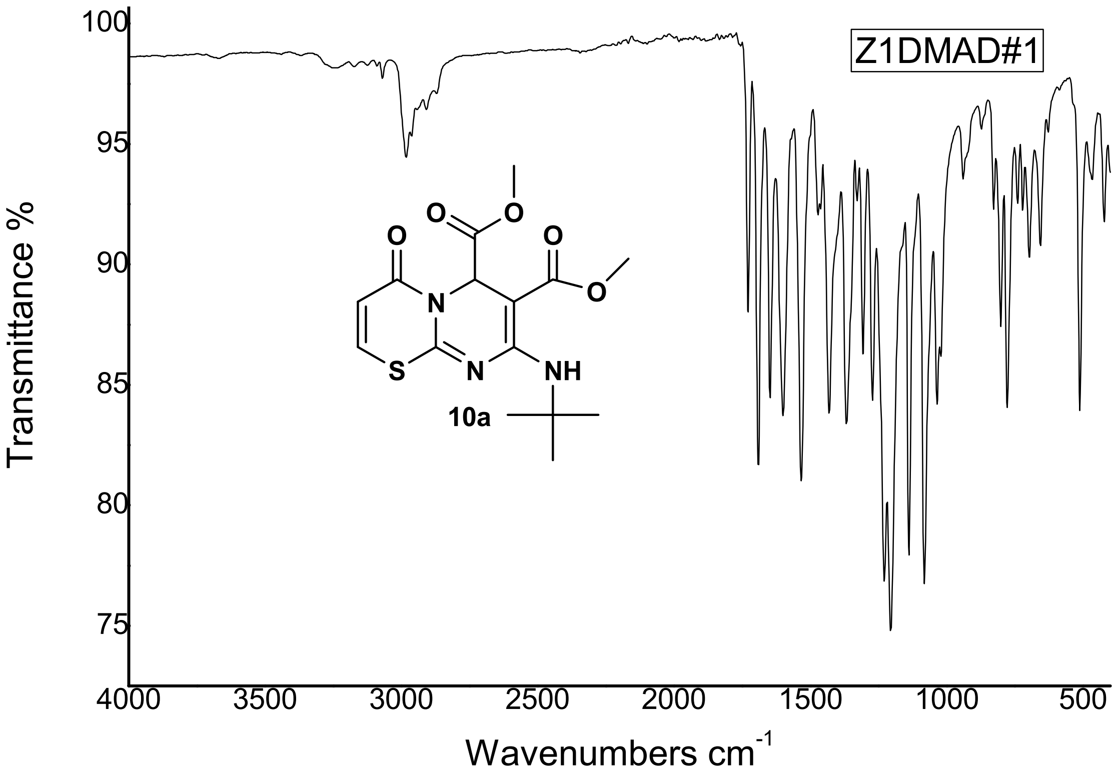
Z1DMAD#1
Transmittance %
Wavenumbers cm-1
10a
O
N
S
NH

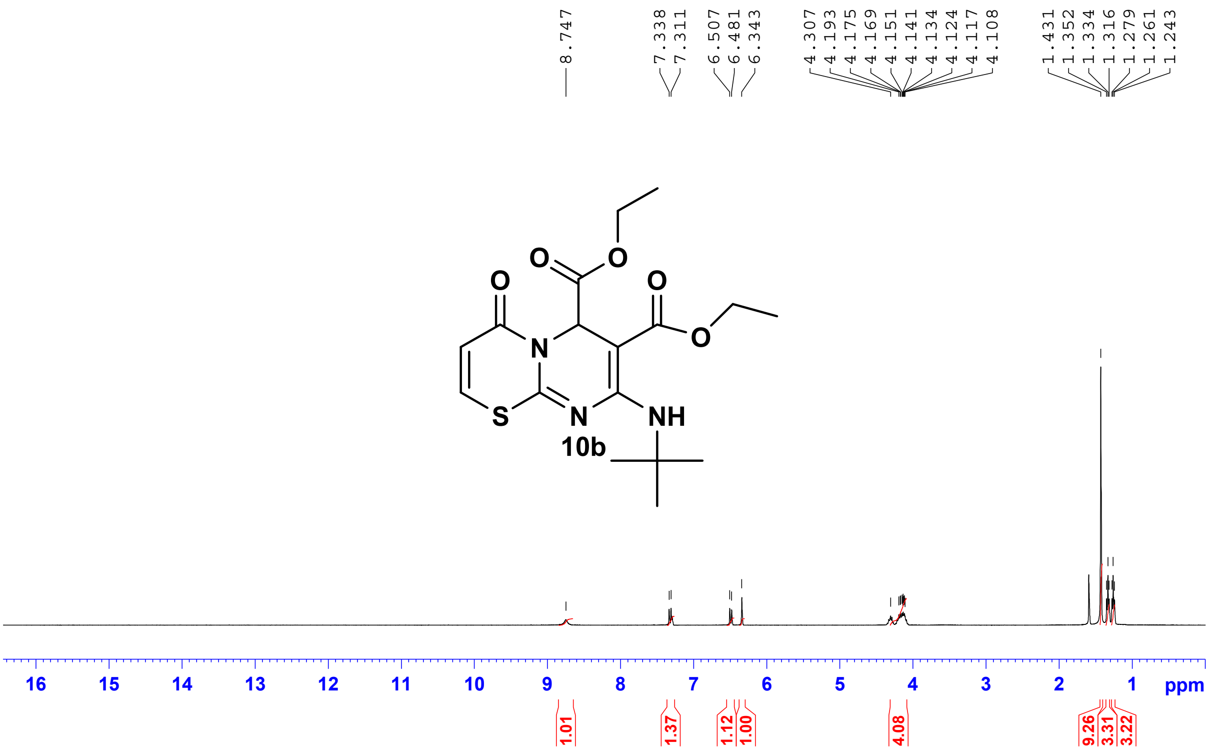
8.747
7.338
7.311
6.507
6.481
6.343
4.307
4.193
4.175
4.169
4.151
4.141
4.134
4.124
4.117
4.108
1.431
1.352
1.334
1.316
1.279
1.261
1.243
10b
ppm
1.01
1.37
1.12
1.00
4.08
9.26
3.31
3.22

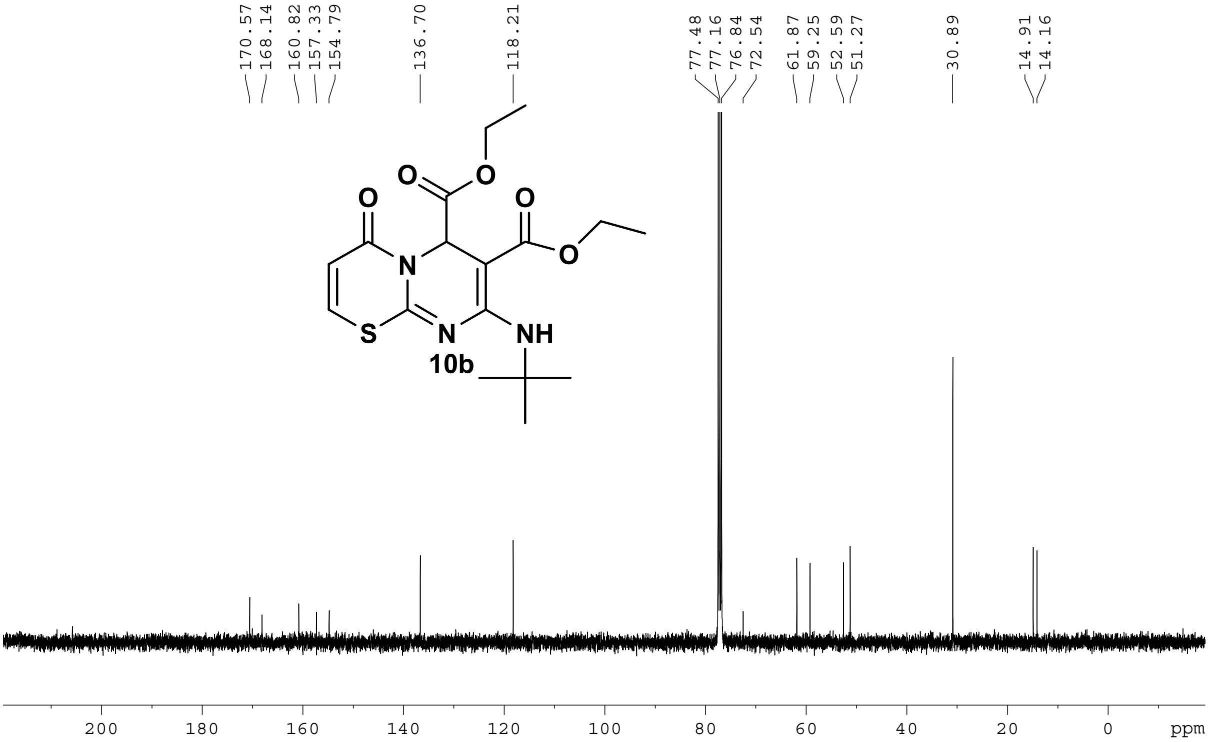

170.57
168.14
160.82
157.33
154.79
136.70
118.21
77.48
77.16
76.84
72.54
61.87
59.25
52.59
51.27
30.89
14.91
14.16
10b
200
180
160
140
120
100
80
60
40
20
0
ppm

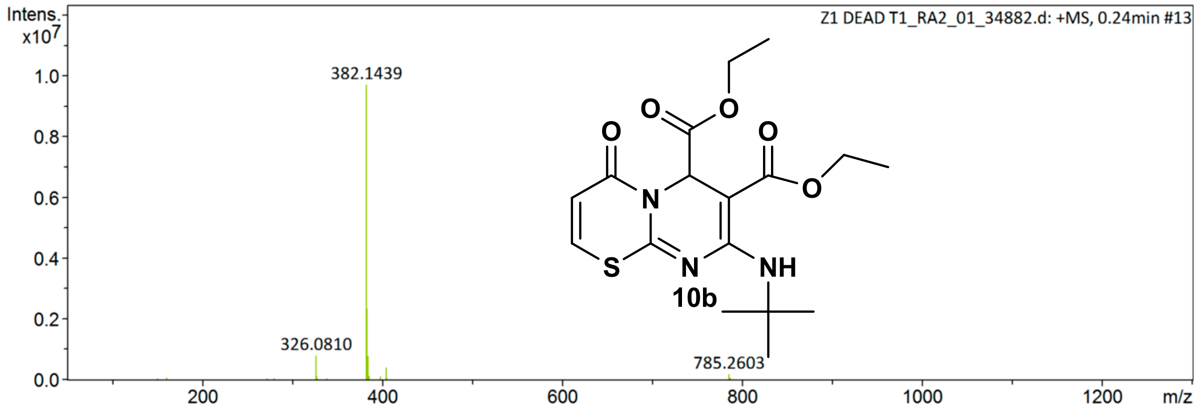

Intens.
x10^7
Z1 DEAD T1_RA2_01_34882.d: +MS, 0.24min #13
382.1439
326.0810
785.2603
10b
1.0
0.8
0.6
0.4
0.2
0.0
200
400
600
800
1000
1200
m/z

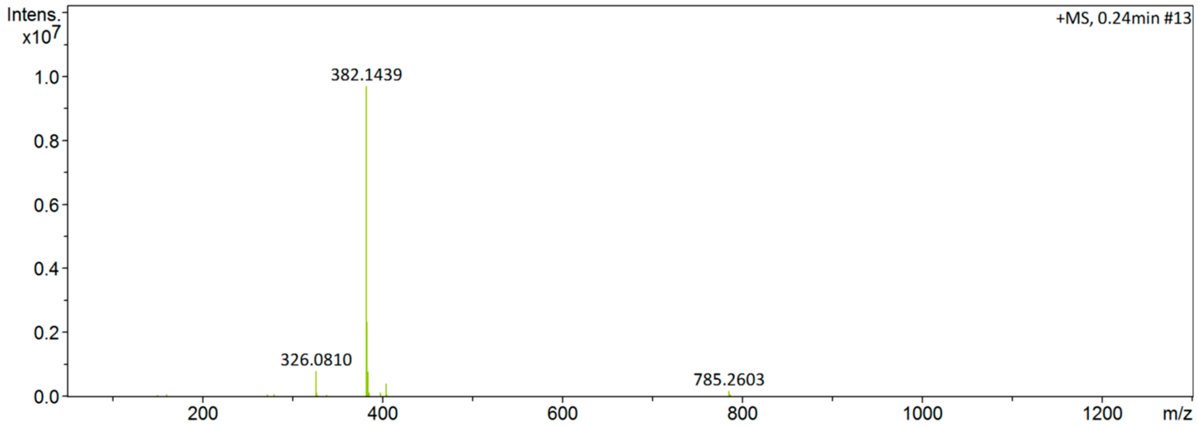

Intens.
x10^7
+MS, 0.24min #13
382.1439
326.0810
785.2603
1.0
0.8
0.6
0.4
0.2
0.0
200
400
600
800
1000
1200
m/z

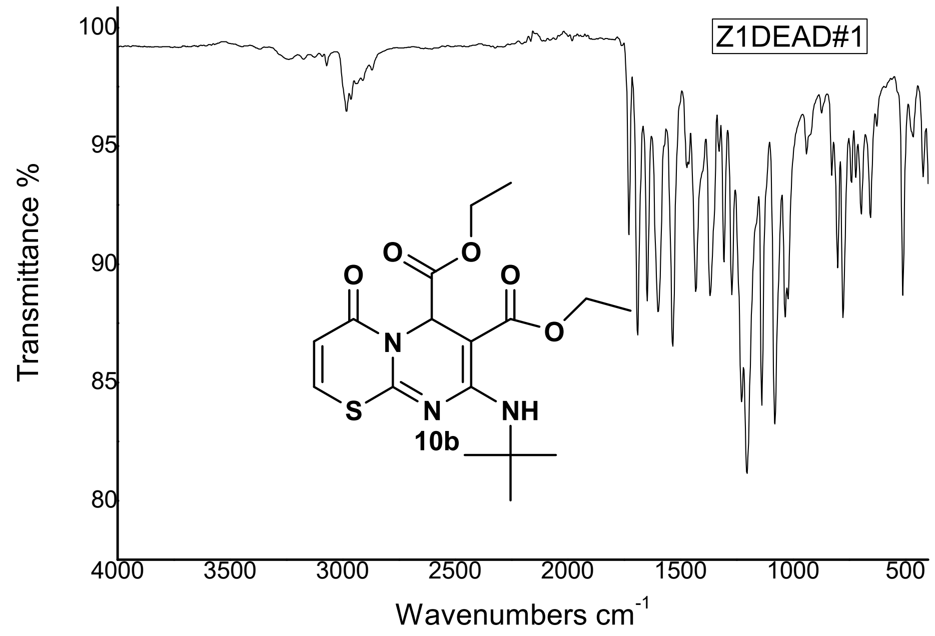
Z1DEAD#1
Transmittance %
100
95
90
85
80
4000
3500
3000
2500
2000
1500
1000
500
Wavenumbers cm-1
O
O
O
O
O
N
S
N
NH
10b

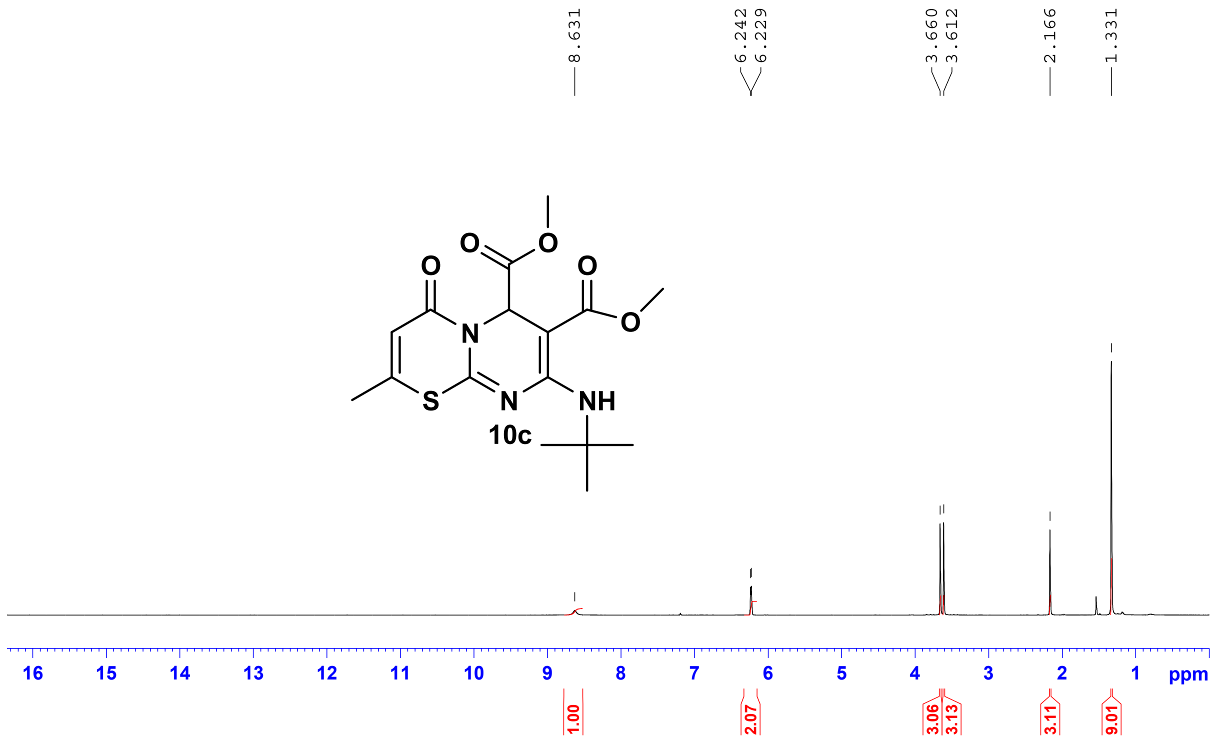
8.631
6.242
6.229
3.660
3.612
2.166
1.331
O
O
O
O
O
N
S
N
NH
10c
16
15
14
13
12
11
10
9
8
7
6
5
4
3
2
1
ppm
1.00
2.07
3.06
3.13
3.11
9.01

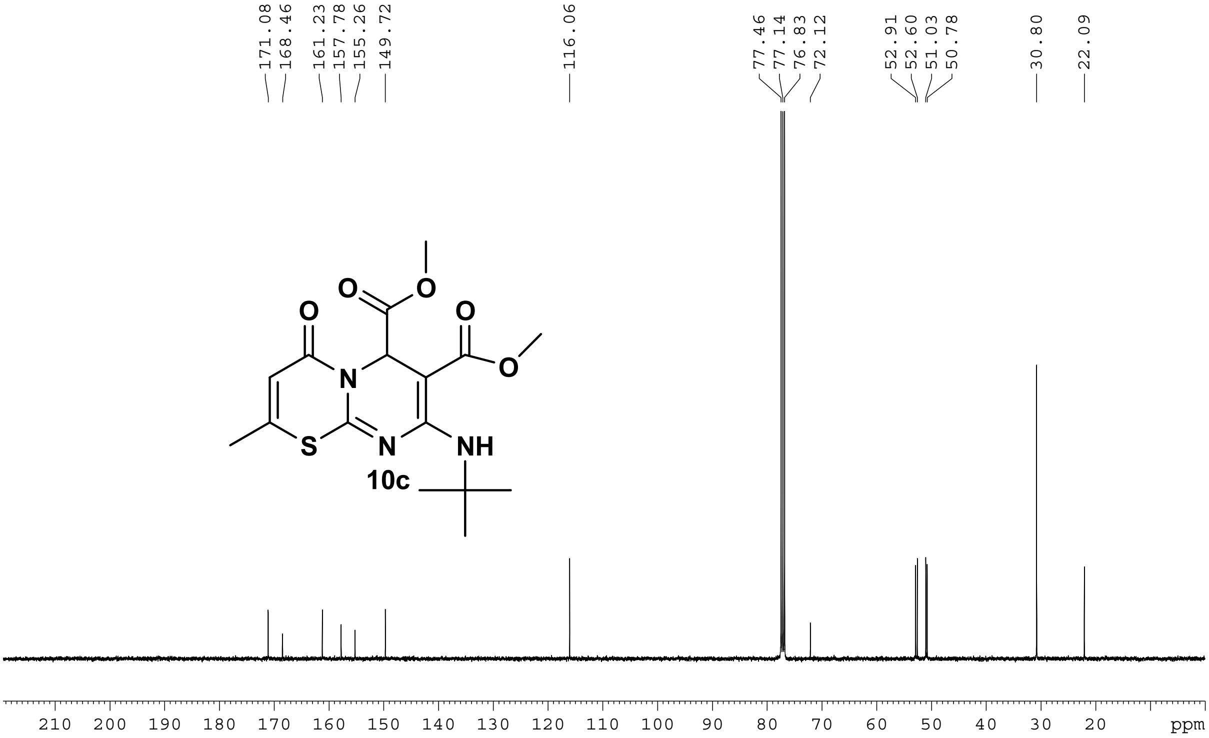
171.08
168.46
161.23
157.78
155.26
149.72
116.06
77.46
77.14
76.83
72.12
52.91
52.60
51.03
50.78
30.80
22.09
10c
210 200 190 180 170 160 150 140 130 120 110 100 90 80 70 60 50 40 30 20 ppm

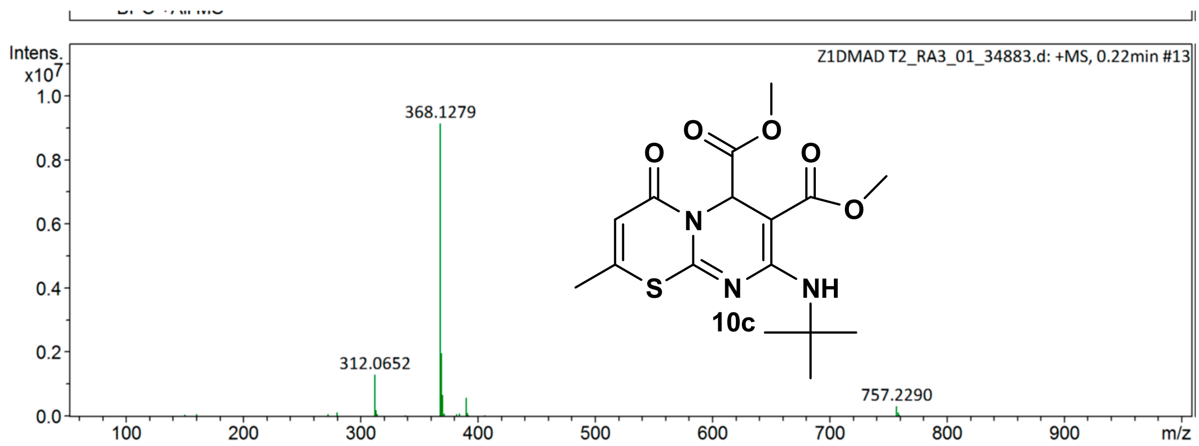
Intens.
x10^7
Z1DMAD T2_RA3_01_34883.d: +MS, 0.22min #13
368.1279
312.0652
757.2290
10c
m/z

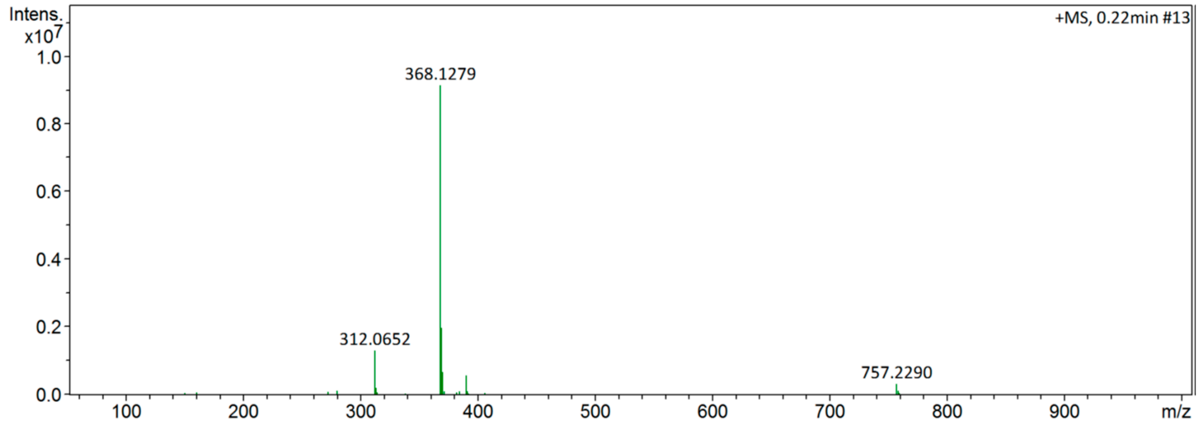
Intens.
x10^7
+MS, 0.22min #13
368.1279
312.0652
757.2290
m/z

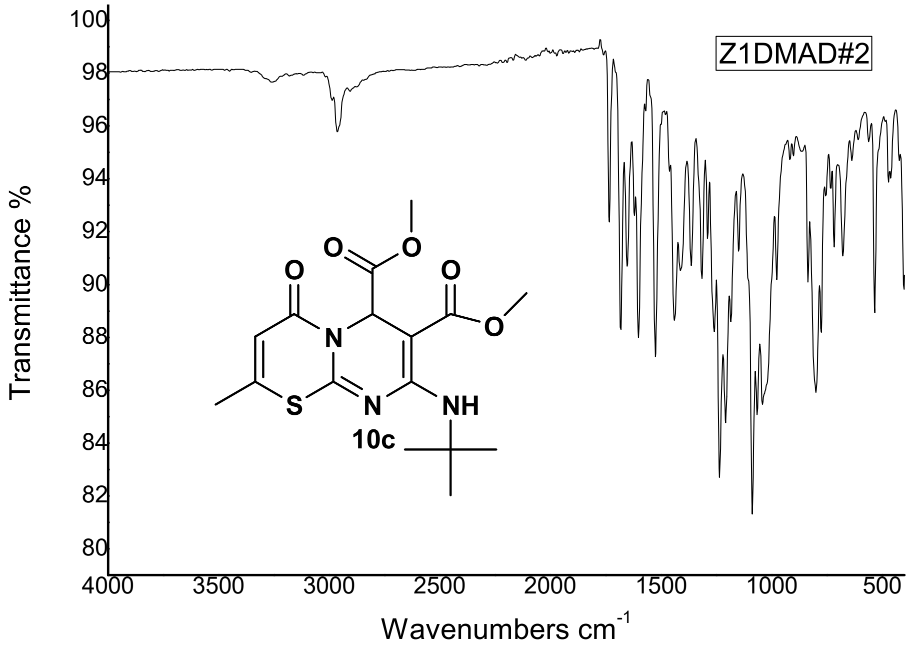
Z1DMAD#2
Transmittance %
Wavenumbers cm-1
100
98
96
94
92
90
88
86
84
82
80
4000
3500
3000
2500
2000
1500
1000
500
O
N
S
NH
10c

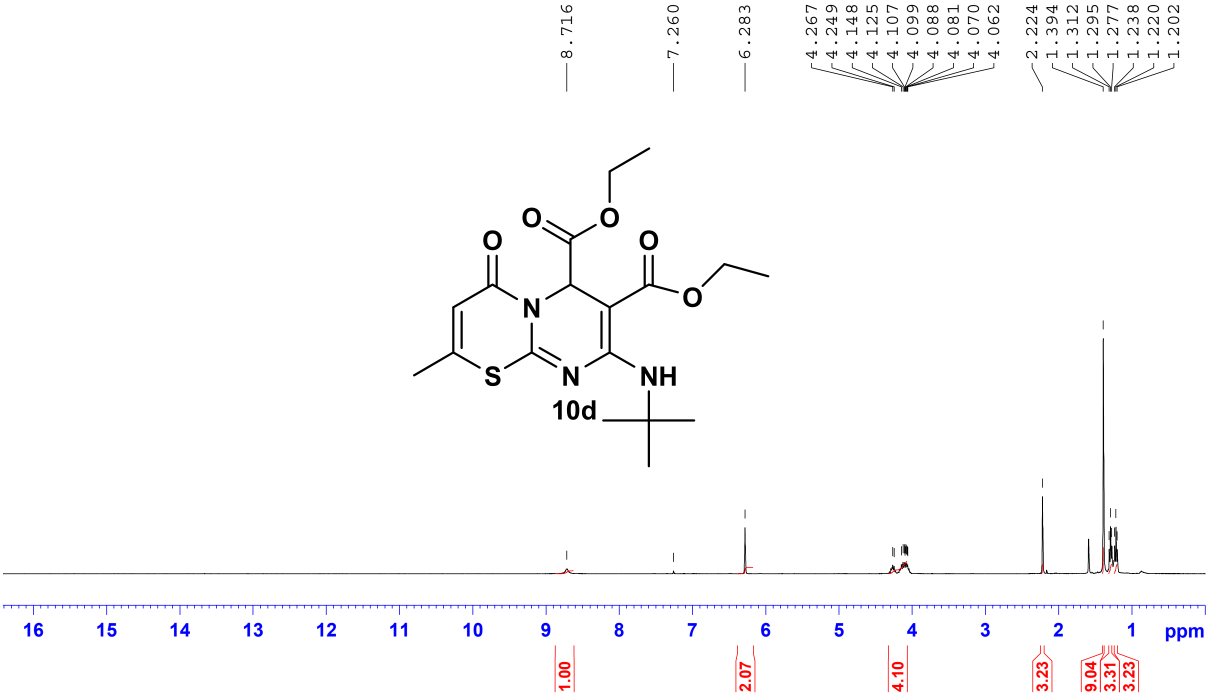
8.716
7.260
6.283
4.267
4.249
4.148
4.125
4.107
4.099
4.088
4.081
4.070
4.062
2.224
1.394
1.312
1.295
1.277
1.238
1.220
1.202
O
N
S
NH
10d
16
15
14
13
12
11
10
9
8
7
6
5
4
3
2
1
ppm
1.00
2.07
4.10
3.23
9.04
3.31
3.23

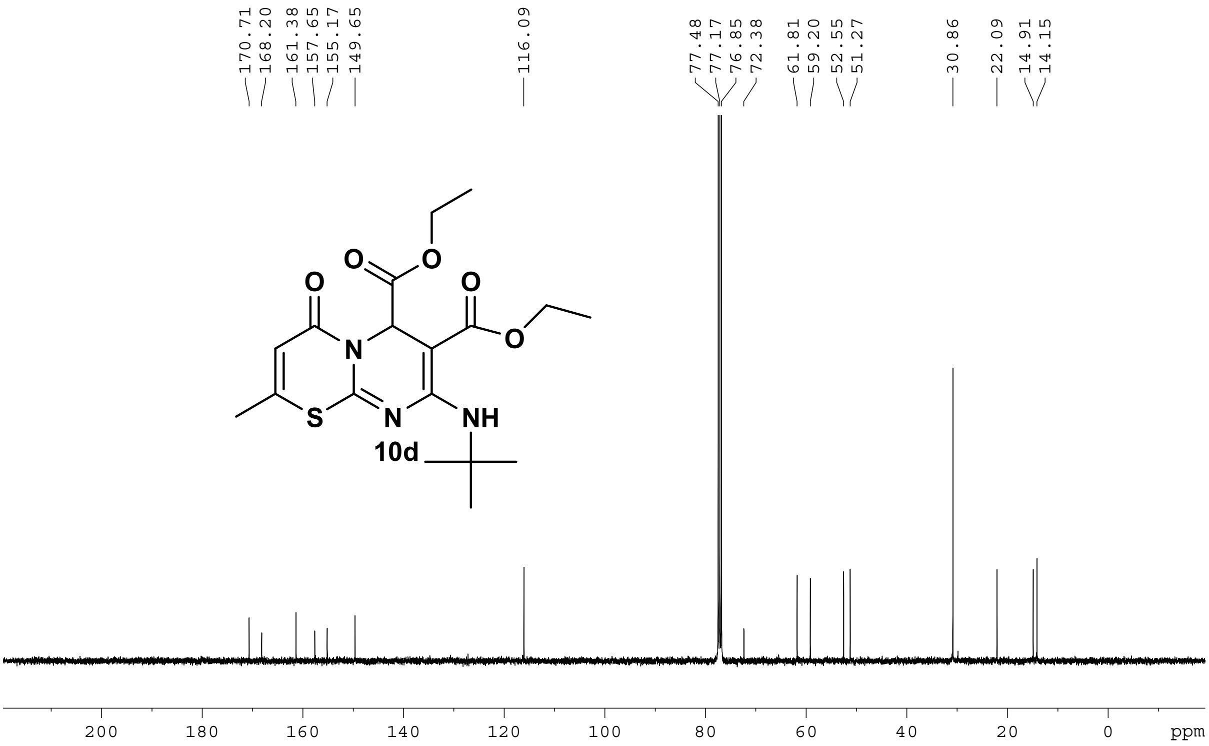
170.71
168.20
161.38
157.65
155.17
149.65
116.09
77.48
77.17
76.85
72.38
61.81
59.20
52.55
51.27
30.86
22.09
14.91
14.15
O
O
O
O
O
N
S
N
NH
10d
200
180
160
140
120
100
80
60
40
20
0
ppm

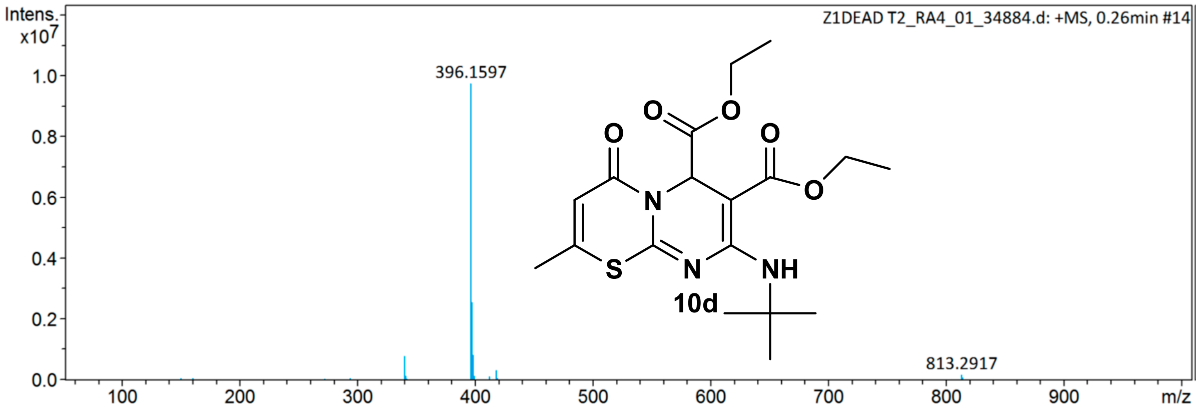
Intens.
x10^7
1.0
0.8
0.6
0.4
0.2
0.0
Z1DEAD T2_RA4_01_34884.d: +MS, 0.26min #14
396.1597
813.2917
O
O
O
O
O
N
S
N
NH
10d
100
200
300
400
500
600
700
800
900
m/z

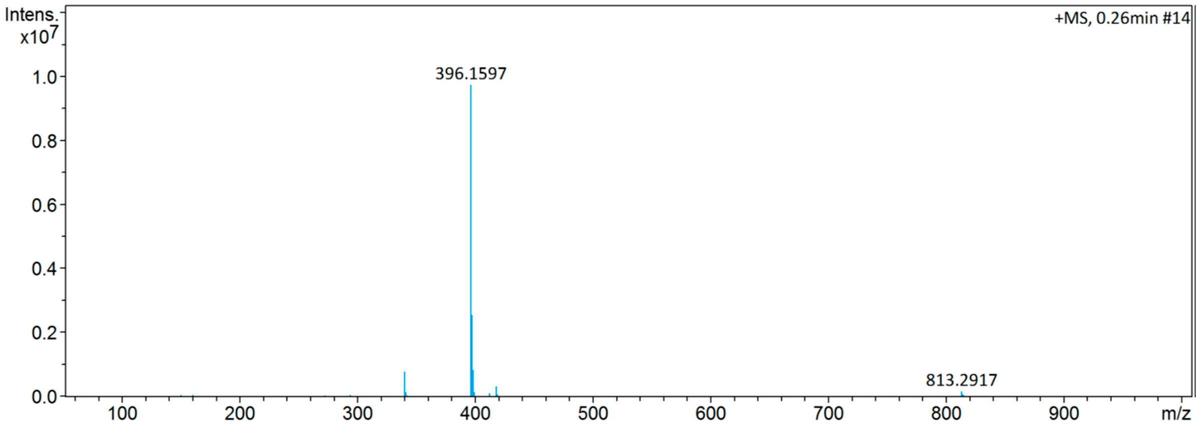
Intens.
x10^7
1.0
0.8
0.6
0.4
0.2
0.0
+MS, 0.26min #14
396.1597
813.2917
100
200
300
400
500
600
700
800
900
m/z

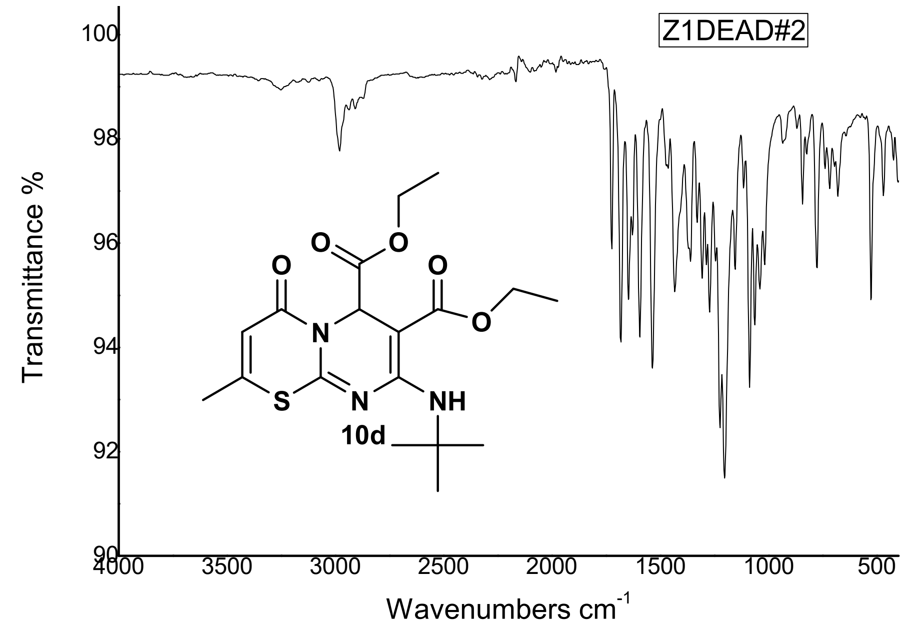
Z1DEAD#2
100
98
96
94
92
90
Transmittance %
4000
3500
3000
2500
2000
1500
1000
500
Wavenumbers cm-1
10d

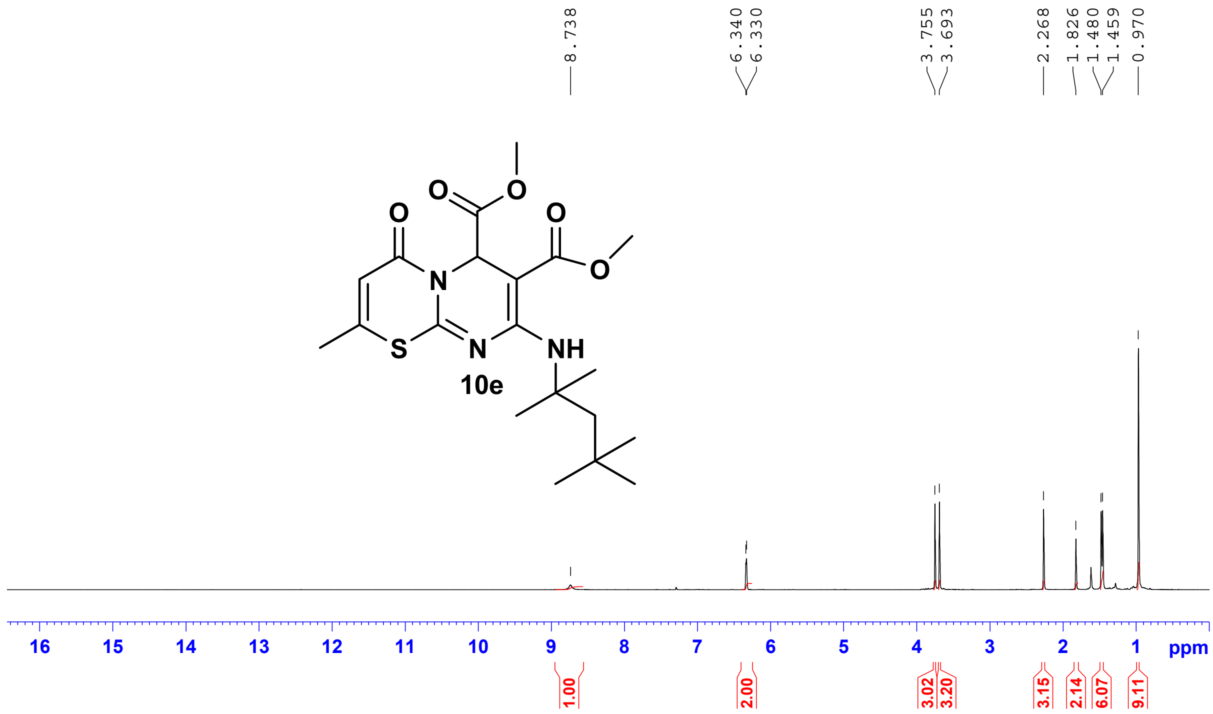
8.738
6.340
6.330
3.755
3.693
2.268
1.826
1.480
1.459
0.970
10e
16
15
14
13
12
11
10
9
8
7
6
5
4
3
2
1
ppm
1.00
2.00
3.02
3.20
3.15
2.14
6.07
9.11

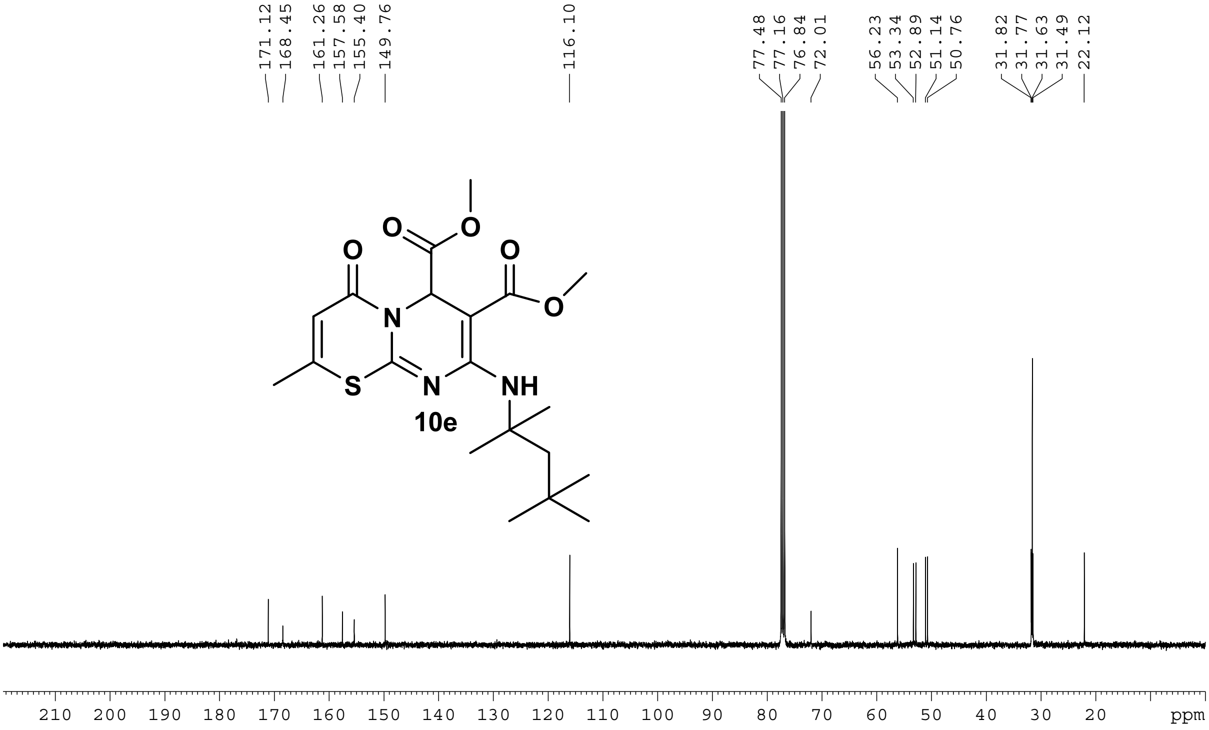
171.12
168.45
161.26
157.58
155.40
149.76
116.10
77.48
77.16
76.84
72.01
56.23
53.34
52.89
51.14
50.76
31.82
31.77
31.63
31.49
22.12
10e
210 200 190 180 170 160 150 140 130 120 110 100 90 80 70 60 50 40 30 20 ppm

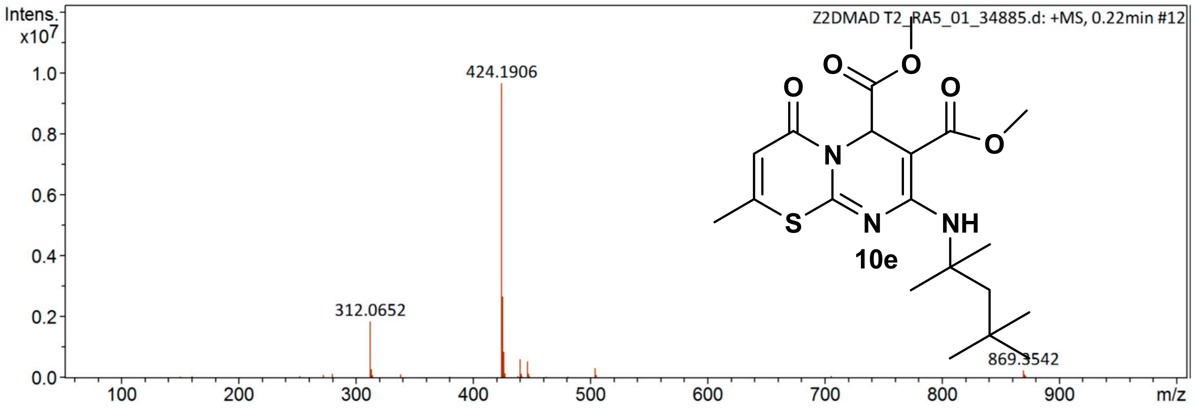
Intens.
x10^7
Z2DMAD T2_RA5_01_34885.d: +MS, 0.22min #12
424.1906
312.0652
869.3542
10e
m/z

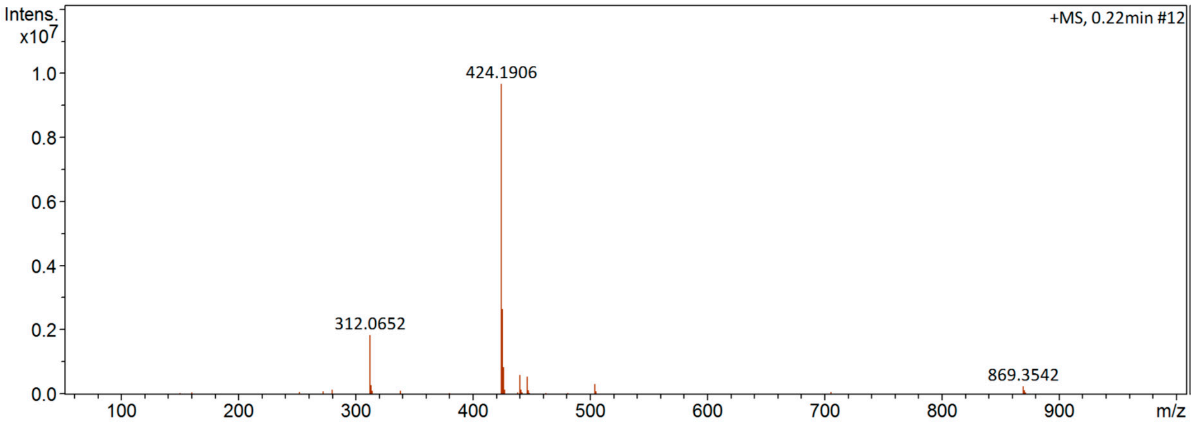
Intens.
x10^7
+MS, 0.22min #12
424.1906
312.0652
869.3542
m/z

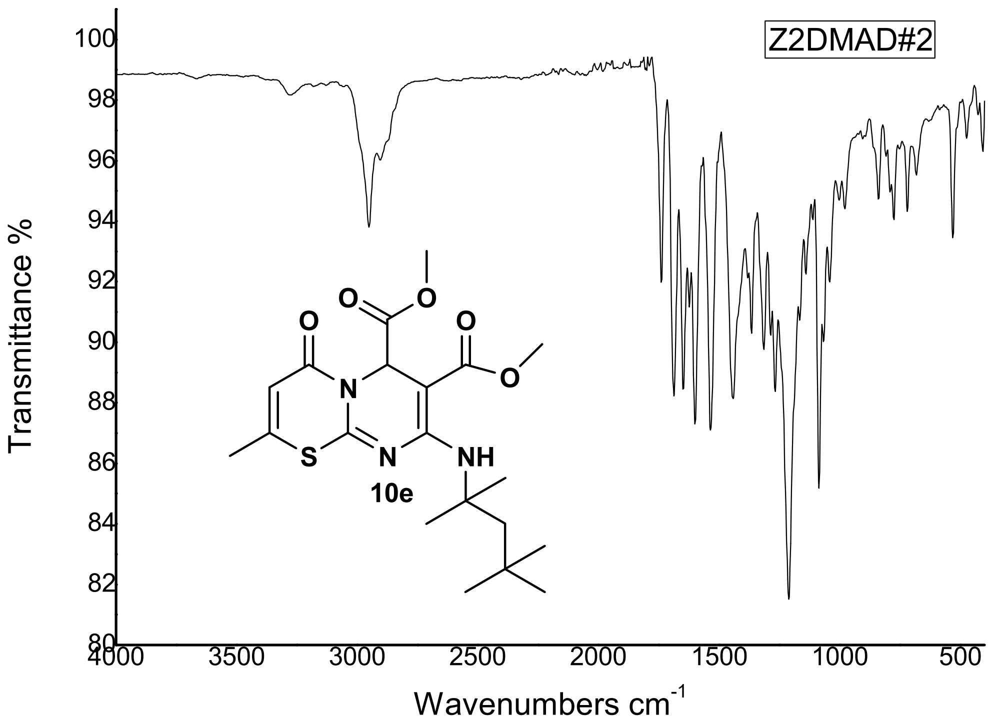
Z2DMAD#2
Transmittance %
Wavenumbers cm-1
10e

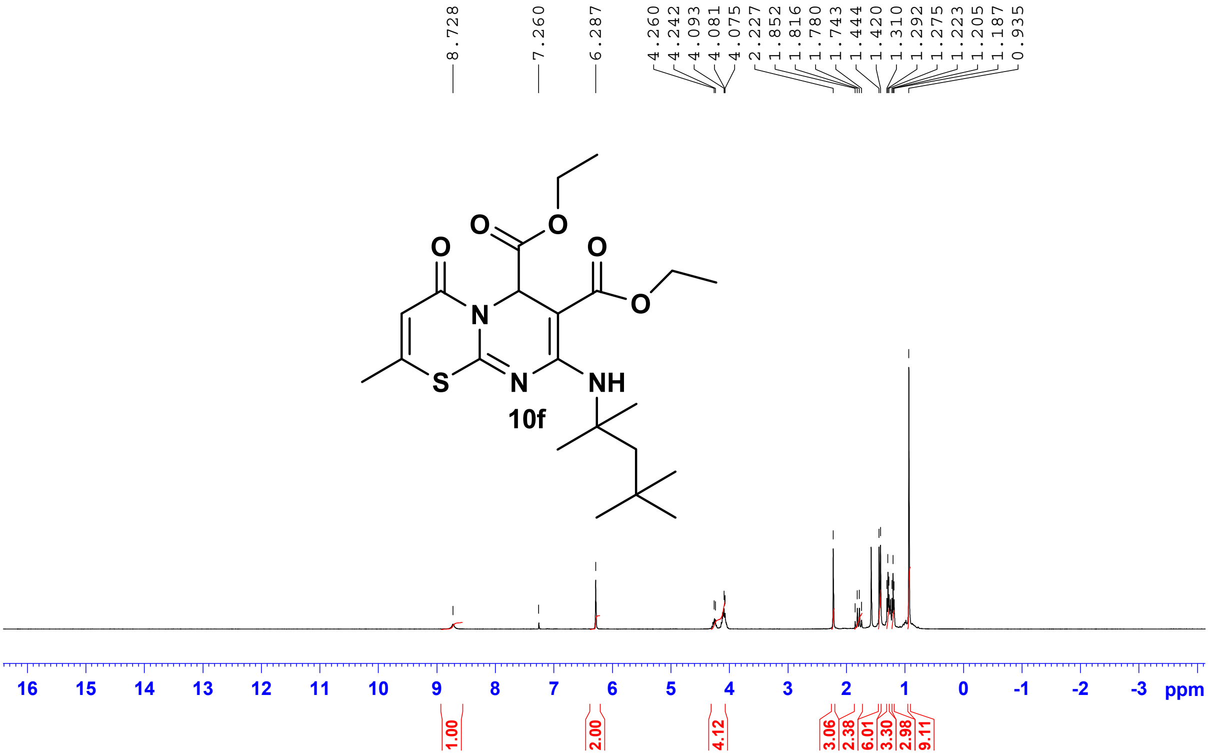
8.728
7.260
6.287
4.260
4.242
4.093
4.081
4.075
2.227
1.852
1.816
1.780
1.743
1.444
1.420
1.310
1.292
1.275
1.223
1.205
1.187
0.935
10f
ppm
1.00
2.00
4.12
3.06
2.38
6.01
3.30
2.98
9.11

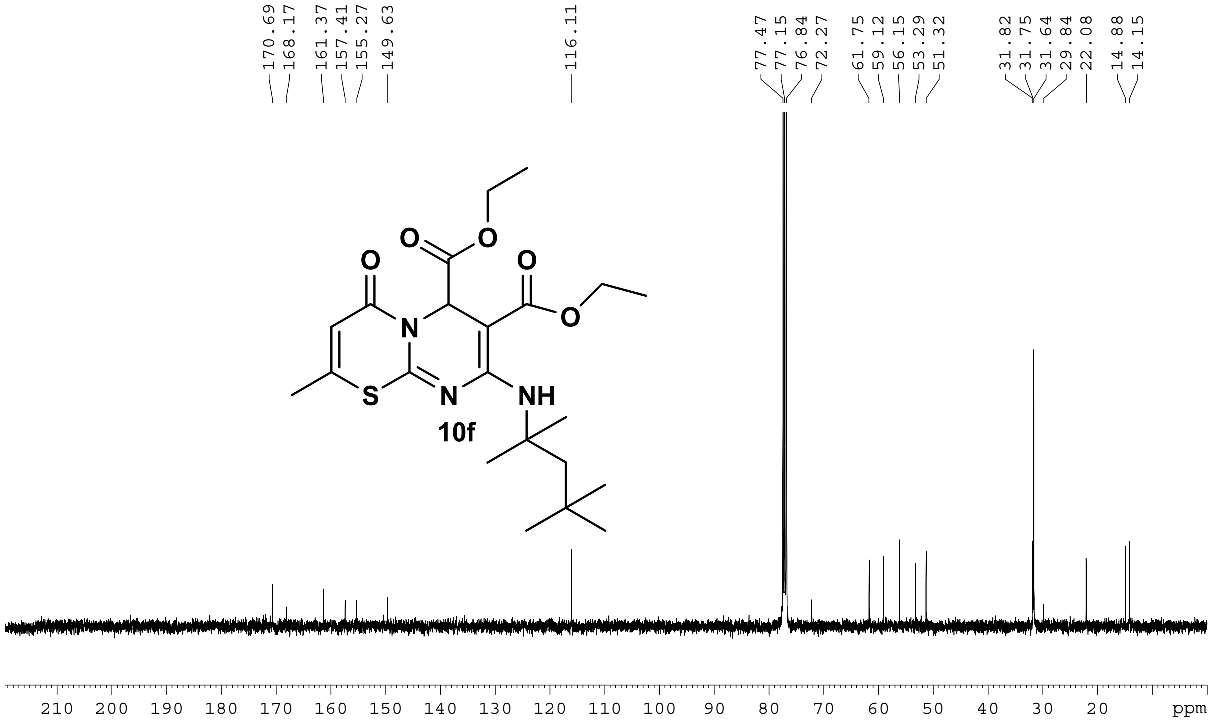
170.69
168.17
161.37
157.41
155.27
149.63
116.11
77.47
77.15
76.84
72.27
61.75
59.12
56.15
53.29
51.32
31.82
31.75
31.64
29.84
22.08
14.88
14.15
O
O
O
O
O
N
S
N
NH
10f
210 200 190 180 170 160 150 140 130 120 110 100 90 80 70 60 50 40 30 20 ppm

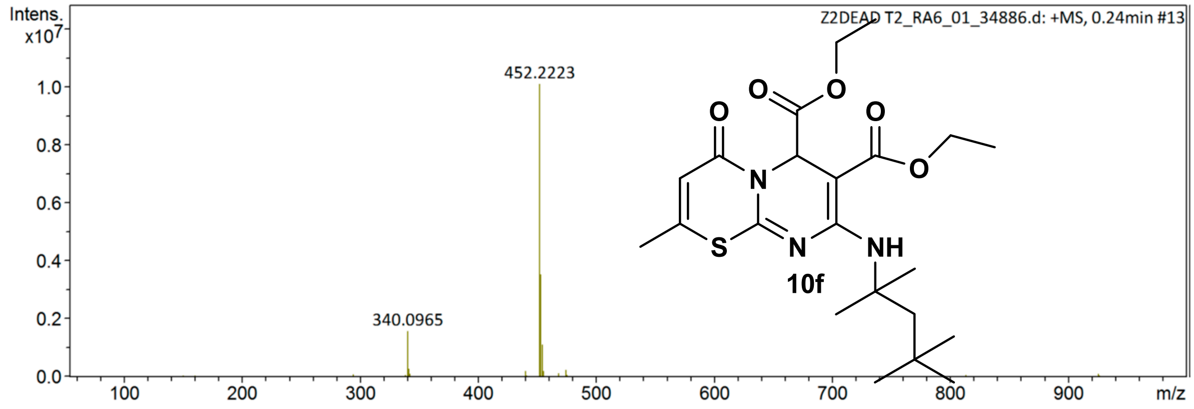
Intens.
x10^7
Z2DEAD T2_RA6_01_34886.d: +MS, 0.24min #13
452.2223
340.0965
1.0
0.8
0.6
0.4
0.2
0.0
100 200 300 400 500 600 700 800 900 m/z
O
O
O
O
O
N
S
N
NH
10f

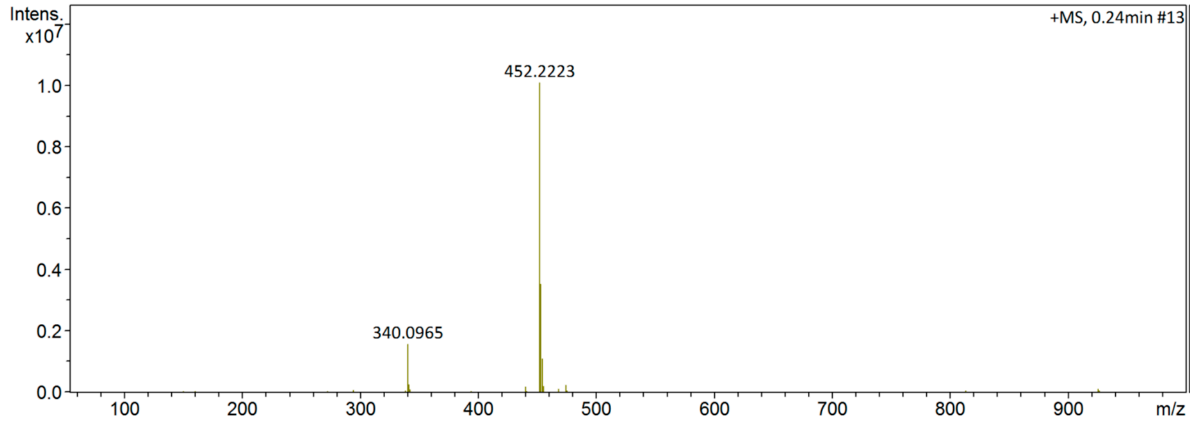
Intens.
x10^7
+MS, 0.24min #13
452.2223
340.0965
1.0
0.8
0.6
0.4
0.2
0.0
100 200 300 400 500 600 700 800 900 m/z

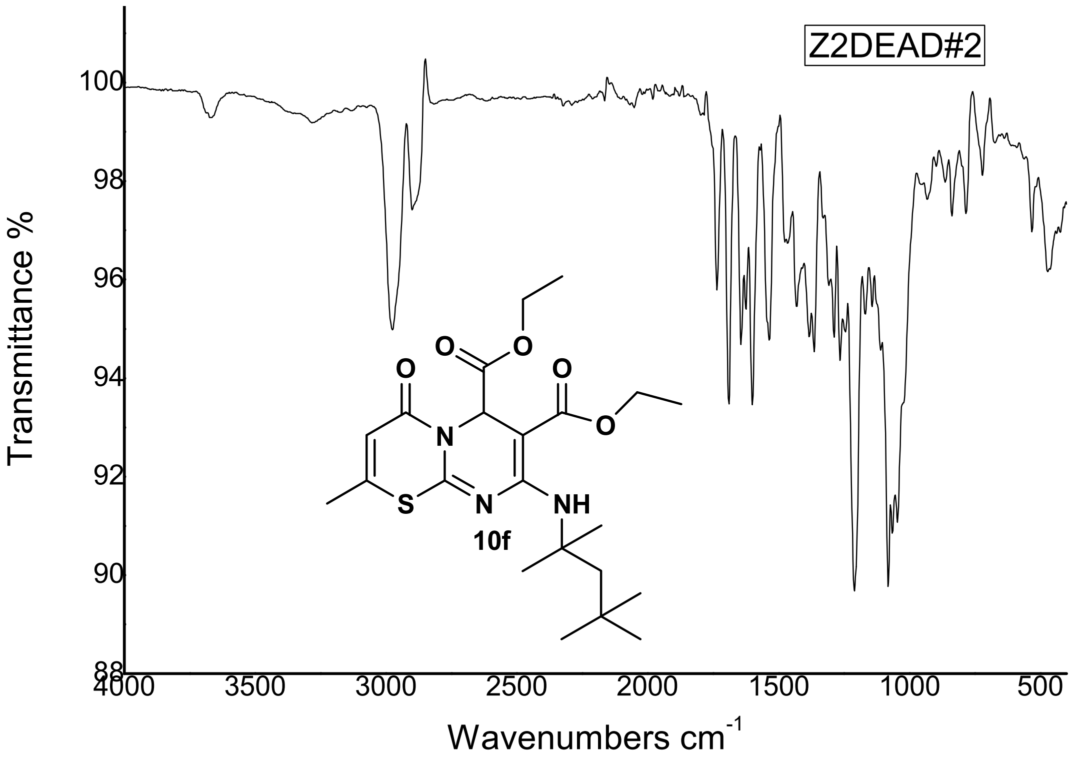
Z2DEAD#2
Transmitance %
100
98
96
94
92
90
88
4000
3500
3000
2500
2000
1500
1000
500
Wavenumbers cm-1
O
O
O
O
O
N
S
N
NH
10f

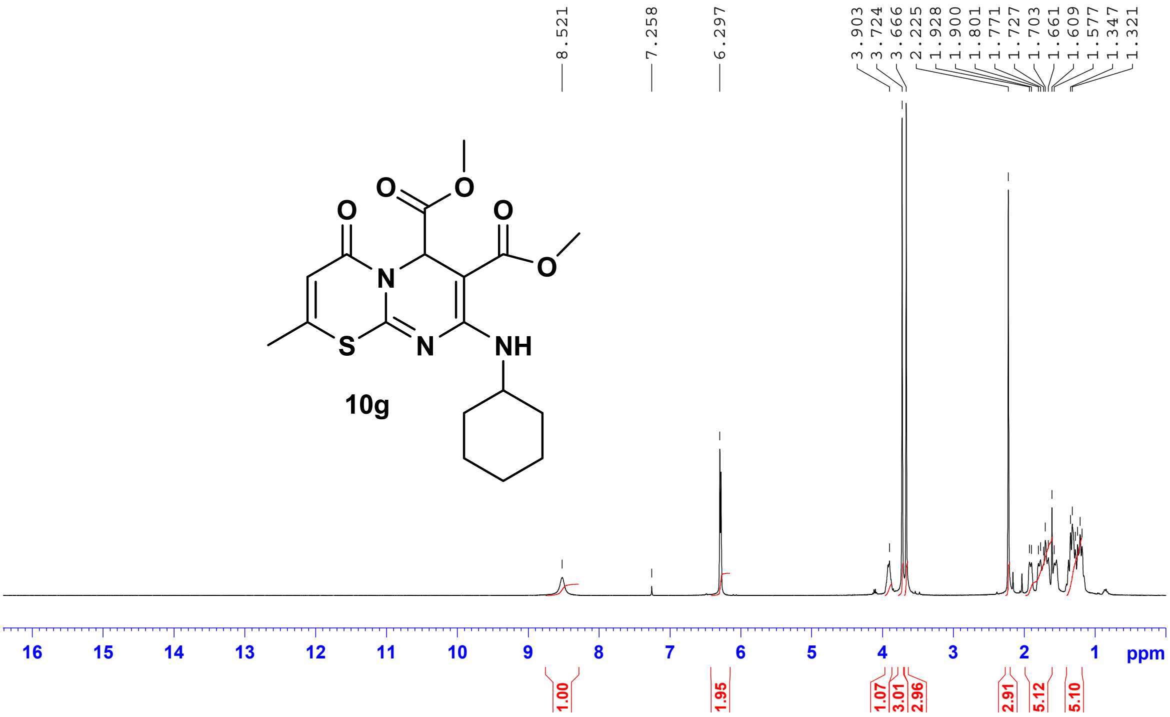
8.521
7.258
6.297
3.903
3.724
3.666
2.225
1.928
1.900
1.801
1.771
1.727
1.703
1.661
1.609
1.577
1.347
1.321
O
O
O
O
O
N
S
N
NH
10g
16
15
14
13
12
11
10
9
8
7
6
5
4
3
2
1
ppm
1.00
1.95
1.07
3.01
2.96
2.91
5.12
5.10

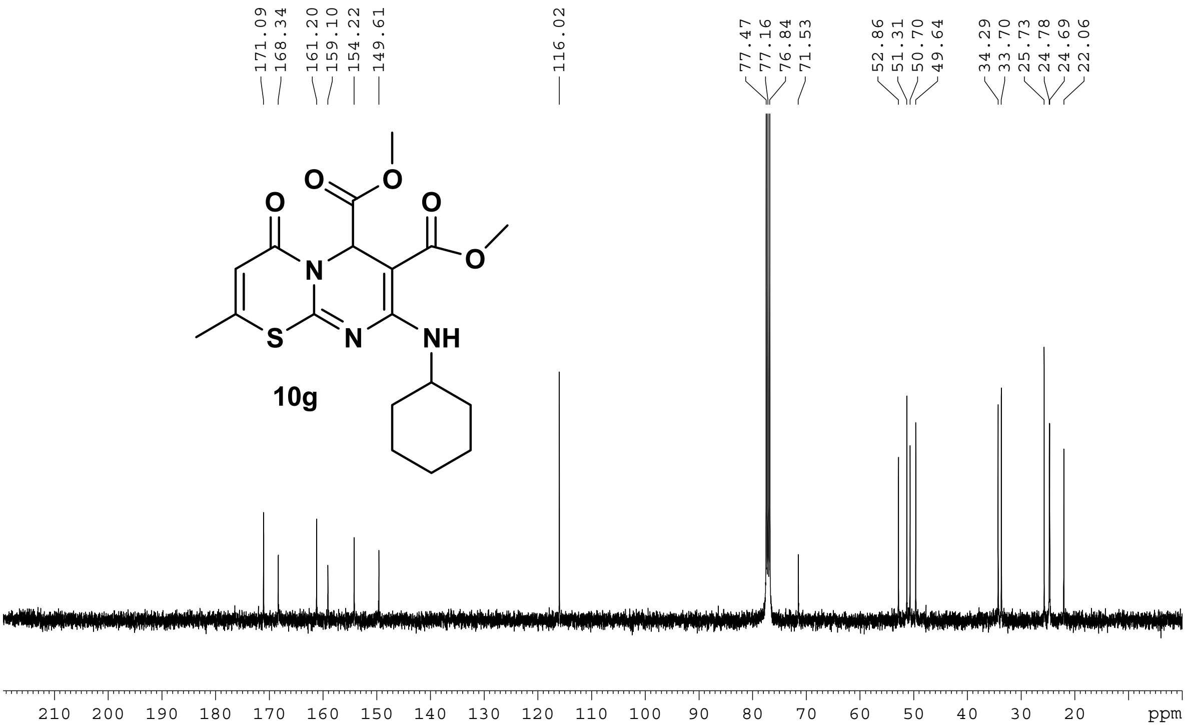
171.09
168.34
161.20
159.10
154.22
149.61
116.02
77.47
77.16
76.84
71.53
52.86
51.31
50.70
49.64
34.29
33.70
25.73
24.78
24.69
22.06
O
O
O
O
O
N
S
N
NH
10g
210 200 190 180 170 160 150 140 130 120 110 100 90 80 70 60 50 40 30 20 ppm

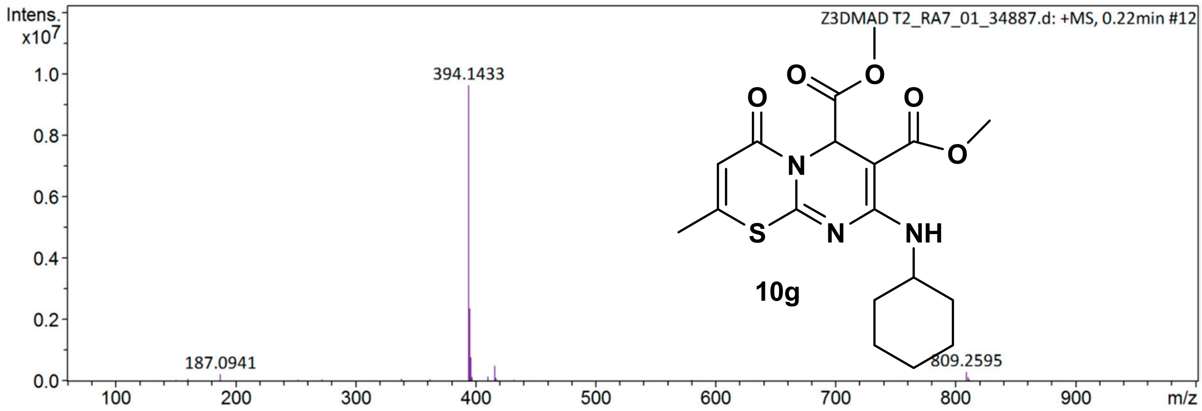
Intens.
x10[7]
Z3DMAD T2_RA7_01_34887.d: +MS, 0.22min #12
1.0
0.8
0.6
0.4
0.2
0.0
394.1433
187.0941
809.2595
O
O
O
O
O
N
S
N
NH
10g
100 200 300 400 500 600 700 800 900 m/z

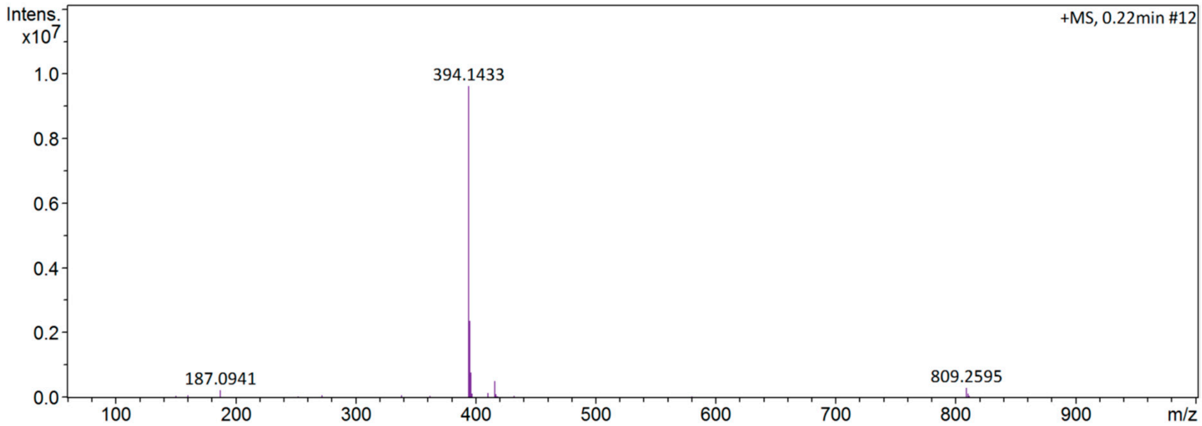
Intens.
x10[7]
+MS, 0.22min #12
1.0
0.8
0.6
0.4
0.2
0.0
394.1433
187.0941
809.2595
100 200 300 400 500 600 700 800 900 m/z

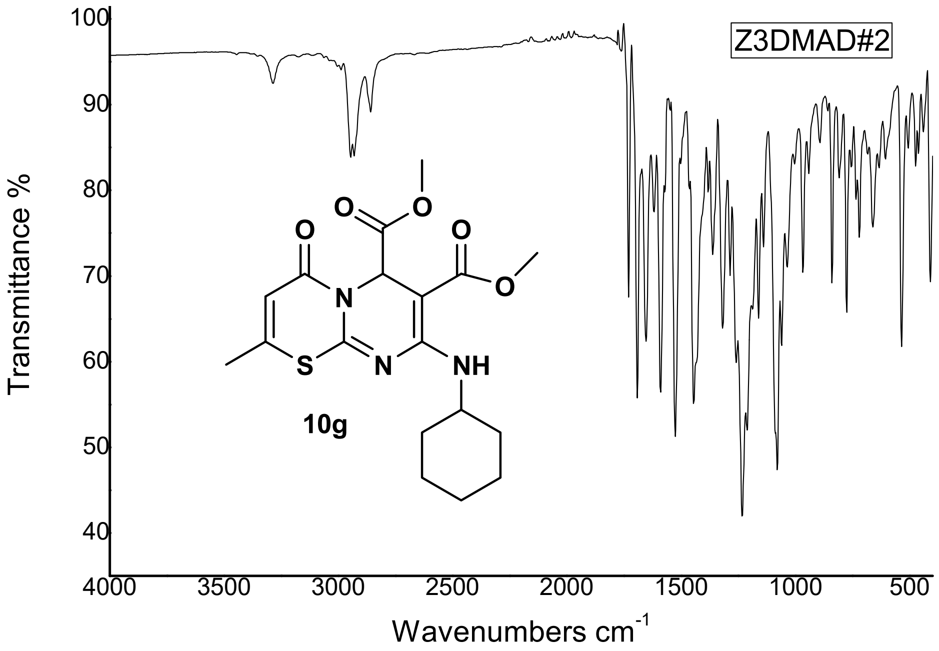
Z3DMAD#2
Transmittance %
Wavenumbers cm-1
O
O
O
O
O
N
S
N
NH
10g
100
90
80
70
60
50
40
4000
3500
3000
2500
2000
1500
1000
500

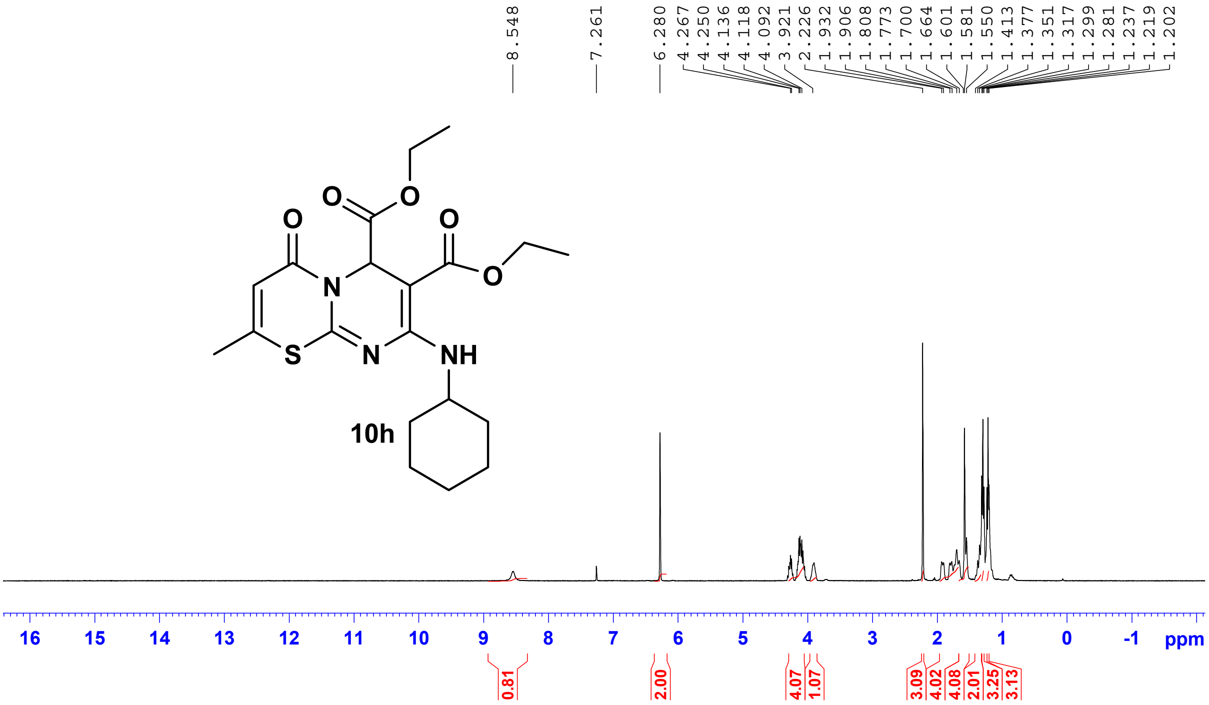
8.548
7.261
6.280
4.267
4.250
4.136
4.118
4.092
3.921
2.226
1.932
1.906
1.808
1.773
1.700
1.664
1.601
1.581
1.550
1.413
1.377
1.351
1.317
1.299
1.281
1.237
1.219
1.202
O
O
O
O
O
N
S
N
NH
10h
16
15
14
13
12
11
10
9
8
7
6
5
4
3
2
1
0
-1
ppm
0.81
2.00
4.07
1.07
3.09
4.02
4.08
2.01
3.25
3.13

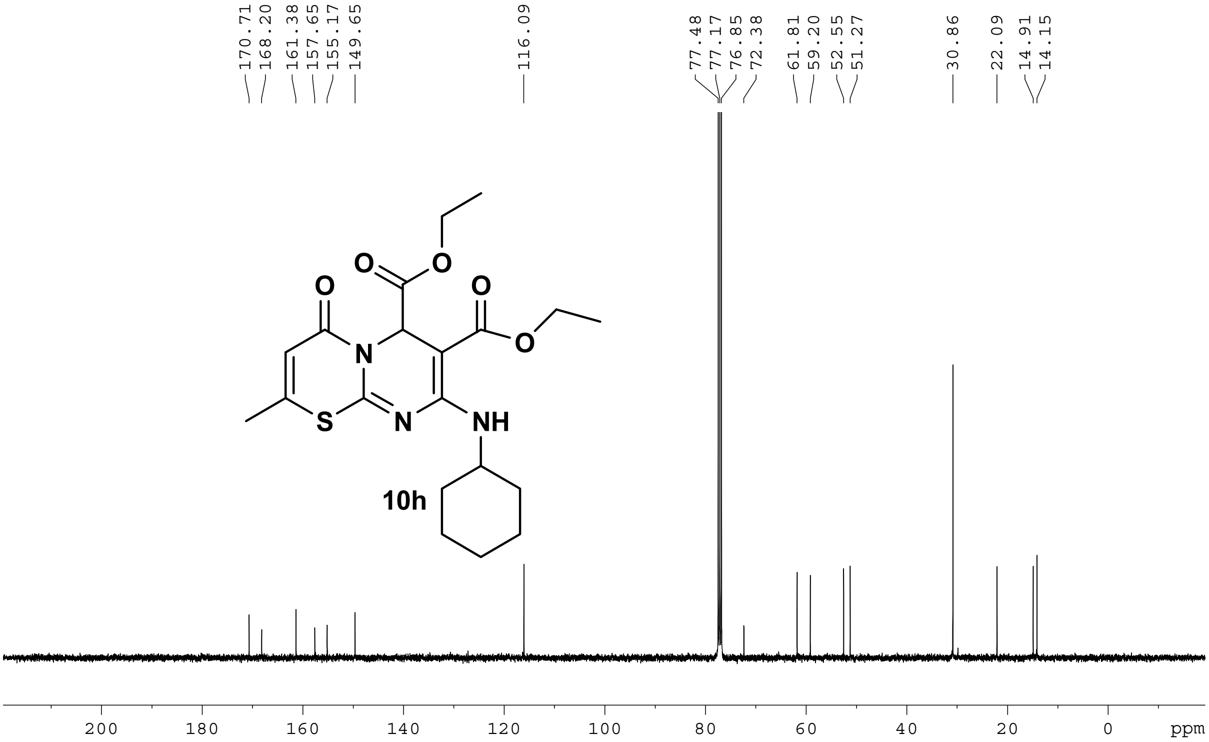
170.71
168.20
161.38
157.65
155.17
149.65
116.09
77.48
77.17
76.85
72.38
61.81
59.20
52.55
51.27
30.86
22.09
14.91
14.15
10h
200
180
160
140
120
100
80
60
40
20
0
ppm

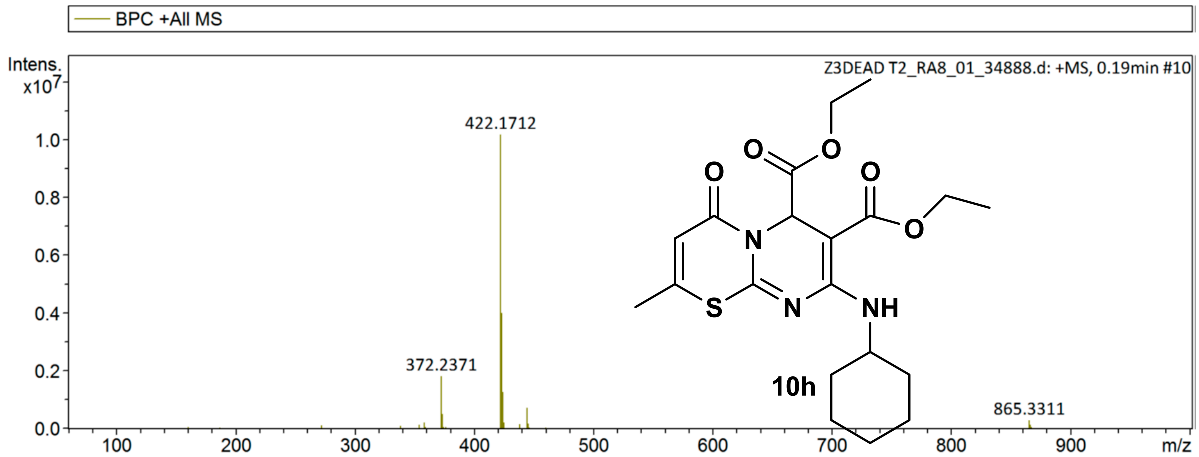
BPC +All MS
Intens.
x10^7
Z3DEAD T2_RA8_01_34888.d: +MS, 0.19min #10
422.1712
372.2371
865.3311
10h
1.0
0.8
0.6
0.4
0.2
0.0
100
200
300
400
500
600
700
800
900
m/z

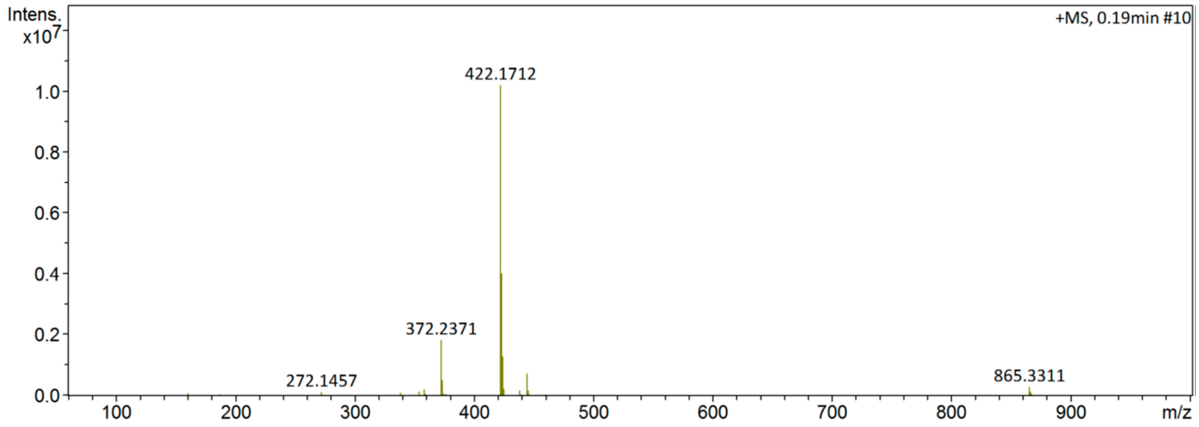
Intens.
x10^7
+MS, 0.19min #10
422.1712
372.2371
272.1457
865.3311
1.0
0.8
0.6
0.4
0.2
0.0
100
200
300
400
500
600
700
800
900
m/z

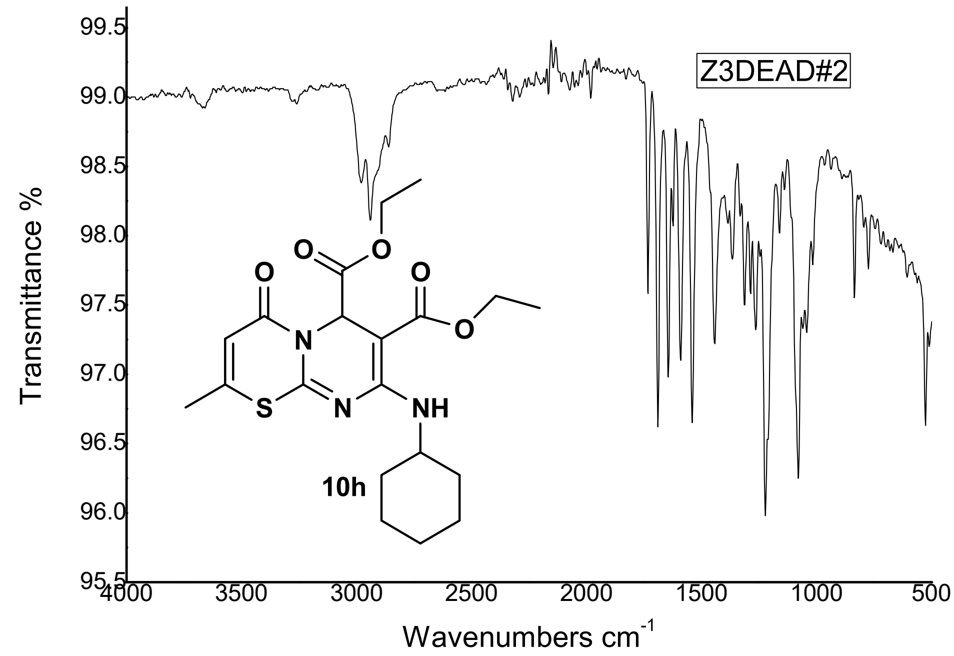
Z3DEAD#2
Transmittance %
99.5
99.0
98.5
98.0
97.5
97.0
96.5
96.0
95.5
4000
3500
3000
2500
2000
1500
1000
500
Wavenumbers cm-1
10h

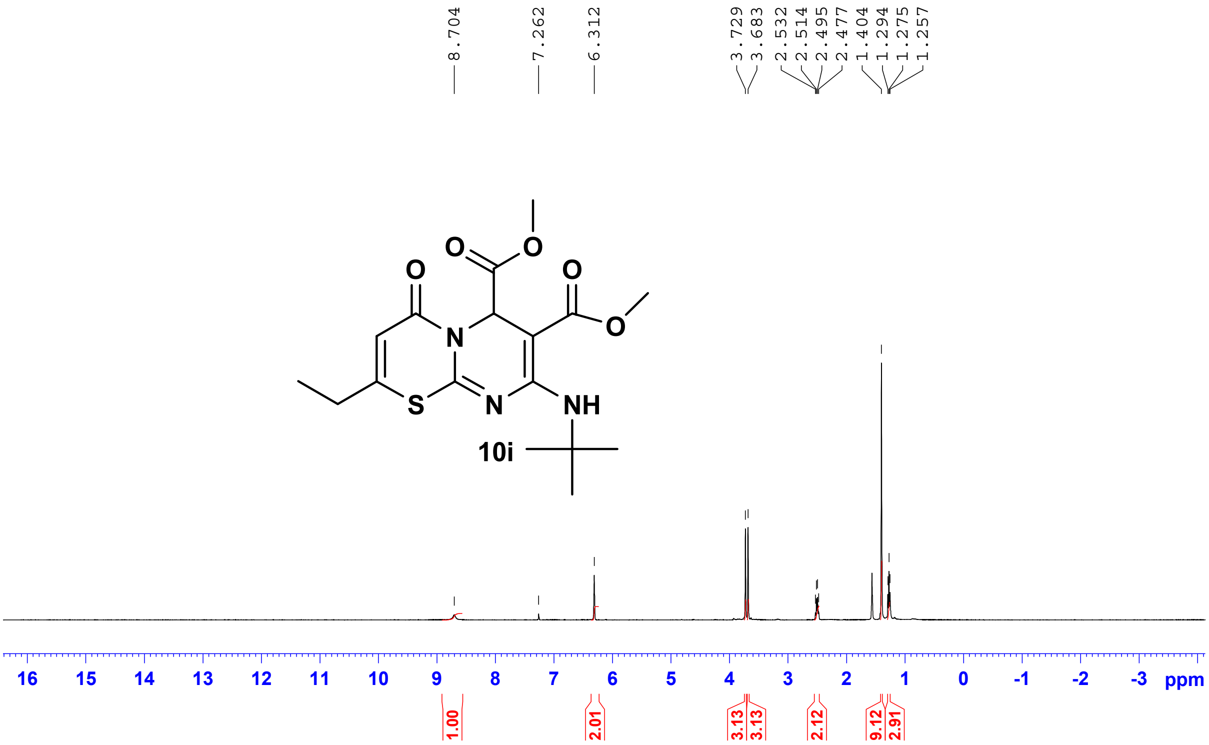
8.704
7.262
6.312
3.729
3.683
2.532
2.514
2.495
2.477
1.404
1.294
1.275
1.257
10i
16 15 14 13 12 11 10 9 8 7 6 5 4 3 2 1 0 -1 -2 -3 ppm
1.00
2.01
3.13
3.13
2.12
9.12
2.91

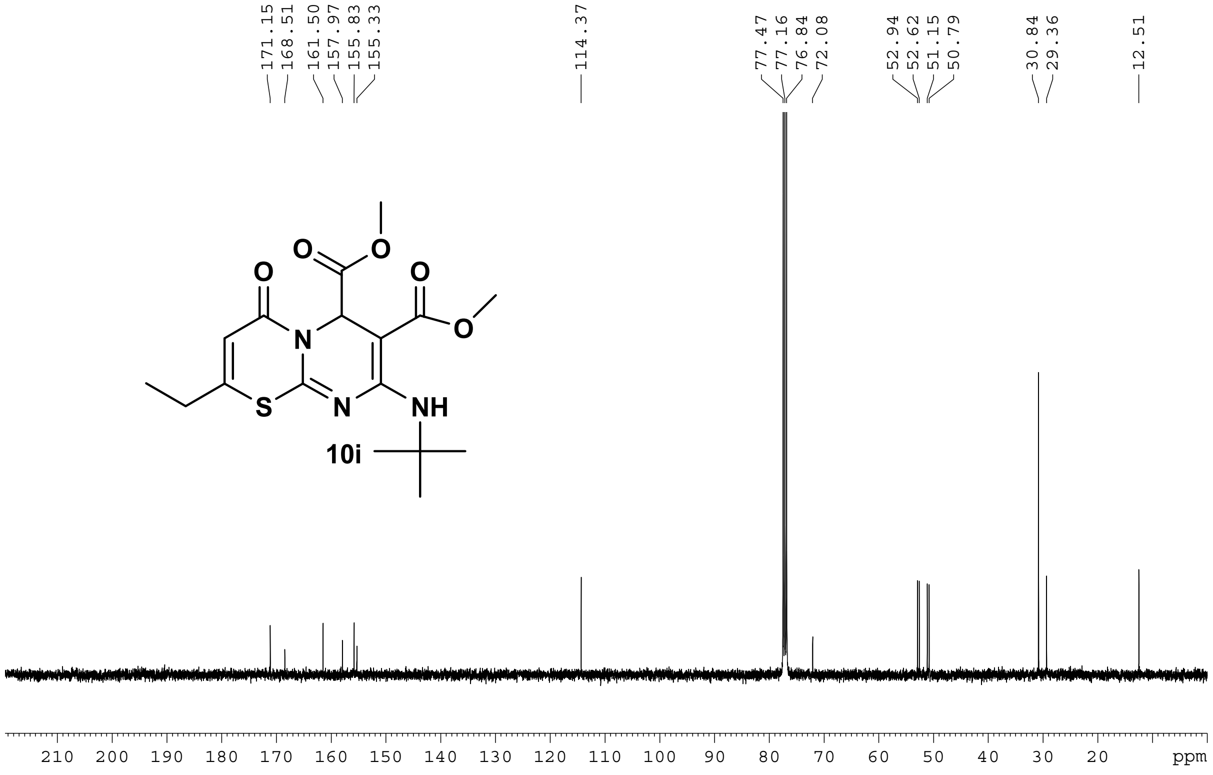
171.15
168.51
161.50
157.97
155.83
155.33
114.37
77.47
77.16
76.84
72.08
52.94
52.62
51.15
50.79
30.84
29.36
12.51
O
O
O
O
O
N
S
N
NH
10i
210 200 190 180 170 160 150 140 130 120 110 100 90 80 70 60 50 40 30 20
ppm

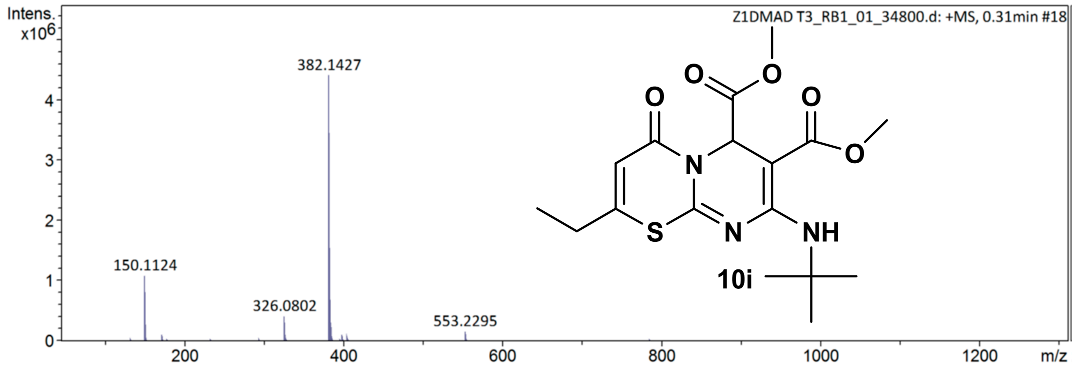
Intens.
x10^6
Z1DMAD T3_RB1_01_34800.d: +MS, 0.31min #18
382.1427
150.1124
326.0802
553.2295
10i
0 1 2 3 4
200 400 600 800 1000 1200
m/z

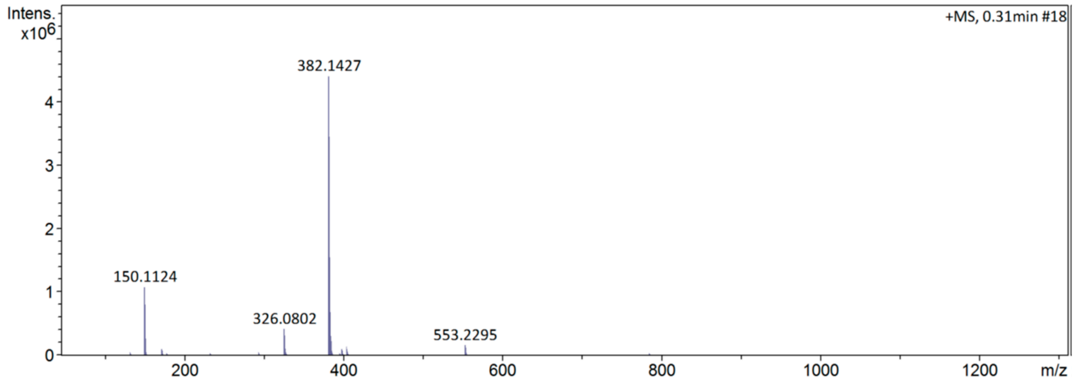
Intens.
x10^6
+MS, 0.31min #18
382.1427
150.1124
326.0802
553.2295
0 1 2 3 4
200 400 600 800 1000 1200
m/z

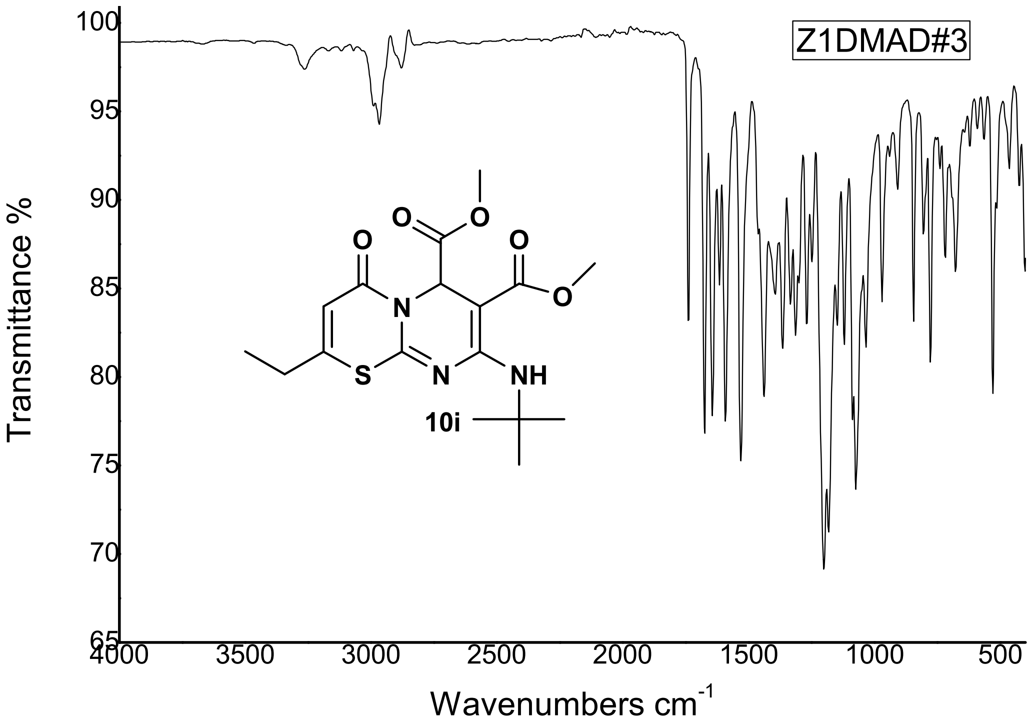
Z1DMAD#3
Transmittance %
Wavenumbers cm-1
10i

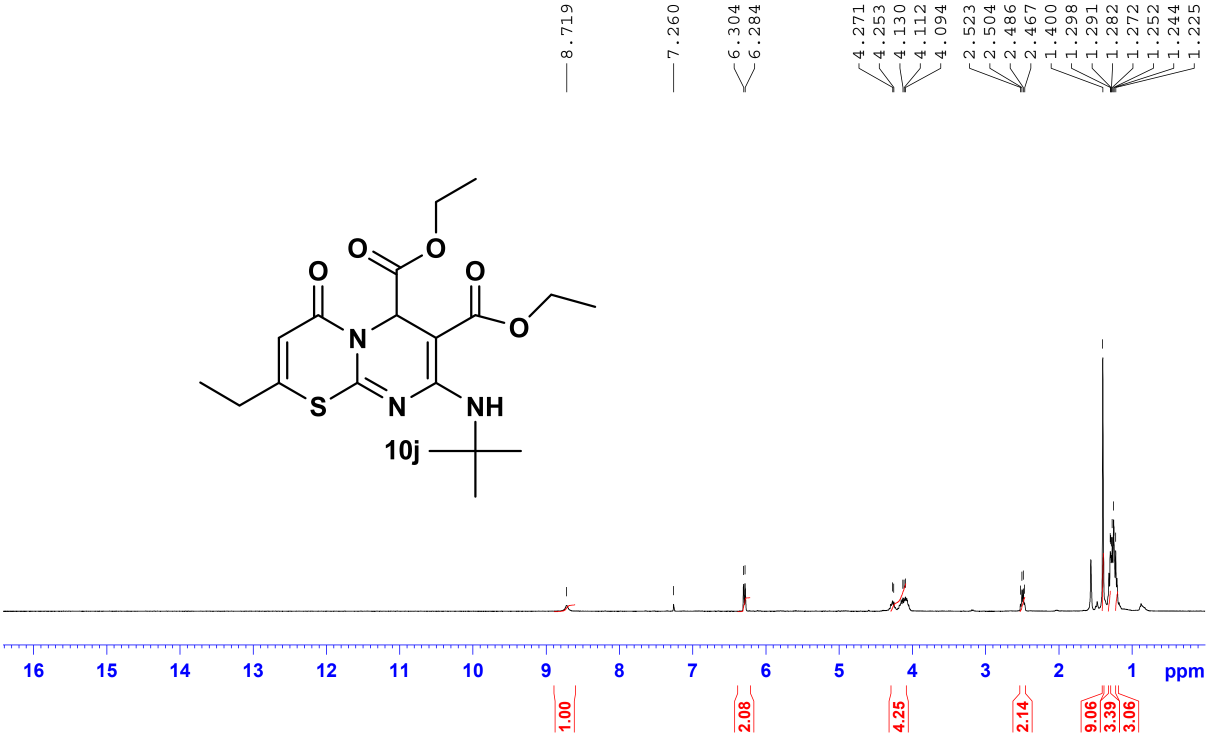
10j
ppm

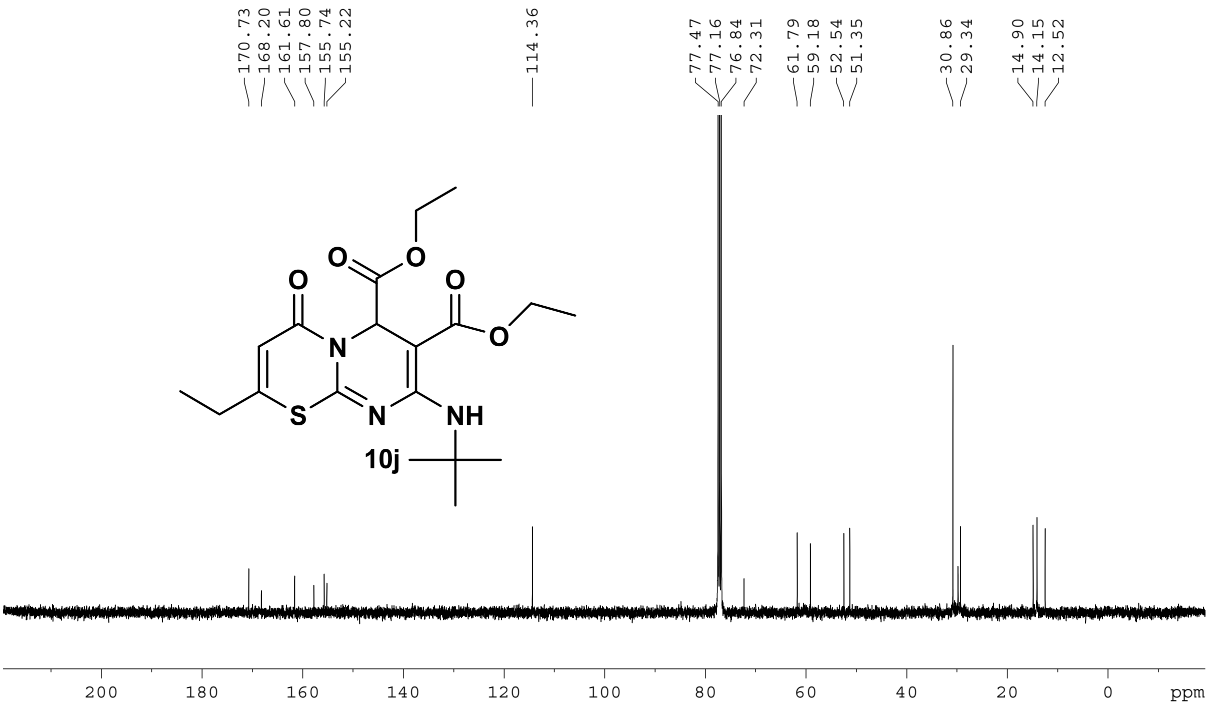

170.73
168.20
161.61
157.80
155.74
155.22
114.36
77.47
77.16
76.84
72.31
61.79
59.18
52.54
51.35
30.86
29.34
14.90
14.15
12.52
10j
200
180
160
140
120
100
80
60
40
20
0
ppm

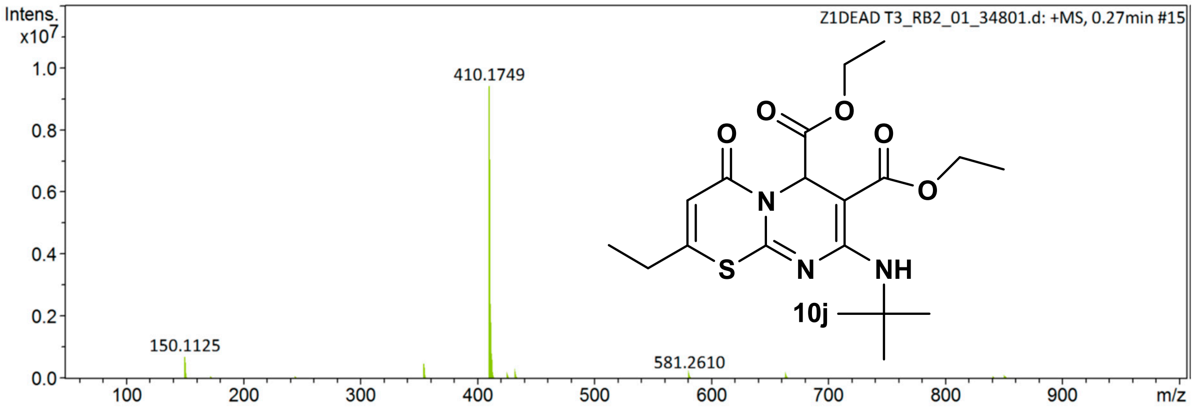

Intens.
x10^7
Z1DEAD T3_RB2_01_34801.d: +MS, 0.27min #15
410.1749
150.1125
581.2610
10j
m/z

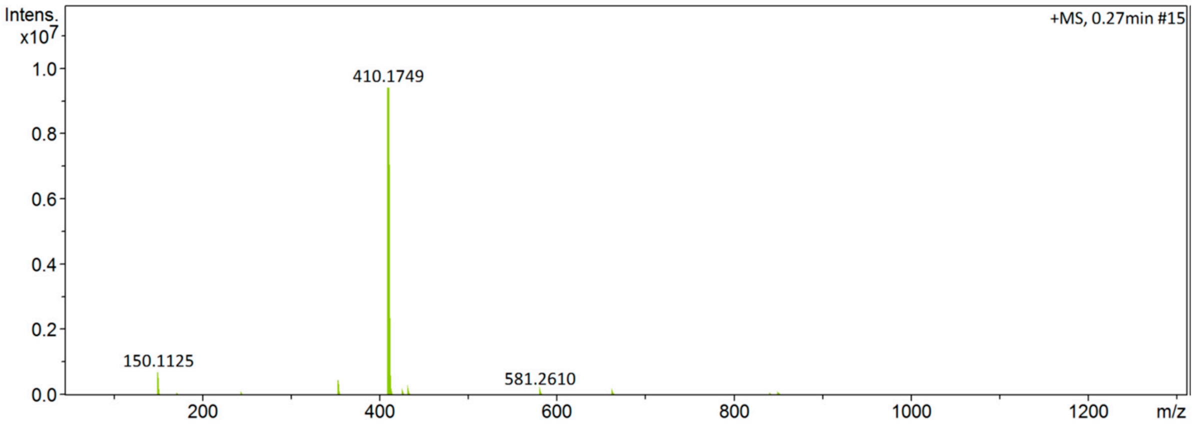

Intens.
x10^7
+MS, 0.27min #15
410.1749
150.1125
581.2610
m/z

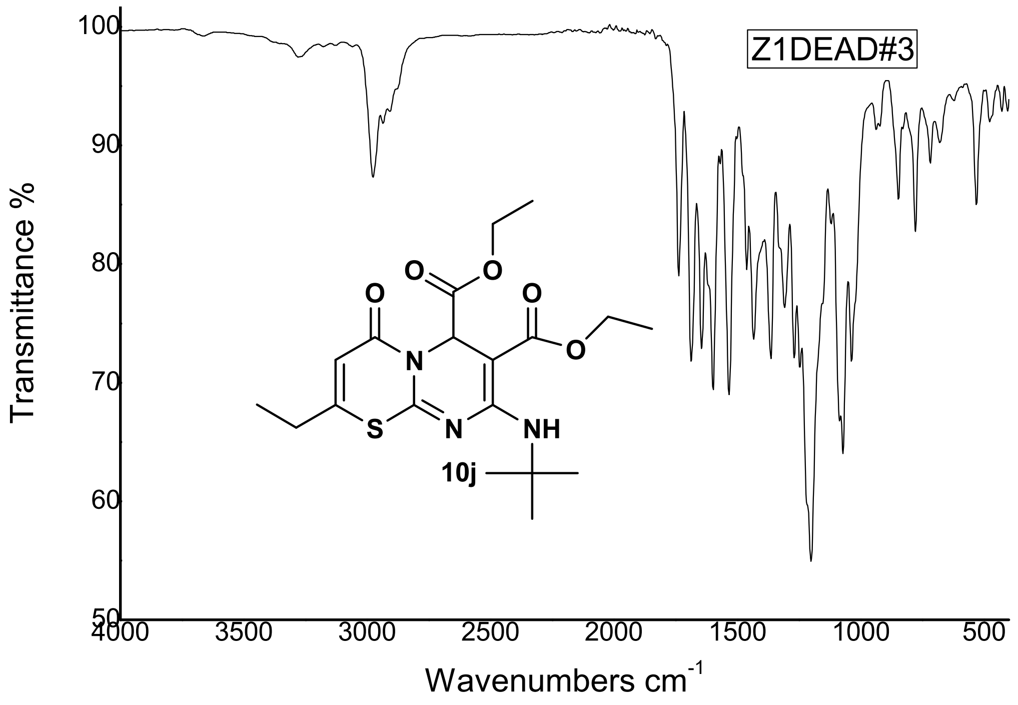
Z1DEAD#3
Transmittance %
Wavenumbers cm-1
100
90
80
70
60
50
4000
3500
3000
2500
2000
1500
1000
500
O
O
O
O
O
N
S
N
NH
10j

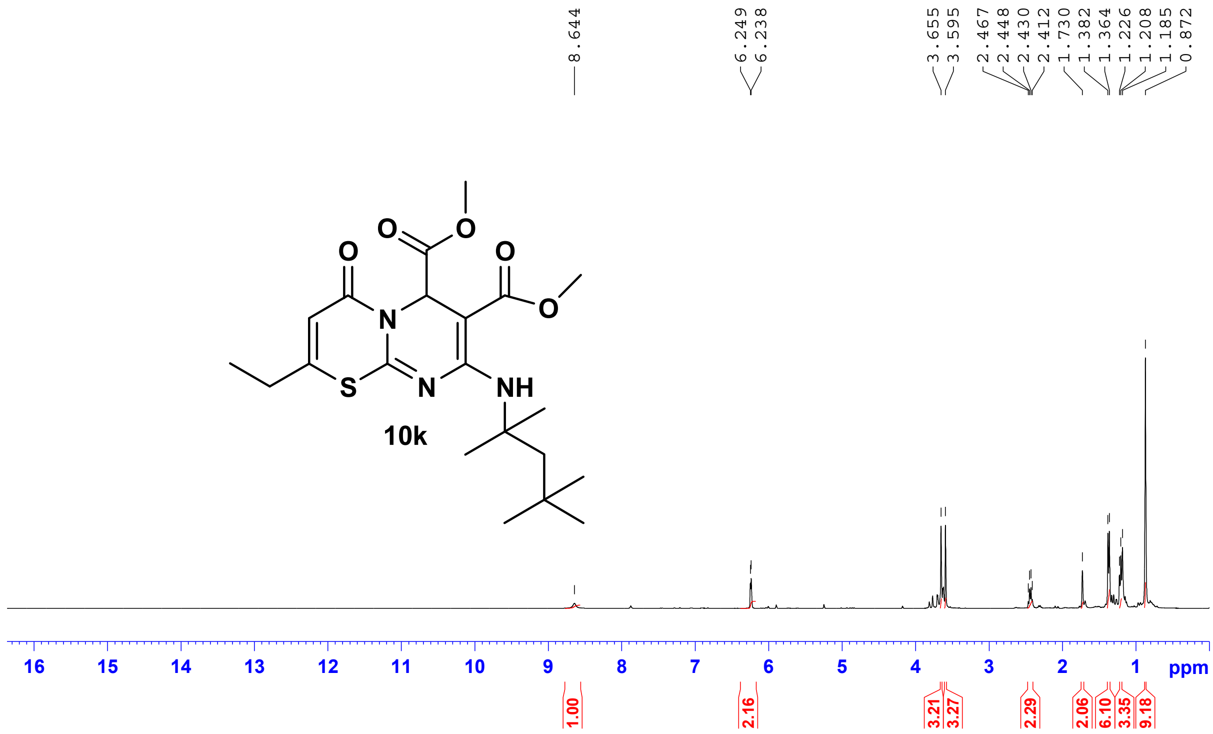
8.644
6.249
6.238
3.655
3.595
2.467
2.448
2.430
2.412
1.730
1.382
1.364
1.226
1.208
1.185
0.872
O
O
O
O
O
N
S
N
NH
10k
16
15
14
13
12
11
10
9
8
7
6
5
4
3
2
1
ppm
1.00
2.16
3.21
3.27
2.29
2.06
6.10
3.35
9.18

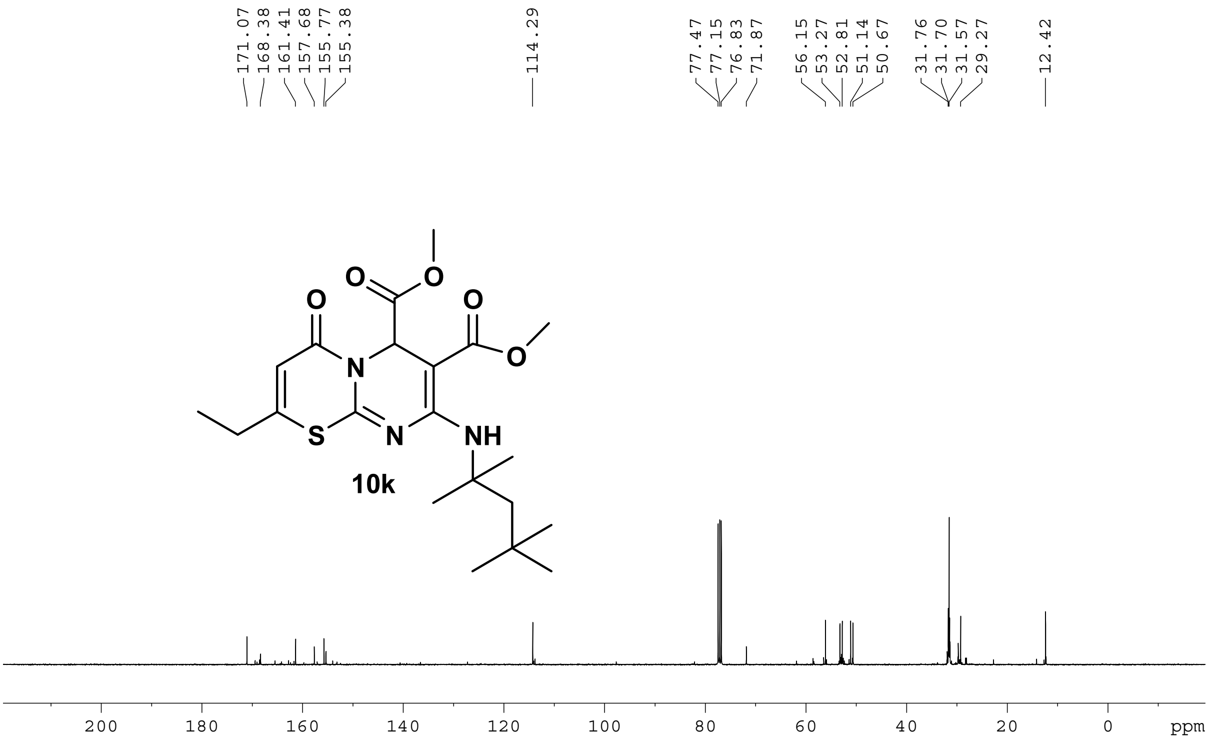
171.07
168.38
161.41
157.68
155.77
155.38
114.29
77.47
77.15
76.83
71.87
56.15
53.27
52.81
51.14
50.67
31.76
31.70
31.57
29.27
12.42
O
O
O
O
O
N
S
N
NH
10k
200
180
160
140
120
100
80
60
40
20
0
ppm

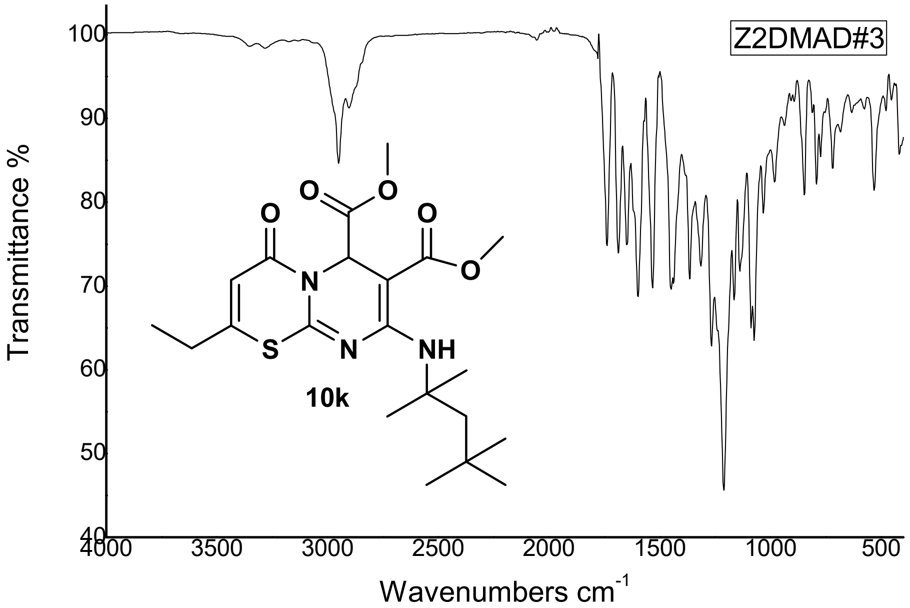
Z2DMAD#3
Transmittance %
100
90
80
70
60
50
40
4000
3500
3000
2500
2000
1500
1000
500
Wavenumbers cm-1
O
O
O
O
O
N
S
N
NH
10k

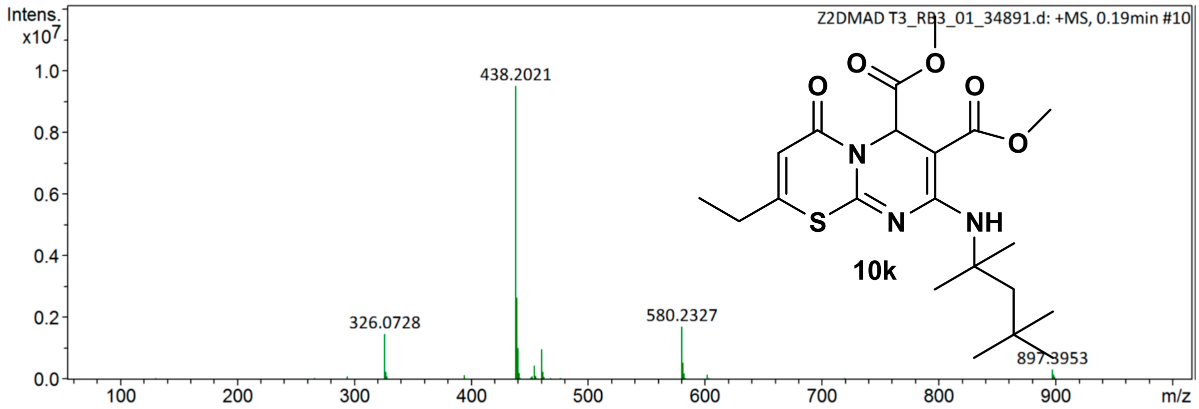
Intens.
x10^7
Z2DMAD T3_RB3_01_34891.d: +MS, 0.19min #10
438.2021
326.0728
580.2327
897.3953
10k
O
N
S
N
NH
m/z
1.0
0.8
0.6
0.4
0.2
0.0
100
200
300
400
500
600
700
800
900

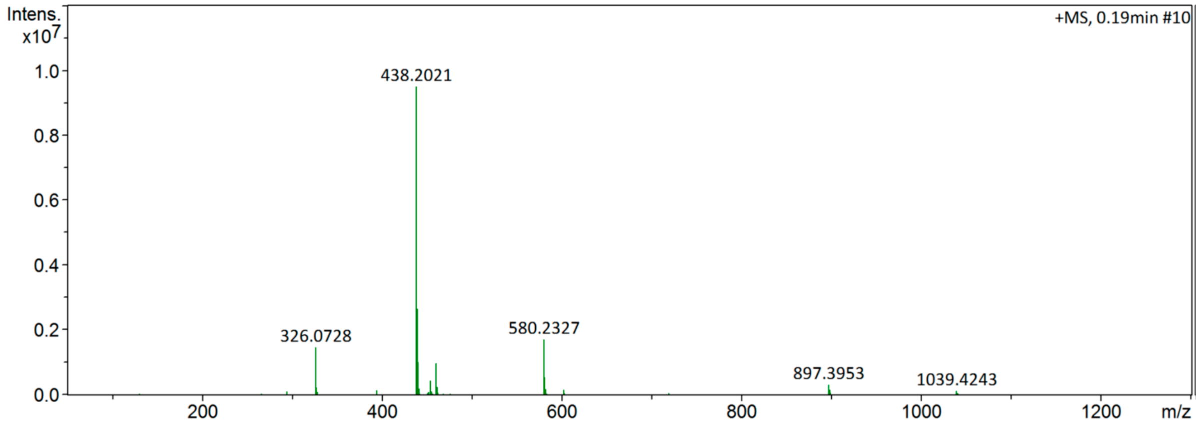
Intens.
x10^7
+MS, 0.19min #10
438.2021
326.0728
580.2327
897.3953
1039.4243
m/z
1.0
0.8
0.6
0.4
0.2
0.0
200
400
600
800
1000
1200

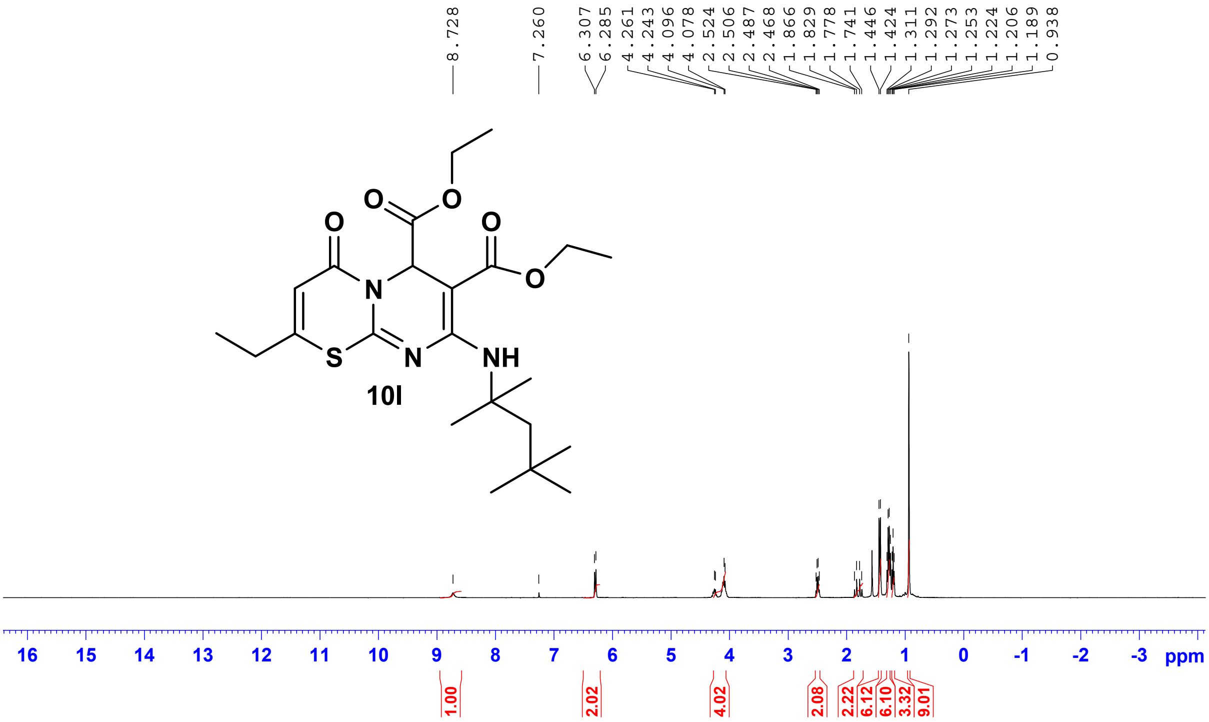
8.728
7.260
6.307
6.285
4.261
4.243
4.096
4.078
2.524
2.506
2.487
2.468
1.866
1.829
1.778
1.741
1.446
1.424
1.311
1.292
1.273
1.253
1.224
1.206
1.189
0.938
10l
O
N
S
N
NH
ppm
1.00
2.02
4.02
2.08
2.22
6.12
6.10
3.32
9.01

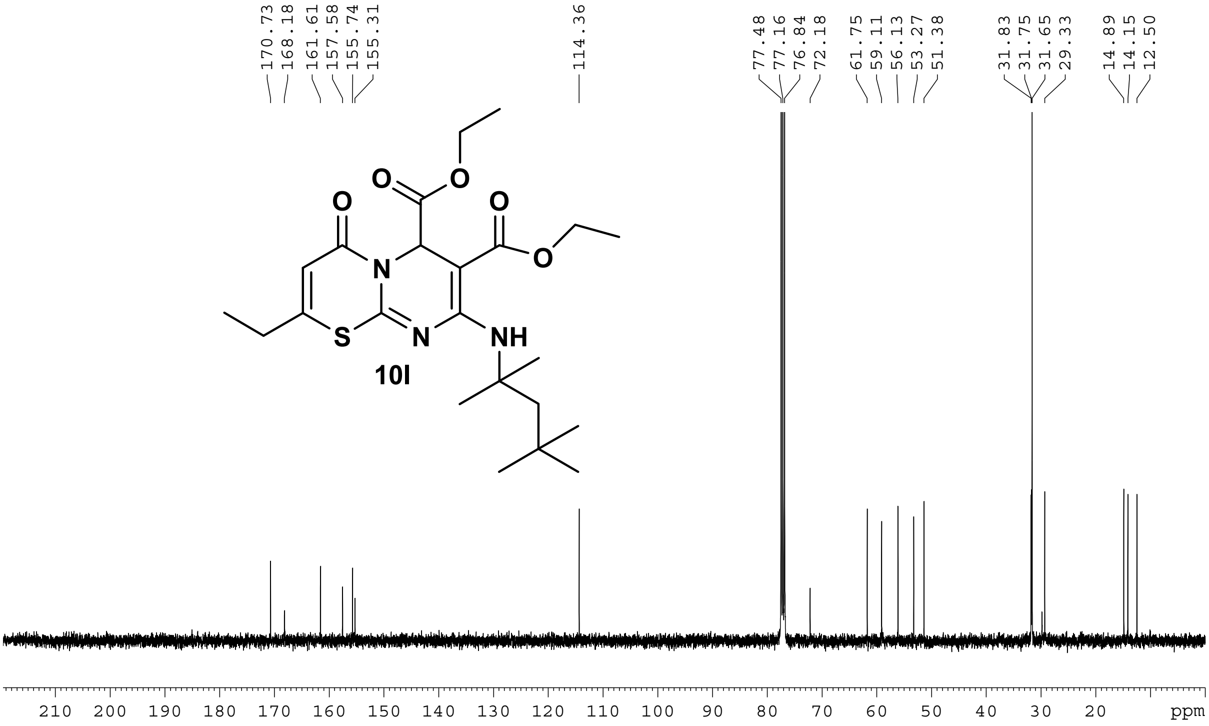
170.73
168.18
161.61
157.58
155.74
155.31
114.36
77.48
77.16
76.84
72.18
61.75
59.11
56.13
53.27
51.38
31.83
31.75
31.65
29.33
14.89
14.15
12.50
O
O
O
O
O
N
S
N
NH
10l
210 200 190 180 170 160 150 140 130 120 110 100 90 80 70 60 50 40 30 20 ppm

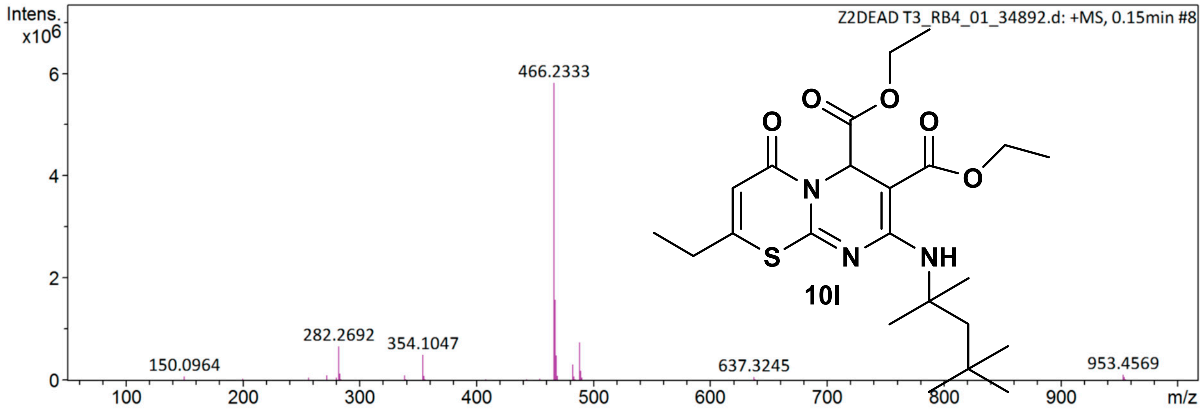
Intens.
x10^6
Z2DEAD T3_RB4_01_34892.d: +MS, 0.15min #8
466.2333
282.2692
354.1047
150.0964
637.3245
953.4569
O
O
O
O
O
N
S
N
NH
10l
m/z

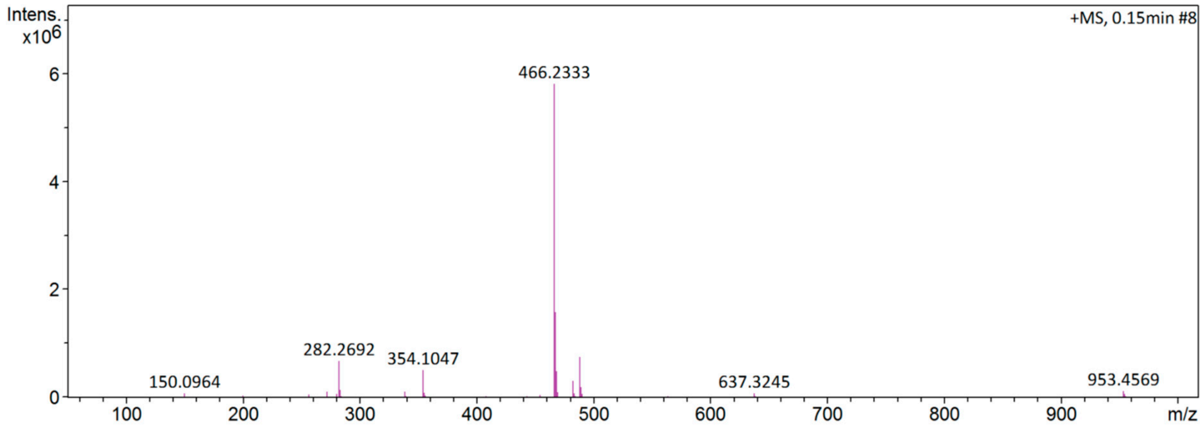
Intens.
x10^6
+MS, 0.15min #8
466.2333
282.2692
354.1047
150.0964
637.3245
953.4569
m/z

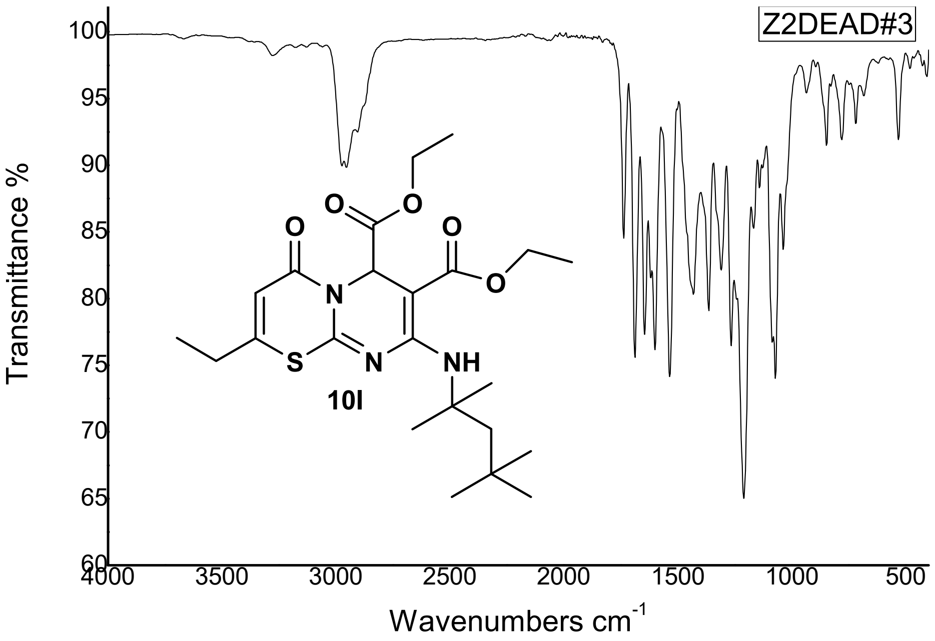
Z2DEAD#3
Transmittance %
Wavenumbers cm-1
100
95
90
85
80
75
70
65
60
4000
3500
3000
2500
2000
1500
1000
500
O
N
S
N
NH
10l

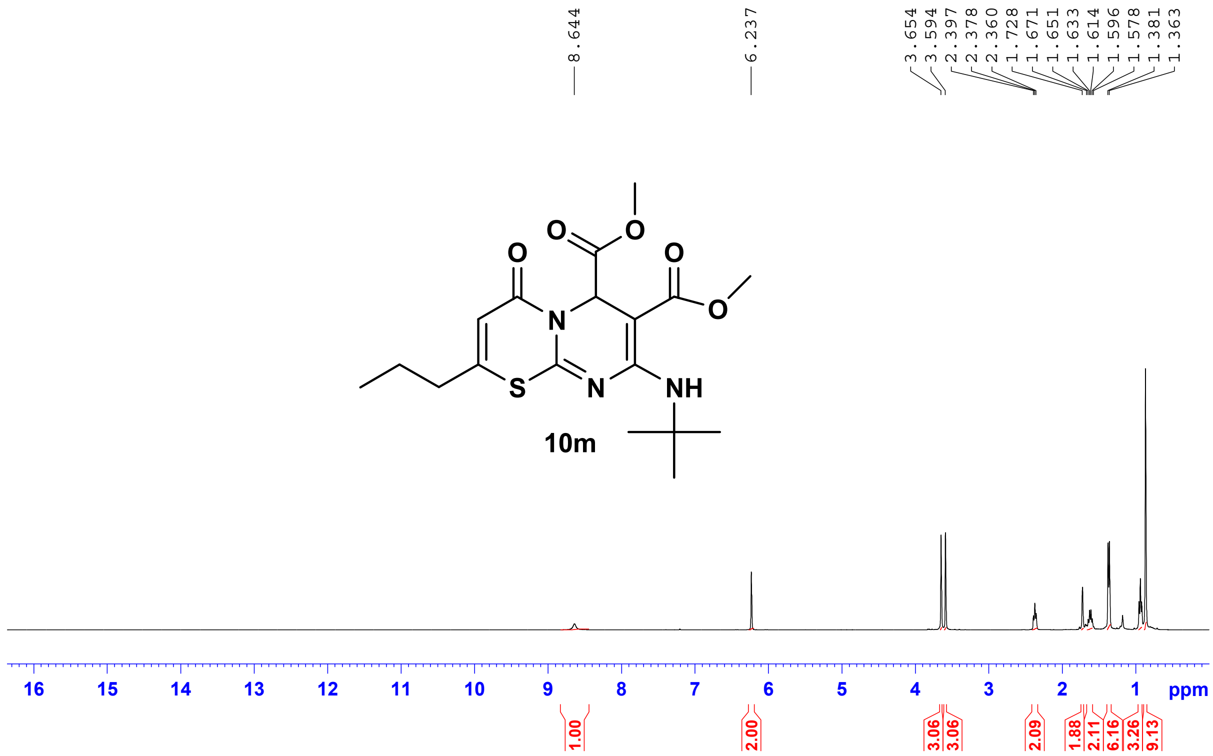
8.644
6.237
3.654
3.594
2.397
2.378
2.360
1.728
1.671
1.651
1.633
1.614
1.596
1.578
1.381
1.363
O
N
S
N
NH
10m
16
15
14
13
12
11
10
9
8
7
6
5
4
3
2
1
ppm
1.00
2.00
3.06
3.06
2.09
1.88
2.11
6.16
3.26
9.13

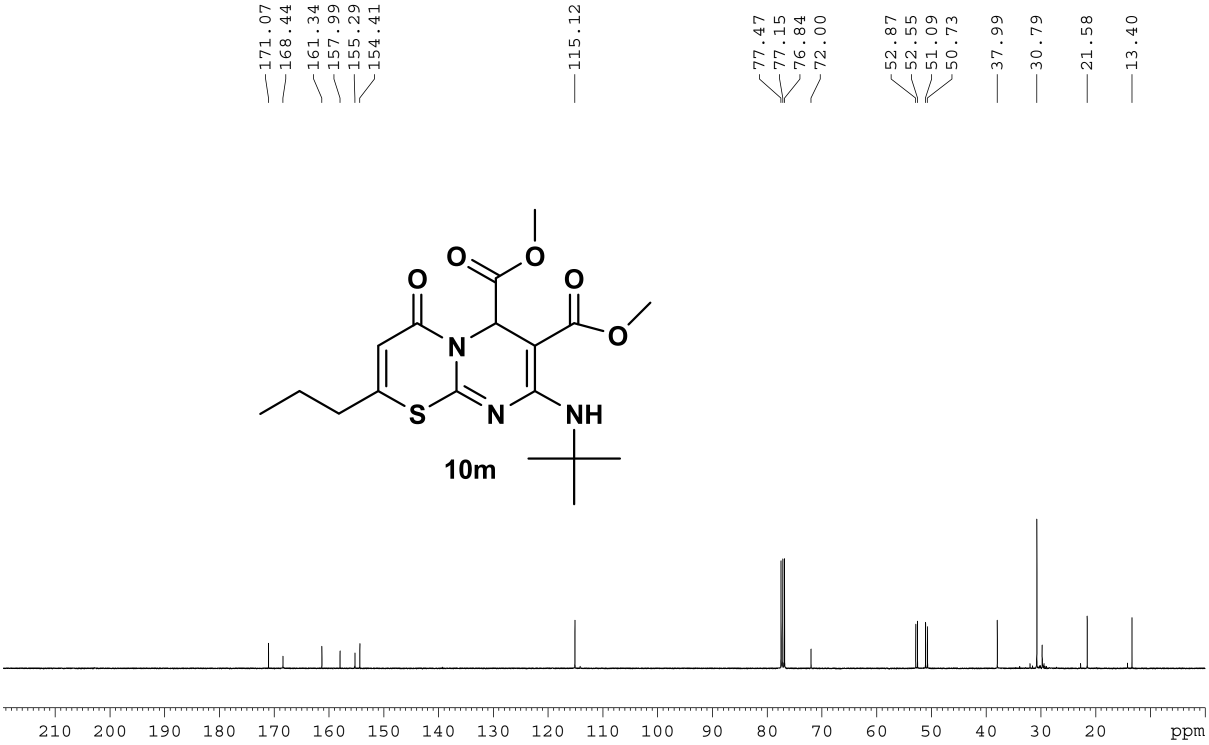
171.07
168.44
161.34
157.99
155.29
154.41
115.12
77.47
77.15
76.84
72.00
52.87
52.55
51.09
50.73
37.99
30.79
21.58
13.40
O
O
O
O
O
N
S
N
NH
10m
210 200 190 180 170 160 150 140 130 120 110 100 90 80 70 60 50 40 30 20 ppm

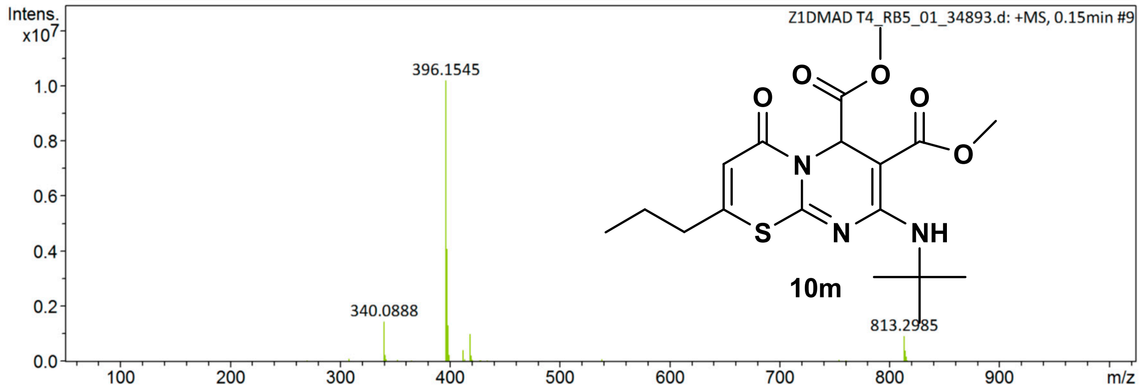
Intens.
x10^7
Z1DMAD T4_RB5_01_34893.d: +MS, 0.15min #9
396.1545
340.0888
813.2985
10m
m/z

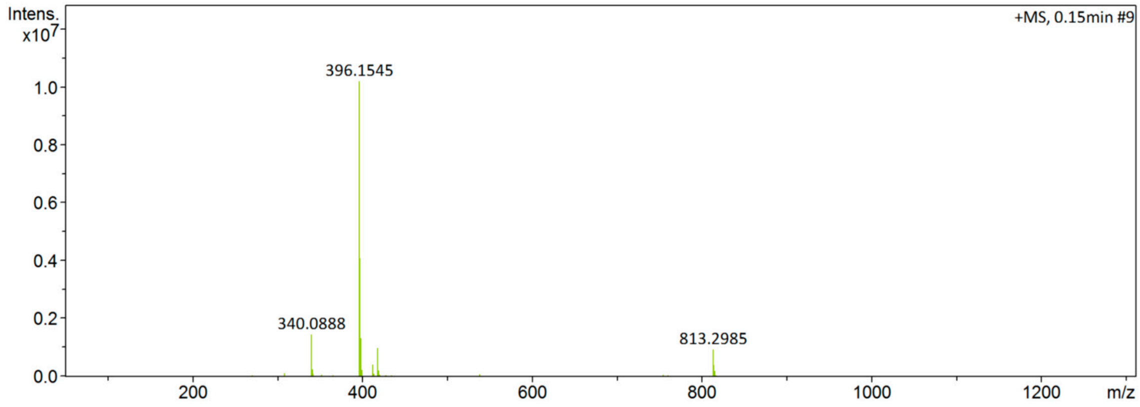
Intens.
x10^7
+MS, 0.15min #9
396.1545
340.0888
813.2985
m/z

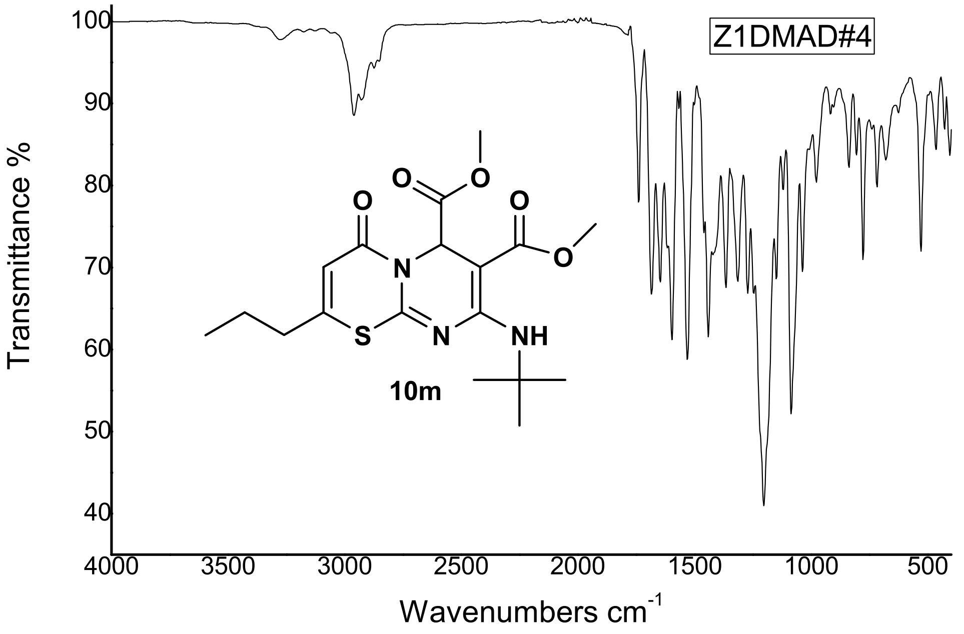

Z1DMAD#4
Transmittance %
100
90
80
70
60
50
40
4000
3500
3000
2500
2000
1500
1000
500
Wavenumbers cm-1
O
N
S
NH
10m

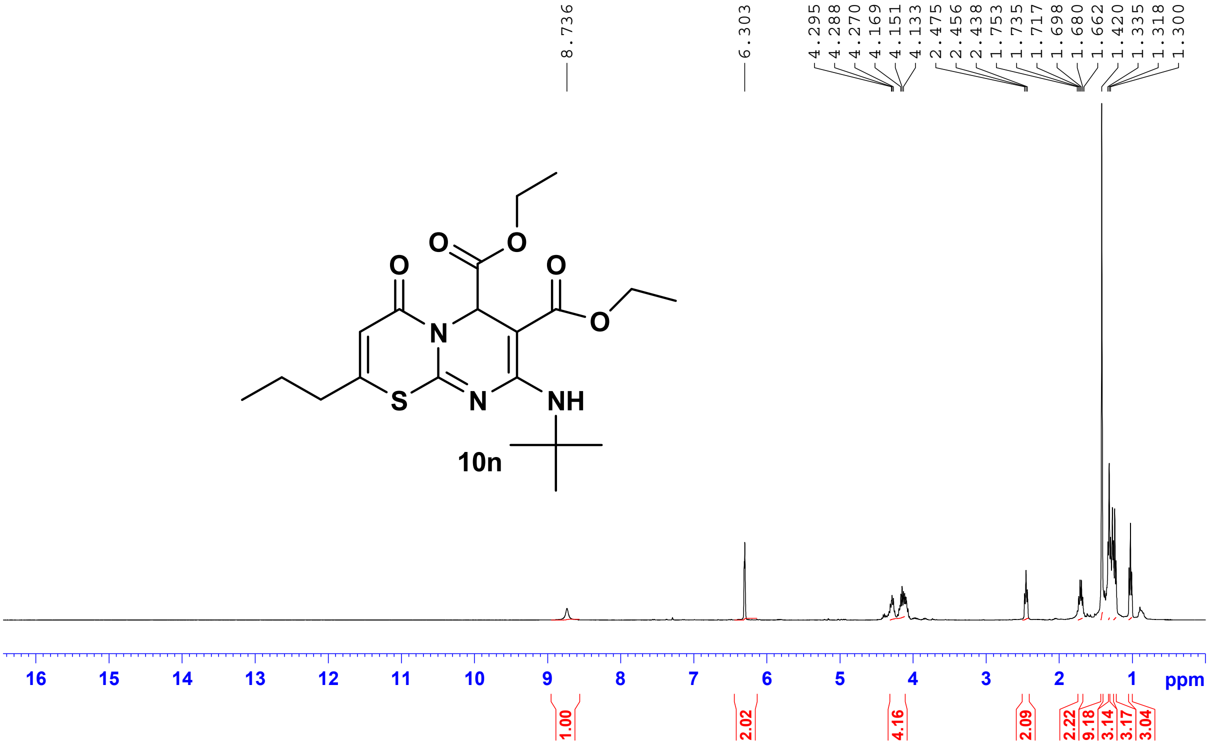

8.736
6.303
4.295
4.288
4.270
4.169
4.151
4.133
2.475
2.456
2.438
1.753
1.735
1.717
1.698
1.680
1.662
1.420
1.335
1.318
1.300
O
N
S
NH
10n
16 15 14 13 12 11 10 9 8 7 6 5 4 3 2 1 ppm
1.00
2.02
4.16
2.09
2.22
9.18
3.14
3.17
3.04

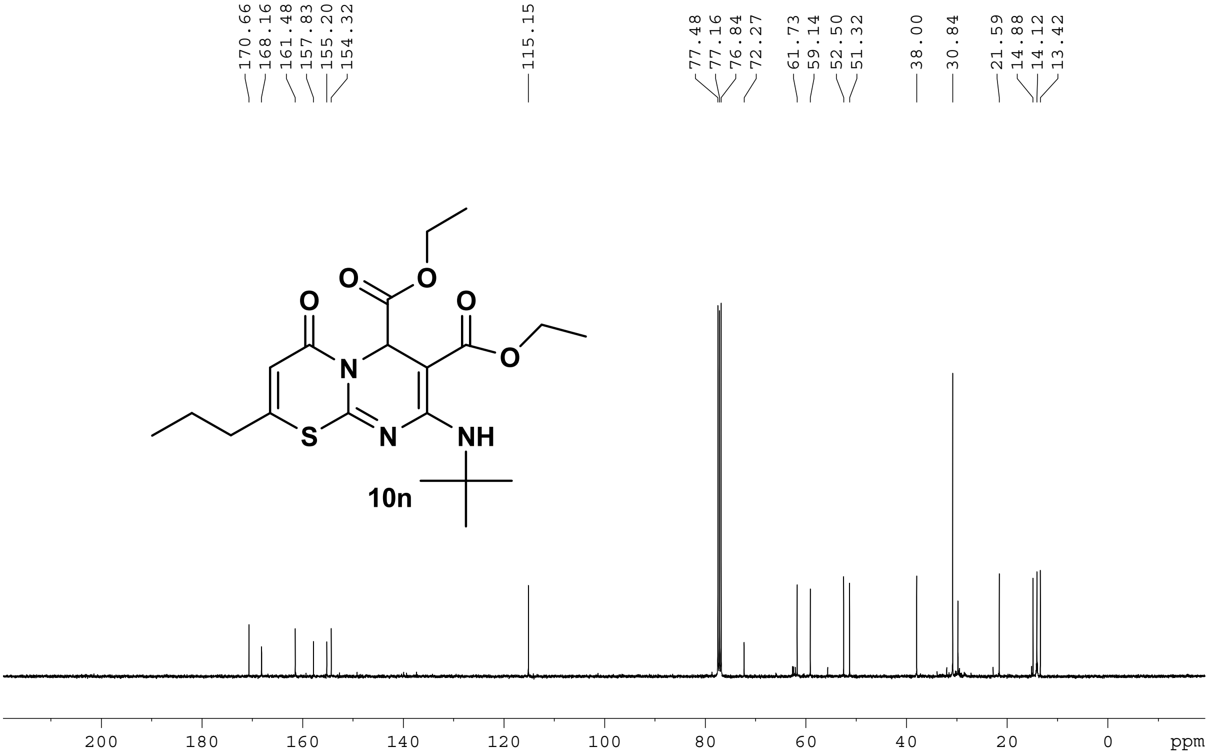
170.66
168.16
161.48
157.83
155.20
154.32
115.15
77.48
77.16
76.84
72.27
61.73
59.14
52.50
51.32
38.00
30.84
21.59
14.88
14.12
13.42
O
O
O
O
O
N
S
N
NH
10n
200
180
160
140
120
100
80
60
40
20
0
ppm

S

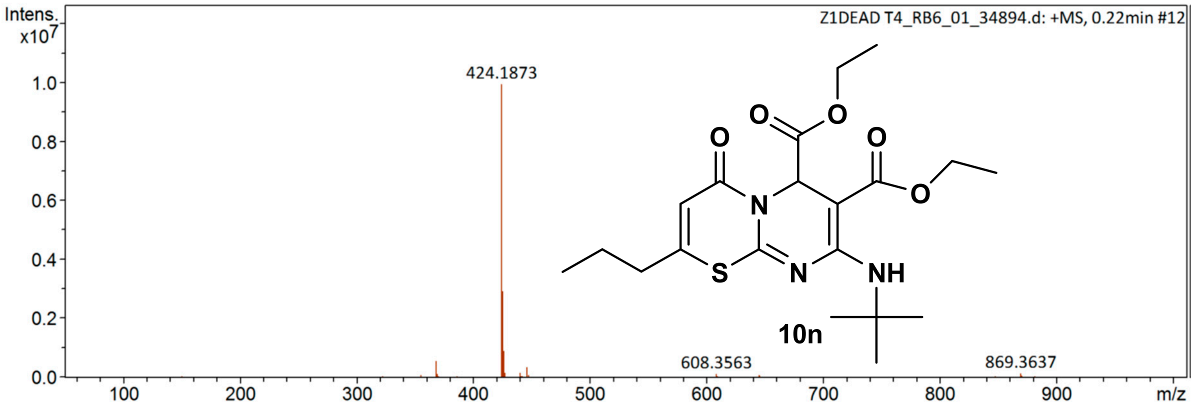
Intens.
x10^7
Z1DEAD T4_RB6_01_34894.d: +MS, 0.22min #12
424.1873
608.3563
869.3637
10n
m/z

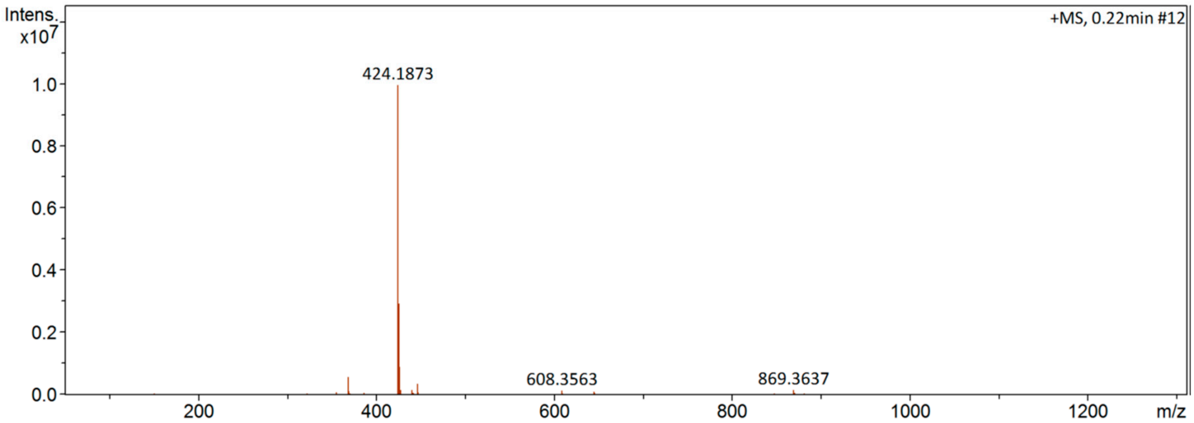
Intens.
x10^7
+MS, 0.22min #12
424.1873
608.3563
869.3637
m/z

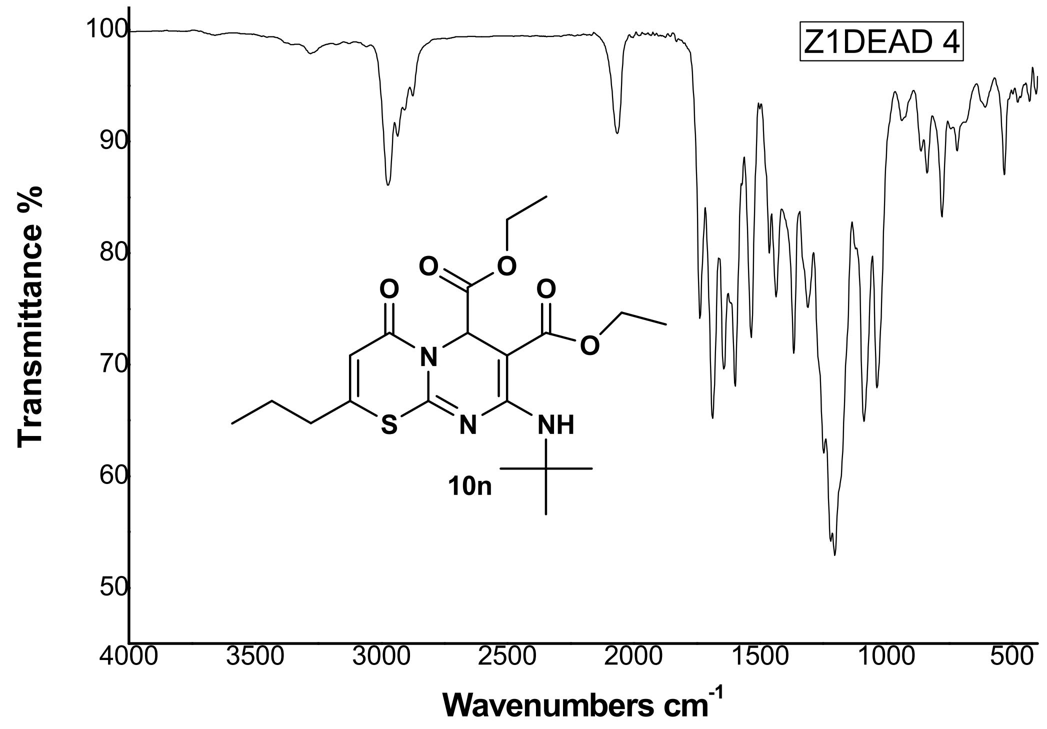
Z1DEAD 4
Transmittance %
Wavenumbers cm-1
100
90
80
70
60
50
4000
3500
3000
2500
2000
1500
1000
500
10n

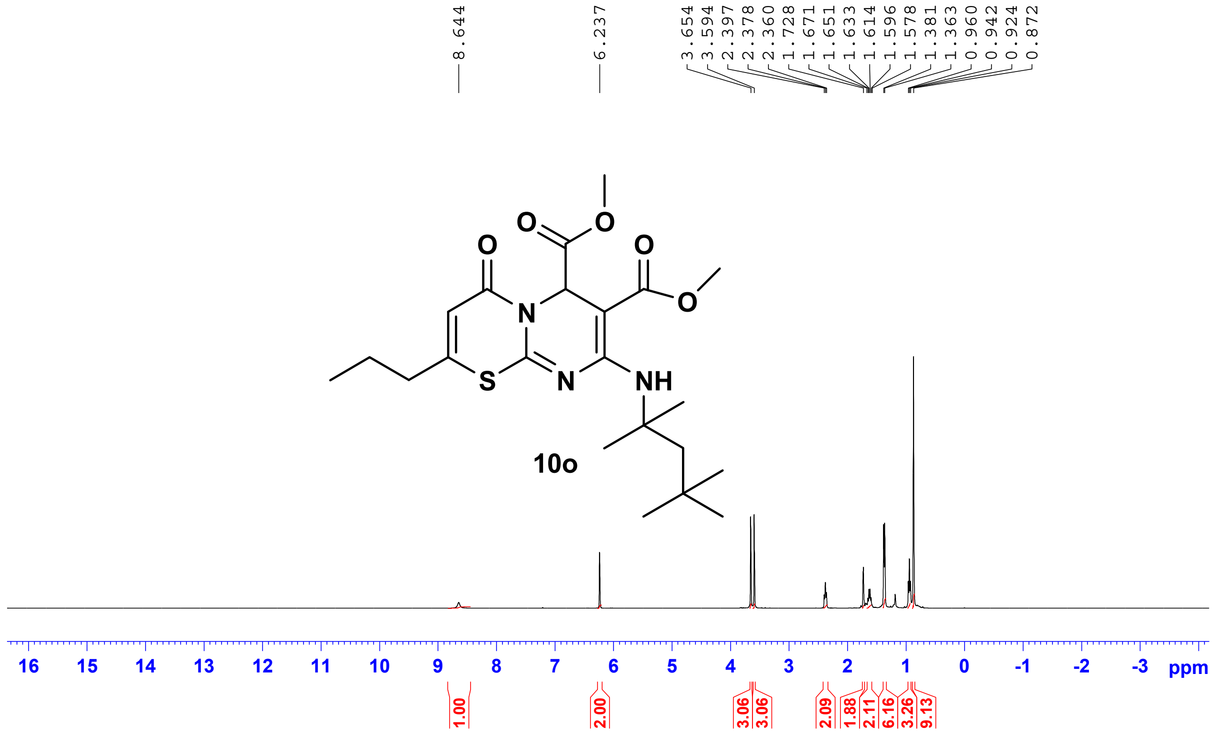
8.644
6.237
3.654
3.594
2.397
2.378
2.360
1.728
1.671
1.651
1.633
1.614
1.596
1.578
1.381
1.363
0.960
0.942
0.924
0.872
10o
16 15 14 13 12 11 10 9 8 7 6 5 4 3 2 1 0 -1 -2 -3 ppm
1.00
2.00
3.06
3.06
2.09
1.88
2.11
6.16
3.26
9.13

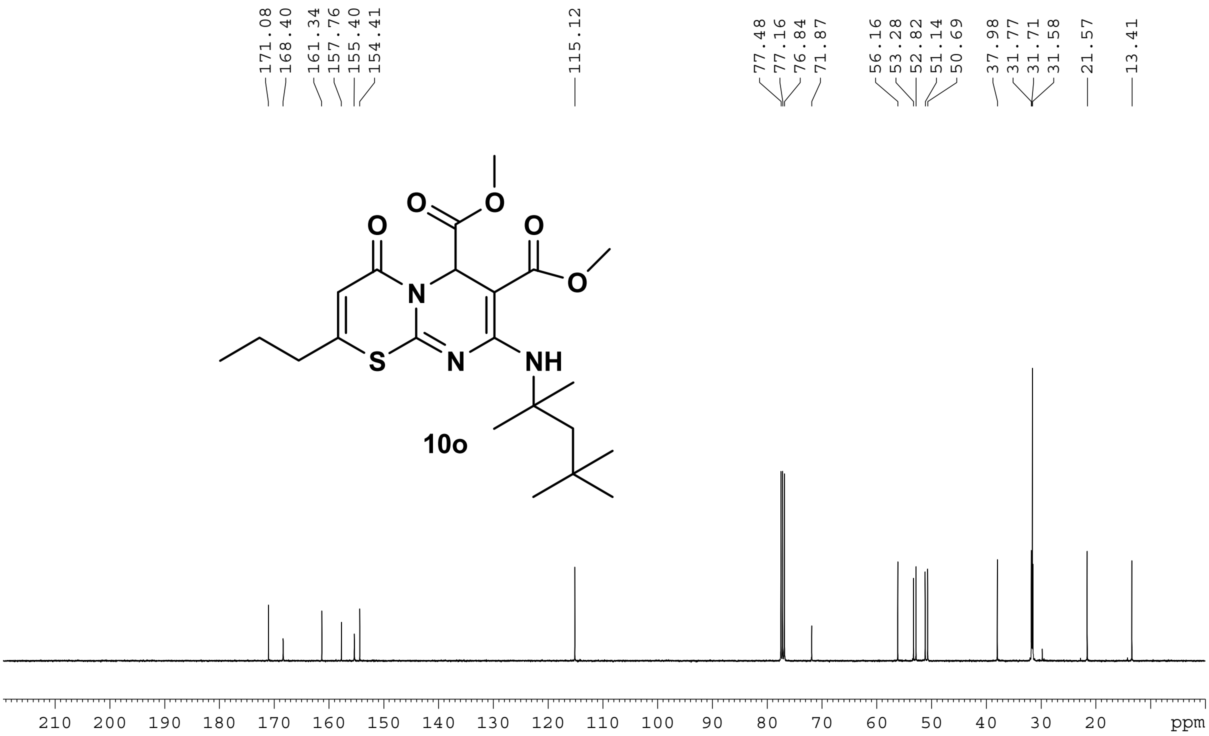
171.08
168.40
161.34
157.76
155.40
154.41
115.12
77.48
77.16
76.84
71.87
56.16
53.28
52.82
51.14
50.69
37.98
31.77
31.71
31.58
21.57
13.41
10o
210 200 190 180 170 160 150 140 130 120 110 100 90 80 70 60 50 40 30 20 ppm

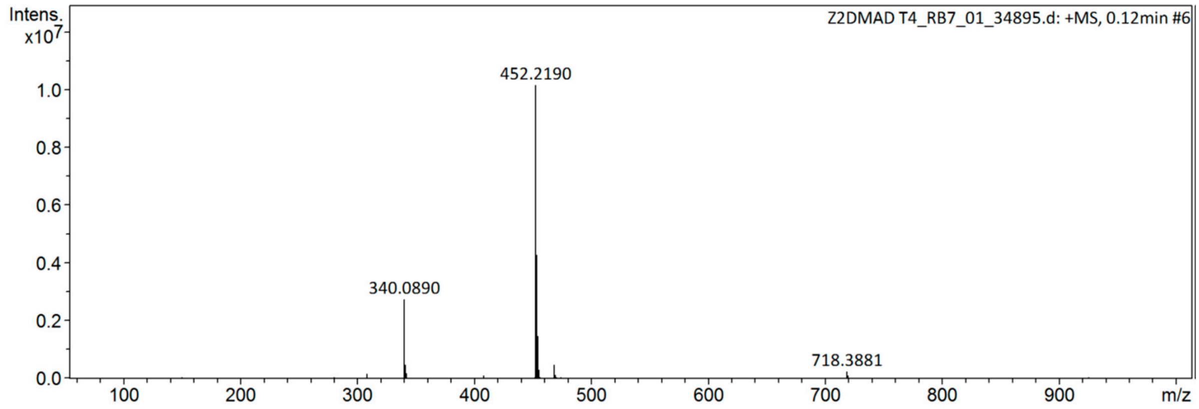
Intens.
x10^7
Z2DMAD T4_RB7_01_34895.d: +MS, 0.12min #6
452.2190
340.0890
718.3881
m/z

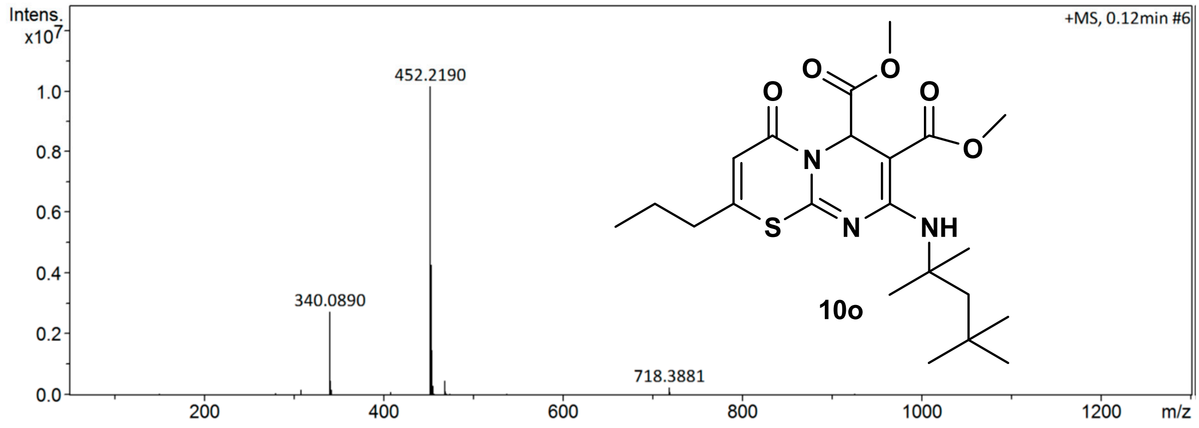
Intens.
x10^7
+MS, 0.12min #6
452.2190
340.0890
718.3881
10o
m/z

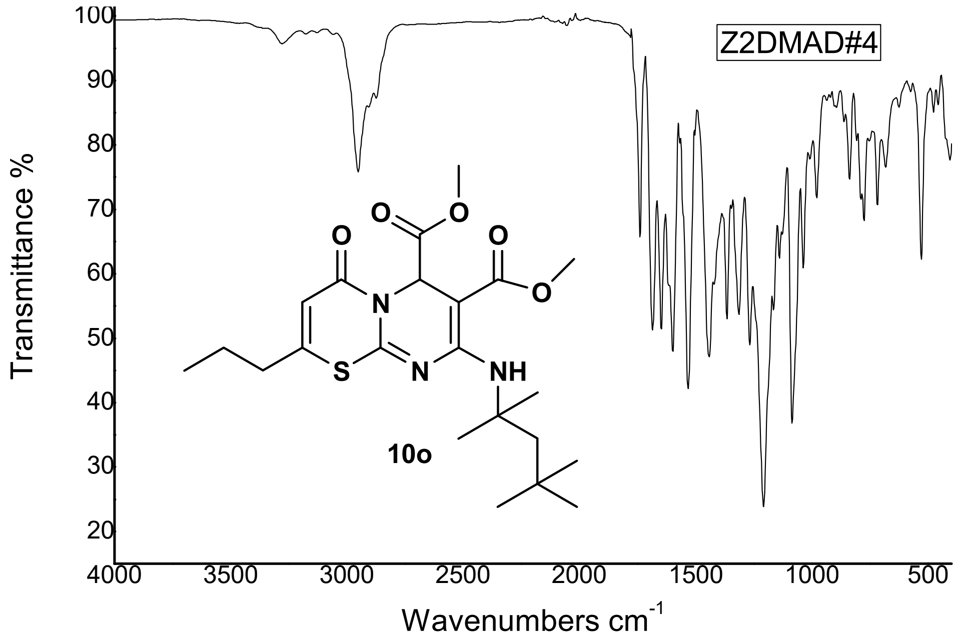
Z2DMAD#4
Transmittance %
100
90
80
70
60
50
40
30
20
4000
3500
3000
2500
2000
1500
1000
500
Wavenumbers cm-1
O
O
O
O
O
N
S
N
NH
10o

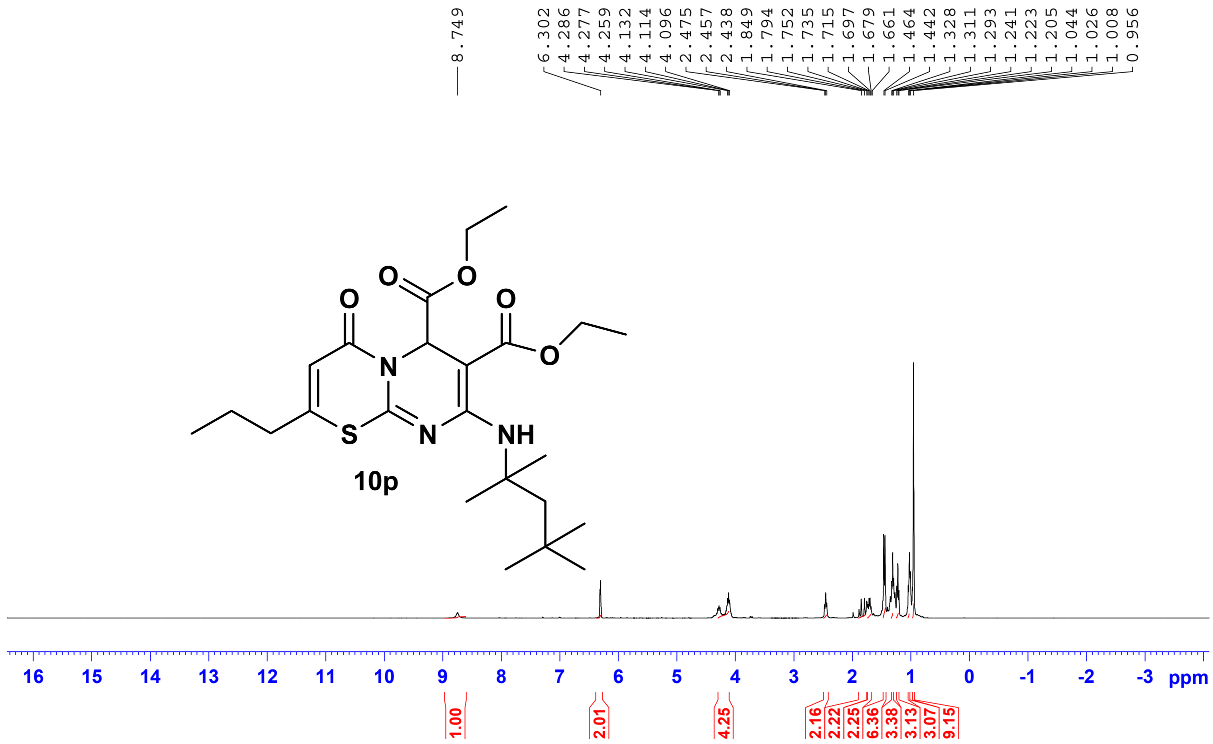
8.749
6.302
4.286
4.277
4.259
4.132
4.114
4.096
2.475
2.457
2.438
1.849
1.794
1.752
1.735
1.715
1.697
1.679
1.661
1.464
1.442
1.328
1.311
1.293
1.241
1.223
1.205
1.044
1.026
1.008
0.956
O
O
O
O
O
N
S
N
NH
10p
16 15 14 13 12 11 10 9 8 7 6 5 4 3 2 1 0 -1 -2 -3 ppm
1.00
2.01
4.25
2.16
2.22
2.25
6.36
3.38
3.13
3.07
9.15

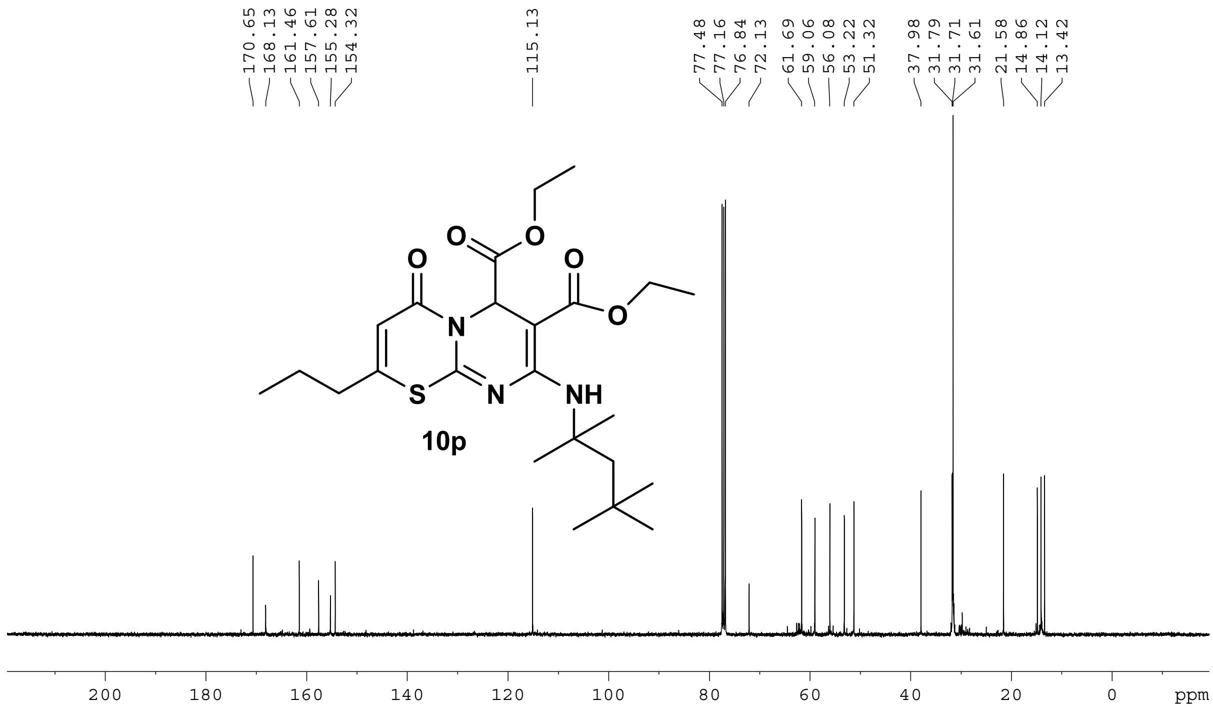
170.65
168.13
161.46
157.61
155.28
154.32
115.13
77.48
77.16
76.84
72.13
61.69
59.06
56.08
53.22
51.32
37.98
31.79
31.71
31.61
21.58
14.86
14.12
13.42
10p
200 180 160 140 120 100 80 60 40 20 0 ppm

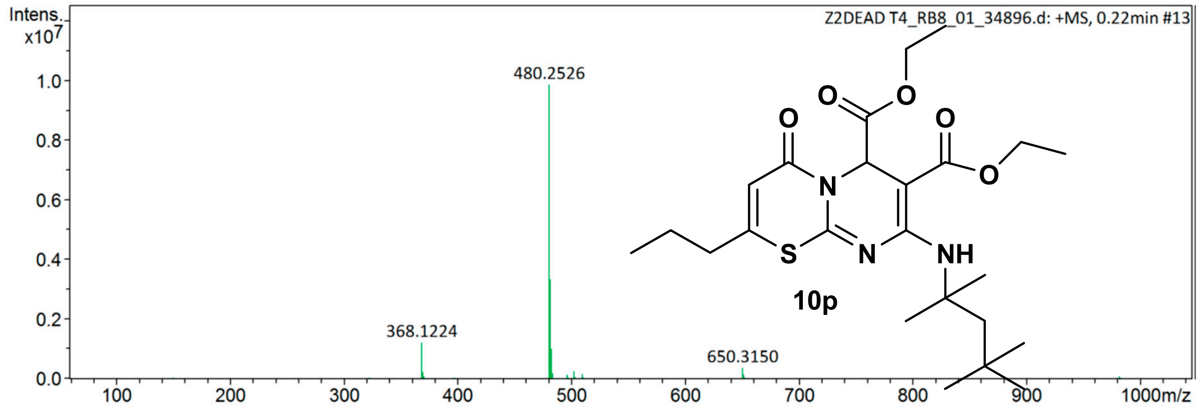
Intens.
x10^7
Z2DEAD T4_RB8_01_34896.d: +MS, 0.22min #13
480.2526
368.1224
650.3150
10p
100 200 300 400 500 600 700 800 900 1000m/z

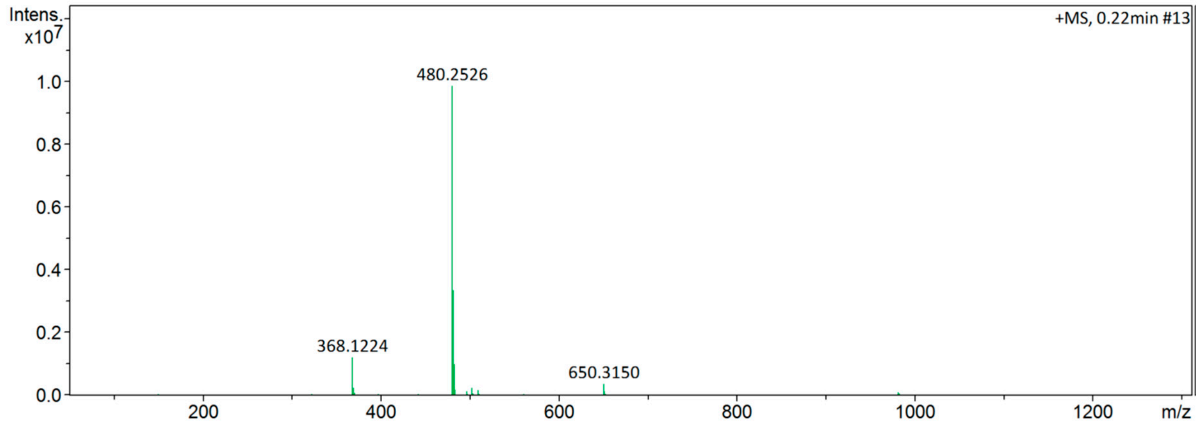
Intens.
x10^7
+MS, 0.22min #13
480.2526
368.1224
650.3150
200 400 600 800 1000 1200 m/z

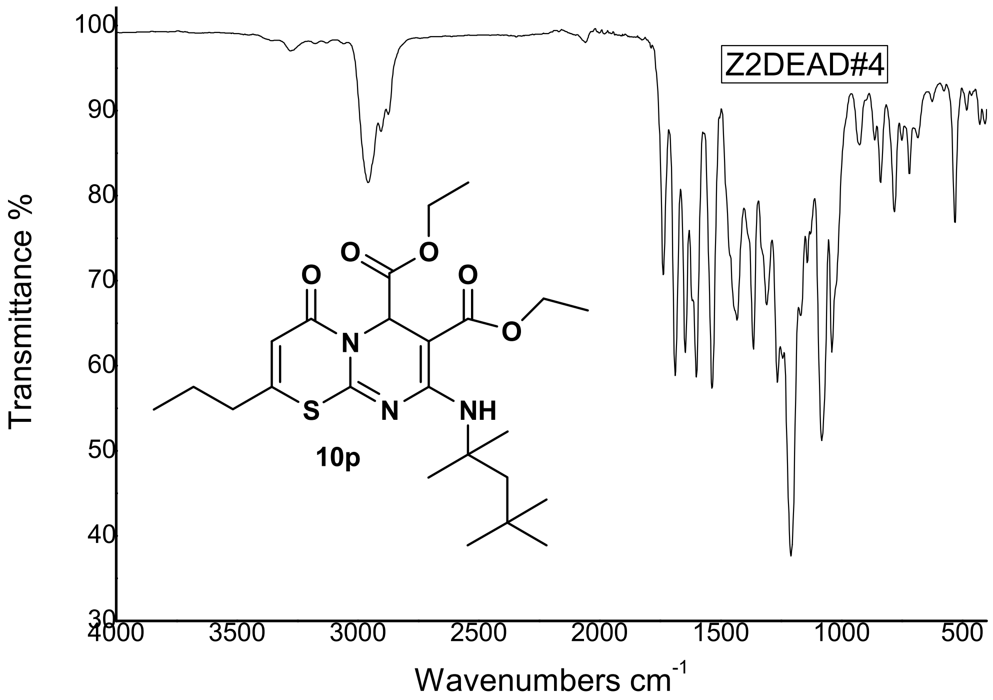
Z2DEAD#4
Transmittance %
Wavenumbers cm-1
100
90
80
70
60
50
40
30
4000
3500
3000
2500
2000
1500
1000
500
O
O
O
O
O
O
N
S
N
NH
10p

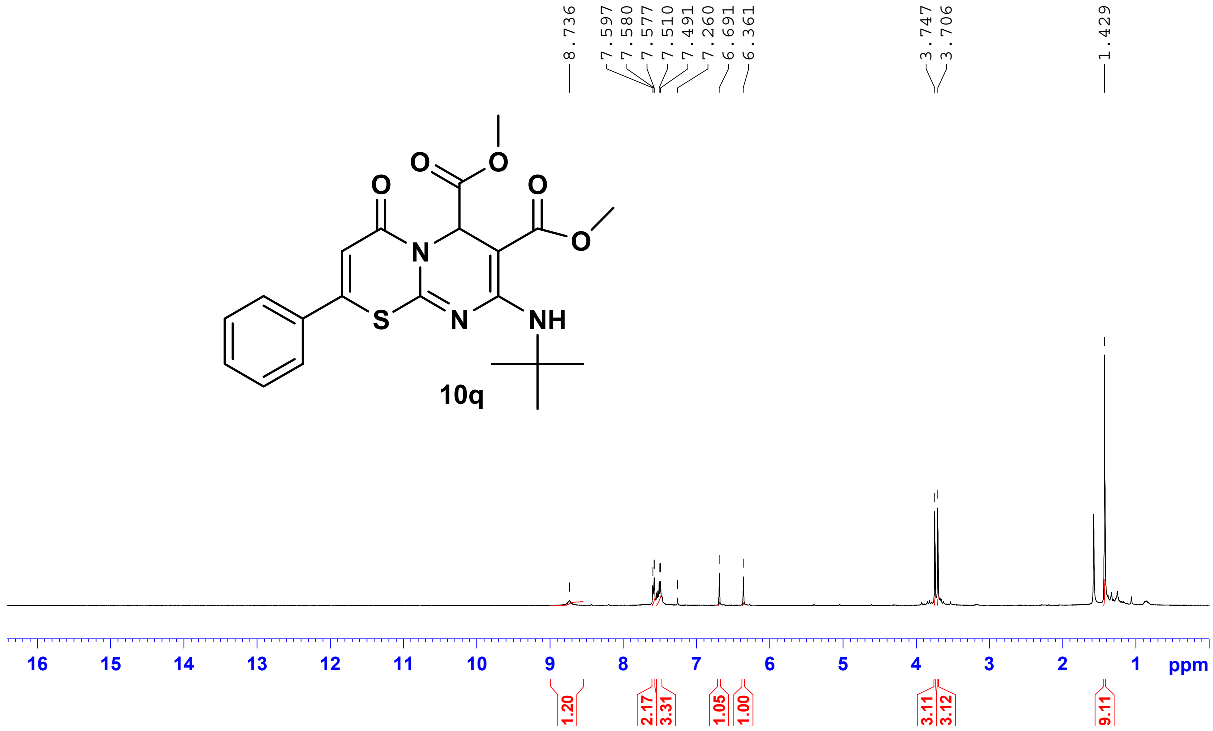
8.736
7.597
7.580
7.577
7.510
7.491
7.260
6.691
6.361
3.747
3.706
1.429
O
O
O
O
O
O
N
S
N
NH
10q
16
15
14
13
12
11
10
9
8
7
6
5
4
3
2
1
ppm
1.20
2.17
3.31
1.05
1.00
3.11
3.12
9.11

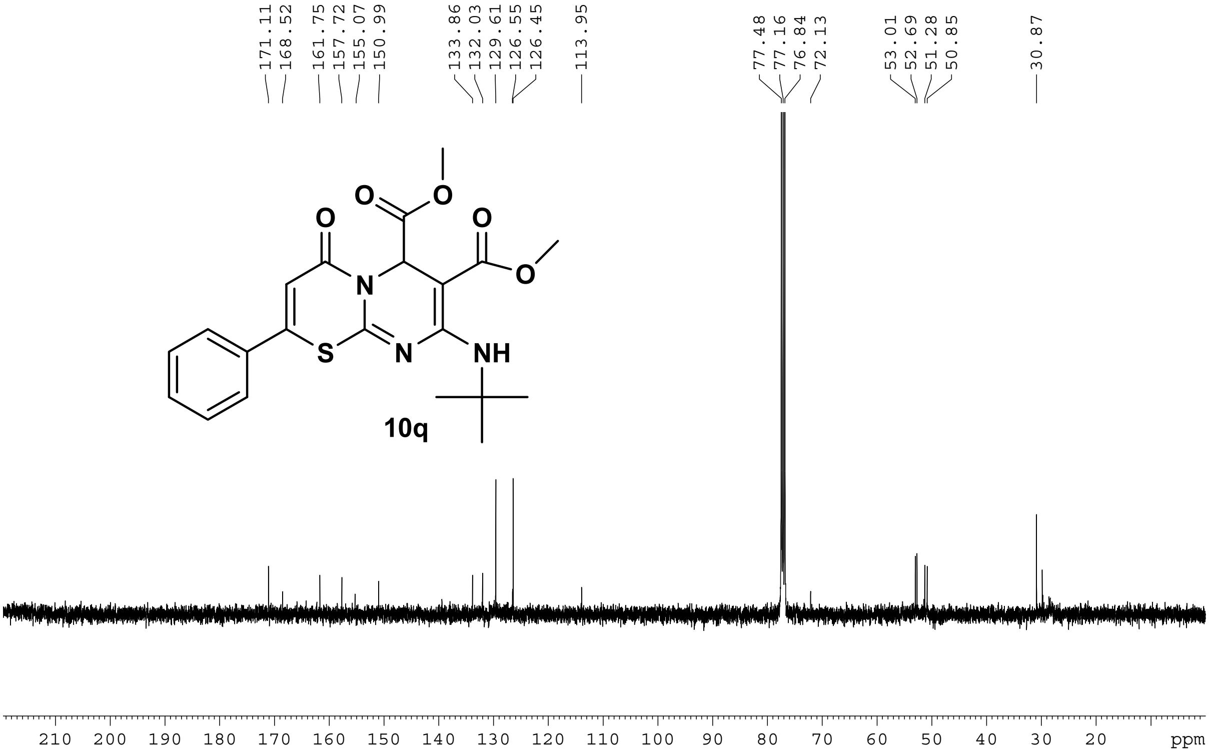

171.11
168.52
161.75
157.72
155.07
150.99
133.86
132.03
129.61
126.55
126.45
113.95
77.48
77.16
76.84
72.13
53.01
52.69
51.28
50.85
30.87
O
O
O
O
O
N
S
N
NH
10q
210 200 190 180 170 160 150 140 130 120 110 100 90 80 70 60 50 40 30 20 ppm

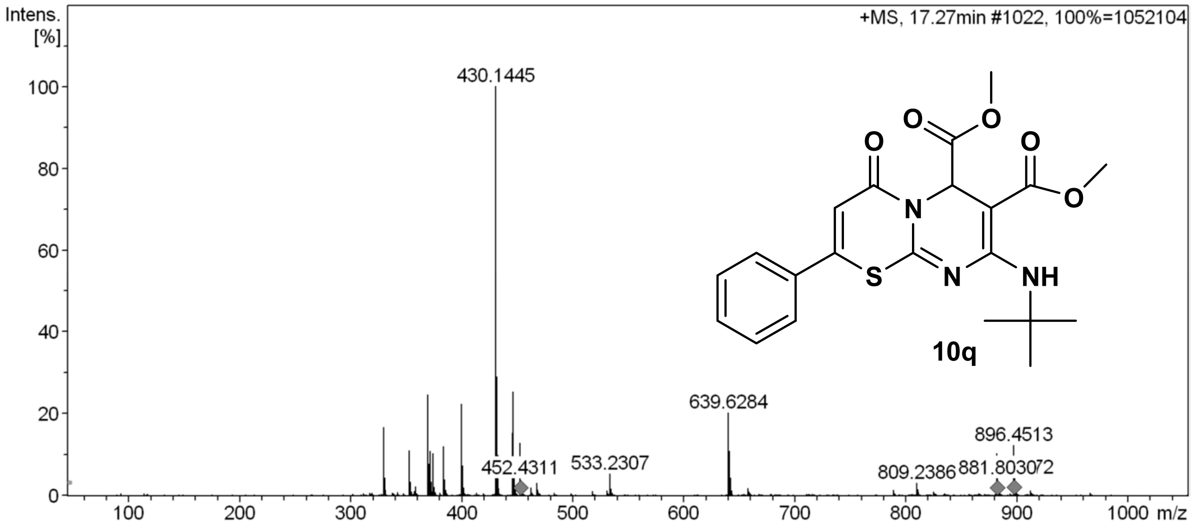

Intens. [%]
+MS, 17.27min #1022, 100%=1052104
430.1445
639.6284
896.4513
452.4311
533.2307
809.2386
881.803072
O
O
O
O
O
N
S
N
NH
10q
0 20 40 60 80 100
100 200 300 400 500 600 700 800 900 1000 m/z

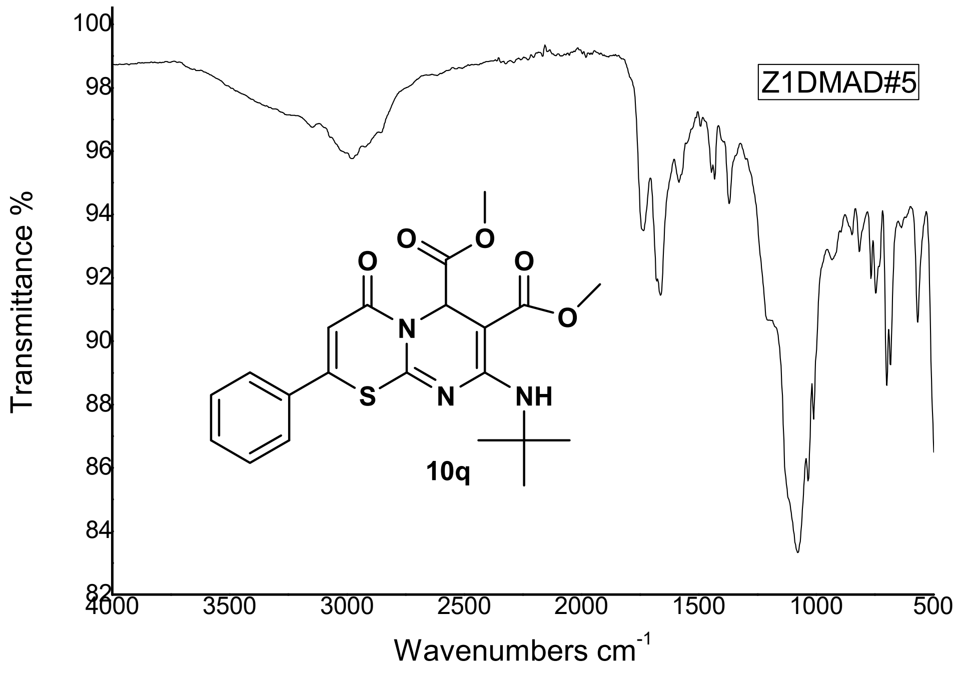
Z1DMAD#5
Transmittance %
Wavenumbers cm-1
10q
O
N
S
NH

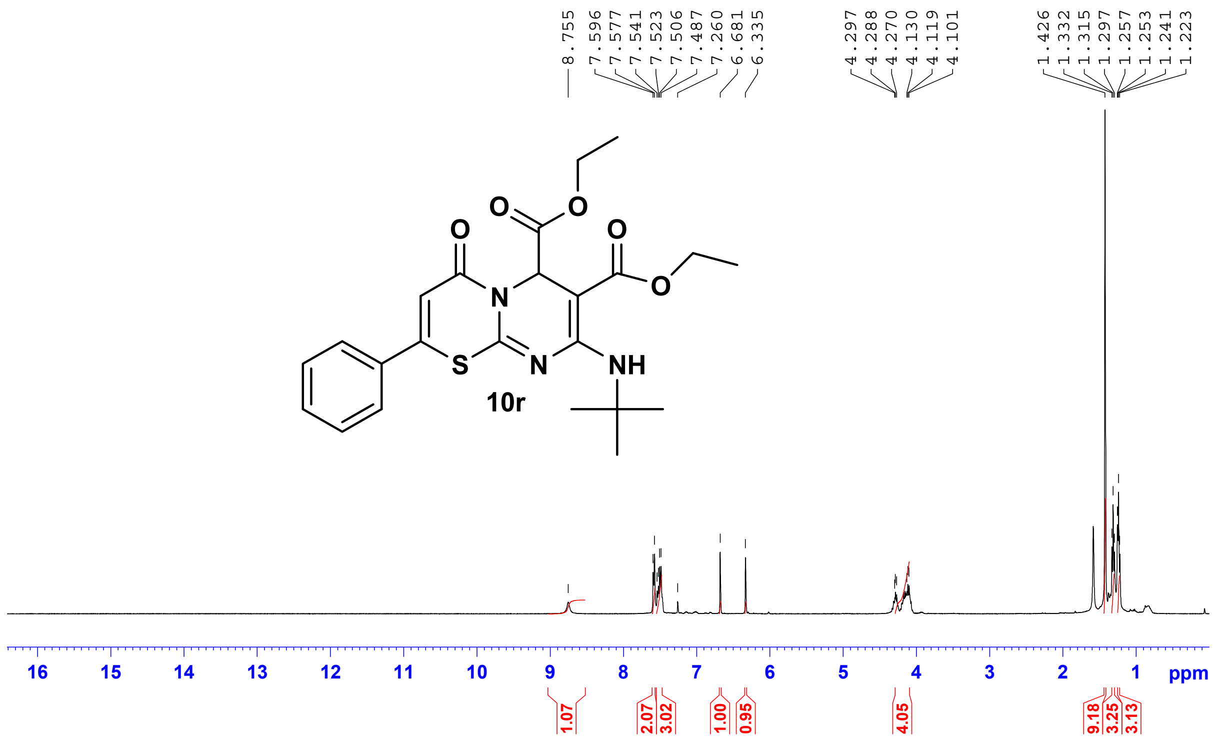
8.755
7.596
7.577
7.541
7.523
7.506
7.487
7.260
6.681
6.335
4.297
4.288
4.270
4.130
4.119
4.101
1.426
1.332
1.315
1.297
1.257
1.253
1.241
1.223
10r
ppm
1.07
2.07
3.02
1.00
0.95
4.05
9.18
3.25
3.13

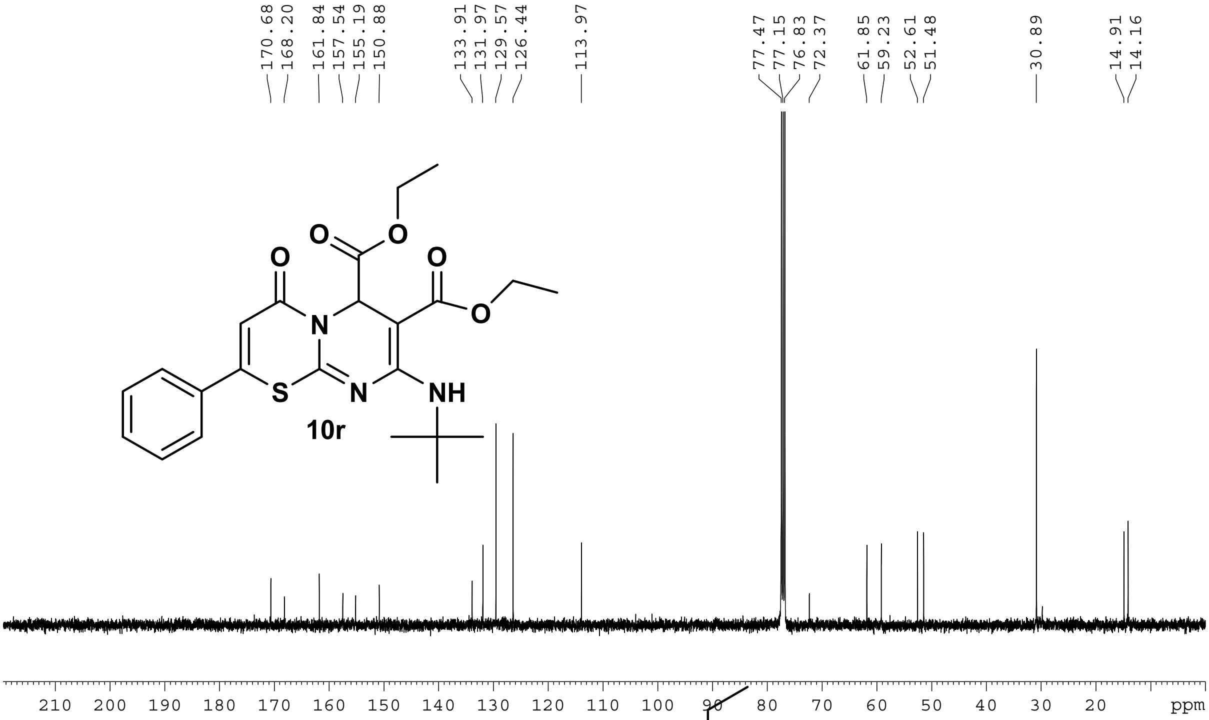
170.68
168.20
161.84
157.54
155.19
150.88
133.91
131.97
129.57
126.44
113.97
77.47
77.15
76.83
72.37
61.85
59.23
52.61
51.48
30.89
14.91
14.16
O
O
O
O
O
N
S
N
NH
10r
210 200 190 180 170 160 150 140 130 120 110 100 90 80 70 60 50 40 30 20 ppm

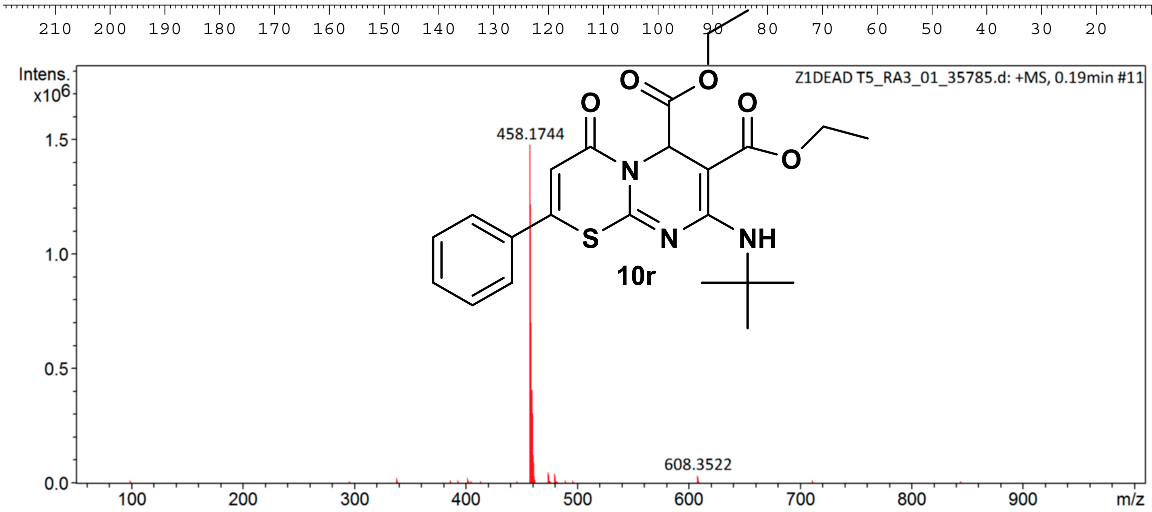
Intens.
x10^6
Z1DEAD T5_RA3_01_35785.d: +MS, 0.19min #11
458.1744
608.3522
O
O
O
O
O
N
S
N
NH
10r
1.5
1.0
0.5
0.0
100 200 300 400 500 600 700 800 900 m/z

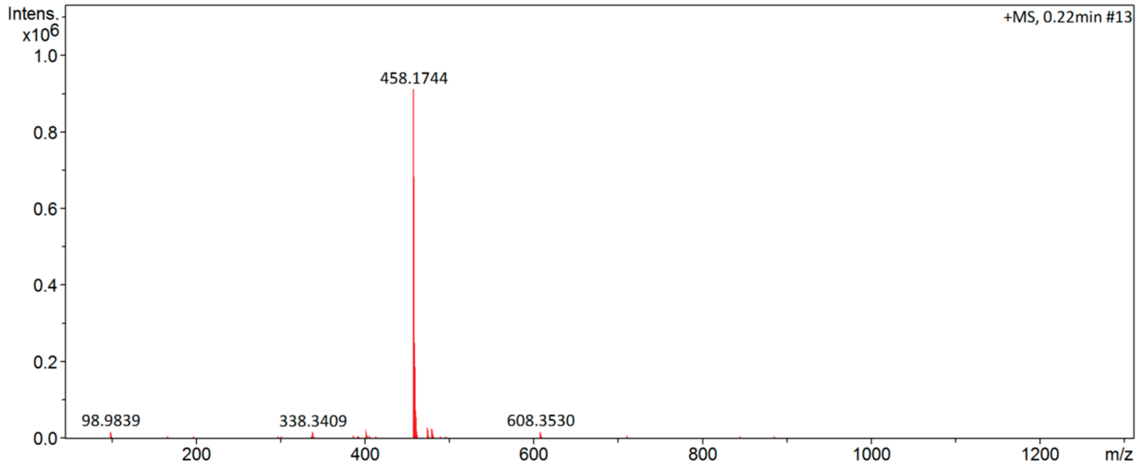
Intens.
x10^6
+MS, 0.22min #13
458.1744
98.9839
338.3409
608.3530
1.0
0.8
0.6
0.4
0.2
0.0
200 400 600 800 1000 1200 m/z

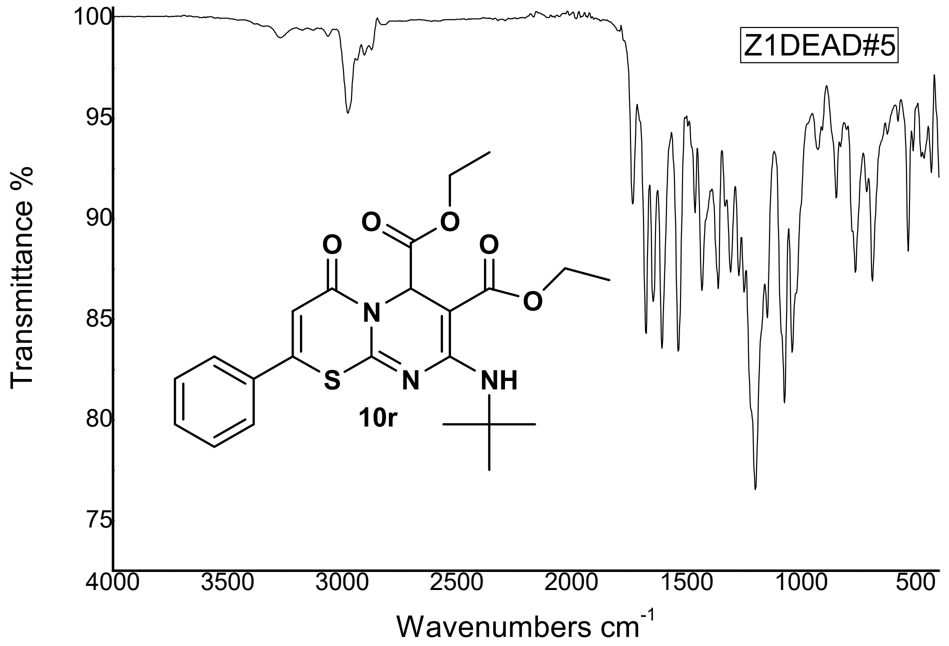

Z1DEAD#5
Transmittance %
Wavenumbers cm-1
100
95
90
85
80
75
4000
3500
3000
2500
2000
1500
1000
500
10r

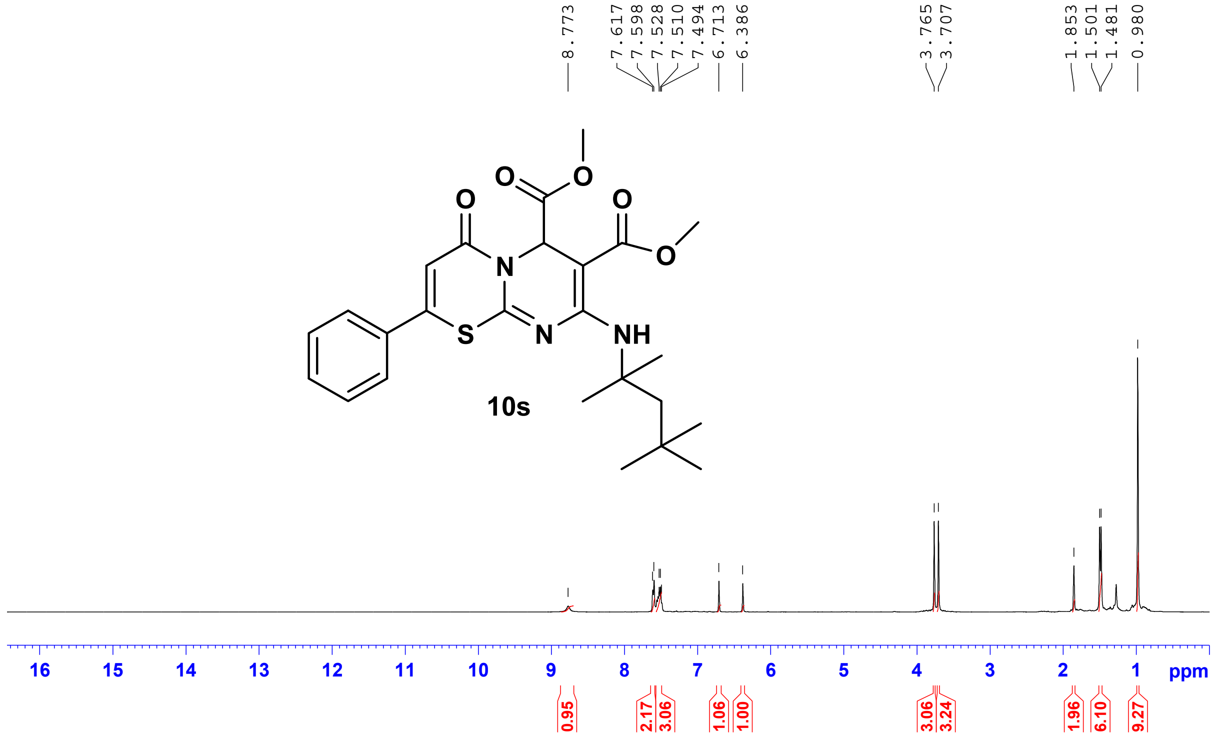

8.773
7.617
7.598
7.528
7.510
7.494
6.713
6.386
3.765
3.707
1.853
1.501
1.481
0.980
10s
ppm
0.95
2.17
3.06
1.06
1.00
3.06
3.24
1.96
6.10
9.27

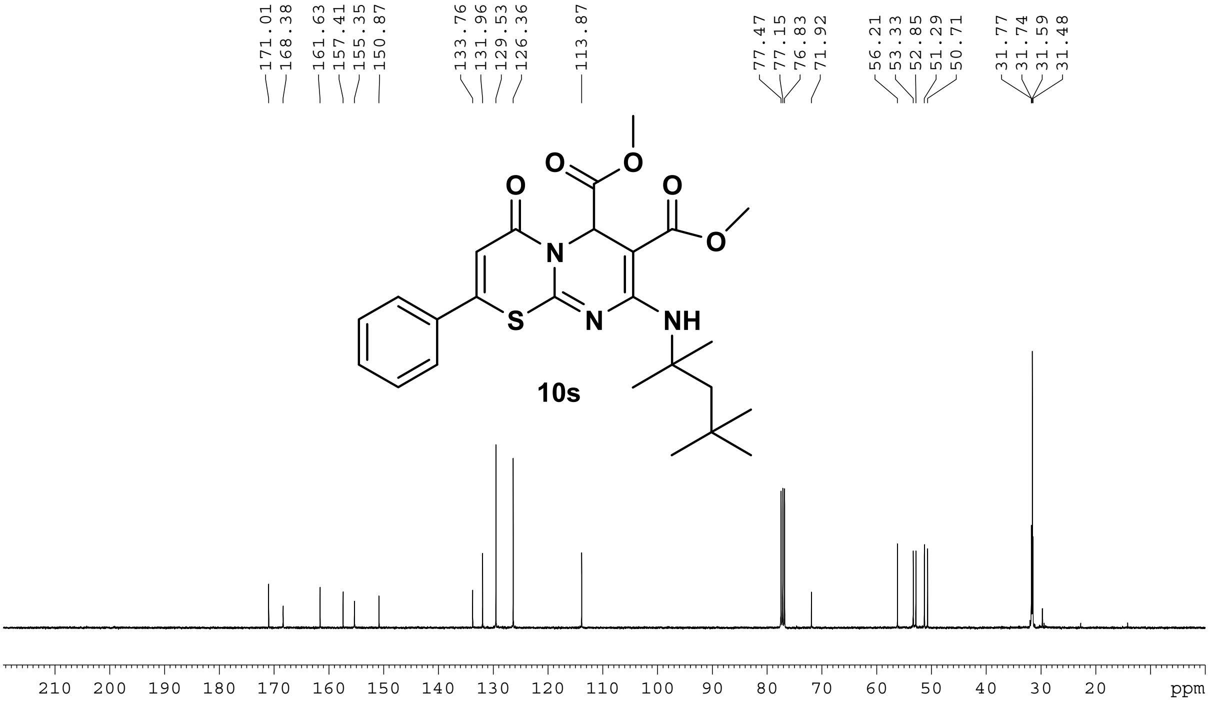
171.01
168.38
161.63
157.41
155.35
150.87
133.76
131.96
129.53
126.36
113.87
77.47
77.15
76.83
71.92
56.21
53.33
52.85
51.29
50.71
31.77
31.74
31.59
31.48
10s
210 200 190 180 170 160 150 140 130 120 110 100 90 80 70 60 50 40 30 20 ppm

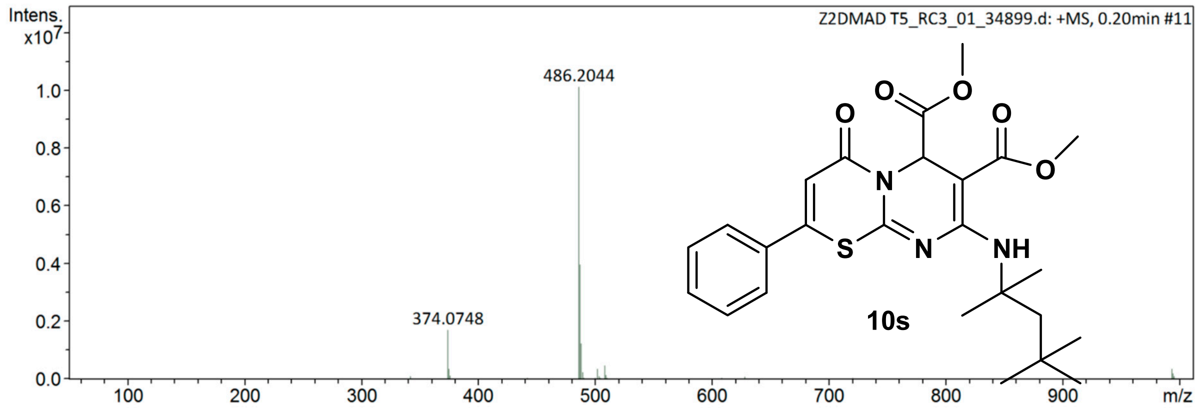
Intens.
x10^7
Z2DMAD T5_RC3_01_34899.d: +MS, 0.20min #11
486.2044
374.0748
10s
m/z

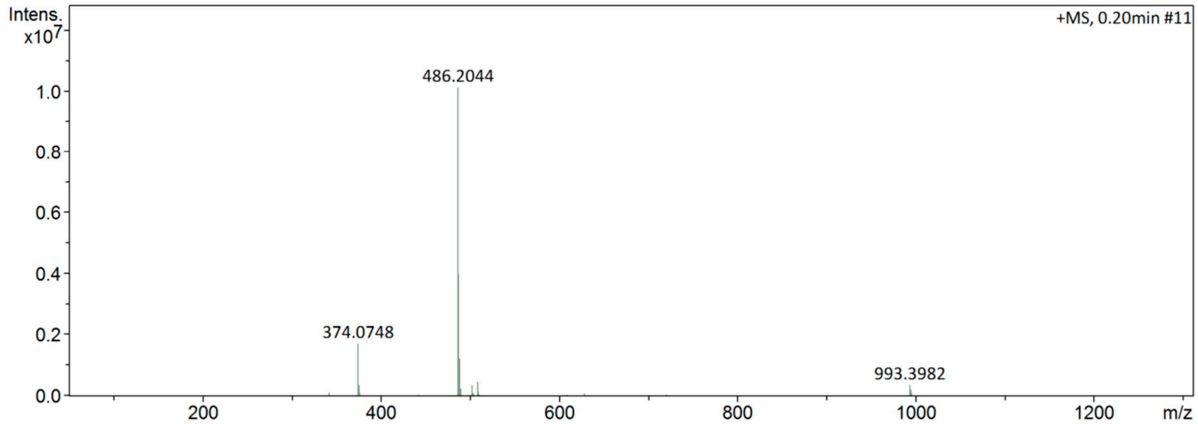
Intens.
x10^7
+MS, 0.20min #11
486.2044
374.0748
993.3982
m/z

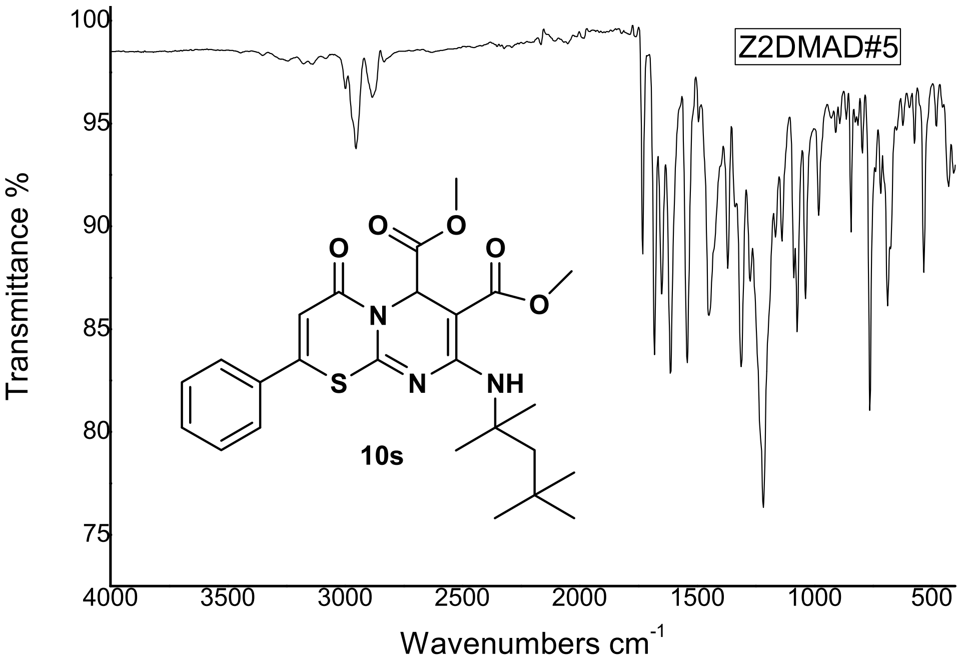

Z2DMAD#5
Transmittance %
Wavenumbers cm-1
10s

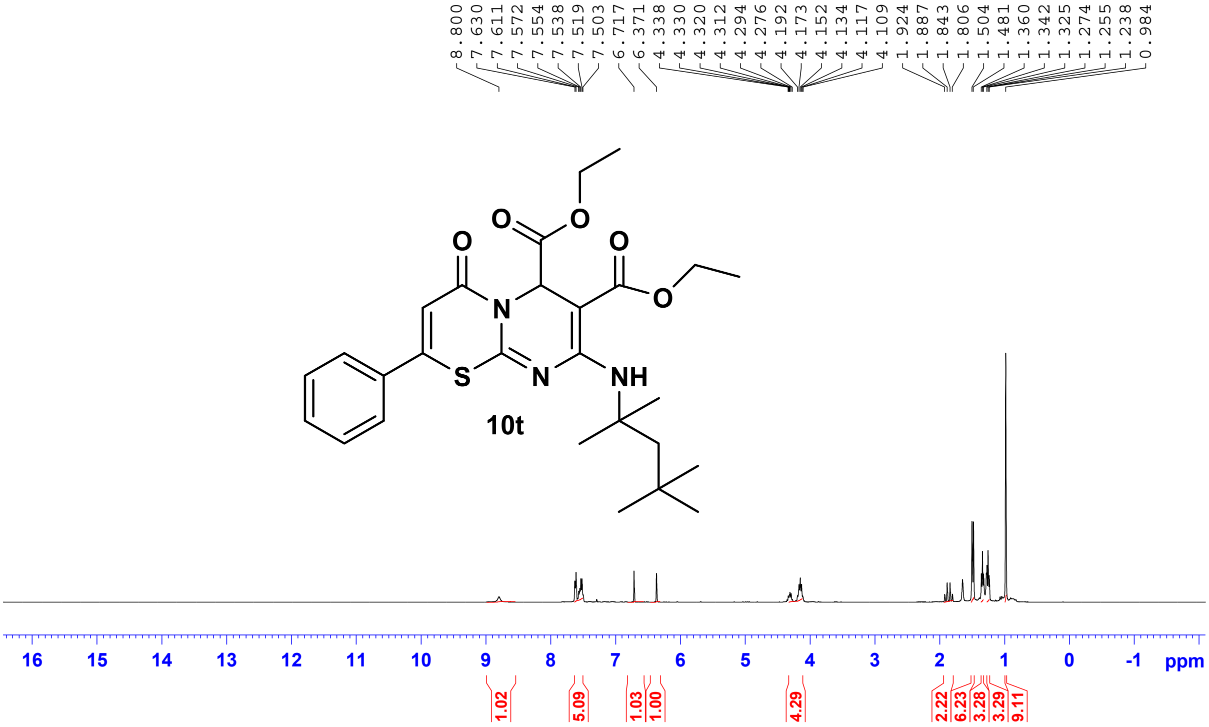

8.800
7.630
7.611
7.572
7.554
7.538
7.519
7.503
6.717
6.371
4.338
4.330
4.320
4.312
4.294
4.276
4.192
4.173
4.152
4.134
4.117
4.109
1.924
1.887
1.843
1.806
1.504
1.481
1.360
1.342
1.325
1.274
1.255
1.238
0.984
10t
ppm
1.02
5.09
1.03
1.00
4.29
2.22
6.23
3.28
3.29
9.11

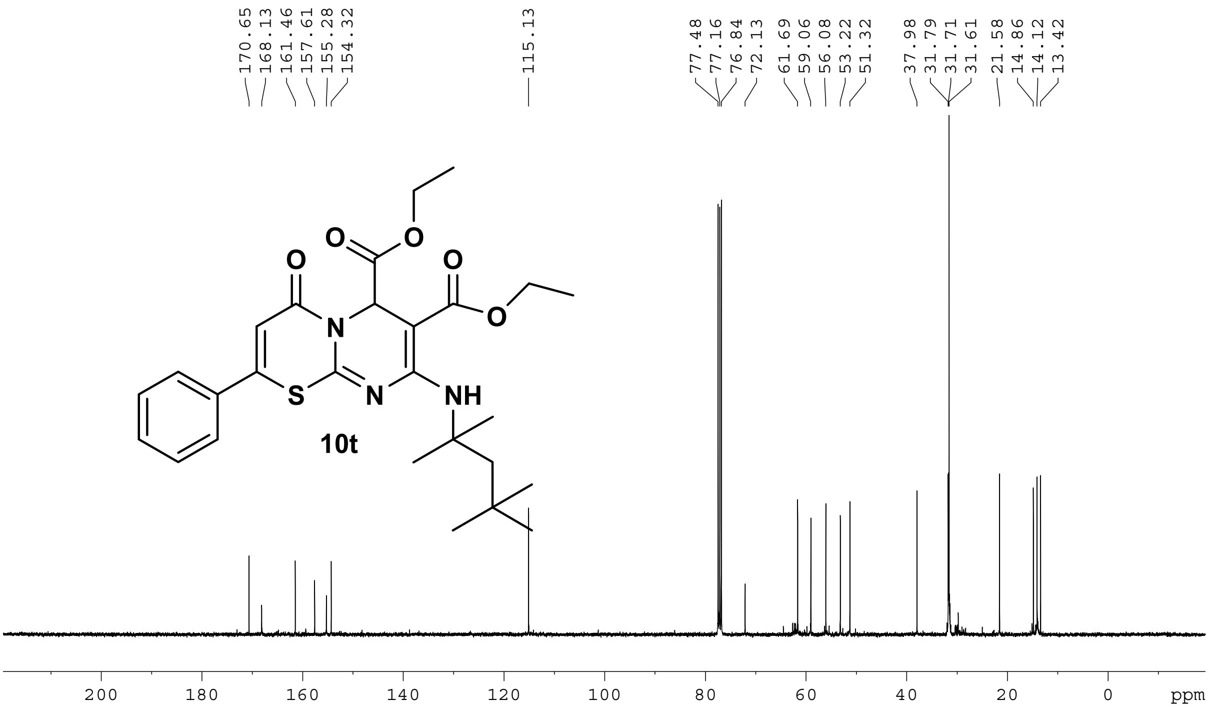
170.65
168.13
161.46
157.61
155.28
154.32
115.13
77.48
77.16
76.84
72.13
61.69
59.06
56.08
53.22
51.32
37.98
31.79
31.71
31.61
21.58
14.86
14.12
13.42
O
O
O
O
O
O
N
S
N
NH
10t
200
180
160
140
120
100
80
60
40
20
0
ppm

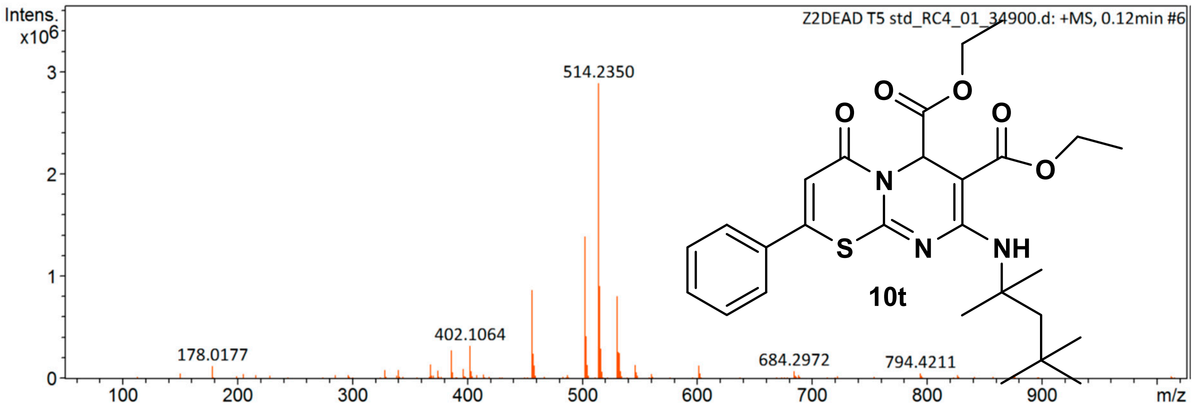
Intens.
x10^6
Z2DEAD T5 std_RC4_01_34900.d: +MS, 0.12min #6
514.2350
402.1064
178.0177
684.2972
794.4211
10t
m/z

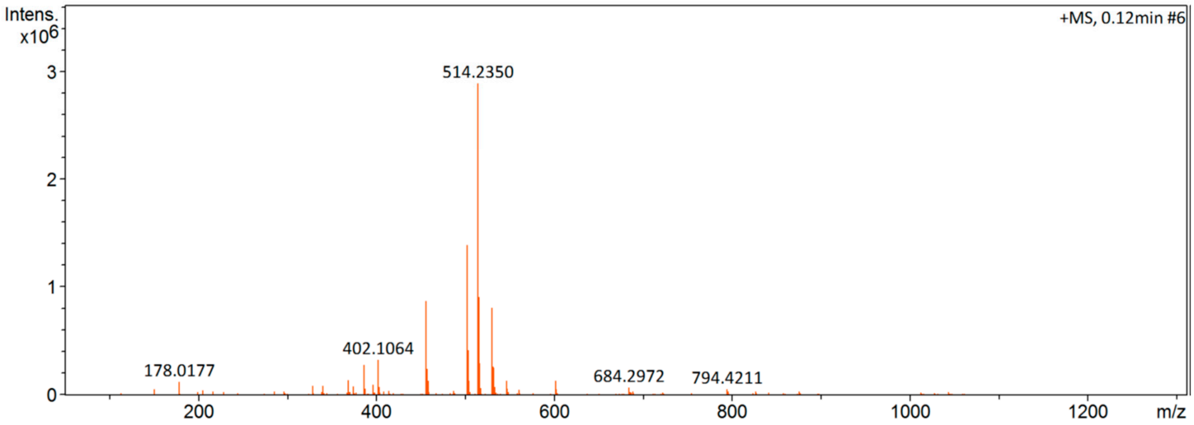
Intens.
x10^6
+MS, 0.12min #6
514.2350
402.1064
178.0177
684.2972
794.4211
m/z

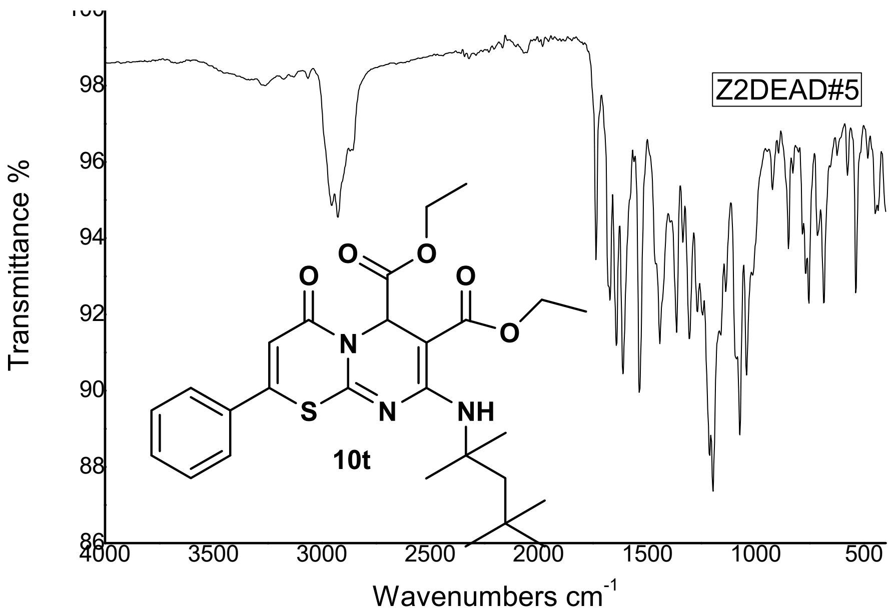
Transmittance %
98
96
94
92
90
88
86
4000
3500
3000
2500
2000
1500
1000
500
Wavenumbers cm-1
Z2DEAD#5
O
O
O
O
O
N
S
N
NH
10t

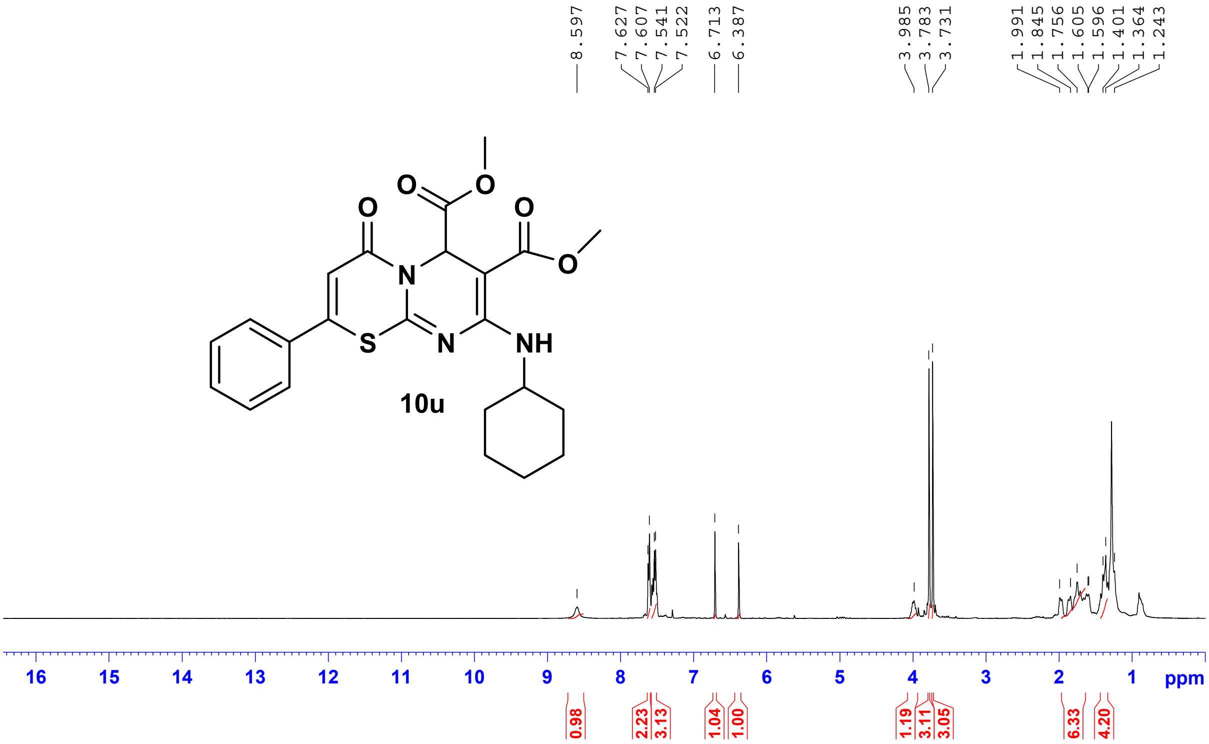
8.597
7.627
7.607
7.541
7.522
6.713
6.387
3.985
3.783
3.731
1.991
1.845
1.756
1.605
1.596
1.401
1.364
1.243
O
O
O
O
O
N
S
N
NH
10u
16
15
14
13
12
11
10
9
8
7
6
5
4
3
2
1
ppm
0.98
2.23
3.13
1.04
1.00
1.19
3.11
3.05
6.33
4.20

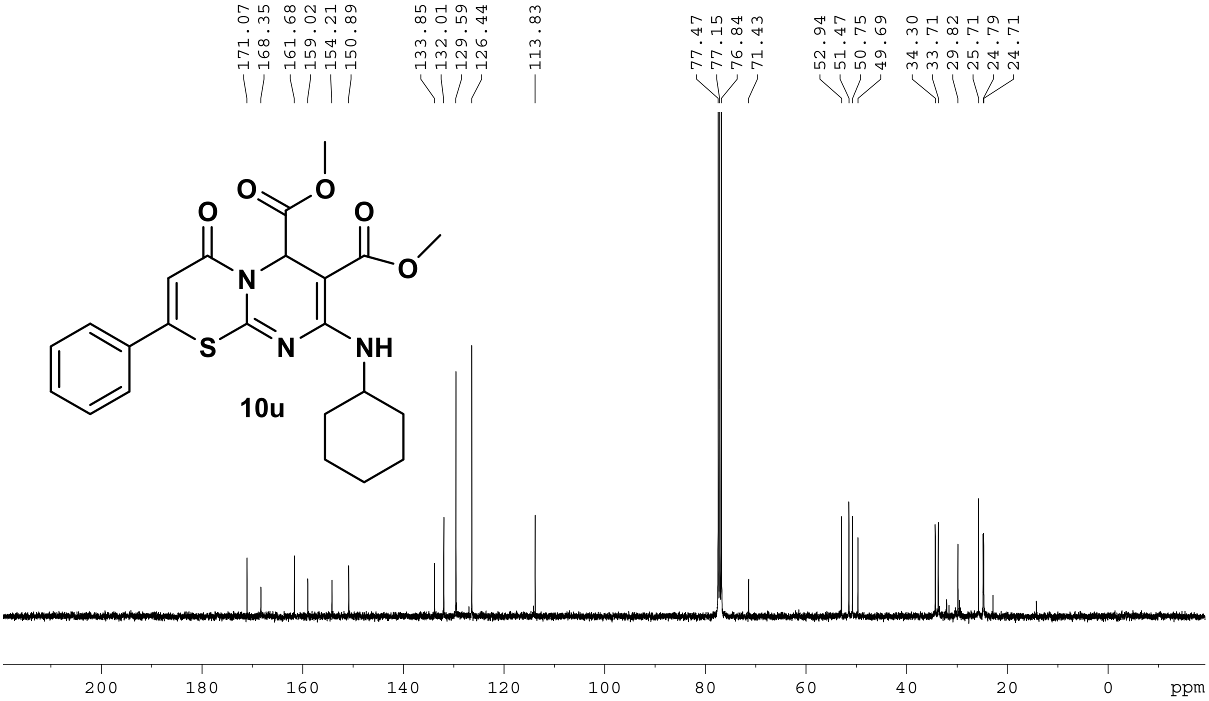
171.07
168.35
161.68
159.02
154.21
150.89
133.85
132.01
129.59
126.44
113.83
77.47
77.15
76.84
71.43
52.94
51.47
50.75
49.69
34.30
33.71
29.82
25.71
24.79
24.71
10u
200 180 160 140 120 100 80 60 40 20 0 ppm

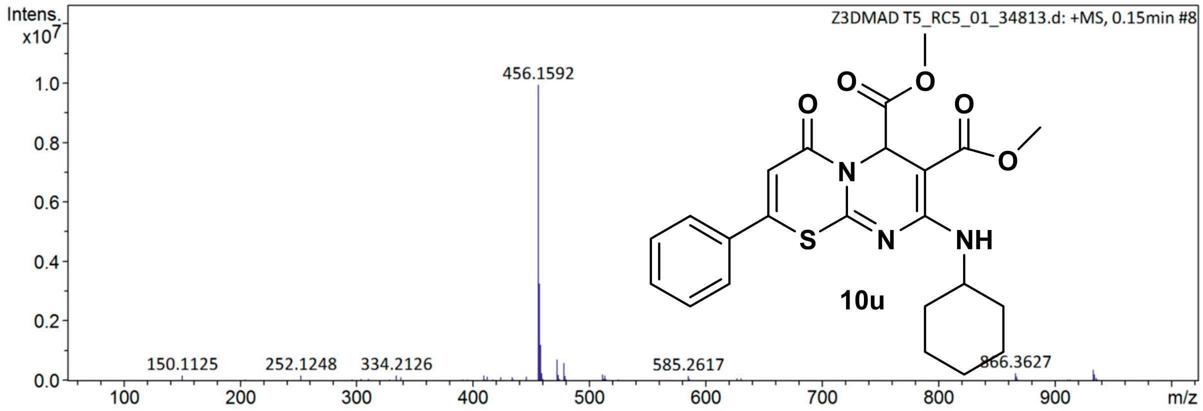
Intens.
x10^7
Z3DMAD T5_RC5_01_34813.d: +MS, 0.15min #8
456.1592
150.1125
252.1248
334.2126
585.2617
866.3627
10u
1.0
0.8
0.6
0.4
0.2
0.0
100 200 300 400 500 600 700 800 900 m/z

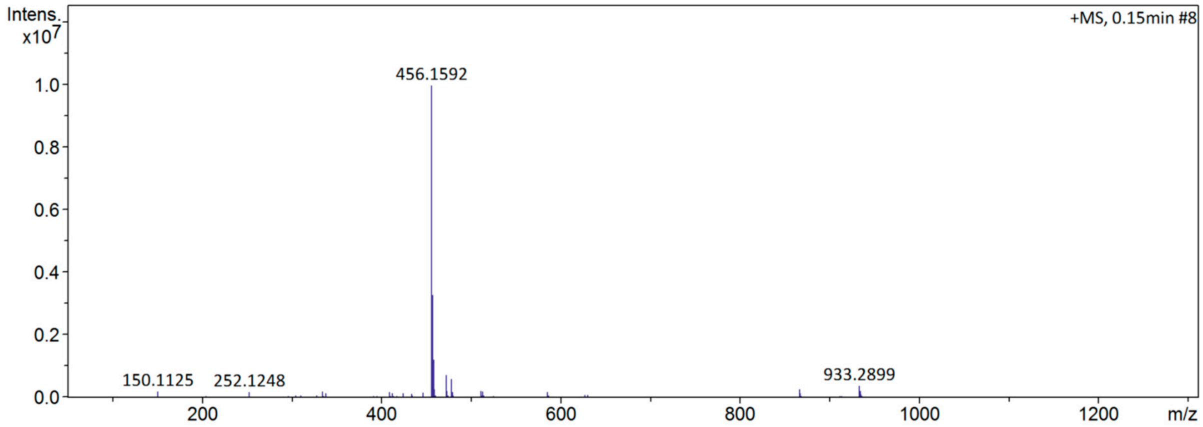
Intens.
x10^7
+MS, 0.15min #8
456.1592
150.1125
252.1248
933.2899
1.0
0.8
0.6
0.4
0.2
0.0
200 400 600 800 1000 1200 m/z

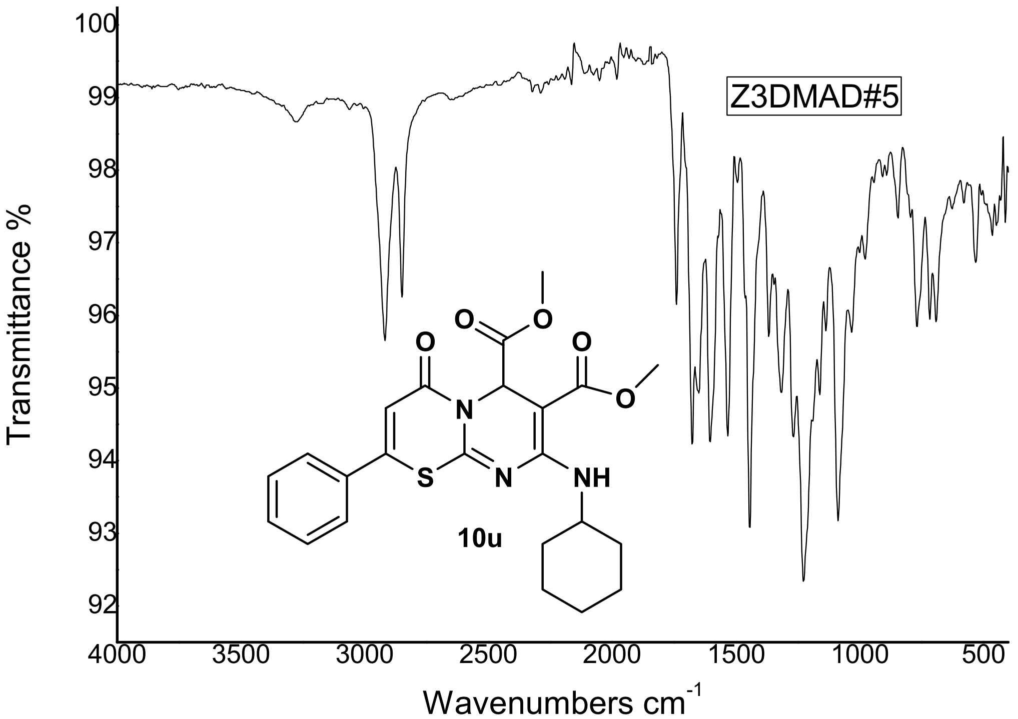
Z3DMAD#5
Transmittance %
Wavenumbers cm-1
10u
100
99
98
97
96
95
94
93
92
4000
3500
3000
2500
2000
1500
1000
500

Methyl 7-oxo-7*H*-thiazolo[3,2-*a*]pyrimidine-5-carboxylate derivatives and their analogs**.**

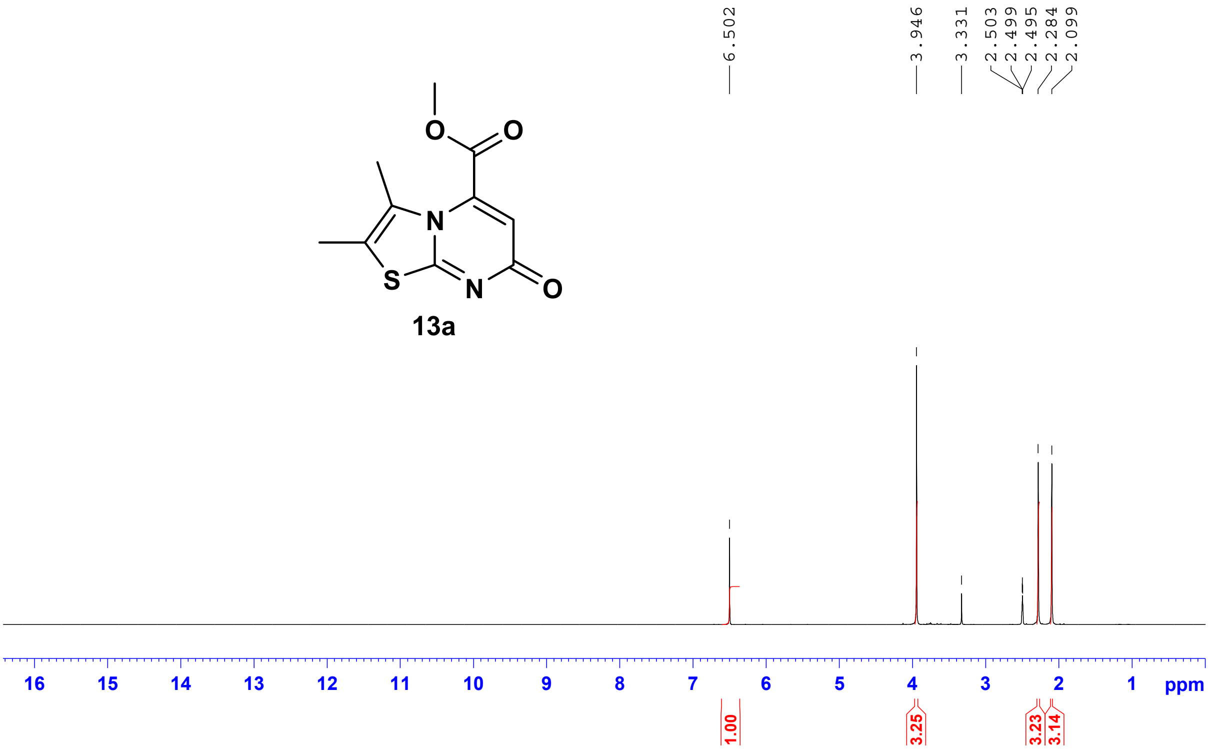

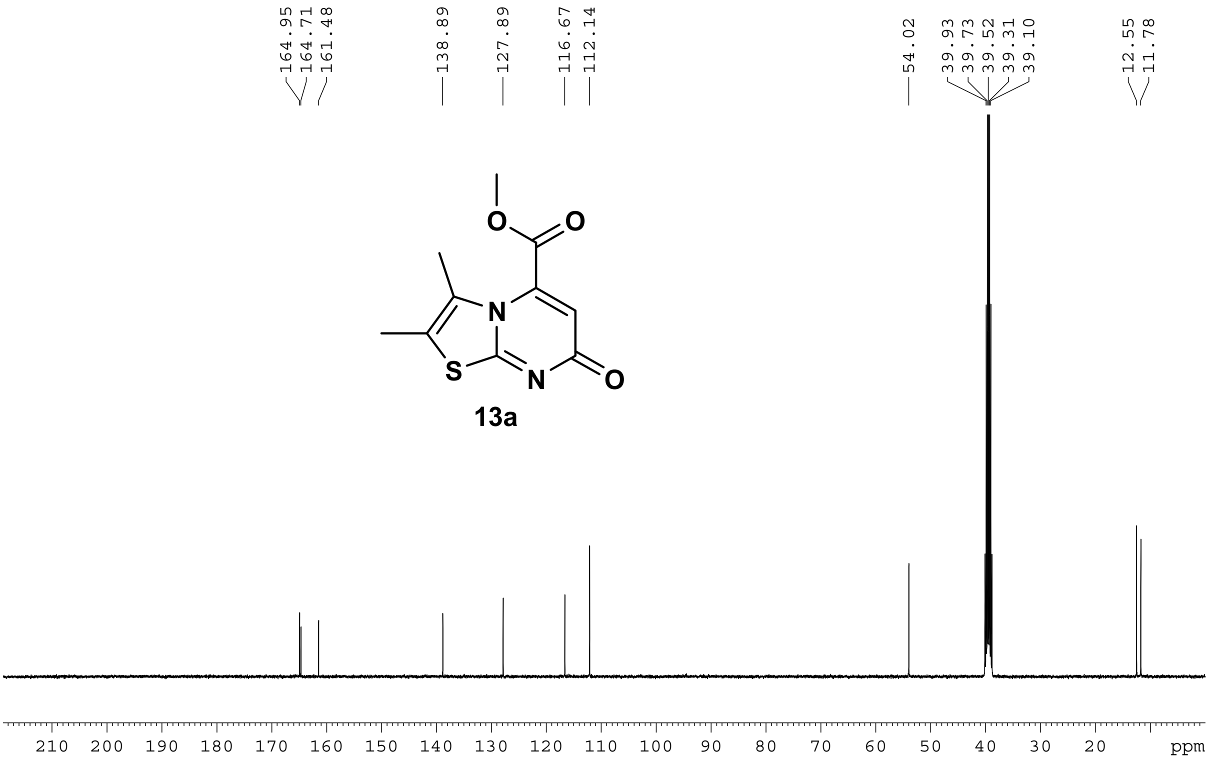

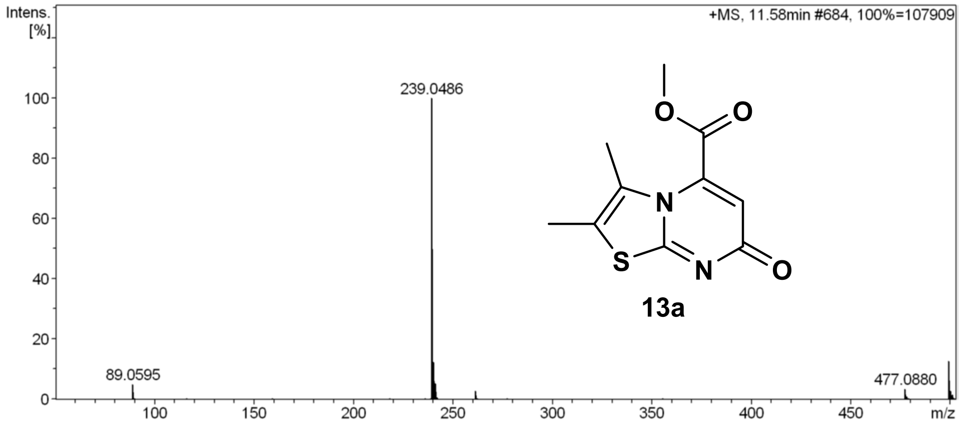
Intens.
[%]
+MS, 11.58min #684, 100%=107909
239.0486
89.0595
477.0880
13a
m/z

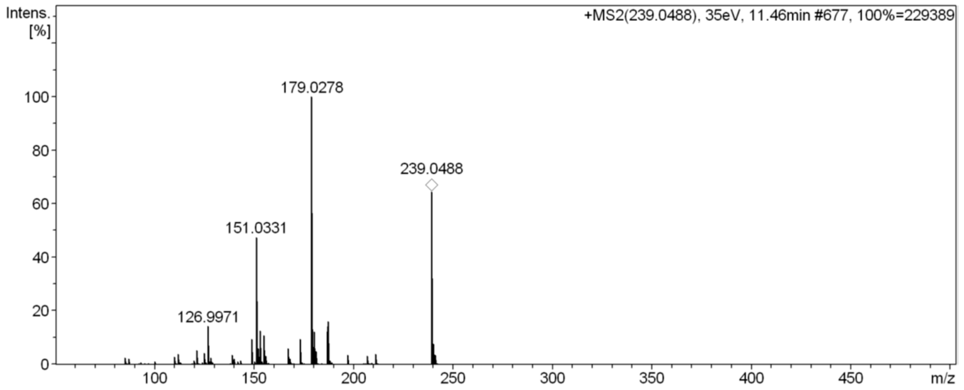
Intens.
[%]
+MS2(239.0488), 35eV, 11.46min #677, 100%=229389
179.0278
239.0488
151.0331
126.9971
m/z

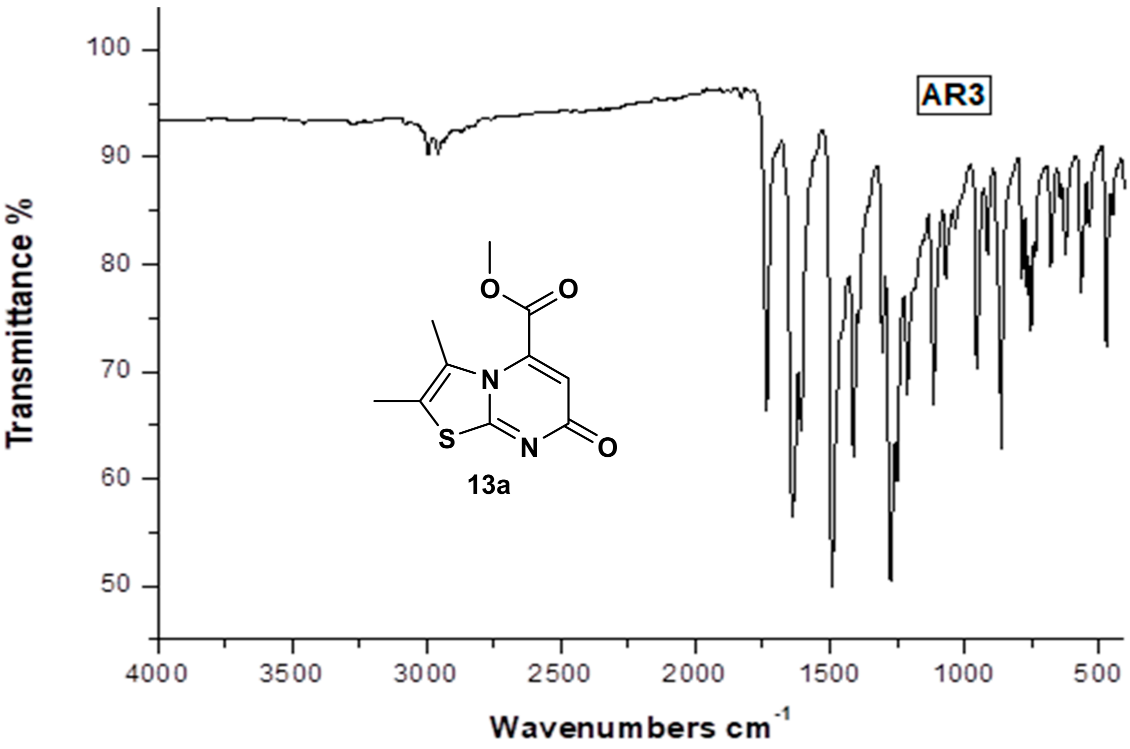
AR3
Transmittance %
13a
Wavenumbers cm-1

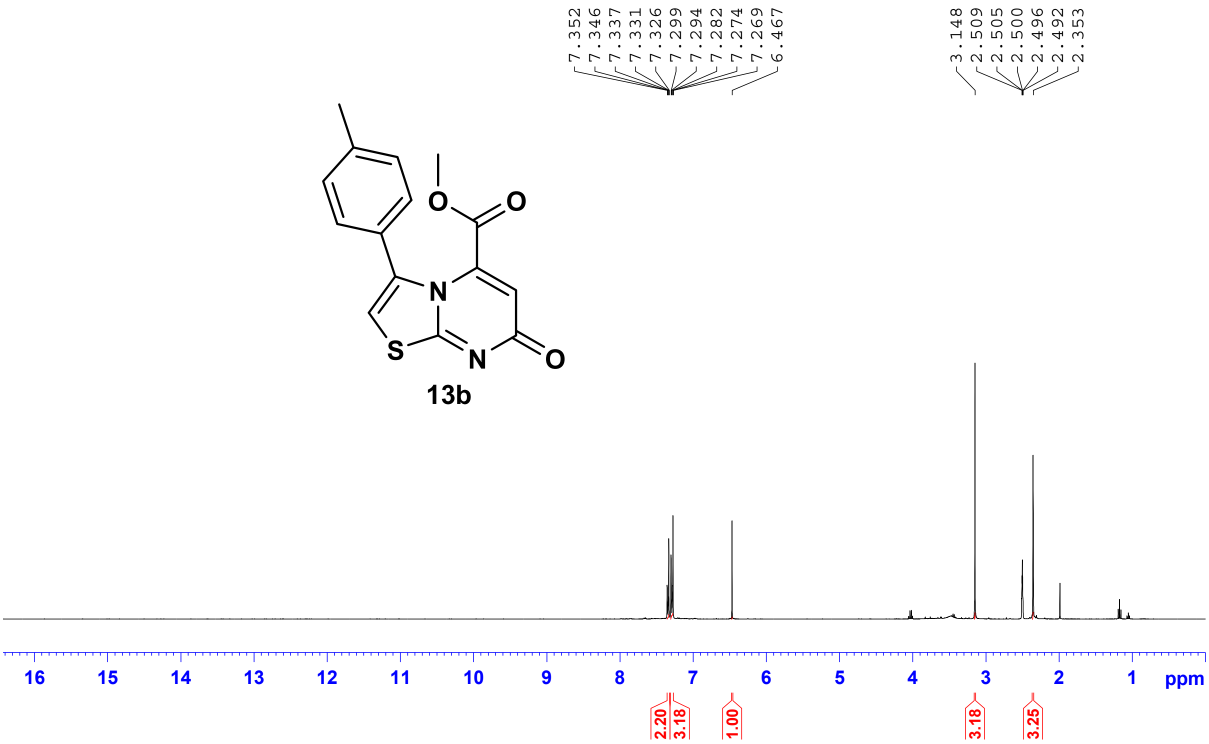
7.352
7.346
7.337
7.331
7.326
7.299
7.294
7.282
7.274
7.269
6.467
3.148
2.509
2.505
2.500
2.496
2.492
2.353
N
O
O
N
S
O
13b
16 15 14 13 12 11 10 9 8 7 6 5 4 3 2 1 ppm
2.20
3.18
1.00
3.18
3.25

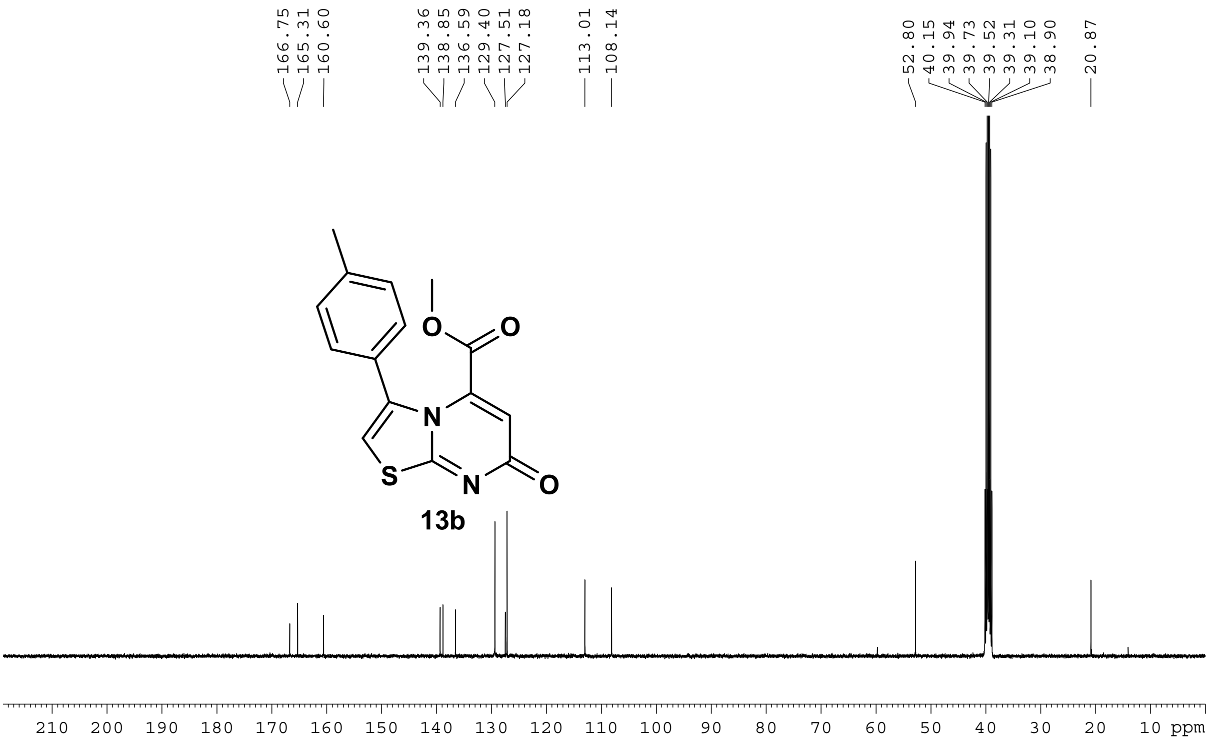
166.75
165.31
160.60
139.36
138.85
136.59
129.40
127.51
127.18
113.01
108.14
52.80
40.15
39.94
39.73
39.52
39.31
39.10
38.90
20.87
N
O
O
N
S
O
13b
210 200 190 180 170 160 150 140 130 120 110 100 90 80 70 60 50 40 30 20 10 ppm

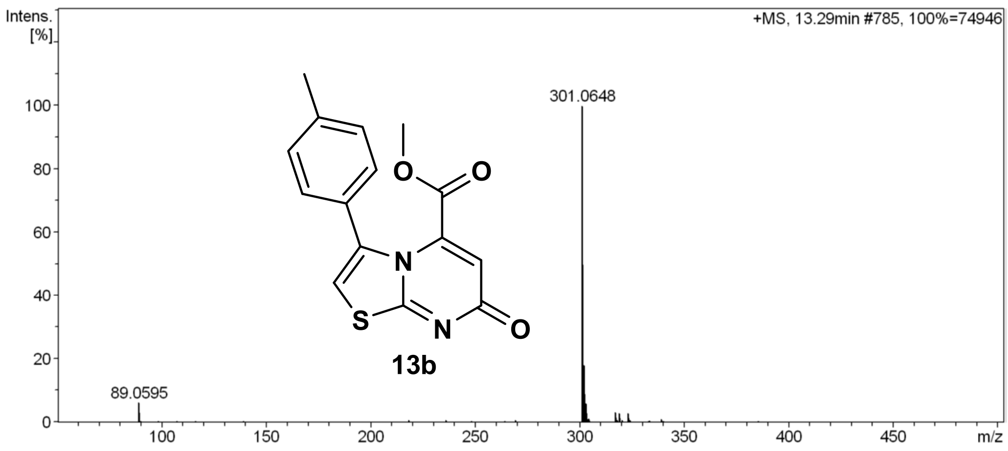
Intens. [%]
+MS, 13.29min #785, 100%=74946
301.0648
89.0595
13b
m/z

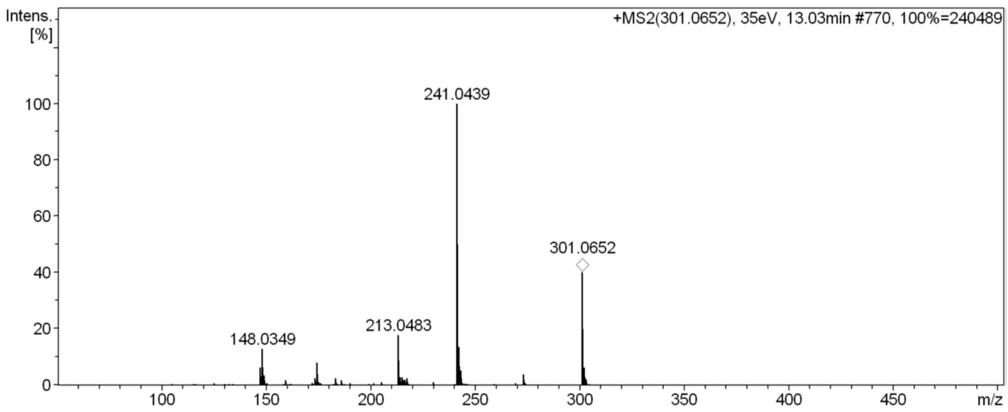
Intens. [%]
+MS2(301.0652), 35eV, 13.03min #770, 100%=240489
241.0439
301.0652
213.0483
148.0349
m/z

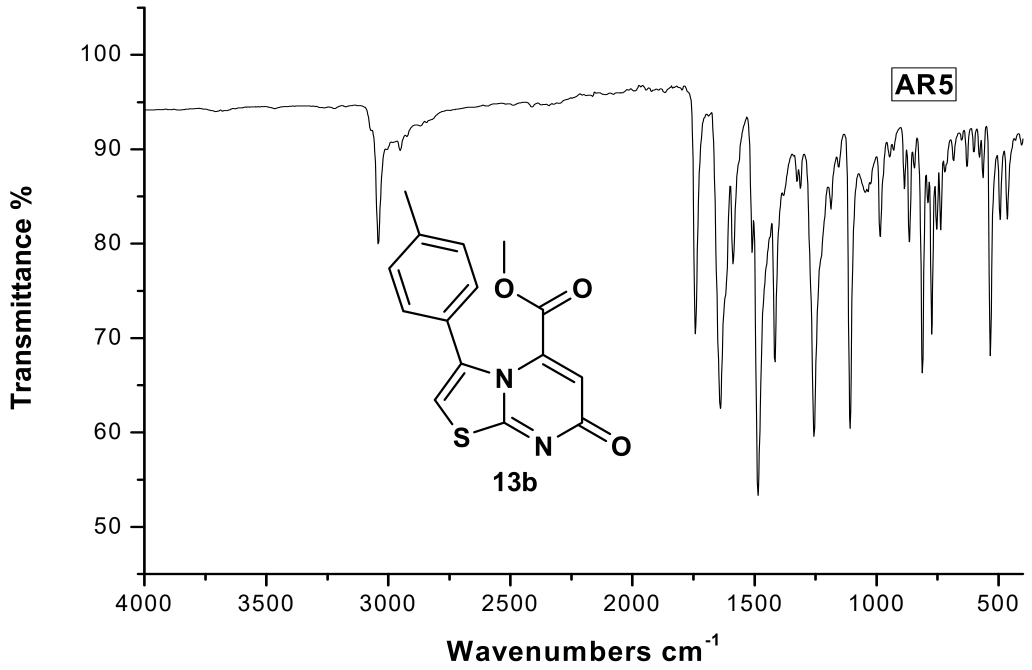
AR5
Transmittance %
13b
Wavenumbers cm-1

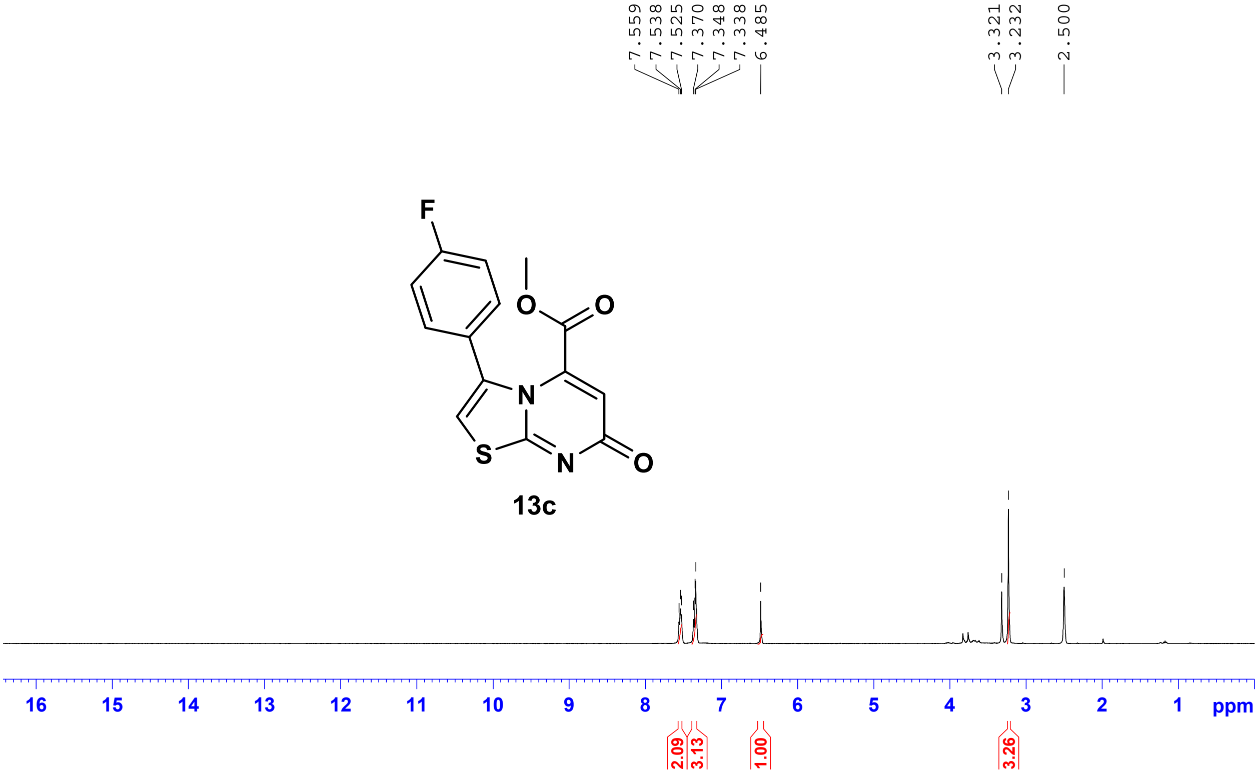
7.559
7.538
7.525
7.370
7.348
7.338
6.485
3.321
3.232
2.500
F
O
O
N
S
N
O
13c
16 15 14 13 12 11 10 9 8 7 6 5 4 3 2 1 ppm
2.09
3.13
1.00
3.26

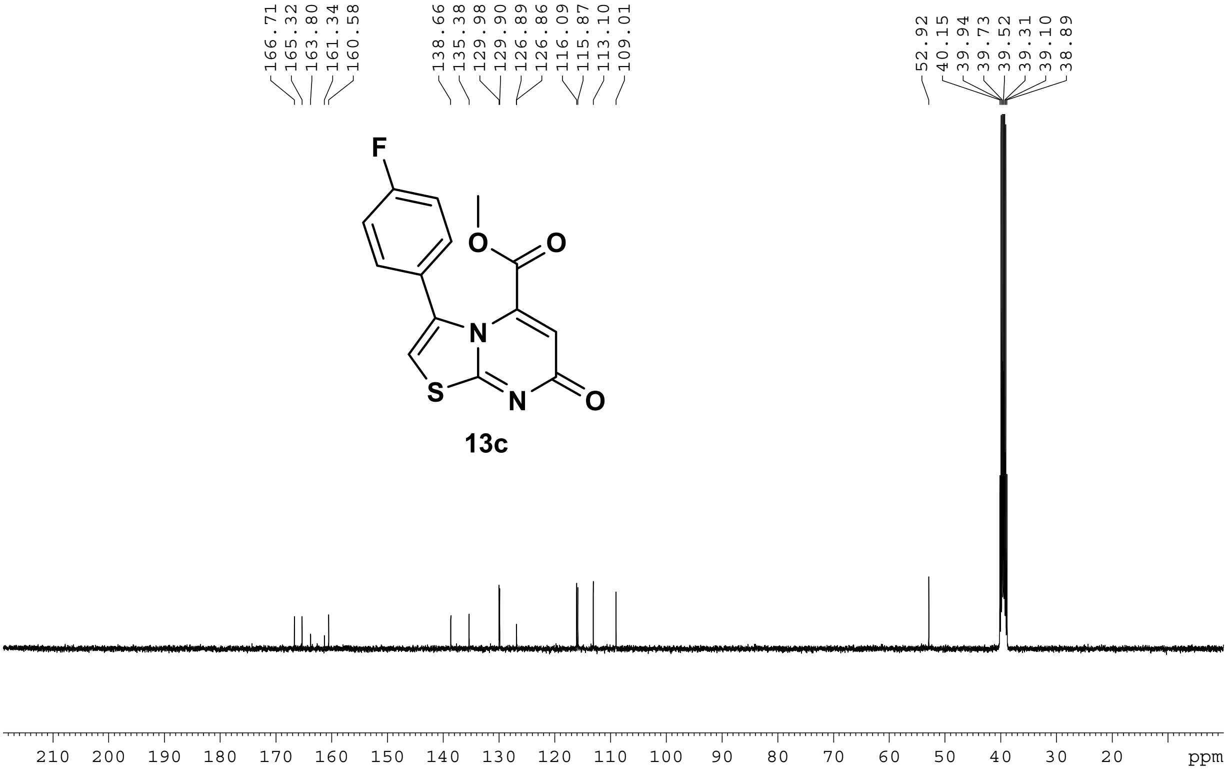
166.71
165.32
163.80
161.34
160.58
138.66
135.38
129.98
129.90
126.89
126.86
116.09
115.87
113.10
109.01
52.92
40.15
39.94
39.73
39.52
39.31
39.10
38.89
F
O
O
N
S
N
O
13c
210 200 190 180 170 160 150 140 130 120 110 100 90 80 70 60 50 40 30 20 ppm

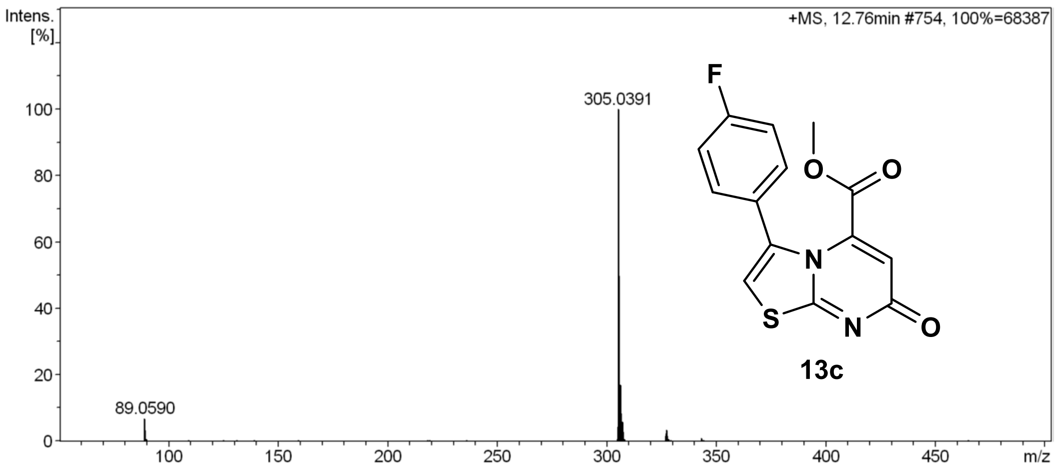

Intens. [%]
+MS, 12.76min #754, 100%=68387
305.0391
89.0590
m/z
13c

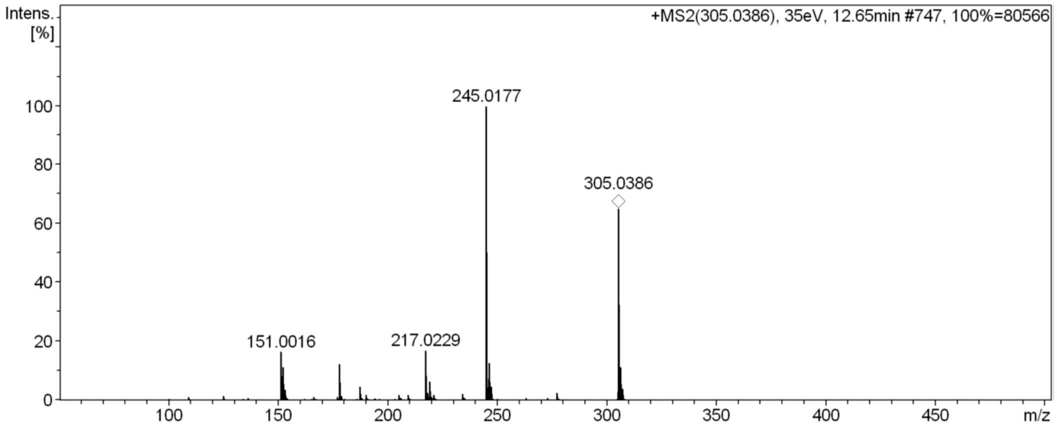

Intens. [%]
+MS2(305.0386), 35eV, 12.65min #747, 100%=80566
245.0177
305.0386
151.0016
217.0229
m/z

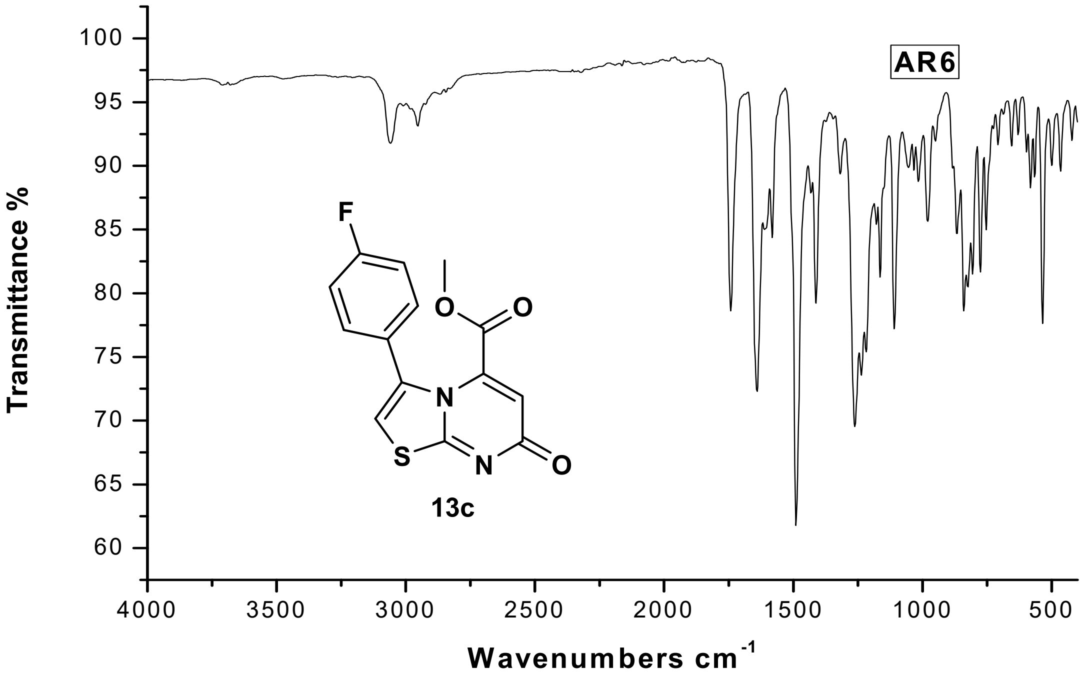

AR6
Transmittance %
Wavenumbers cm-1
13c

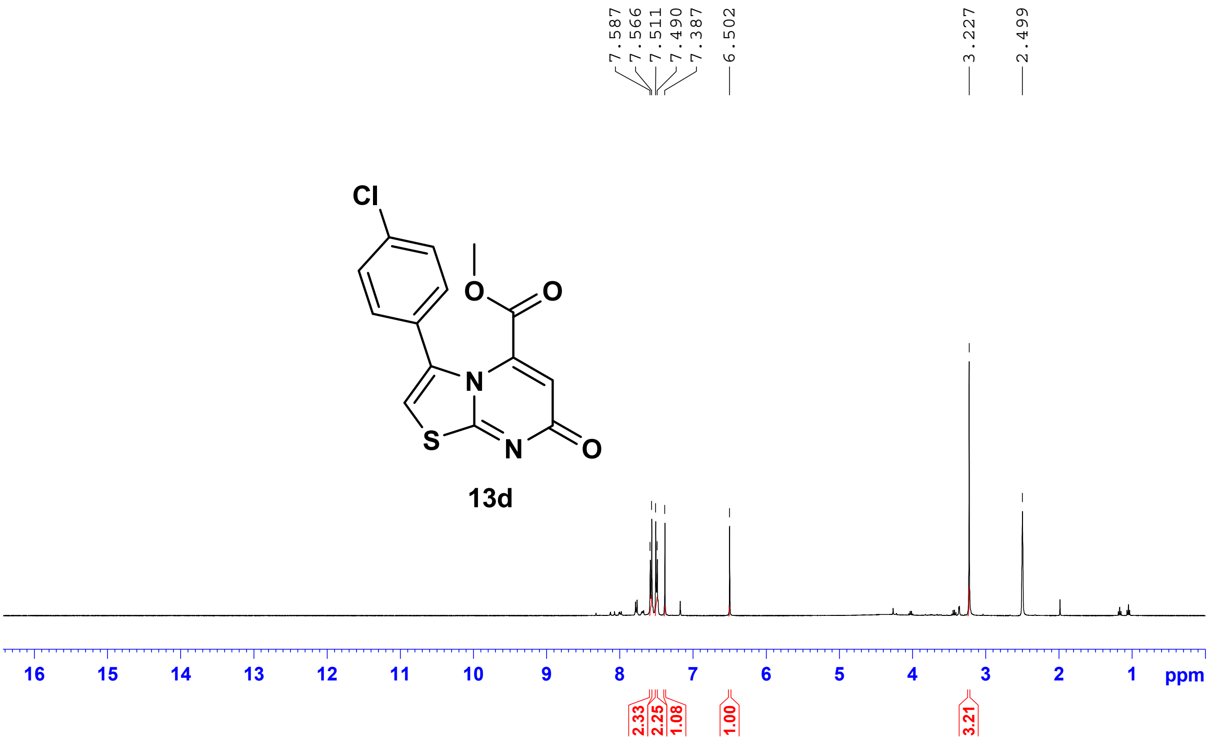
7.587
7.566
7.511
7.490
7.387
6.502
3.227
2.499
Cl
O
O
N
S
N
O
13d
16 15 14 13 12 11 10 9 8 7 6 5 4 3 2 1 ppm
2.33
2.25
1.08
1.00
3.21

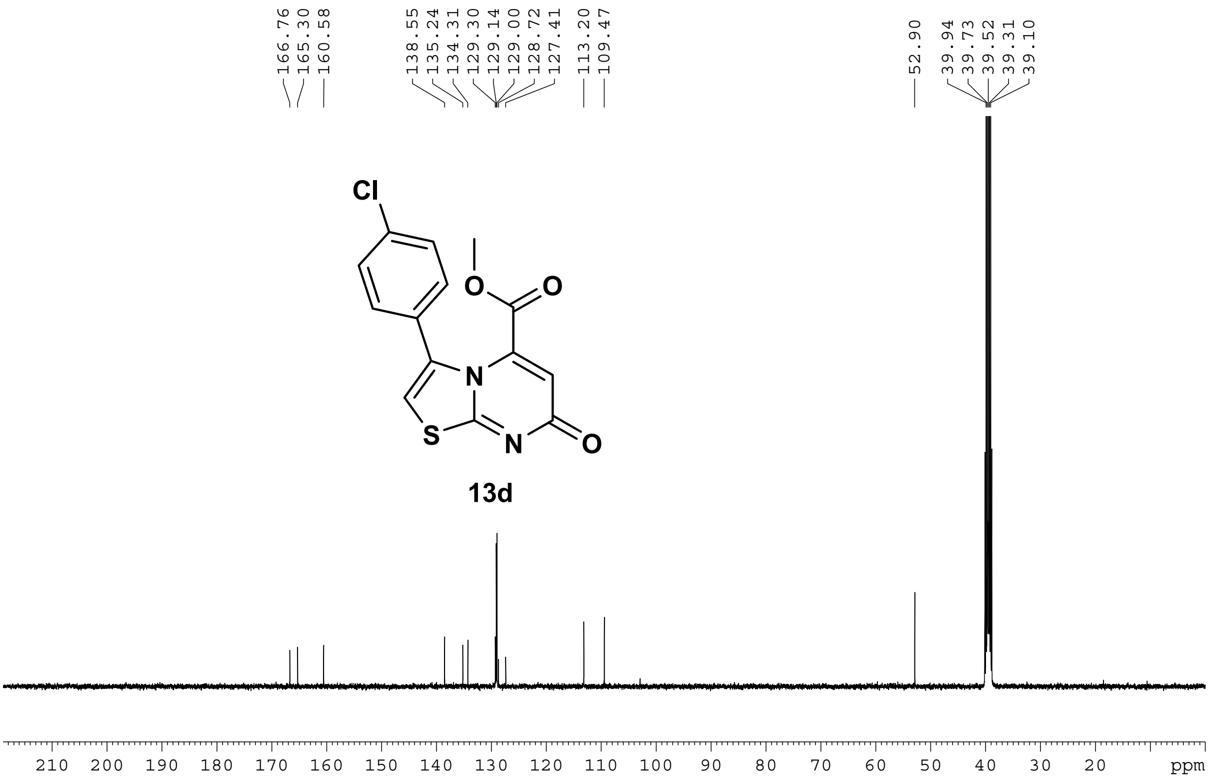
166.76
165.30
160.58
138.55
135.24
134.31
129.30
129.14
129.00
128.72
127.41
113.20
109.47
52.90
39.94
39.73
39.52
39.31
39.10
Cl
O
O
N
S
N
O
13d
210 200 190 180 170 160 150 140 130 120 110 100 90 80 70 60 50 40 30 20 ppm

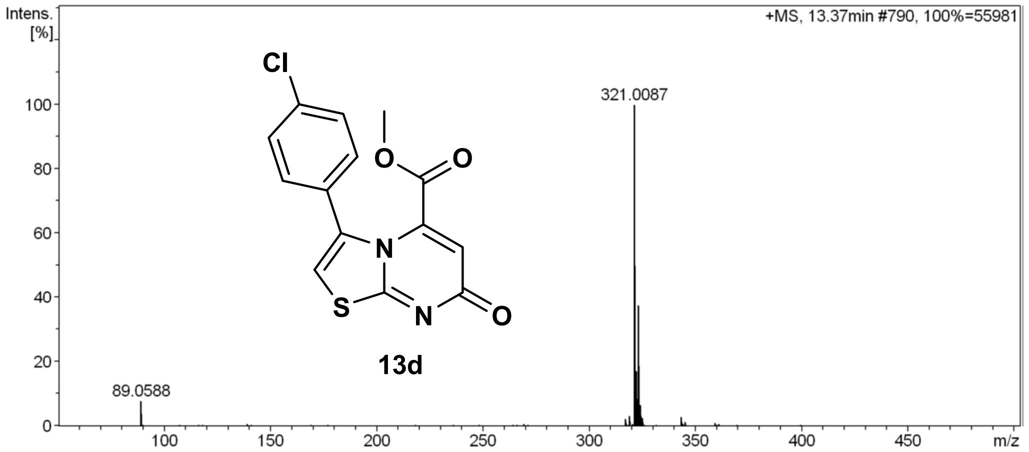
Intens.
[%]
+MS, 13.37min #790, 100%=55981
Cl
O
O
N
S
N
O
13d
321.0087
89.0588
m/z

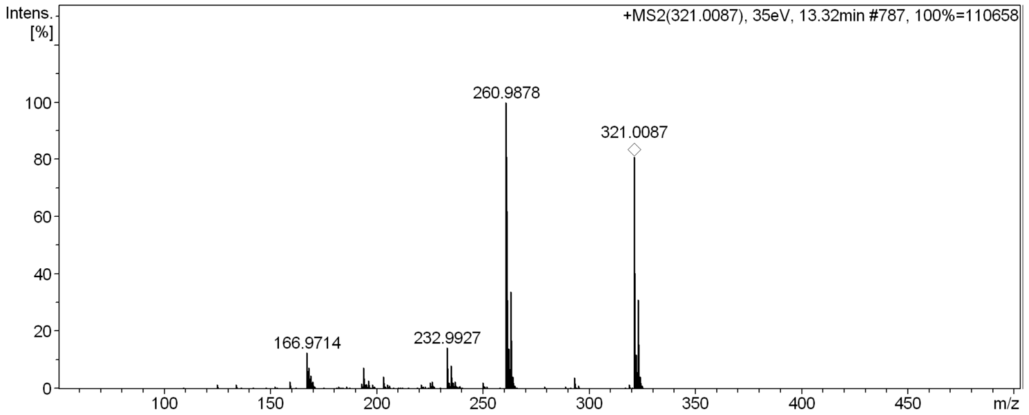
Intens.
[%]
+MS2(321.0087), 35eV, 13.32min #787, 100%=110658
260.9878
321.0087
166.9714
232.9927
m/z

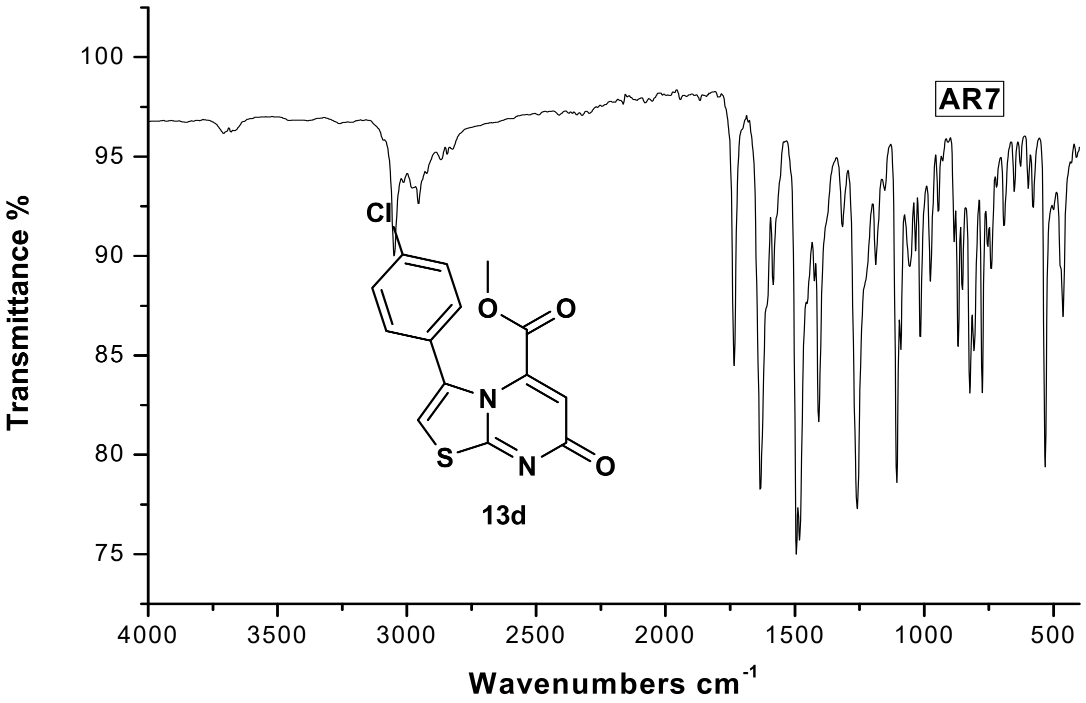
AR7
Transmittance %
Wavenumbers cm-1
Cl
O
O
N
S
N
O
13d

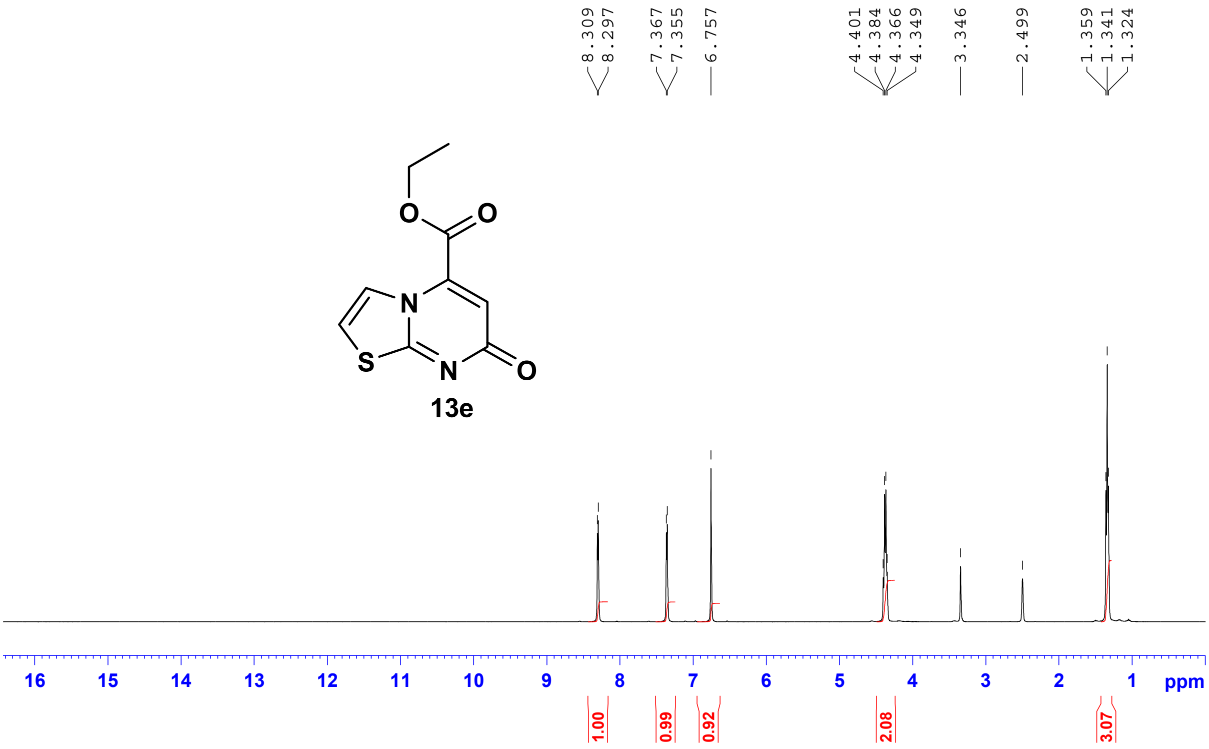
8.309
8.297
7.367
7.355
6.757
4.401
4.384
4.366
4.349
3.346
2.499
1.359
1.341
1.324
O
O
N
S
N
O
13e
16 15 14 13 12 11 10 9 8 7 6 5 4 3 2 1 ppm
1.00
0.99
0.92
2.08
3.07

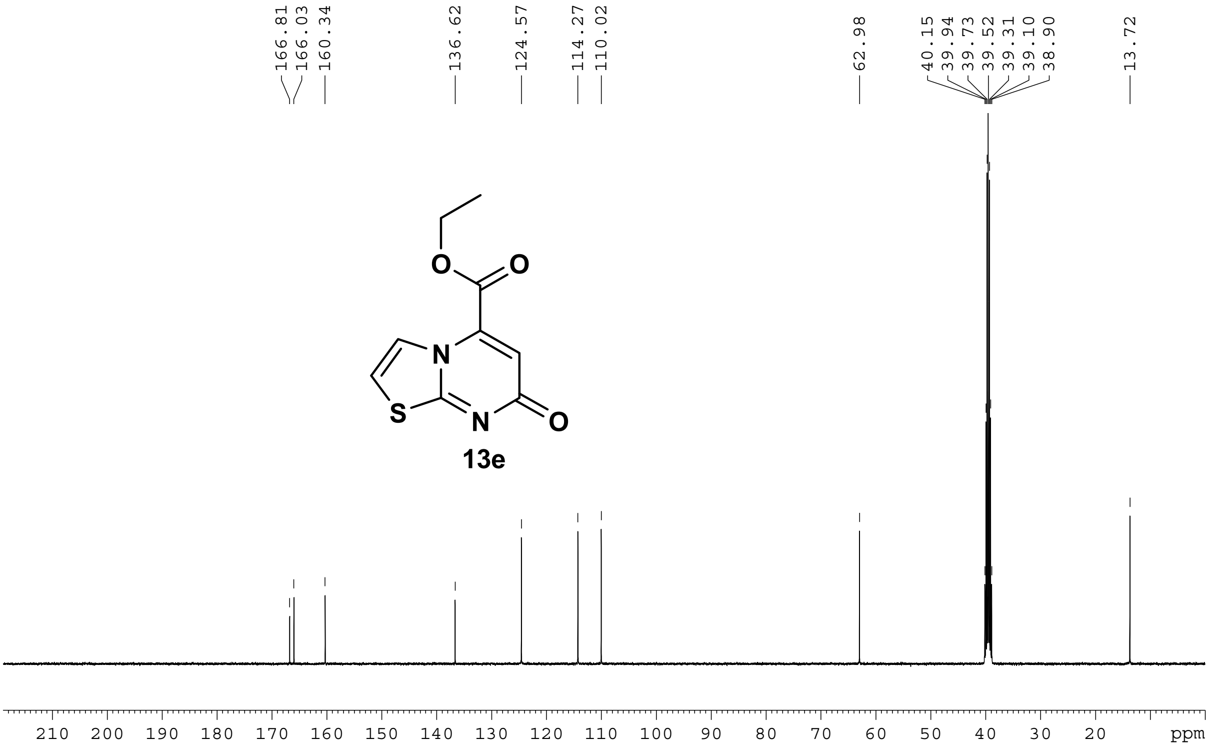
166.81
166.03
160.34
136.62
124.57
114.27
110.02
62.98
40.15
39.94
39.73
39.52
39.31
39.10
38.90
13.72
O
O
N
S
N
O
13e
210 200 190 180 170 160 150 140 130 120 110 100 90 80 70 60 50 40 30 20 ppm

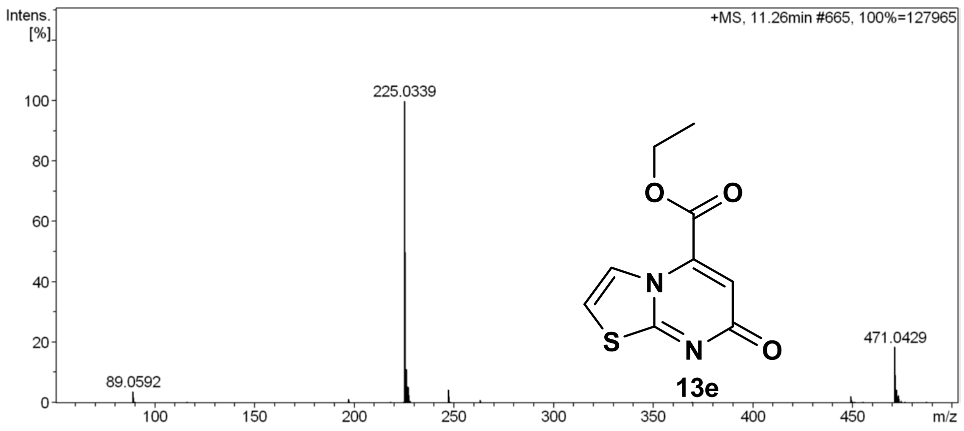
Intens.
[%]
+MS, 11.26min #665, 100%=127965
225.0339
89.0592
471.0429
m/z
13e

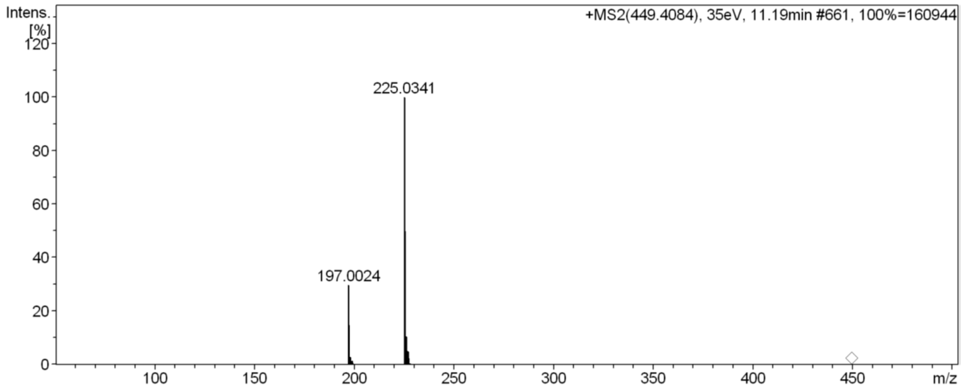
Intens.
[%]
+MS2(449.4084), 35eV, 11.19min #661, 100%=160944
225.0341
197.0024
m/z

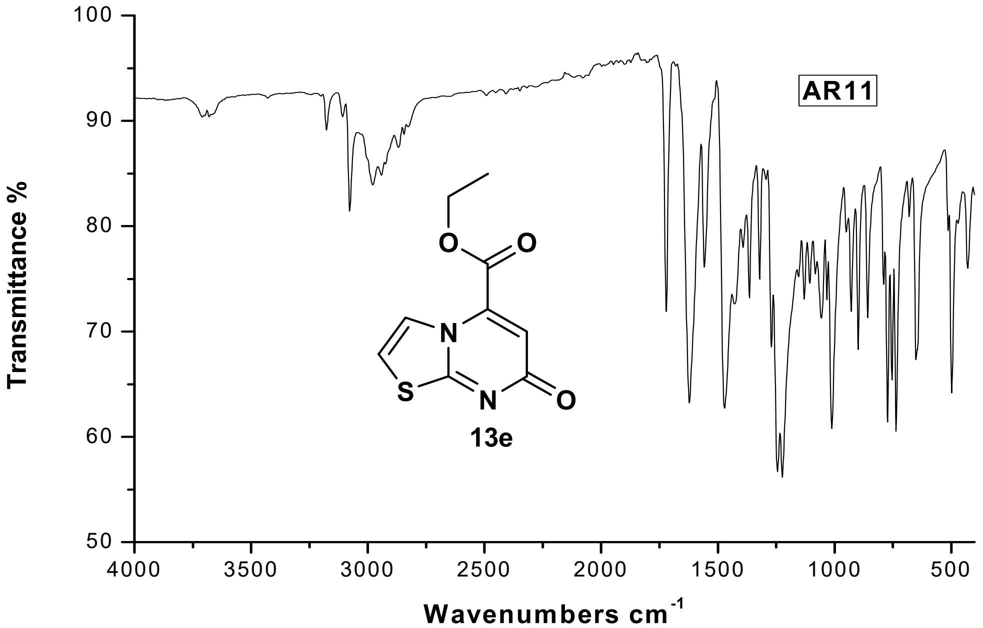
AR11
Transmittance %
Wavenumbers cm-1
13e

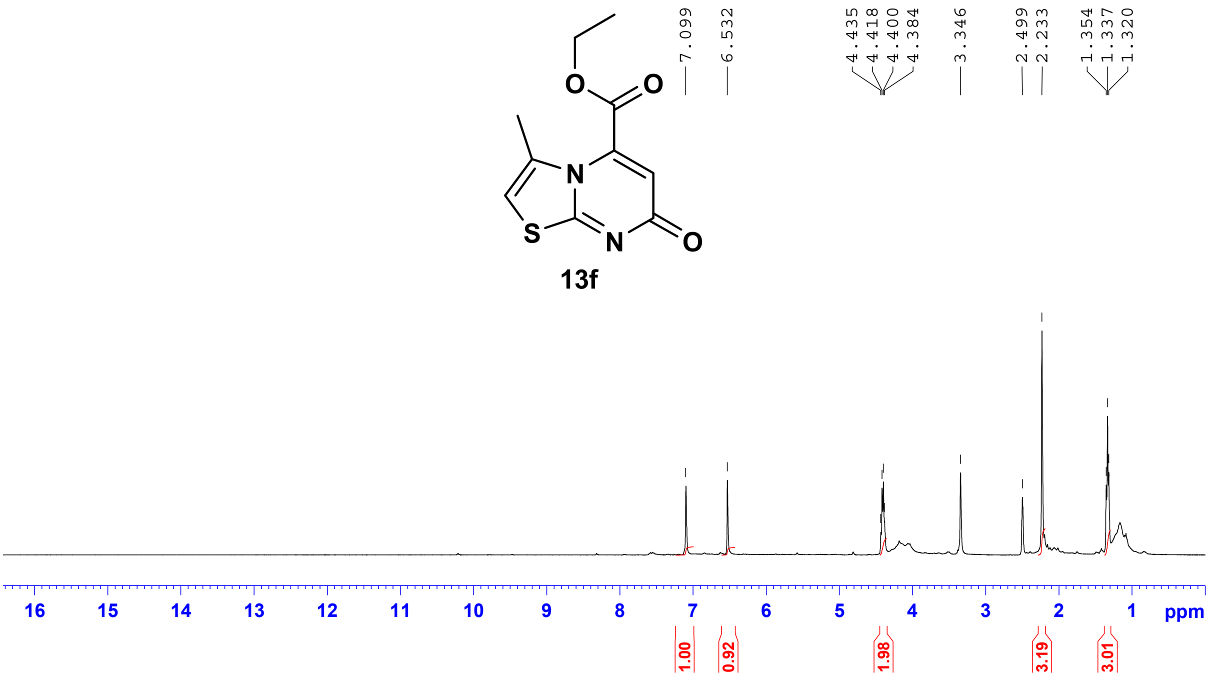

7.099
6.532
4.435
4.418
4.400
4.384
3.346
2.499
2.233
1.354
1.337
1.320
13f
16 15 14 13 12 11 10 9 8 7 6 5 4 3 2 1 ppm
1.00
0.92
1.98
3.19
3.01

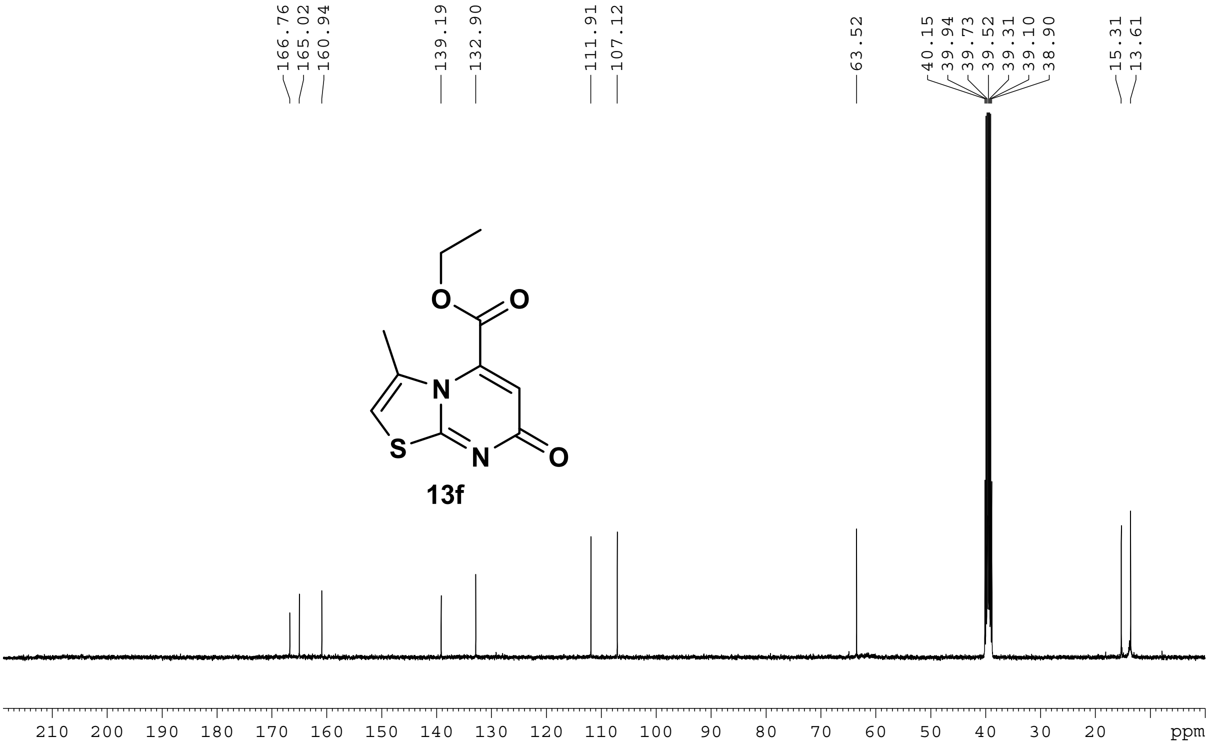

166.76
165.02
160.94
139.19
132.90
111.91
107.12
63.52
40.15
39.94
39.73
39.52
39.31
39.10
38.90
15.31
13.61
13f
210 200 190 180 170 160 150 140 130 120 110 100 90 80 70 60 50 40 30 20 ppm

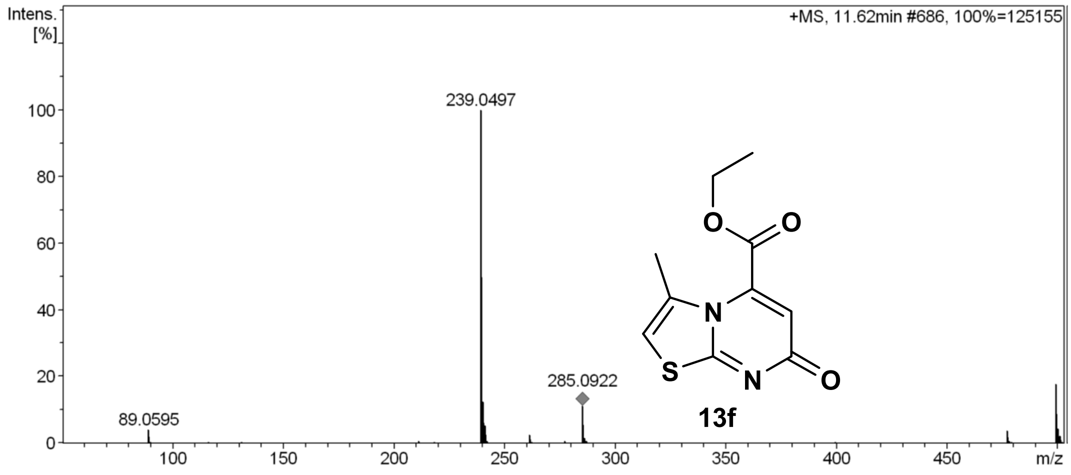
Intens. [%]
+MS, 11.62min #686, 100%=125155
239.0497
285.0922
89.0595
13f
m/z

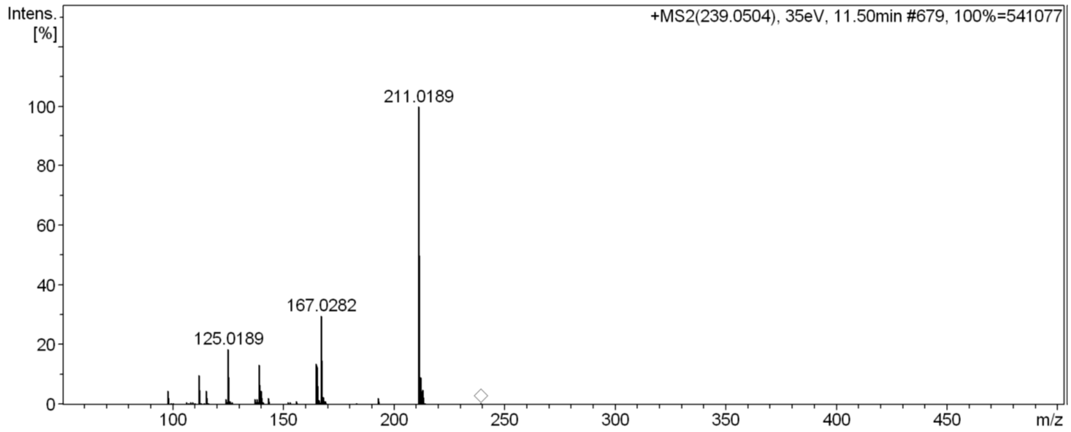
Intens. [%]
+MS2(239.0504), 35eV, 11.50min #679, 100%=541077
211.0189
167.0282
125.0189
m/z

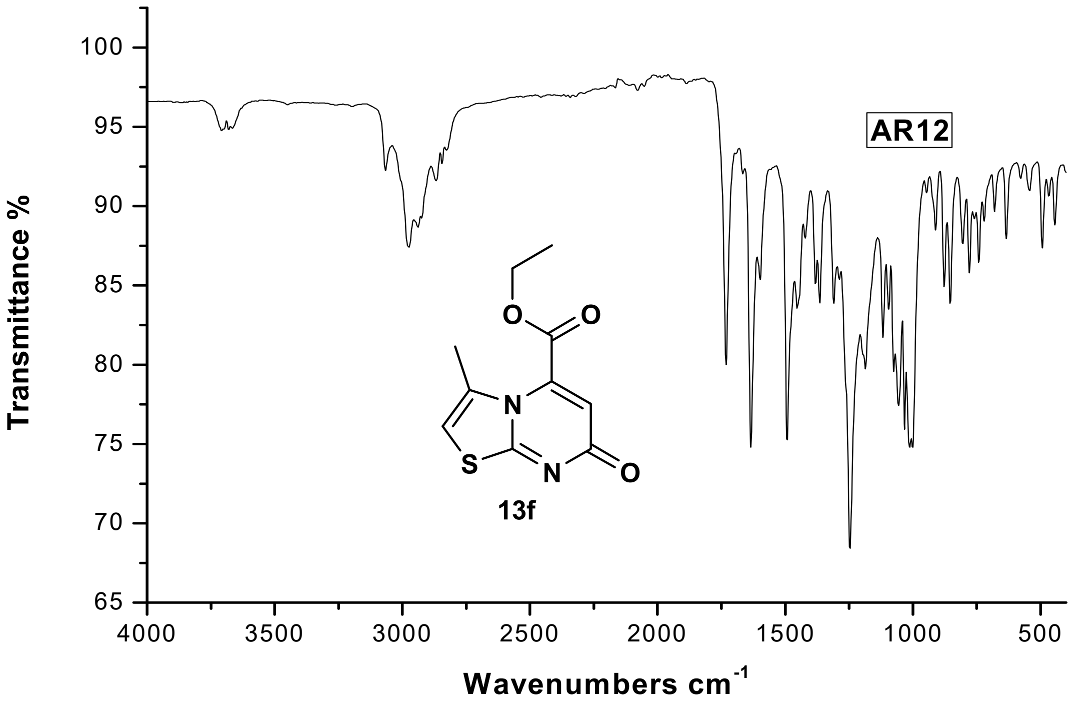
AR12
Transmittance %
Wavenumbers cm-1
13f

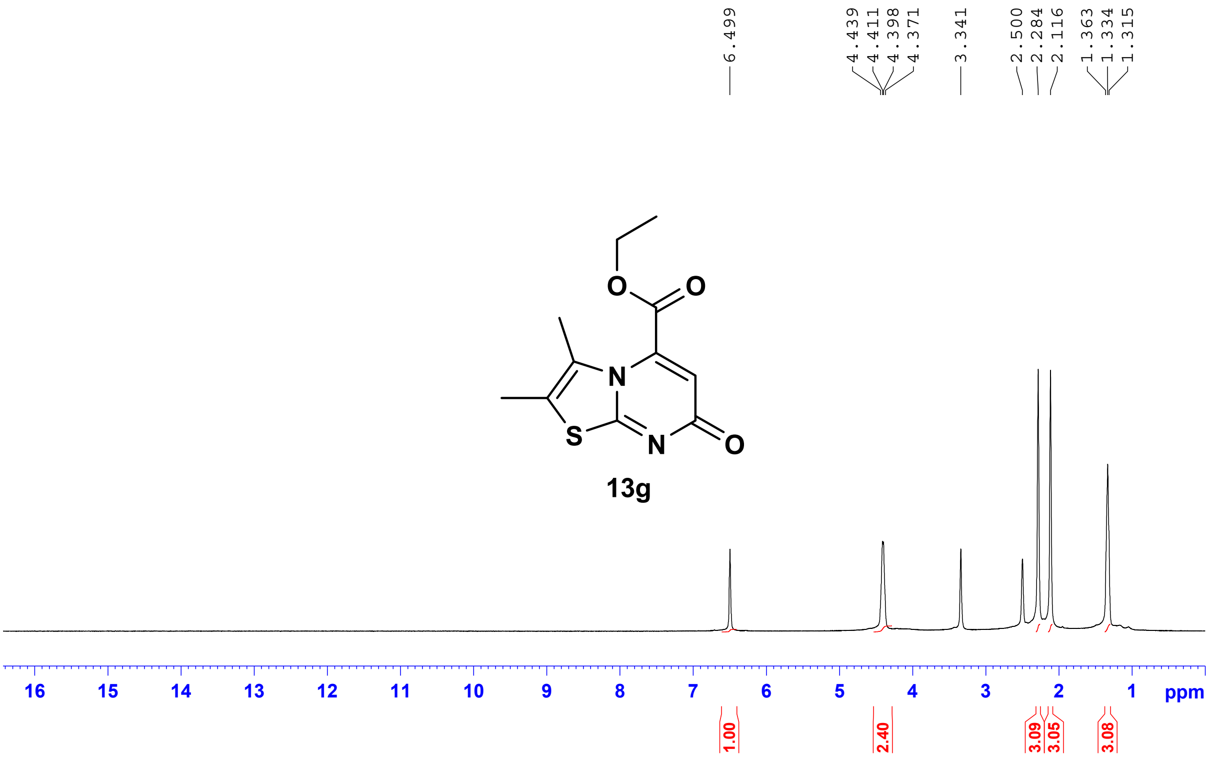
6.499
4.439
4.411
4.398
4.371
3.341
2.500
2.284
2.116
1.363
1.334
1.315
O
O
N
S
N
O
13g
16 15 14 13 12 11 10 9 8 7 6 5 4 3 2 1 ppm
1.00
2.40
3.09
3.05
3.08

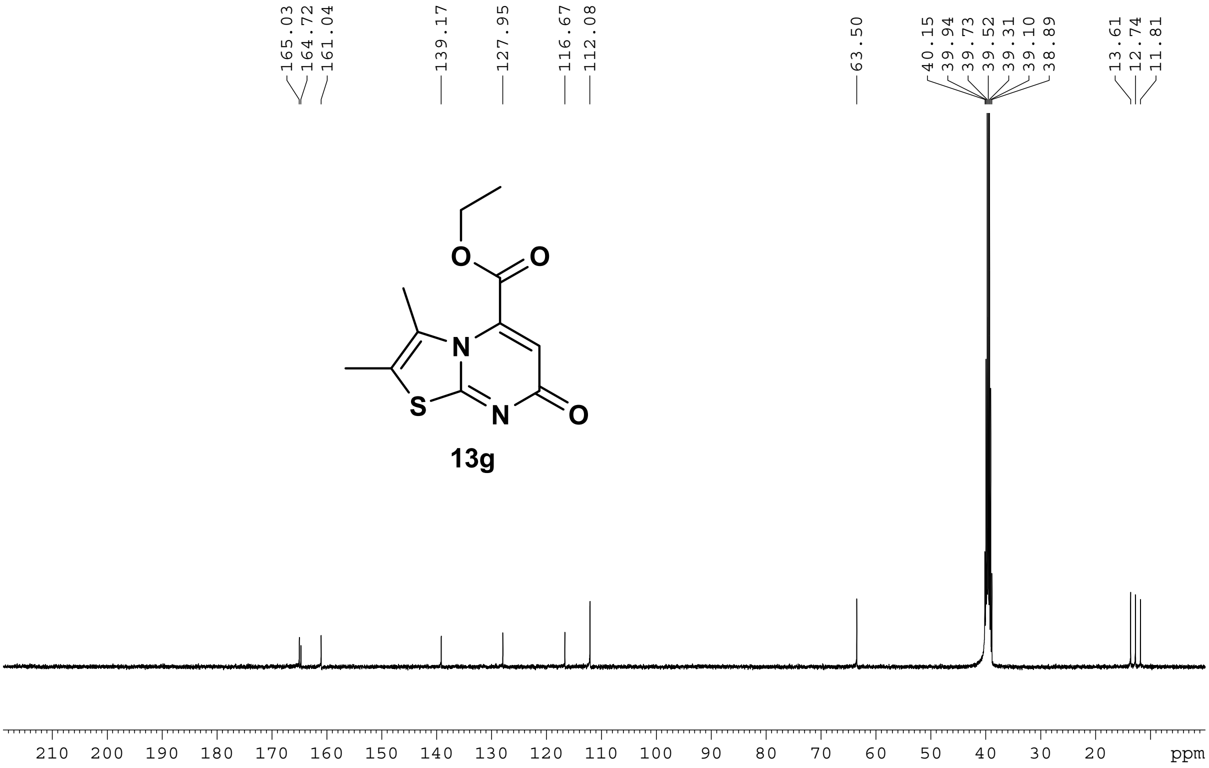
165.03
164.72
161.04
139.17
127.95
116.67
112.08
63.50
40.15
39.94
39.73
39.52
39.31
39.10
38.89
13.61
12.74
11.81
O
O
N
S
N
O
13g
210 200 190 180 170 160 150 140 130 120 110 100 90 80 70 60 50 40 30 20 ppm

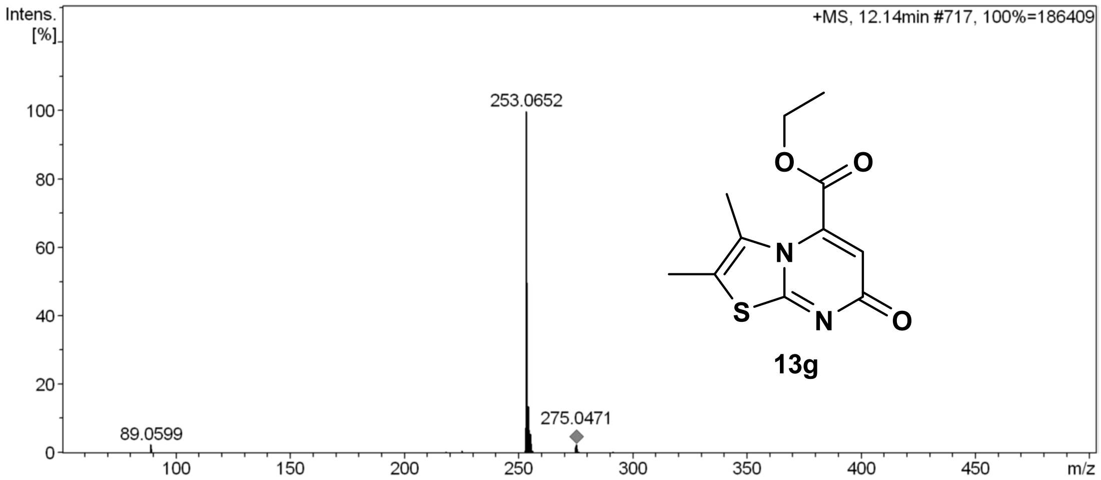
Intens. [%]
+MS, 12.14min #717, 100%=186409
253.0652
275.0471
89.0599
m/z
O
O
N
S
N
O
13g

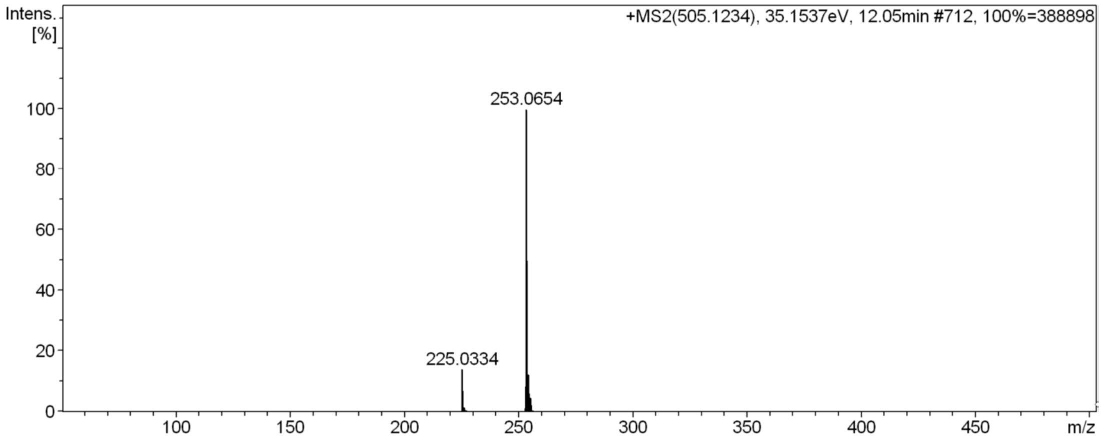
Intens. [%]
+MS2(505.1234), 35.1537eV, 12.05min #712, 100%=388898
253.0654
225.0334
m/z

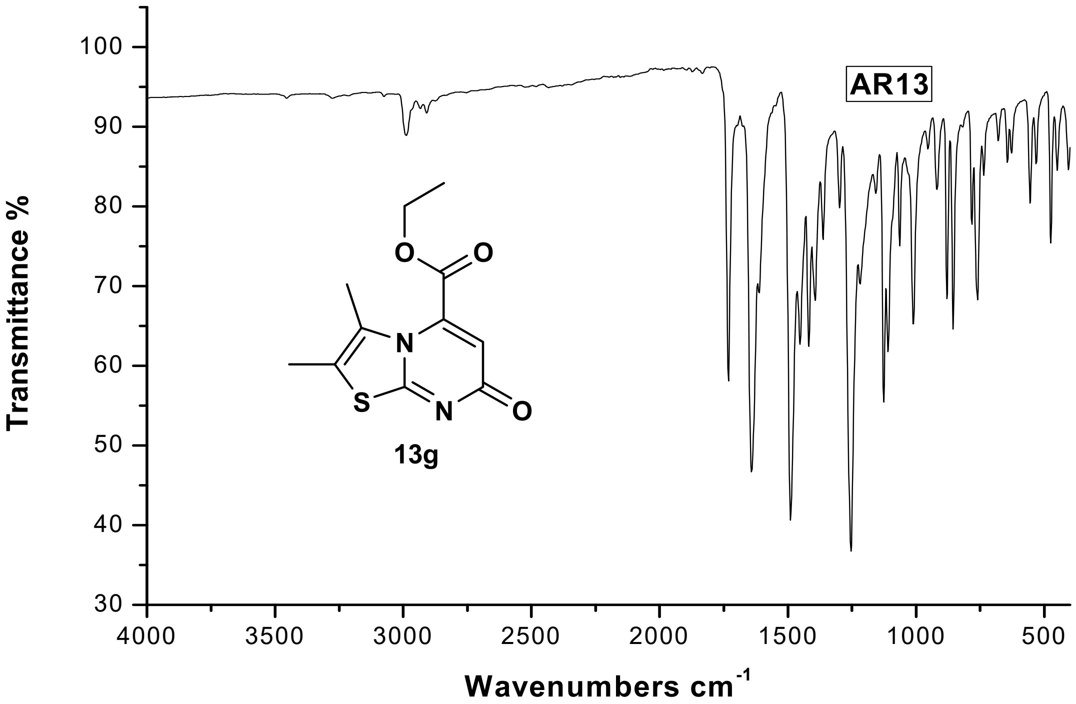
AR13
Transmittance %
Wavenumbers cm-1
O
O
N
S
N
O
13g

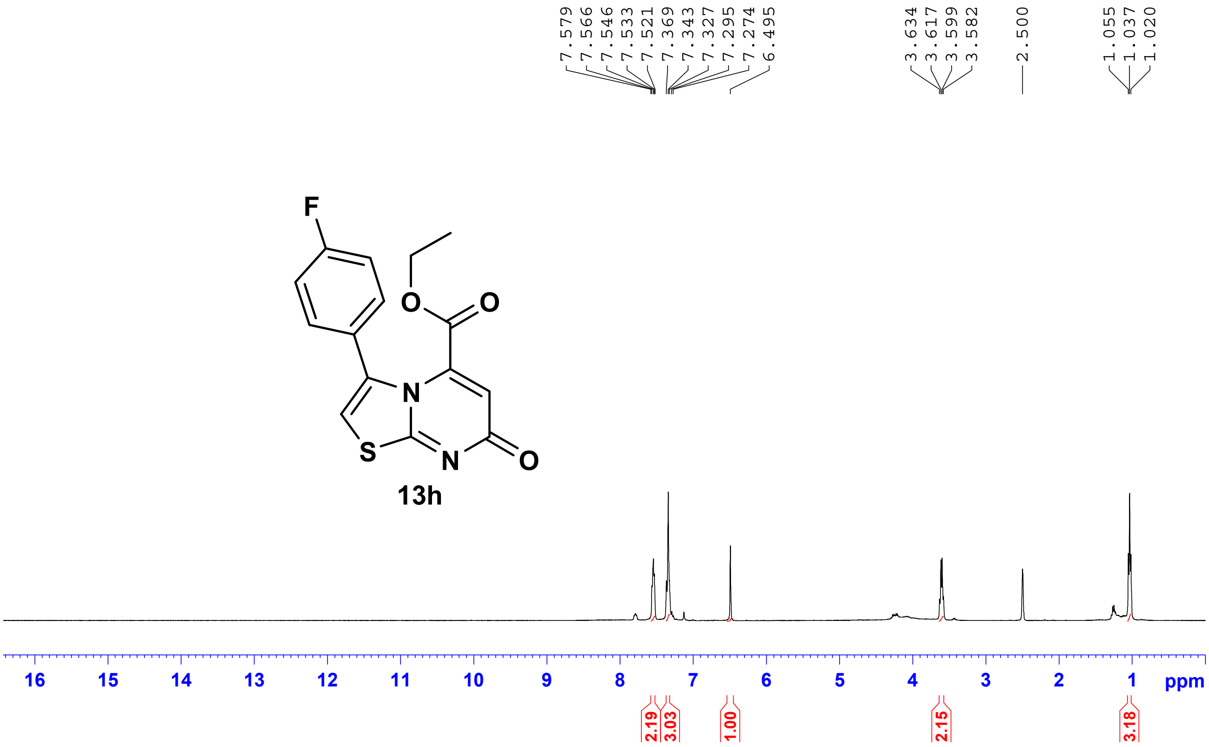
7.579
7.566
7.546
7.533
7.521
7.369
7.343
7.327
7.295
7.274
6.495
3.634
3.617
3.599
3.582
2.500
1.055
1.037
1.020
F
O
O
N
S
N
O
13h
16 15 14 13 12 11 10 9 8 7 6 5 4 3 2 1 ppm
2.19
3.03
1.00
2.15
3.18

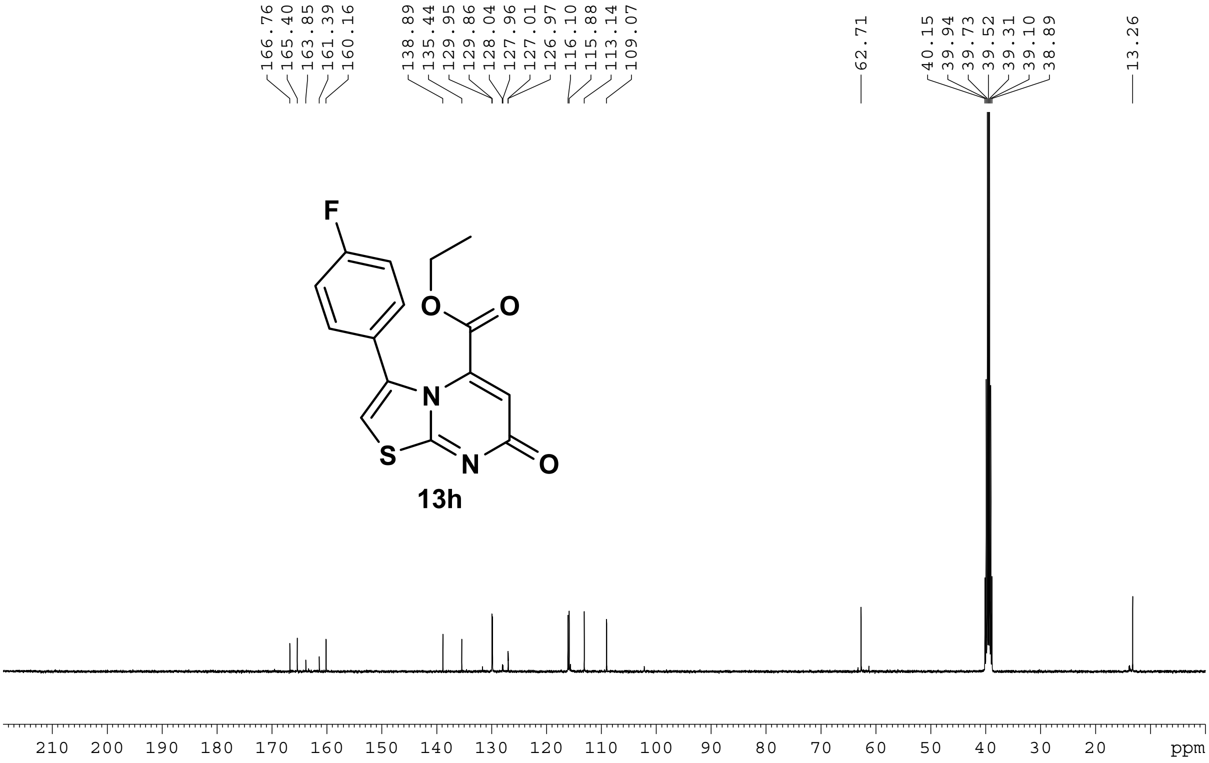
166.76
165.40
163.85
161.39
160.16
138.89
135.44
129.95
129.86
128.04
127.96
127.01
126.97
116.10
115.88
113.14
109.07
62.71
40.15
39.94
39.73
39.52
39.31
39.10
38.89
13.26
F
O
O
N
S
N
O
13h
210 200 190 180 170 160 150 140 130 120 110 100 90 80 70 60 50 40 30 20 ppm

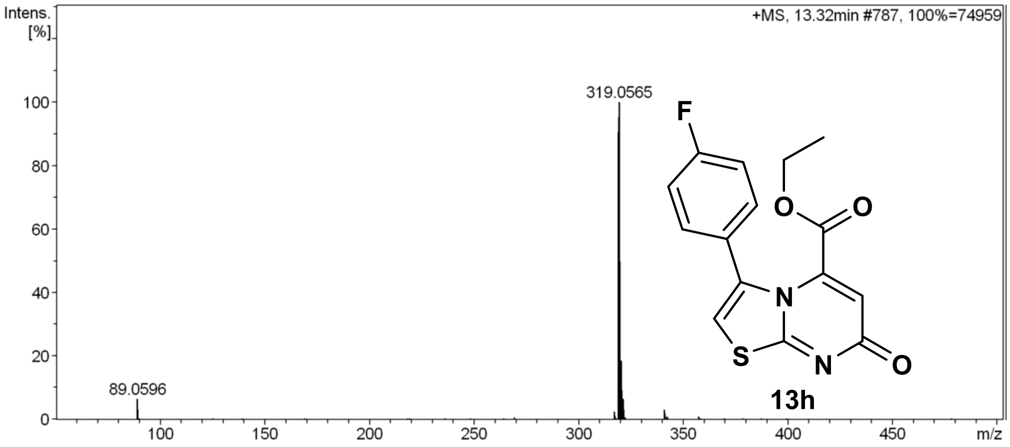
Intens.
[%]
+MS, 13.32min #787, 100%=74959
319.0565
89.0596
0
20
40
60
80
100
100
150
200
250
300
350
400
450
m/z
F
O
O
N
S
N
O
13h

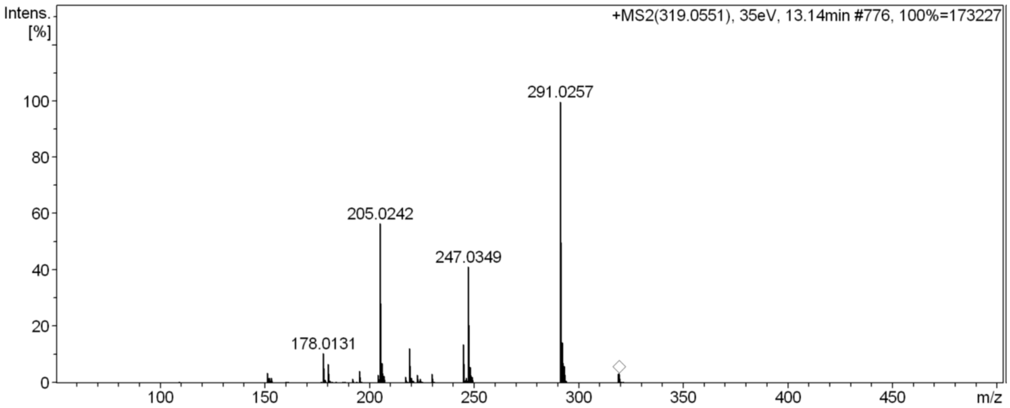
Intens.
[%]
+MS2(319.0551), 35eV, 13.14min #776, 100%=173227
291.0257
205.0242
247.0349
178.0131
0
20
40
60
80
100
100
150
200
250
300
350
400
450
m/z

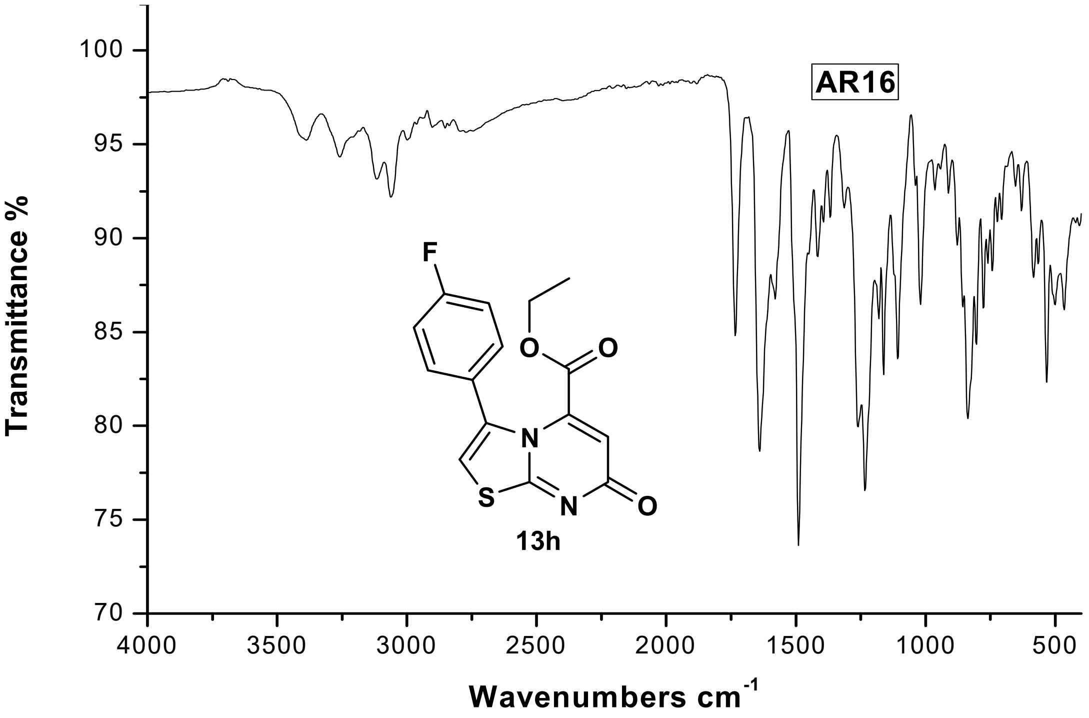
AR16
Transmittance %
100
95
90
85
80
75
70
4000
3500
3000
2500
2000
1500
1000
500
Wavenumbers cm-1
F
O
O
N
S
N
O
13h

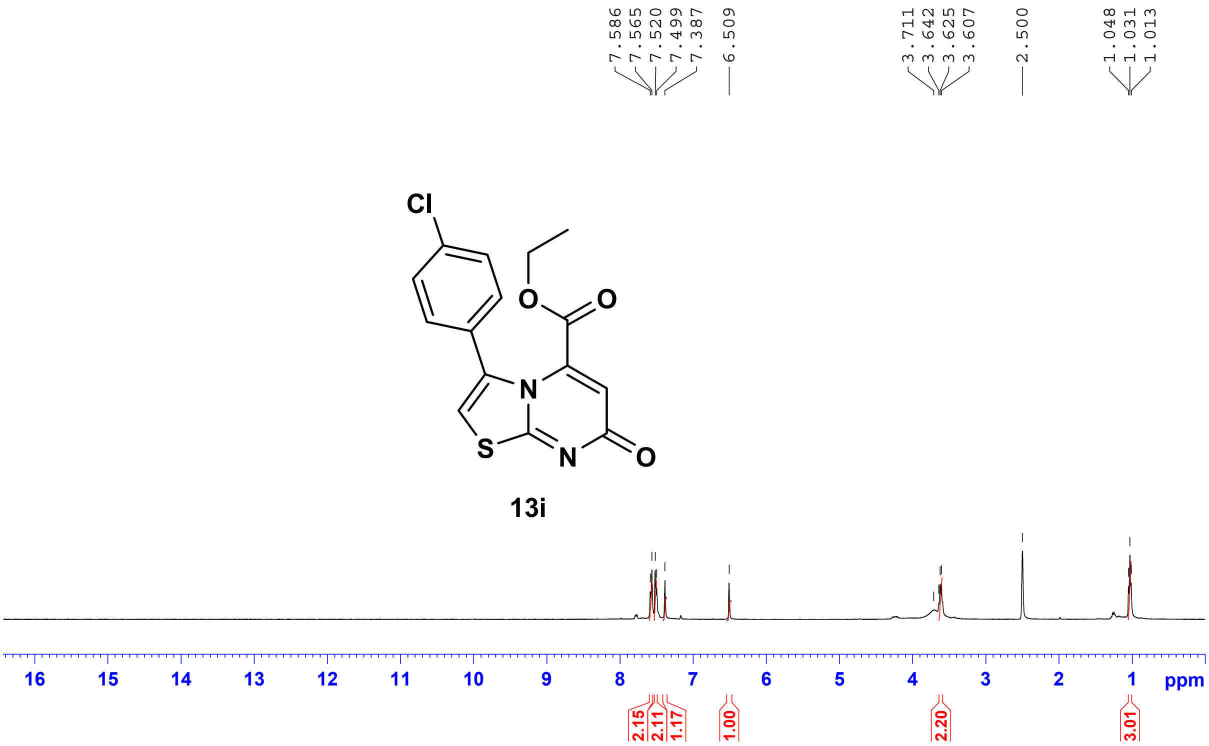
7.586
7.565
7.520
7.499
7.387
6.509
3.711
3.642
3.625
3.607
2.500
1.048
1.031
1.013
Cl
O
O
N
S
N
O
13i
16 15 14 13 12 11 10 9 8 7 6 5 4 3 2 1 ppm
2.15
2.11
1.17
1.00
2.20
3.01

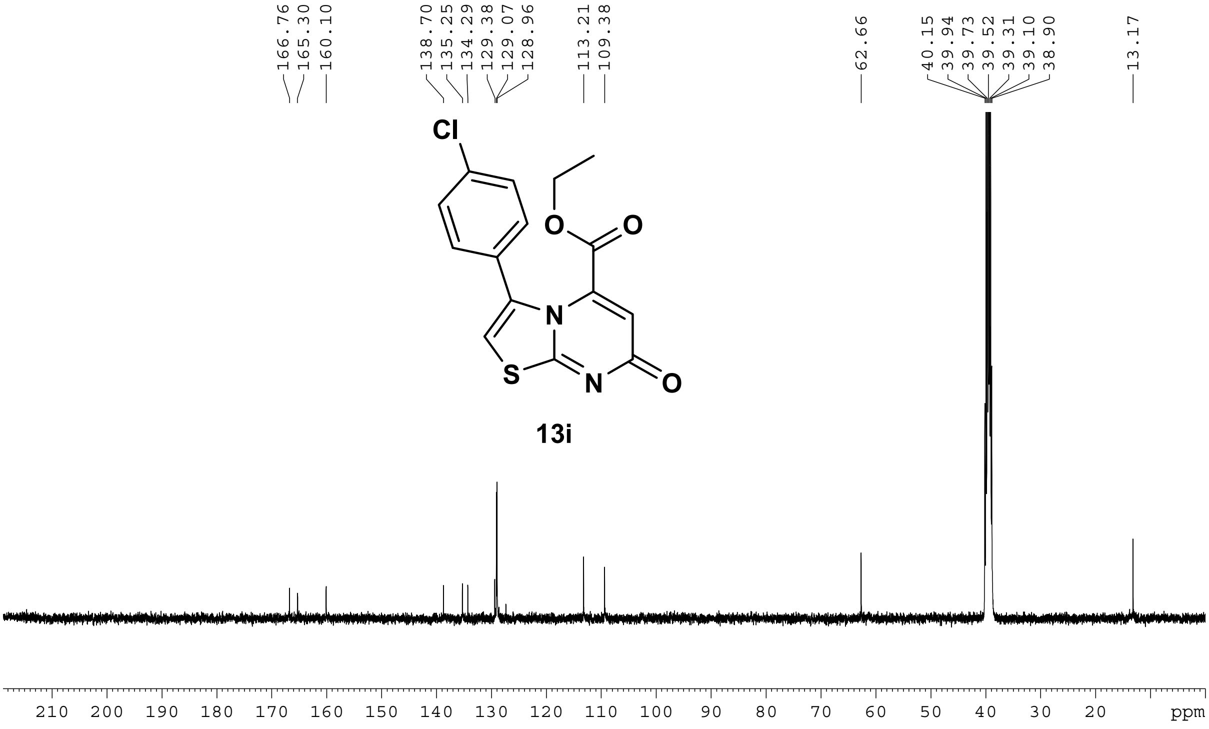
166.76
165.30
160.10
138.70
135.25
134.29
129.38
129.07
128.96
113.21
109.38
62.66
40.15
39.94
39.73
39.52
39.31
39.10
38.90
13.17
Cl
O
O
N
S
N
O
13i
210 200 190 180 170 160 150 140 130 120 110 100 90 80 70 60 50 40 30 20 ppm

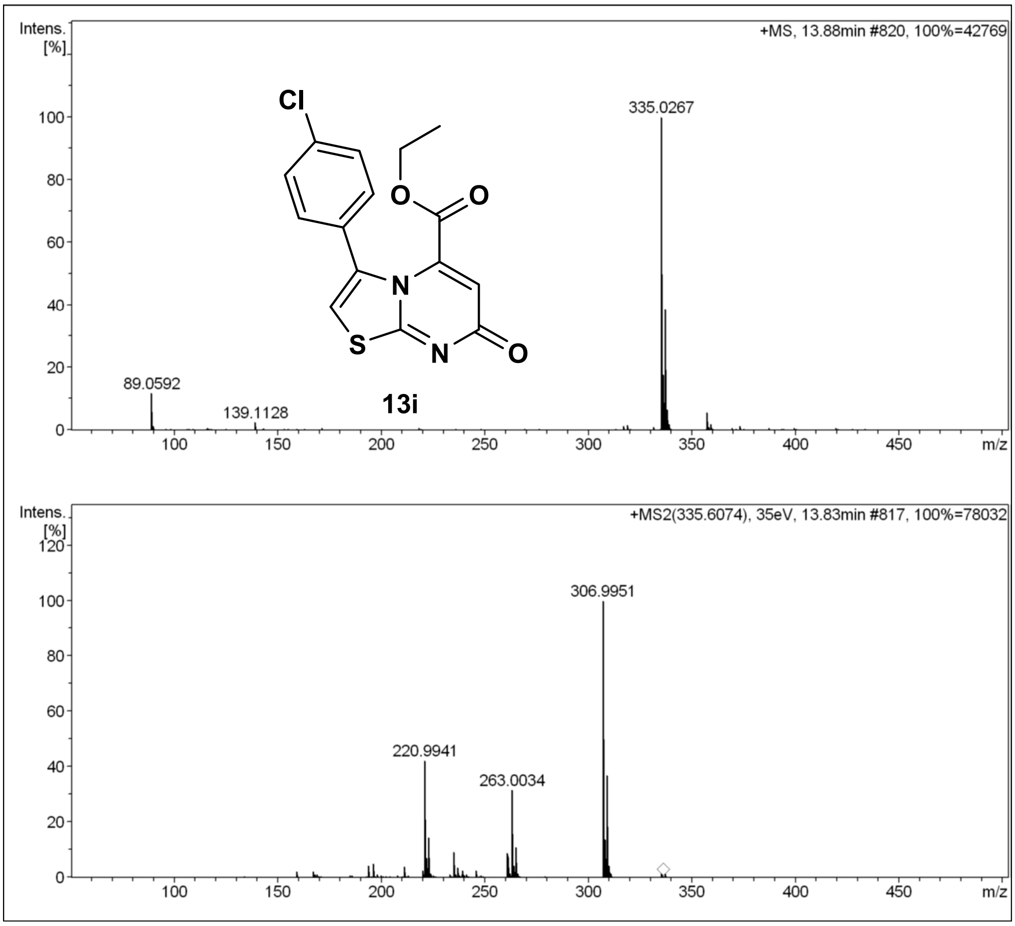

Intens. [%]
+MS, 13.88min #820, 100%=42769
335.0267
89.0592
139.1128
Cl
O
O
N
S
N
O
13i
m/z
Intens. [%]
+MS2(335.6074), 35eV, 13.83min #817, 100%=78032
306.9951
220.9941
263.0034
m/z

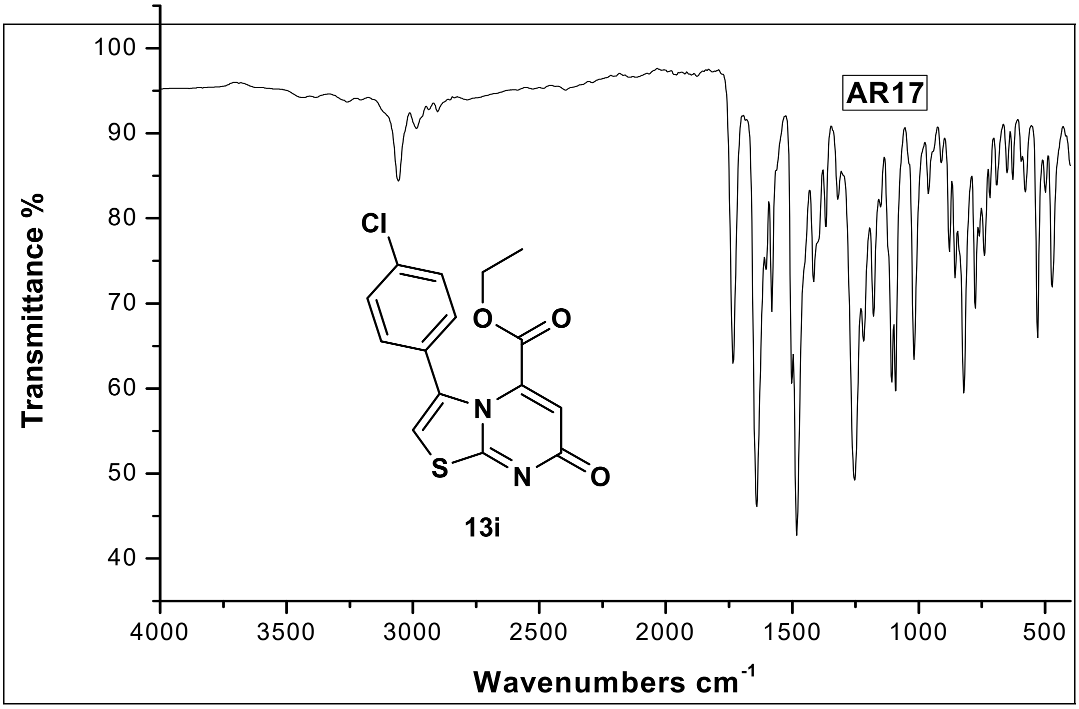

AR17
Transmittance %
Cl
O
O
N
S
N
O
13i
Wavenumbers cm^{-1}

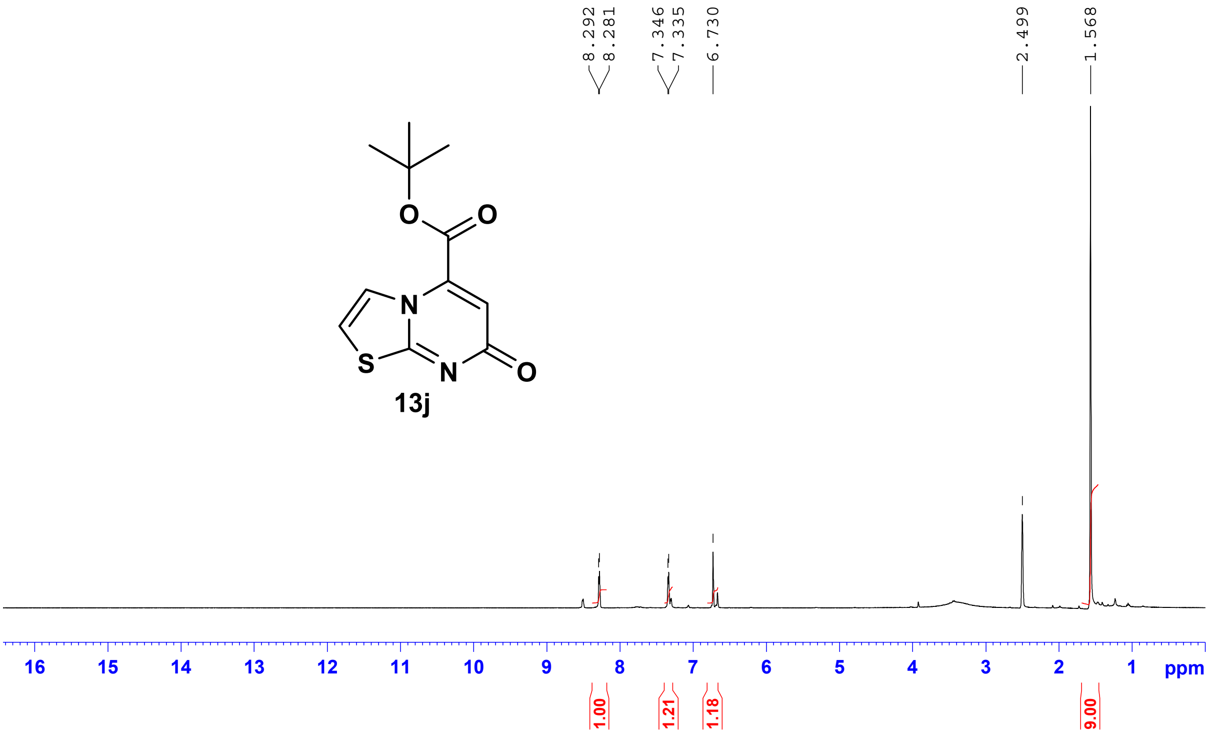
8.292
8.281
7.346
7.335
6.730
2.499
1.568
O
O
N
S
N
O
13j
16 15 14 13 12 11 10 9 8 7 6 5 4 3 2 1 ppm
1.00
1.21
1.18
9.00

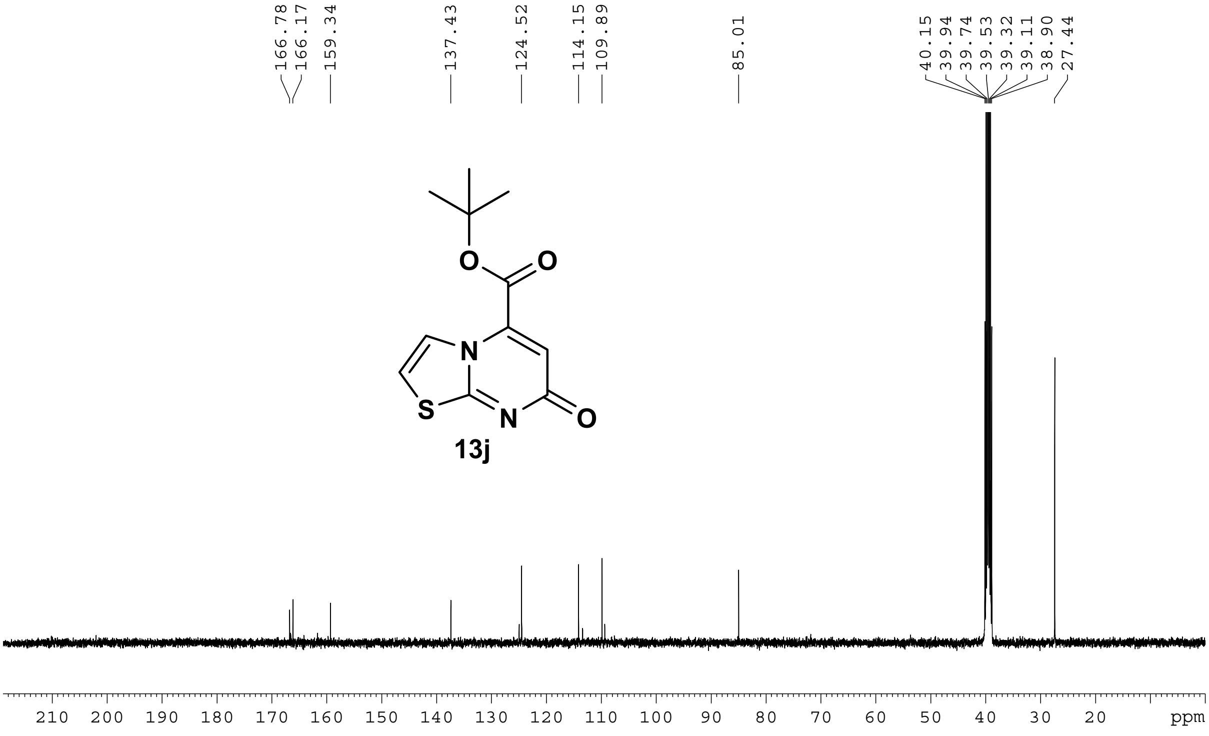
166.78
166.17
159.34
137.43
124.52
114.15
109.89
85.01
40.15
39.94
39.74
39.53
39.32
39.11
38.90
27.44
O
O
N
S
N
O
13j
210 200 190 180 170 160 150 140 130 120 110 100 90 80 70 60 50 40 30 20 ppm

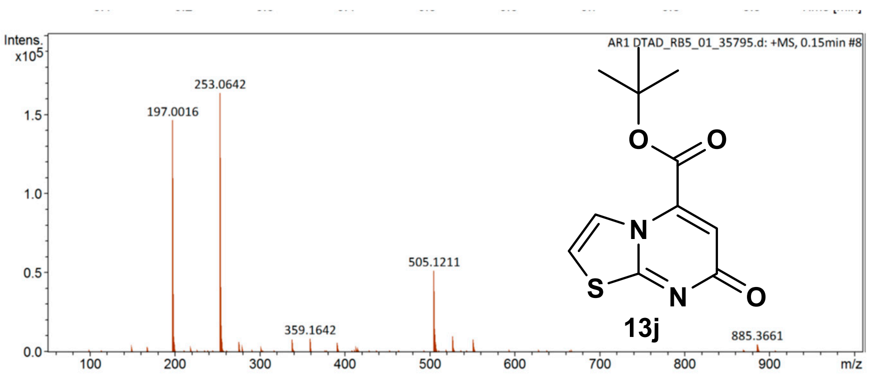
Intens.
x10^5
AR1 DTAD_RB5_01_35795.d: +MS, 0.15min #8
197.0016
253.0642
359.1642
505.1211
885.3661
13j
m/z

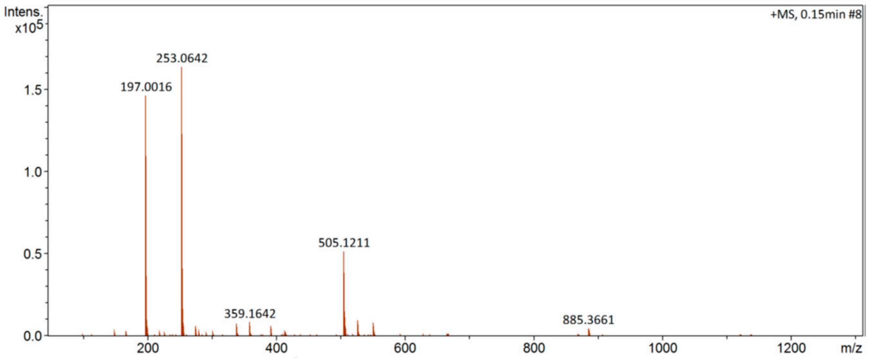
Intens.
x10^5
+MS, 0.15min #8
197.0016
253.0642
359.1642
505.1211
885.3661
m/z

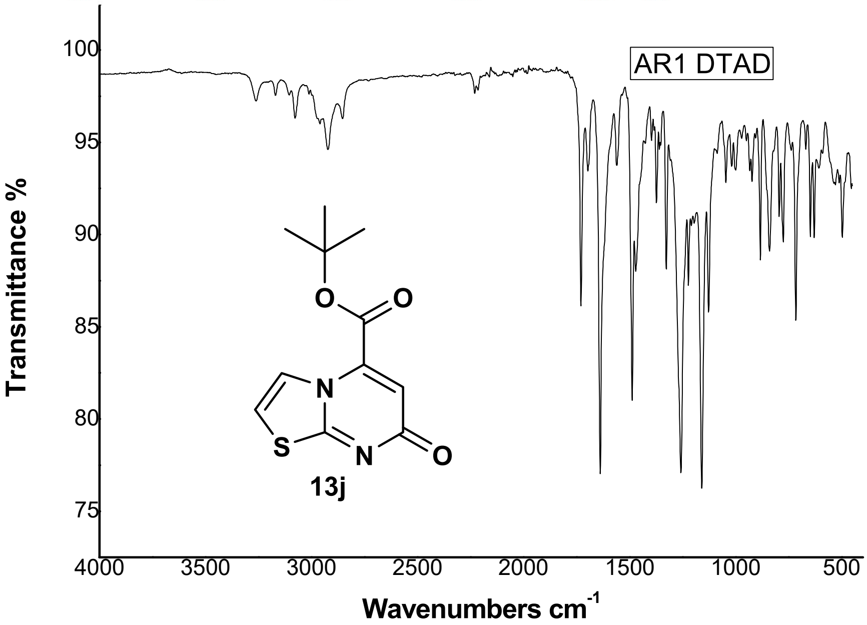
AR1 DTAD
Transmittance %
13j
Wavenumbers cm-1

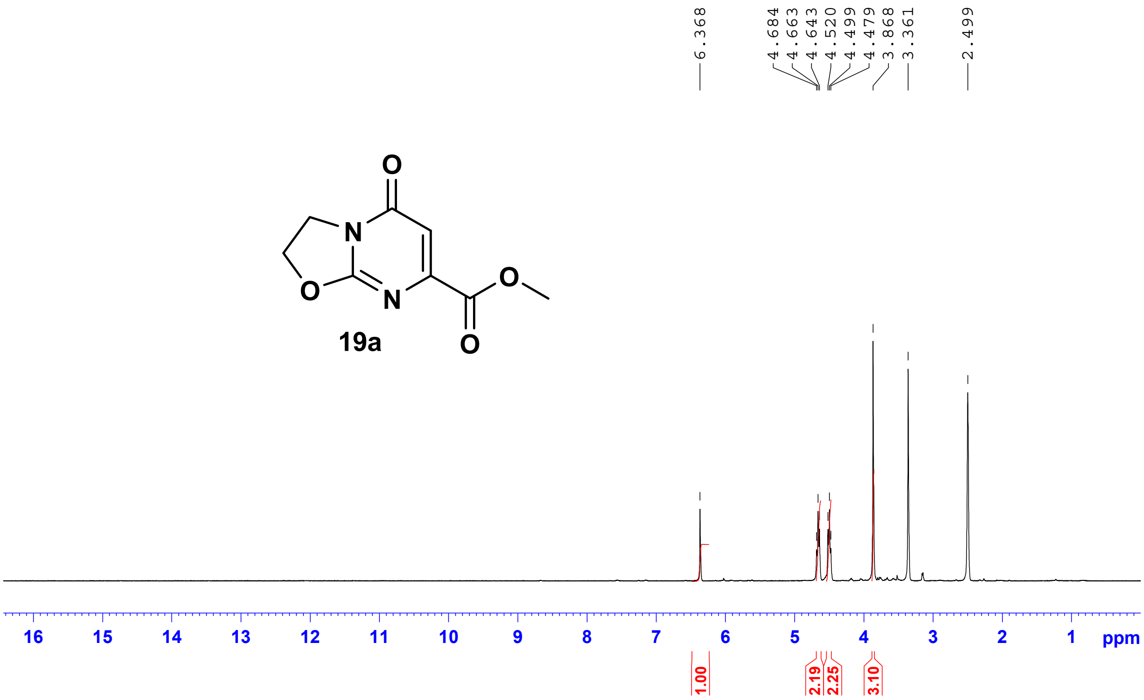

6.368
4.684
4.663
4.643
4.520
4.499
4.479
3.868
3.361
2.499
O
N
O
N
O
O
19a
16
15
14
13
12
11
10
9
8
7
6
5
4
3
2
1
ppm
1.00
2.19
2.25
3.10

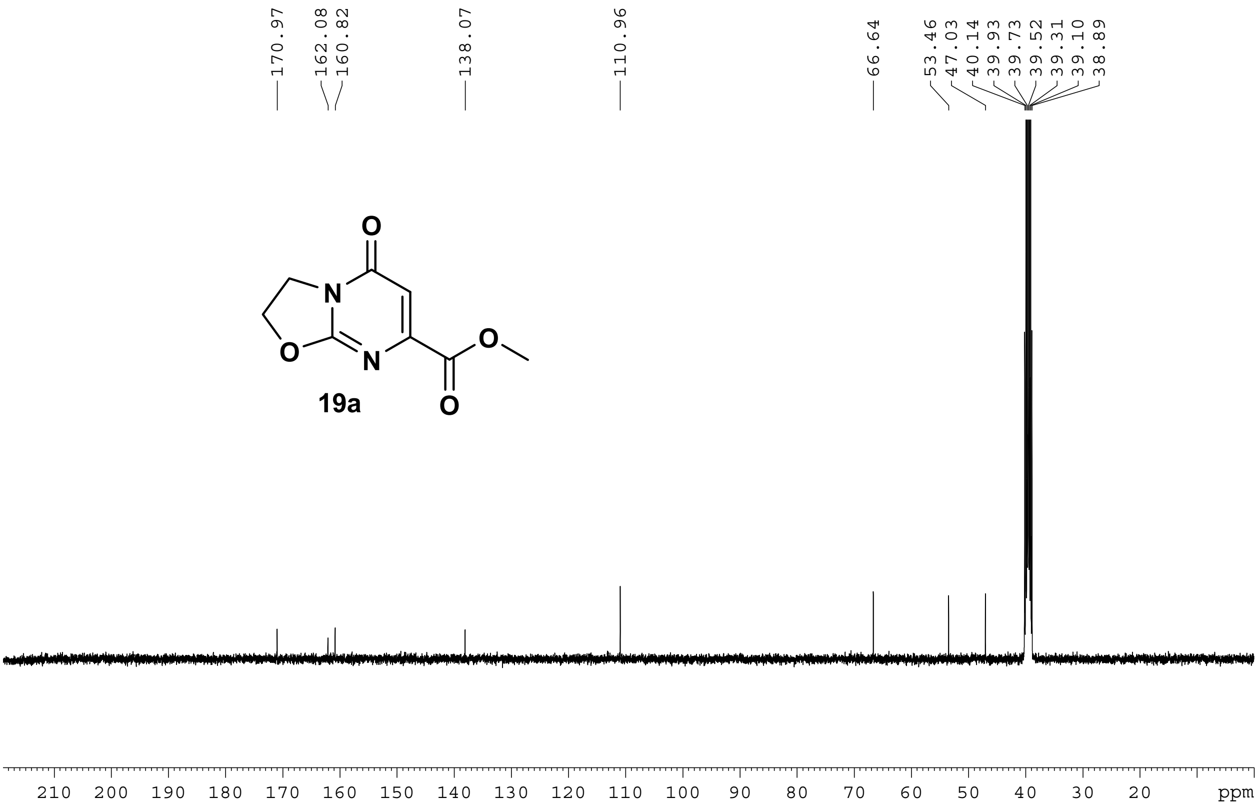
170.97
162.08
160.82
138.07
110.96
66.64
53.46
47.03
40.14
39.93
39.73
39.52
39.31
39.10
38.89
19a
ppm

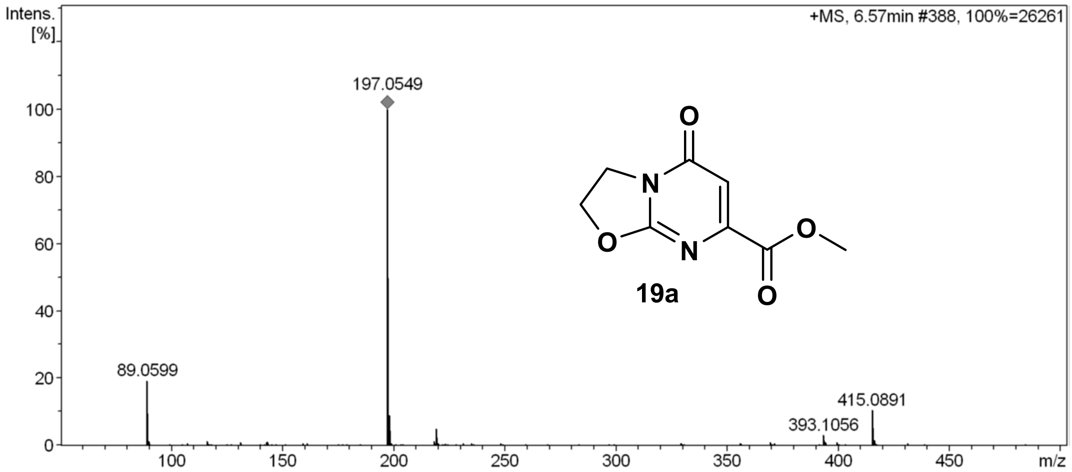
Intens. [%]
+MS, 6.57min #388, 100%=26261
197.0549
89.0599
415.0891
393.1056
19a
m/z

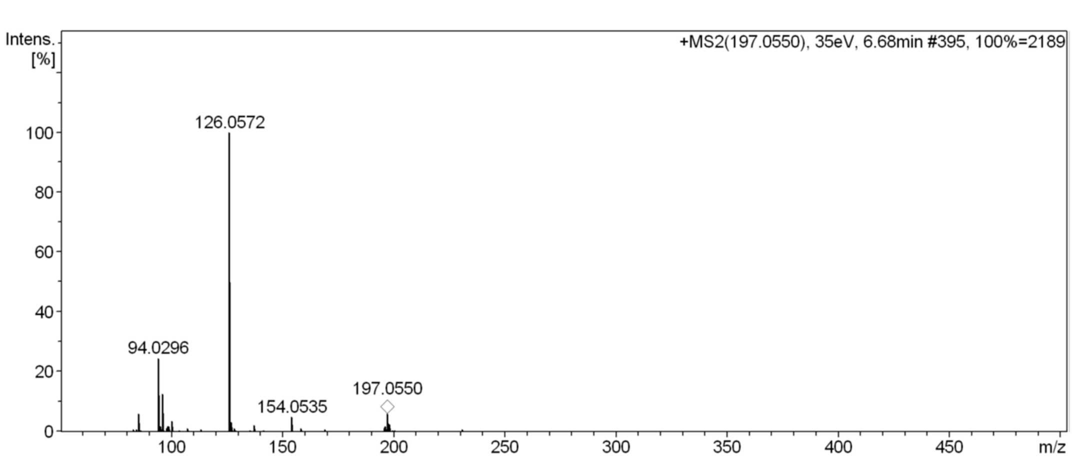
Intens. [%]
+MS2(197.0550), 35eV, 6.68min #395, 100%=2189
126.0572
94.0296
154.0535
197.0550
m/z

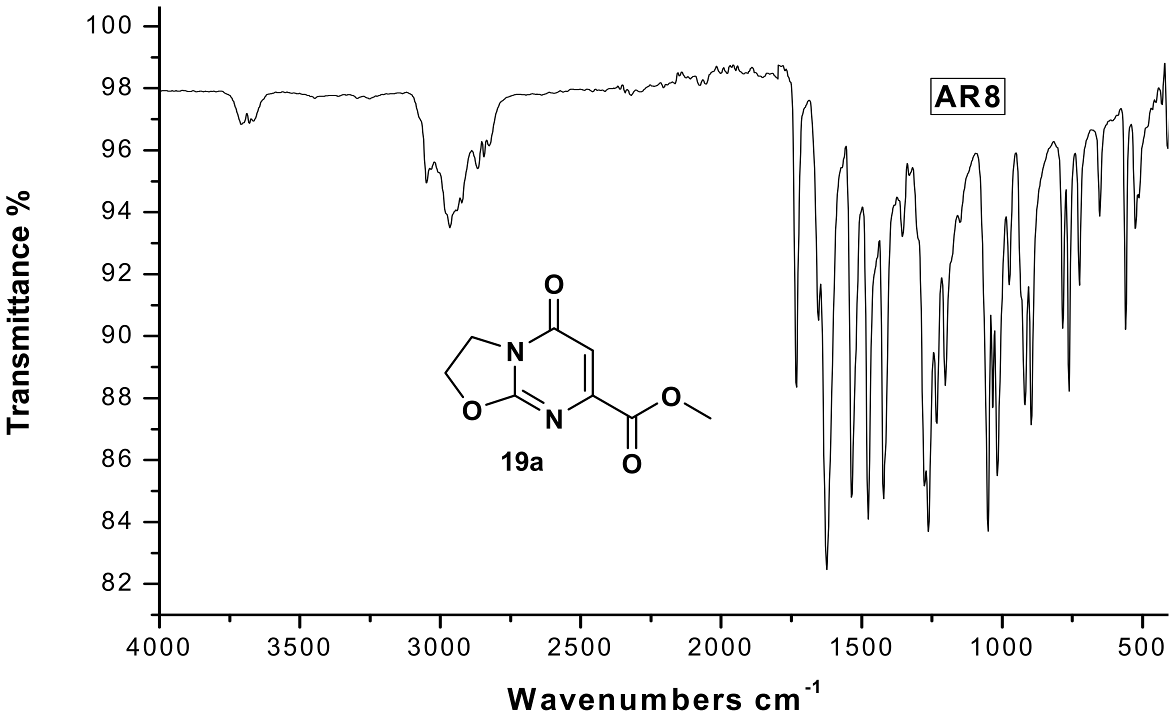
AR8
Transmittance %
Wavenumbers cm-1
19a

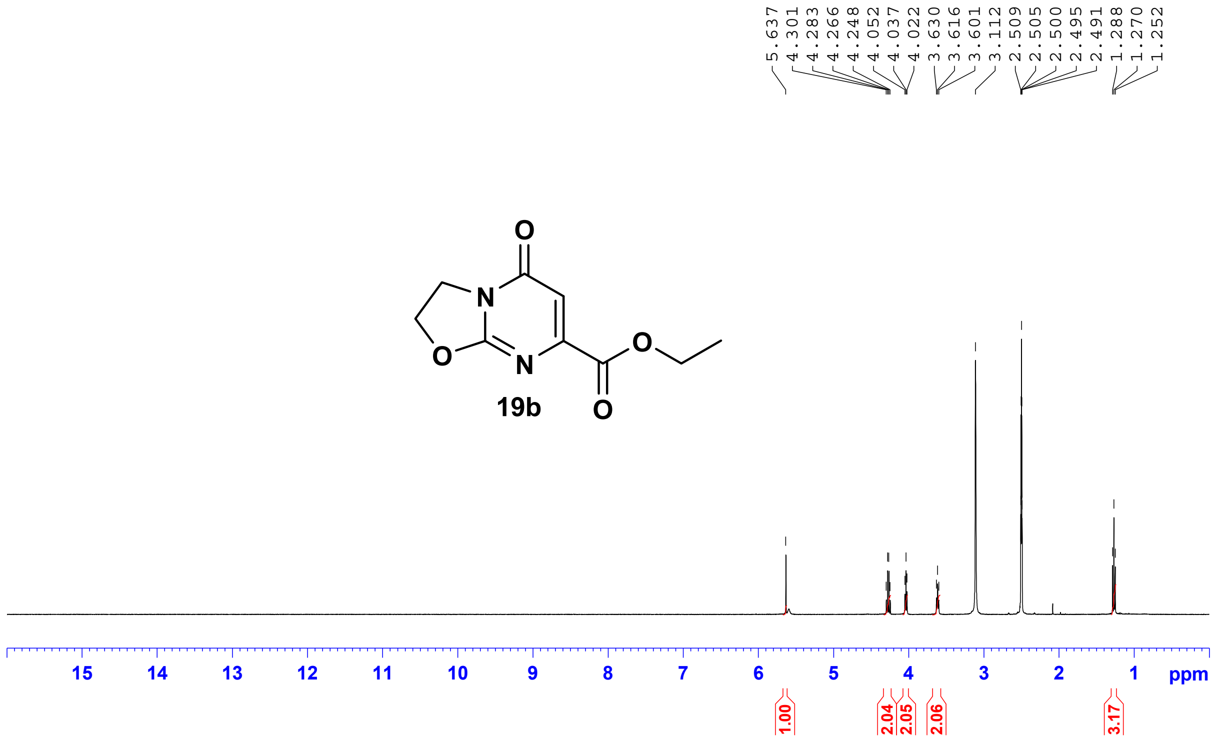
5.637
4.301
4.283
4.266
4.248
4.052
4.037
4.022
3.630
3.616
3.601
3.112
2.509
2.505
2.500
2.495
2.491
1.288
1.270
1.252
19b
ppm
1.00
2.04
2.05
2.06
3.17

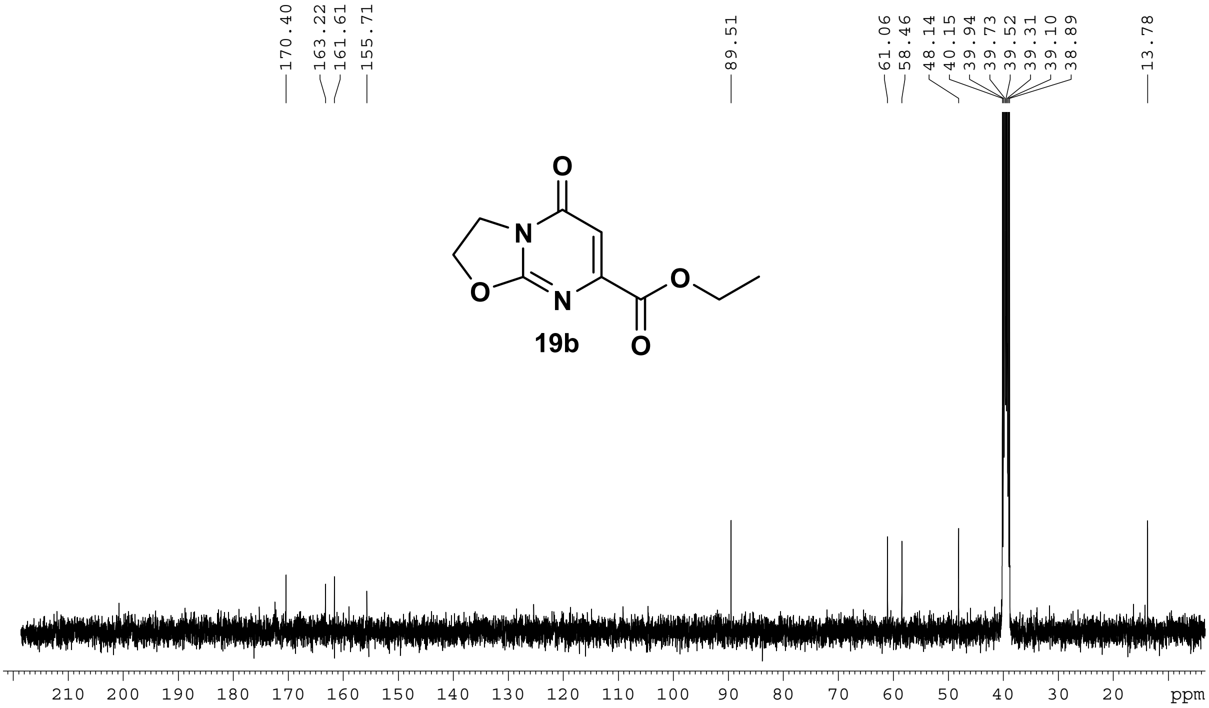

170.40
163.22
161.61
155.71
89.51
61.06
58.46
48.14
40.15
39.94
39.73
39.52
39.31
39.10
38.89
13.78
O
N
O
N
O
O
19b
210 200 190 180 170 160 150 140 130 120 110 100 90 80 70 60 50 40 30 20 ppm

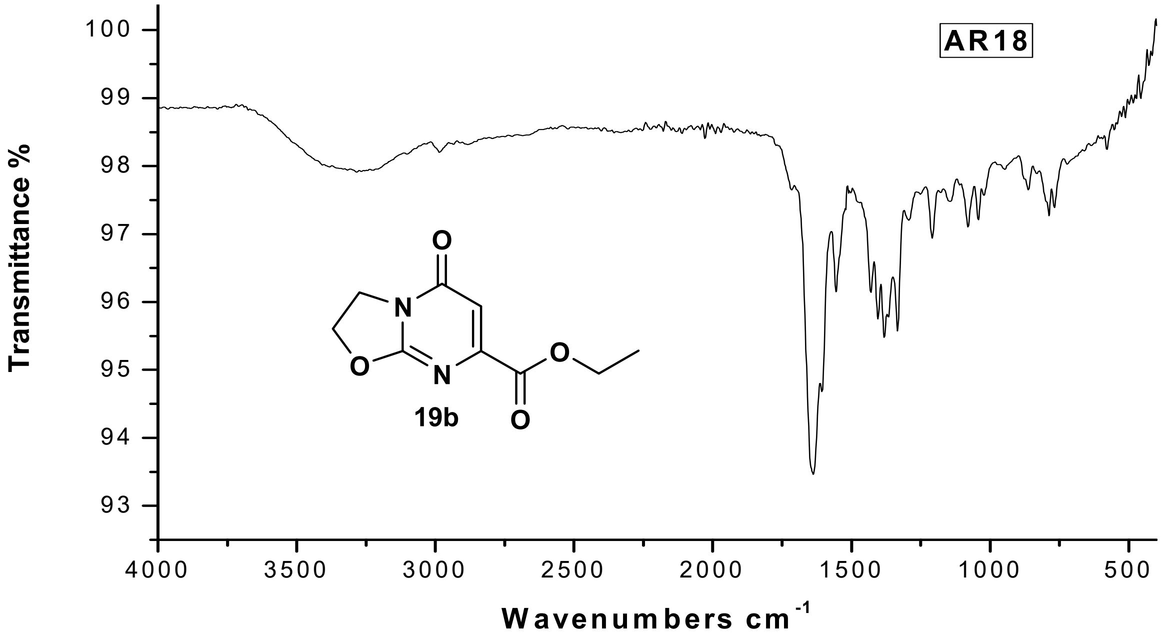

AR18
Transmittance %
100
99
98
97
96
95
94
93
O
N
O
N
O
O
19b
4000 3500 3000 2500 2000 1500 1000 500
Wavenumbers cm-1

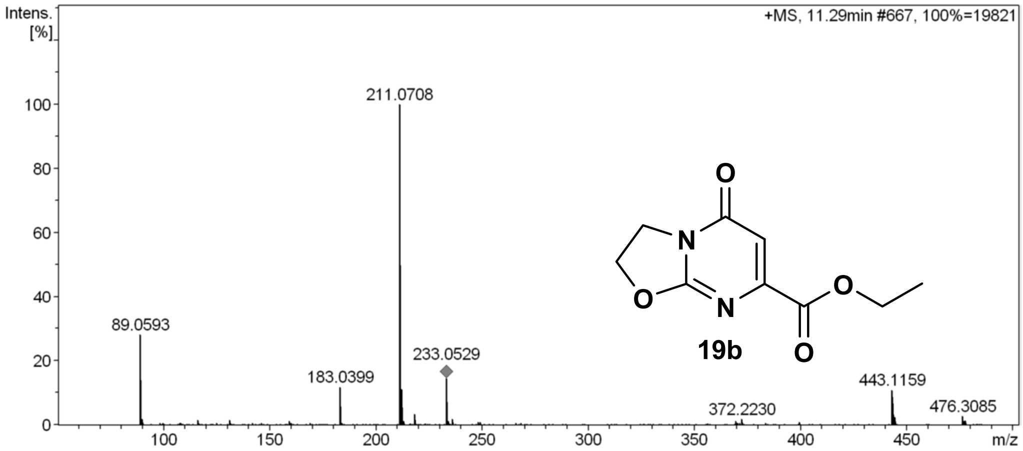
Intens. [%]
+MS, 11.29min #667, 100%=19821
211.0708
89.0593
183.0399
233.0529
372.2230
443.1159
476.3085
m/z
19b

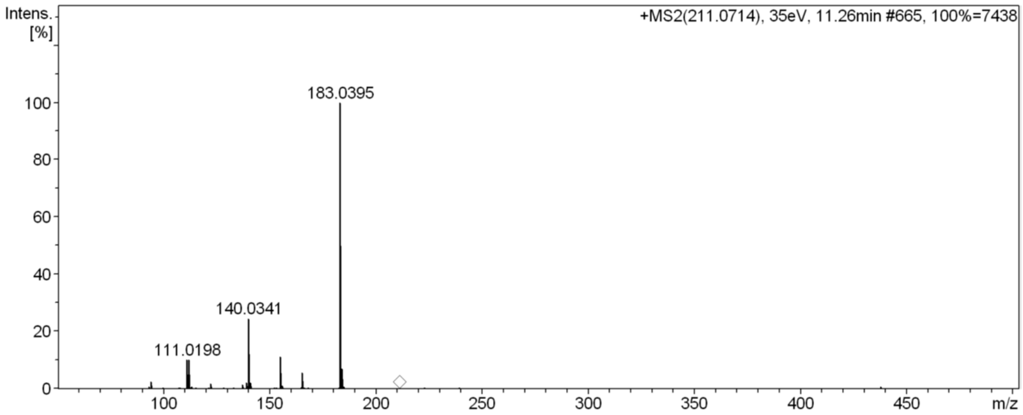
Intens. [%]
+MS2(211.0714), 35eV, 11.26min #665, 100%=7438
183.0395
140.0341
111.0198
m/z

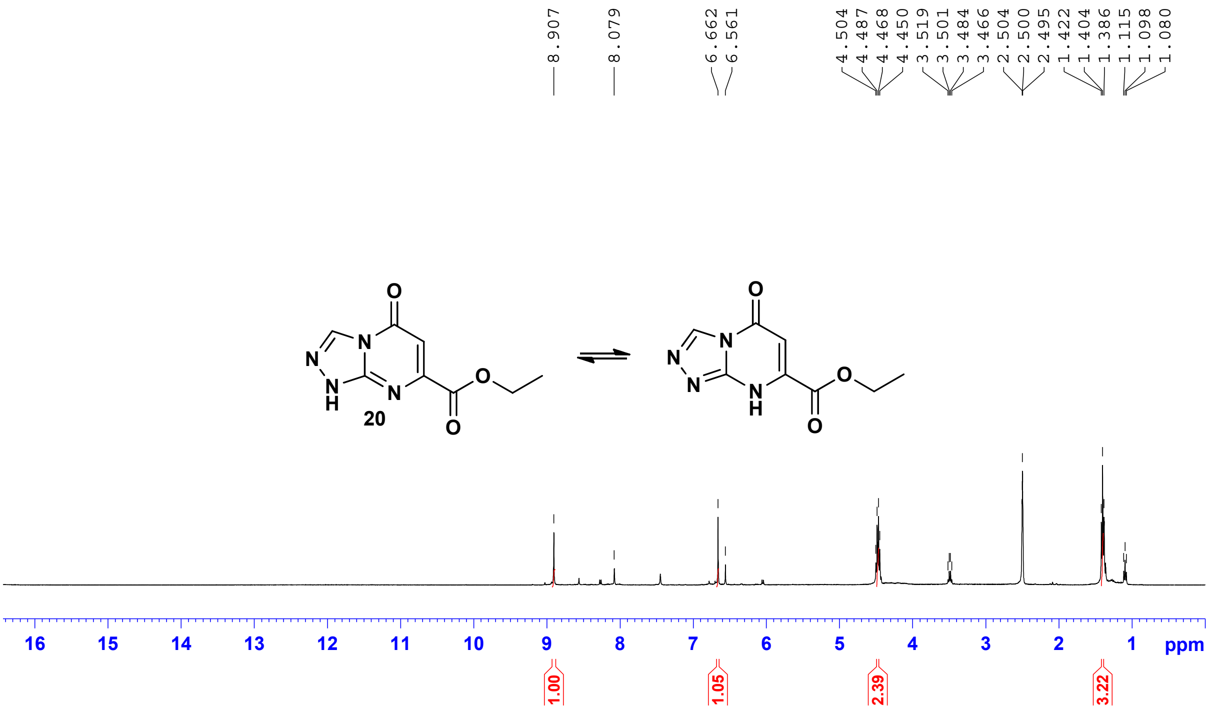
8.907
8.079
6.662
6.561
4.504
4.487
4.468
4.450
3.519
3.501
3.484
3.466
2.504
2.500
2.495
1.422
1.404
1.386
1.115
1.098
1.080
20
ppm
1.00
1.05
2.39
3.22

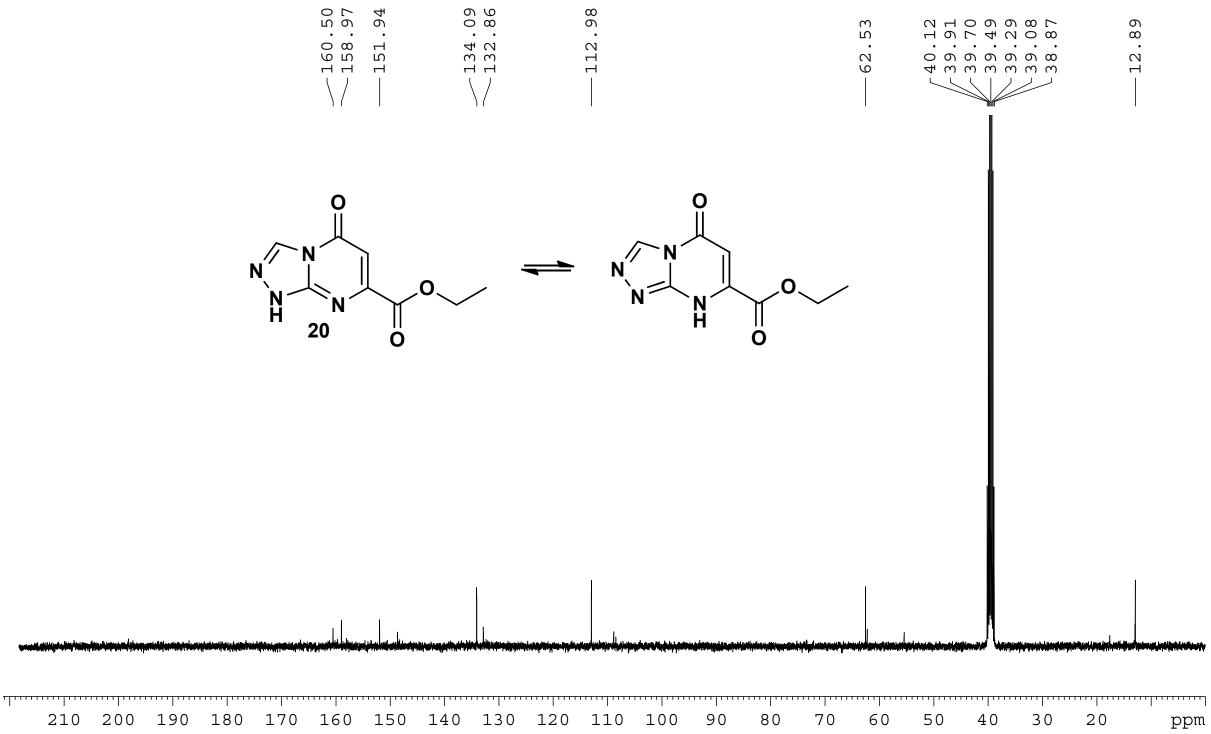
160.50
158.97
151.94
134.09
132.86
112.98
62.53
40.12
39.91
39.70
39.49
39.29
39.08
38.87
12.89
O
N
N
H
N
N
O
O
20
210 200 190 180 170 160 150 140 130 120 110 100 90 80 70 60 50 40 30 20 ppm

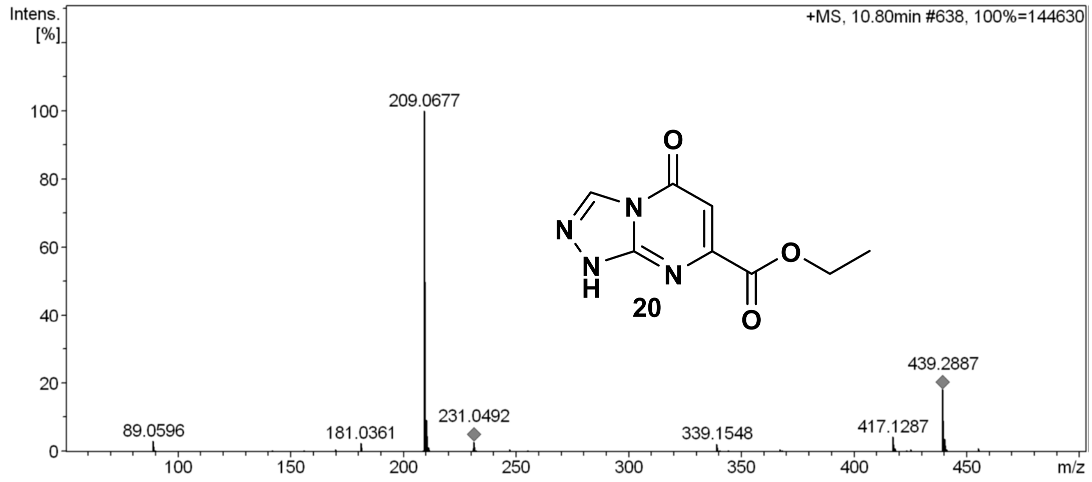
Intens.
[%]
+MS, 10.80min #638, 100%=144630
209.0677
89.0596
181.0361
231.0492
339.1548
417.1287
439.2887
20
100 150 200 250 300 350 400 450 m/z

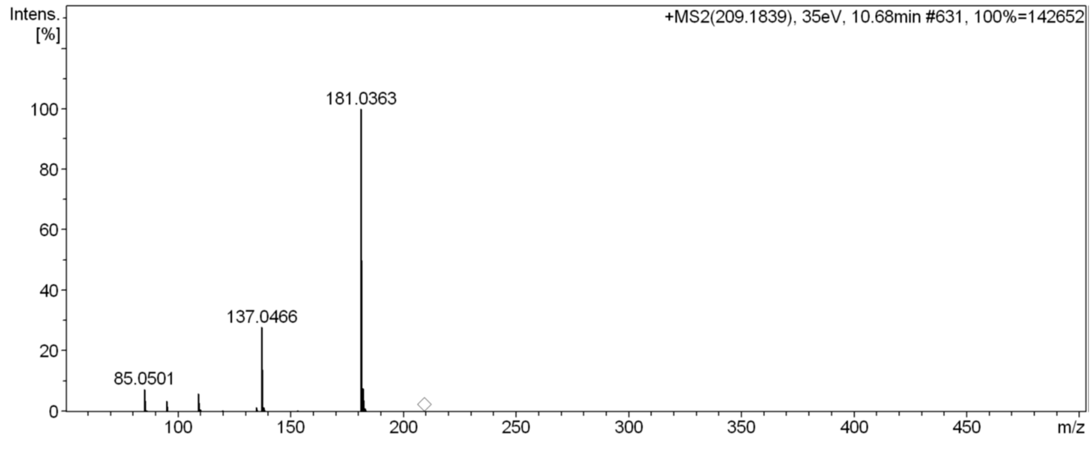
Intens.
[%]
+MS2(209.1839), 35eV, 10.68min #631, 100%=142652
181.0363
137.0466
85.0501
100 150 200 250 300 350 400 450 m/z

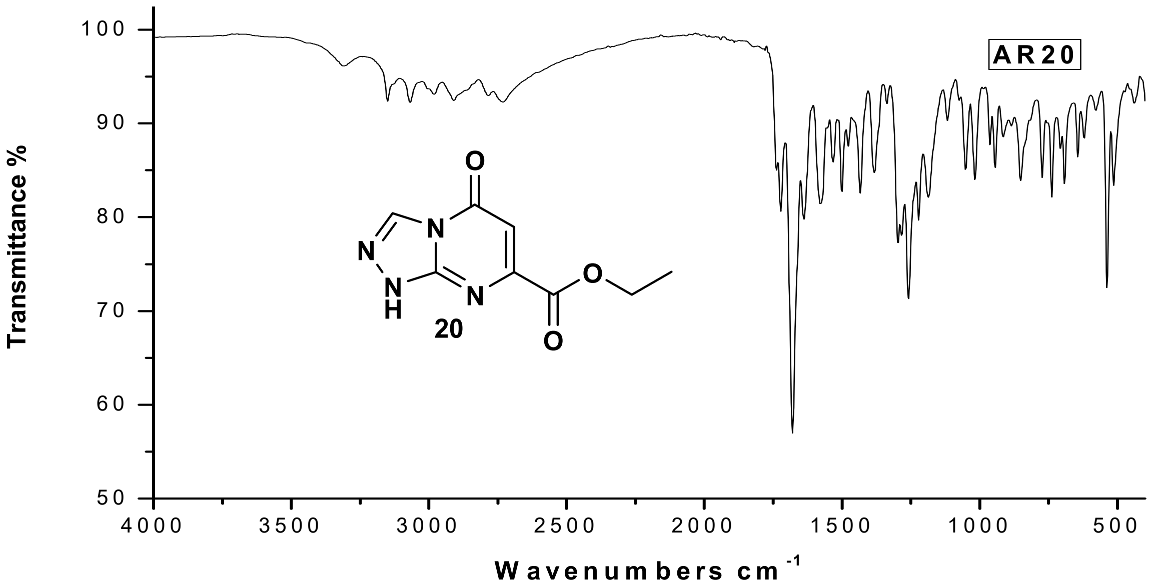
AR20
Transmittance %
Wavenumbers cm -1
100
90
80
70
60
50
4000
3500
3000
2500
2000
1500
1000
500
O
N
N
N
H
N
O
O
20

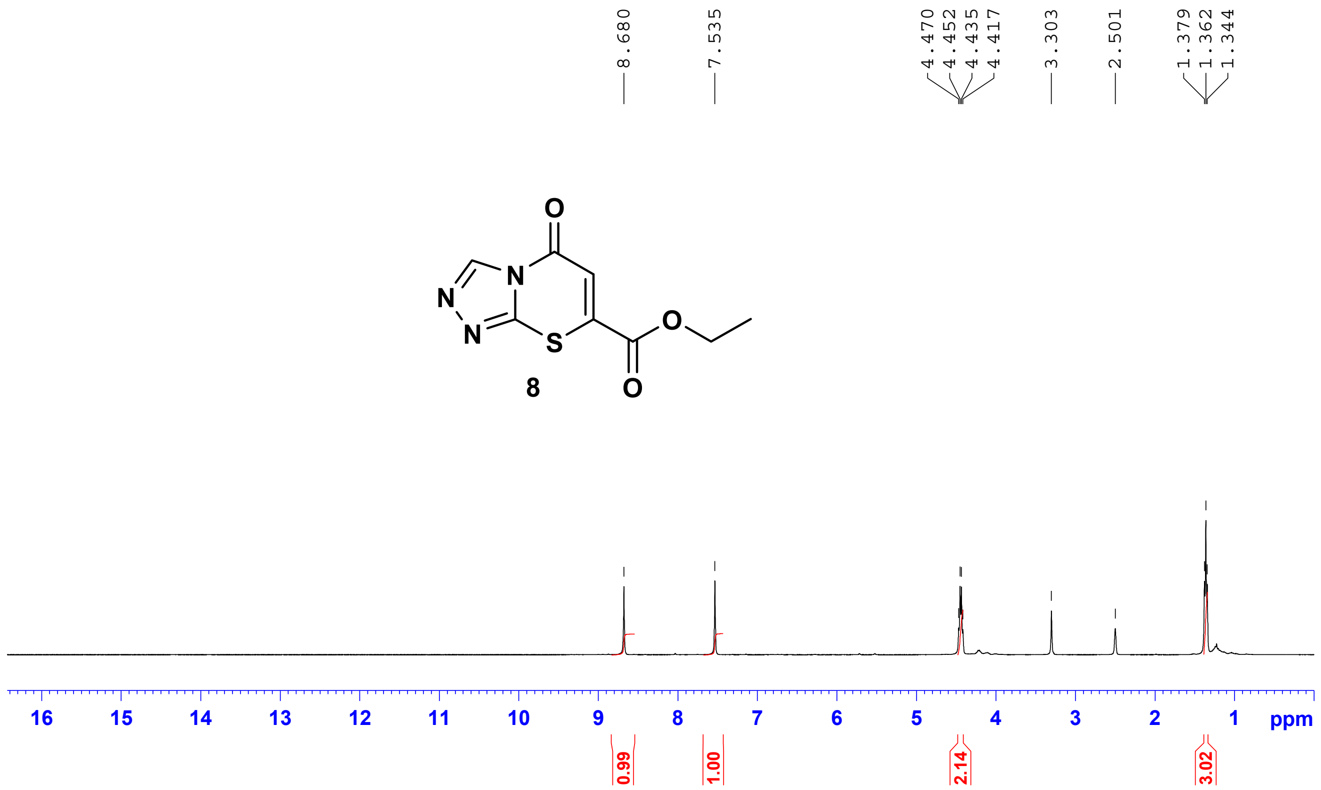
8.680
7.535
4.470
4.452
4.435
4.417
3.303
2.501
1.379
1.362
1.344
O
N
N
N
S
O
O
8
16
15
14
13
12
11
10
9
8
7
6
5
4
3
2
1
ppm
0.99
1.00
2.14
3.02

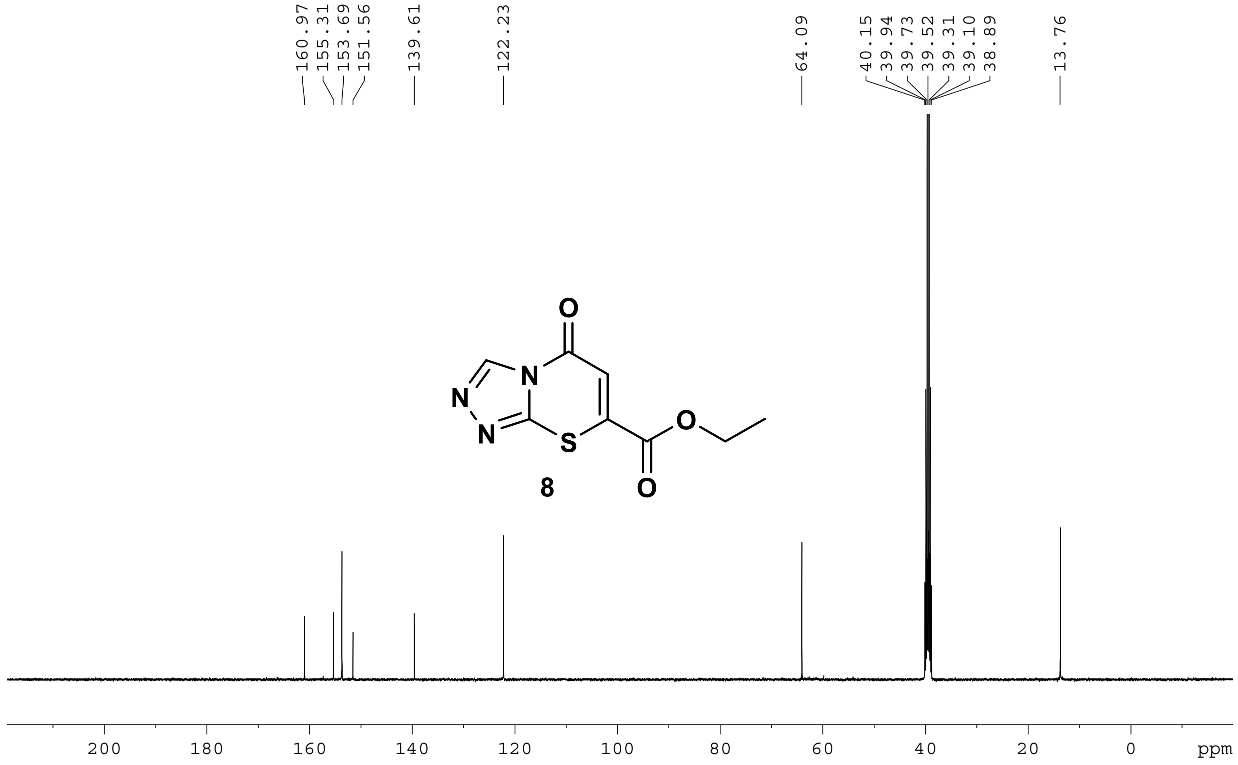
160.97
155.31
153.69
151.56
139.61
122.23
64.09
40.15
39.94
39.73
39.52
39.31
39.10
38.89
13.76
O
N
N
N
S
O
O
8
200
180
160
140
120
100
80
60
40
20
0
ppm

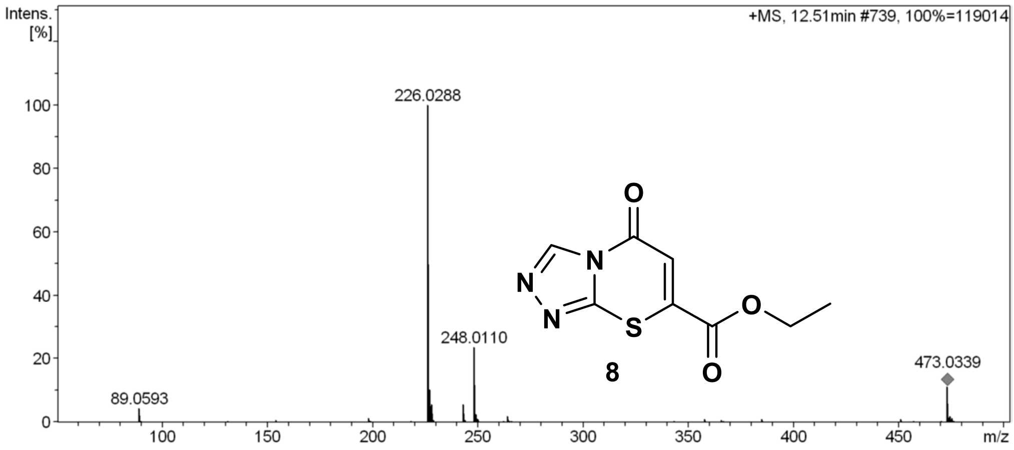
Intens.
[%]
+MS, 12.51min #739, 100%=119014
226.0288
248.0110
473.0339
89.0593
8
m/z

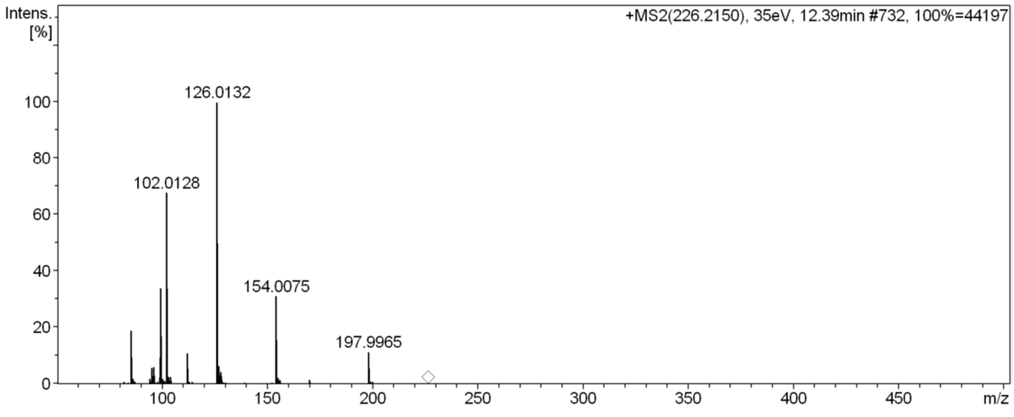
Intens.
[%]
+MS2(226.2150), 35eV, 12.39min #732, 100%=44197
126.0132
102.0128
154.0075
197.9965
m/z

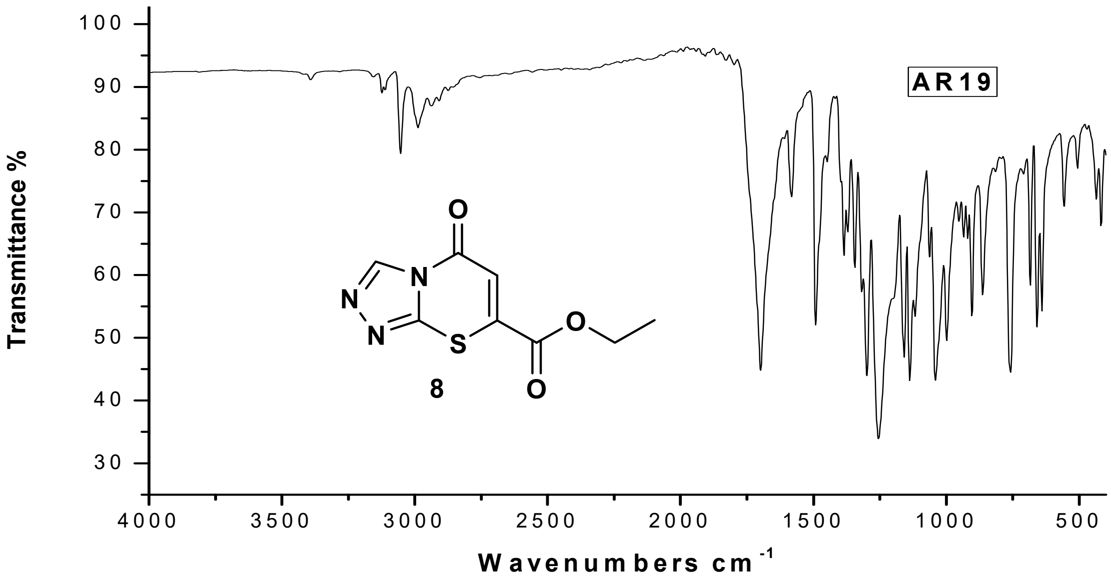

AR19
Transmittance %
Wavenumbers cm -1
100
90
80
70
60
50
40
30
4000
3500
3000
2500
2000
1500
1000
500
O
N
N
N
S
O
O
8

Table S2. Detailed mass spectrometry data of the synthesised compounds

Compound	Chemical Formula [M+1]	Calculated m/z	Measured m/z	Mass error Abs	Mass error (ppm)
**10a**	$C_{15}H_{20}N_3O_5S^+$	354.1118	354.1113	-0.0005	-1.411
**10b**	$C_{17}H_{24}N_3O_5S^+$	382.1431	382.1439	0.0008	2.093
**10c**	$C_{16}H_{22}N_3O_5S^+$	368.1275	368.1279	0.0004	1.086
**10d**	$C_{18}H_{26}N_3O_5S^+$	396.1588	396.1597	0.0009	2.272
**10e**	$C_{20}H_{30}N_3O_5S^+$	424.1901	424.1906	0.0005	1.179
**10f**	$C_{22}H_{34}N_3O_5S^+$	452.2214	452.2223	0.0009	1.990
**10g**	$C_{18}H_{24}N_3O_5S^+$	394.1431	394.1433	0.0002	0.507
**10h**	$C_{20}H_{28}N_3O_5S^+$	422.1744	422.1712	-0.0032	-7.579
**10i**	$C_{17}H_{24}N_3O_5S^+$	382.1430	382.1427	-0.0003	-0.785
**10j**	$C_{19}H_{28}N_3O_5S^+$	410.1744	410.1749	0.0005	1.218
**10k**	$C_{21}H_{32}N_3O_5S$	438.2057	438.2021	-0.0036	-8.215
**10l**	$C_{23}H_{36}N_3O_5S^+$	466.2370	466.2333	-0.0037	-7.935
**10m**	$C_{18}H_{26}N_3O_5S$	396.1588	396.1545	-0.0043	-10.854
**10n**	$C_{20}H_{30}N_3O_5S^+$	424.1901	424.1873	-0.0028	-6.600
**10o**	$C_{22}H_{34}N_3O_5S^+$	452.2214	452.2190	0.0024	1.105
**10p**	$C_{24}H_{38}N_3O_5S^+$	480.2527	480.2526	-0.0001	-0.208
**10q**	$C_{21}H_{24}N_3O_5S^+$	430.1431	430.1445	0.0015	3.254
**10r**	$C_{23}H_{28}N_3O_5S^+$	458.1744	458.1744	0.0000	0.000
**10s**	$C_{25}H_{32}N_3O_5S^+$	486.2057	486.2044	-0.0013	-2.673

**10t**	$C_{27}H_{36}N_3O_5S^+$	514.2370	514.2350	-0.0020	-3.889
**10u**	$C_{23}H_{26}N_3O_5S^+$	456.1588	456.1592	0.0004	0.876
**13a**	$C_{10}H_{11}N_2O_3S^+$	239.0485	239.0486	0.0001	0.418
**13b**	$C_{15}H_{13}N_2O_3S^+$	301.0641	301.0648.	0.0007	2.325
**13c**	$C_{14}H_{10}FN_2O_3S^+$	305.0391	305.0391	0.0000	0.000
**13d**	$C_{14}H_{10}ClN_2O_3S^+$	321.0095	321.0087	-0.0008	-2.492
**13e**	$C_9H_9N_2O_3S^{+\ +}$	225.0328	225.0339	0.0011	4.888
**13f**	$C_{10}H_{11}N_2O_3S^+$	239.0485	239.0497	0.0012	5.019
**13g**	$C_{11}H_{13}N_2O_3S^+$	253.0641	253.0652	0.0011	4.346
**13h**	$C_{15}H_{12}FN_2O_3S^+$	319.0547	319.0565.	0.0018	5.641
**13i**	$C_{15}H_{12}ClN_2O_3S^+$	335.0252	335.0267	0.0015	4.477
**13j**	$C_{11}H_{13}N_2O_3S^+$	253.0641	253.0642	0.0001	0.395
**19a**	$C_8H_9N_2O_4^+$	197.0557	197.0549	-0.0008	-4.059
**19b**	$C_9H_{11}N_2O_4^+$	211.0713	211.0708	-0.0005	-2.368
**20**	$C_8H_9N_4O_3^+$	209.0669	209.0677	0.0008	3.826
**5**	$C_8H_8N_3O_3S^+$	226.0281	226.0288.	0.0007	3.096

References

1. Oda, T., Watanabe, S.; Nakamura, T. Type C virus particles produced in human T-cell lines derived from acute lymphoblastic leukemia and a leukemic T-lymphoid malignancy, Acta Med Okayama., 1983, 37, 529-33.

2. Varghese, E.; Büsselberg, D. Auranofin, an Anti-Rheumatic Gold Compound, Modulates Apoptosis by Elevating the Intracellular Calcium Concentration ([Ca2+]i) in MCF-7 Breast Cancer Cells, Cancers, 2014, 6, 2243-2258.

3. Folks T.M., Clouse K.A., Justement J., Rabsonf A., Duho E., Kehrl J.H., Fauci A.S., Proc. Natl Acad. Sci. USA, 1989, 86, 2365-2368; doi:10.1073/pnas.86.7.2365.

4. Rosenwirth B., Billich A., Datema R., Donatsch P., Hammerschmid F., Harrison R., Hiestand P., Jaksche H., Mayer P., Peichl P., Quesniaux V., Schatz F., Schuurman H.-J., Traber R., Wenger R., Wolff B., Zenke G, Zurini M., Antimicrob. Agents Chemother., 1994, 38, 1763-72; doi: 10.1128/aac.38.8.1763.
